# Advanced assessment through intact glycopeptide analysis of Infliximab’s biologics and biosimilar

**DOI:** 10.3389/fmolb.2022.1006866

**Published:** 2022-11-29

**Authors:** Hyejin Kim, Geul Bang, Ye Eun Park, Moonhee Park, Jung Hoon Choi, Myung Jin Oh, Hyun Joo An, Jong Shin Yoo, Youngja Hwang Park, Jin Young Kim, Heeyoun Hwang

**Affiliations:** ^1^ Research Center for Bioconvergence Analysis, Korea Basic Science Institute, Cheongju, South Korea; ^2^ Department of Bio and Brain Engineering, Korea Advanced Institute of Science and Technology, Daejeon, South Korea; ^3^ Metabolomics Laboratory, College of Pharmacy, Korea University, Sejong, South Korea; ^4^ Department of Bio-Chemical Analysis, Korea Basic Science Institute, Cheongju, South Korea; ^5^ Graduate School of Analytical Science and Technology, Chungnam National University, Daejeon, South Korea; ^6^ Asia-Pacific Glycomics Reference Site, Chungnam National University, Daejeon, South Korea

**Keywords:** monoclonal antibody, post-translational modification, ultra-high resolution mass spectrometry, infliximab, glycopeptide

## Abstract

Characterization of therapeutic monoclonal antibodies (mAbs) represents a major challenge for analytical sciences due to their heterogeneity associated with post-translational modifications (PTMs). The protein glycosylation requires comprehensive identification, which could influence on the mAbs’ structure and their function. Here, we demonstrated high-resolution tandem mass spectrometry with an ultra-high-performance liquid chromatography for characterization and comparison between biologics and biosimilar of infliximab at an advanced level. Comparing the N- and O-glycopeptides profiles, a total of 49 and 54 glycopeptides was identified for each product of the biologics and biosimilar, respectively. We also discovered one novel N-glycosylation site at the light chain from both biopharmaceuticals and one novel O-glycopeptide at the heavy chain from only biosimilar. Site-specific glycopeptide analysis process will be a robust and useful technique for evaluating therapeutic mAbs and complex glycoprotein products.

## Introduction

Biosimilars are defined as biological medicinal products that are highly similar to and have no clinically meaningful differences from existing reference products. Over the past 10 years, the creation of biosimilar medications has increased globally in an effort to decrease the financial burden on the healthcare system associated with expensive biologic medications with originator. Biologics and biosimilar products represent heterogeneous variants characterized by differences in glycosylation, oxidation, glycation, aggregation state, and deamidation. Glycosylation is an important post-translational protein modification that alters protein properties, including the pharmacokinetics (PK), pharmacodynamics (PD), effector functions, solubility, and stability (Arnold et al., 2007; Liu, 2015; Higel et al., 2016). Even slight differences in glycosylation patterns can induce immunogenicity; therefore, consistency in glycosylation is necessary to demonstrate a drug’s safety and efficacy and to determine whether a biosimilar candidate is highly similar to the reference product. For example, presence of high mannose type N-glycans in therapeutic mAbs leads to a decreased half-life in humans (Goetze et al., 2011). N-glycolyl-neuraminic acid (NeuGc), a sialic acid found in mammal other than human, can cause an immune response in human (Padler-Karavani and Varki, 2011). Hence, the comparison of glycosylation in the biologics and biosimilar product is critical for ensuring a similar level of efficacy and safety (Pisupati et al., 2017).

For the evaluation of therapeutic mAbs, the glycan analysis with LC-MS/MS has used the releasing method of N- and O-glycans from proteins by peptide-N-glycosidase F (PNGase F) and alkaline β-elimination, respectively. However, such analyses do not provide information on the glycosylation site in a protein, because they are performed with the cleaved glycans from the mAbs (Fekete et al., 2016). In other hand, a top-down approach characterizes glycosylation by analyzing the intact protein using LC-MS, which provides only information on the level of total glycosylation (Fekete et al., 2016; Zhang et al., 2016). A third, middle-down approach, analyzes protein fragments; mAbs are treated with immunoglobulin G-degrading enzyme to cleave heavy chains below the hinge region or dithiothreitol (DTT) to reduce disulfide bonds. As middle-down analysis often results in protein fragments with high masses, fewer glycan compositions can be distinguished due to the lack of resolution as compared with other mass spectrometry (MS)-based methods (De Leoz et al., 2020). Finally, glycopeptide analysis is considered as a bottom-up approach, where the glycopeptides produced by enzyme digestion of glycoprotein are analyzed using ESI-MS or MALDI-MS. Tandem MS analyses of glycopeptides, particularly, can reveal the glycosylation site, glycan composition, and glycan structure (Desaire, 2013; Zhang et al., 2016; Oh et al., 2022).

Infliximab is a representative mAbs that interacts with target tumor necrosis factor α (TNF-α) for treating autoimmune diseases, such as rheumatoid arthritis, psoriatic arthritis, ankylosing spondylitis, adult plaque psoriasis, pediatric and adult ulcerative colitis and Crohn’s disease (Scallon et al., 2002; Willrich et al., 2015) This drug is based on a combination of murine variable regions and the human Fc region of IgG1 expressed in a Sp2/0 cell line. There is one N-glycosylation site in the Fc region of infliximab (Lee et al., 2017). In a previous study, 28 glycan forms were identified in infliximab (Pisupati et al., 2017).

In this study, N-linked glycosylation of the biologics and biosimilar of infliximab, respectively, were analyzed at the glycopeptide level with a focus on profiling the glycan composition through tandem MS spectra. Based on the number of identified glycopeptide spectra matches (GSMs), the originator and biosimilar of Infliximab were compared quantitatively and the similarity between of them was evaluated.

## Materials and methods

### Sample preparation

Each five batches of the Infliximab biologics of Remicade® (LOT#GBL15016, GGL49016, GKL81014, HEL20014, and ICL88011, Janssen) and its corresponding biosimilar of Remsima® (LOT#14B1C002BA1, 15B1C01BA1, 15B1C05BA1, 15B1C07BA1, and 18B1C23BA1, Celltrion) were dissolved in water and diluted to achieve a concentration of 20 μg/μL. Subsequently, 50 μl of this drug solution was buffer-exchanged into 50 mM ammonium bicarbonate (ABC, 1,066-33-7, Sigma-Aldrich) buffer using a 10 kDa MWCO Amicon Ultra Centrifugal Filter (UFC501096, Millipore). Each drug was quantified with a Qubit Protein Assay Kit (Q33212, Invitrogen) and then diluted with 50 mM ABC buffer to achieve a concentration of 1 μg/μL. Then, 30 μg samples were denaturized for 5 min at 90°C. The samples were, then, reduced with 20 mM 1,4-dithiothreitol (DTT, 3483-12–3, Sigma-Aldrich) for 1 h at 60°C, alkylated with 40 mM iodoacetamide (IAA, 114-48-9, Sigma-Aldrich) for 45 min at room temperature in the dark, and digested with 1.5 μg of trypsin (V528A, Promega) overnight at 37°C. Subsequently, tryptic peptides were dried by SpeedVac. Enrichment of glycopeptides was performed with the ZIC-HILIC ProteoExtract^®^ Glycopeptide Enrichment Kit (72103-3, Novagen) in accordance with the manufacturer’s protocol and dried by SpeedVac. Dried glycopeptides of the therapeutic mAbs were dissolved in 30 μl H_2_O/FA (99.9:0.1, v:v).

### Nano LC-ESI-MS/MS analysis

A nano-flow ultra-high-performance liquid chromatography (UHPLC) system (EASY-nLC™ 1,200 System, Thermo Fisher Scientific, San Jose, CA, United States) coupled to a Thermo Scientific Orbitrap Fusion Tribrid™ mass spectrometer was used for all experiments for the analyses of infliximab glycopeptides. Two or 5 μl of tryptic glycopeptides (1 μg/μL) were injected and separated on EASY-Spray PepMap™ RSLC C18 Column ES803A (2 μm, 100 Å, 75 μm × 50 cm, Thermo Fisher Scientific), operated at 40°C. A gradient from 5% to 95% mobile phase B was applied over 90 min with a flow rate of 250 nL/min; mobile phase A was H_2_O/FA (99.9:0.1, v:v) and mobile phase B was acetonitrile/H_2_O/FA (80:19.9:0.1, v:v:v). The ESI voltage was 1800–1850 V, and the ion transfer tube temperature was 275°C.

UHPLC-MS/MS data were acquired based on a data-dependent top-speed mode that consisted of a full scan that maximized the number of MS2 scans over 3 s of cycle time. A full scan (MS1) was detected by the Orbitrap analyzer at a resolution of 120 K, with a mass range of 350–2,500 m/z. The automatic gain control (AGC) target value was 4e5, the maximum injection time was 100 ms, and the included charge states were 2–10. The second scan (MS2) included HCD-triggered CID in EThcD mode. HCD spectra were detected by the Orbitrap analyzer at a resolution of 30 K, with a fixed collision energy of 30%. The maximum injection time was 54 ms, isolation window size was 2, AGC target value was 5e4, and fixed first mass was 110 m/z. If HexNAc and the fragment ions of HexNAc (204.0872, 126.0550, 138.0555, 168.0661, and 186.0766 m/z) were detected at least once or twice, then the CID and EThcD MS2 on the same precursor ion were triggered. The CID and EThcD spectra were detected by the Orbitrap analyzer at a resolution of 30 K, maximum injection time of 54 ms, isolation window size of 2 and AGC target value of 5e4. CID was performed at a collision energy of 35%, and EThcD was performed with a SA collision energy of 15% and reacted over 100 ms.

### Data analysis

For the glycopeptide analysis, raw files were converted to ms1 and ms2 by RawConverter (The Scripps Research Institute, United States) (He et al., 2015). To construct GPA 2.0 databases, a proteome search was conducted by the Integrated Proteomics Platform (IP2) for MS data analysis (Bruker, Massachusetts, United States). The following IP2 parameters were used: DBs were mixed based on the sequences of Infliximab (SILu™MAb Infliximab, MSQC9, Sigma-Aldrich, MO, United States), human IgGs, and whole mouse proteins (Uniprot, download at 2019), the precursor/peptide mass tolerance was 10 ppm, the fragment mass tolerance was 20 ppm, and the maximum number of internal missed cleavages was 1. Cysteine residues were searched with a static modification for carboxyaminomethylation (+57.02146) and differential modifications for methionine oxidation. The minimum number of peptides per protein was 2, and the false positive rate was 0.01 at the spectrum level.

Site-specific N- and O-glycopeptides were identified by GlycoProteome Analyzer (GPA) 2.0 (KBSI, Korea) (Park et al., 2016, 2020), where the glycopeptide DBs were generated with the identified proteins in combination with 351 N-glycans and 11 O-glycans (Park et al., 2016, 2020; Hwang et al., 2020; Lee et al., 2020). The following parameters of GPA 2.0 were used: N- and O-glycopeptide search was ‘allowed,’ multiple glycosylation sites were “disallowed,” the MS2 noise threshold was 2.0, precursor and HCD mass tolerances were 0.02 Da, CID and ETD mass tolerances were 0.05 Da, and Y-score threshold was 60.0. After the analysis, the identified glycopeptides were re-calculated their corresponding the precursor mass error with PPM. In order to quantify identified glycopeptides, we used an in-house program coded by Python 3.8 which extracts and sums the intensities of M, M + 1, and M + 2 from the corresponding isotope distribution in their chromatogram. To evaluate similarity of glycosylation of originator and biosimilar, the Pearson correlation coefficient was calculated by an in-house program, written in python (version 2.7) using Pandas module (version 0.24.2, https://pandas.pydata.org/).

## Results

### Identification of site-specific N- and O-glycopeptides from infliximab

**FIGURE 1:**
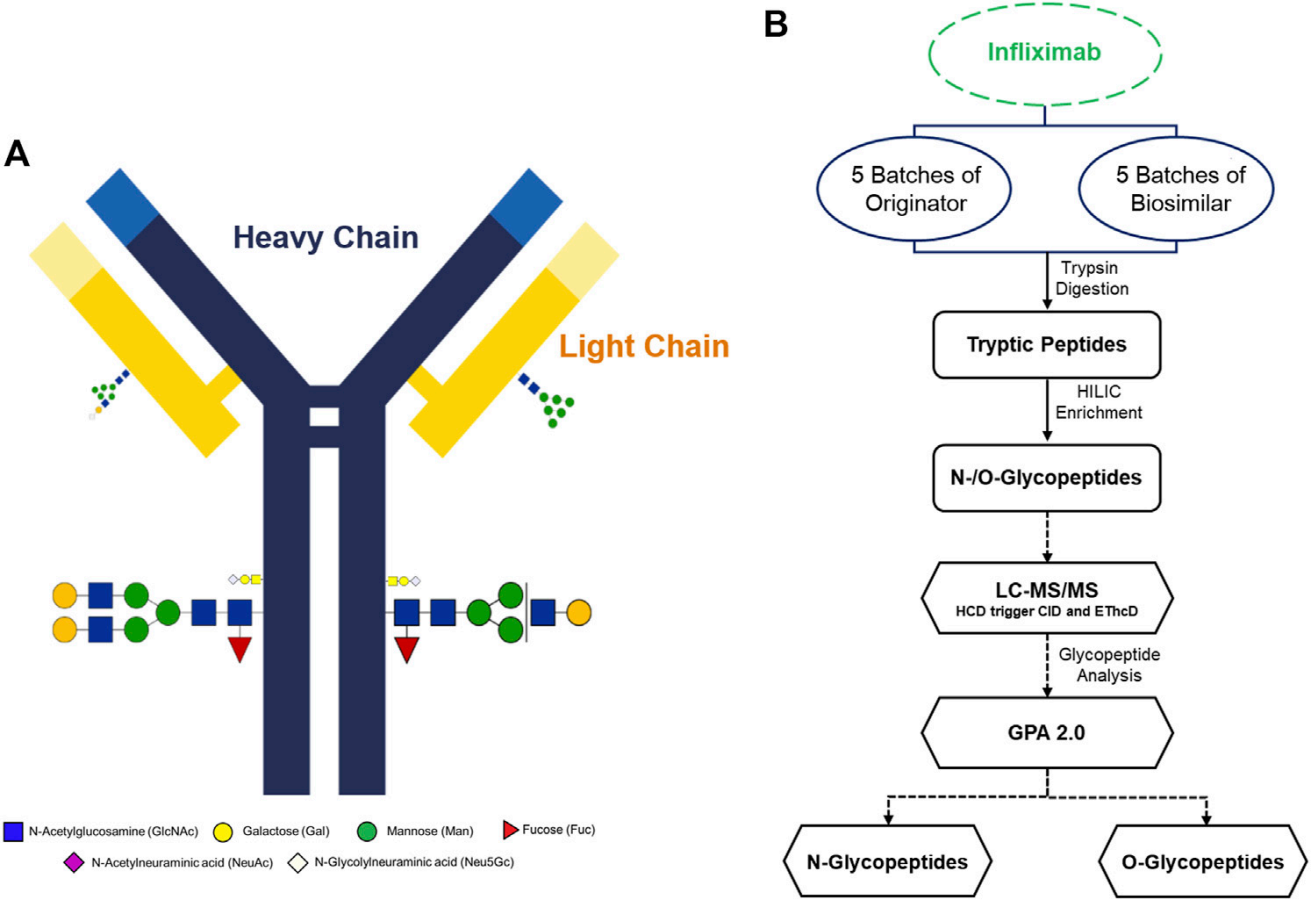
Overview of the experimental strategy and molecular structure of Infliximab. **(A)** Schematic illustration of Infliximab. The heavy chains are indicated as blue lines, and the light chains are indicated as yellow lines. **(B)** A glycoproteome workflow for the analysis of N- and O-glycopeptides of Infliximab.

Infliximab is a genetically engineered mAbs and a tetrameric glycoprotein that consists of two light and two heavy chains. A simple schematic diagram of infliximab with glycoproteins, heavy chains and light chains was constructed. According to previous studies, there is one N-glycosylation site located in the Fc region (Figure 1A) (Pisupati et al., 2017). In this study, we have shown that utilizing tandem mass spectra of glycopeptides could be used for site-specific glycosylation profiling and quantification of Infliximab. A flow chart was developed to show the workflow of the analysis of N- and O-glycopeptides of the originator and biosimilar of Infliximab. (Figure 1B). Five batches each of originator and biosimilar were prepared to evaluate the similarity of site-specific glycosylation using the Pearson correlation coefficient based on the number of glycopeptide spectra matches (GSMs). Glycopeptides were prepared through trypsin digestion and HILIC enrichment, and then a LC-MS/MS analysis was performed. N- and O-glycopeptides could be identified using the GPA 2.0 processing program. Glycopeptides were denoted by peptide sequence and their monosaccharide compositions as the number of hexose (Hex), N-acetyl hexosamine (HexNAc), fucose (Fuc), N-acetylneuraminic acid (NeuAc), N-glycolylneuraminic acid (NeuGc) as following; peptide sequence_#Hex_#HexNAc_#Fuc_#NeuAc_#NeuGc, e.g. EEQYNSTYR_4_3_1_0_1.

**FIGURE 2:**
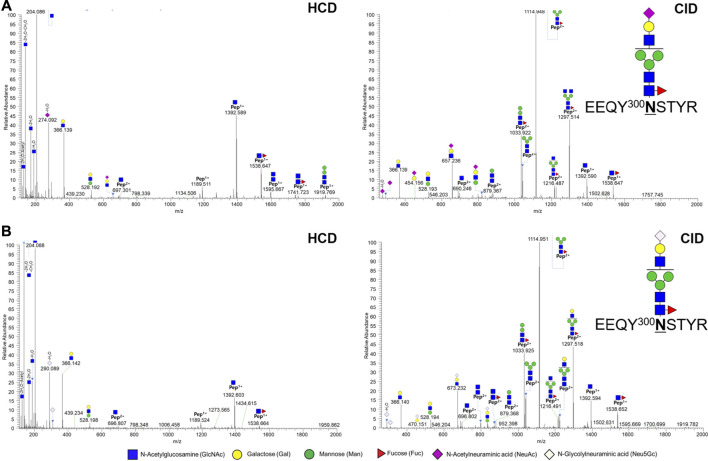
Representative tandem spectra of N-glycopeptides with NeuAc and N-glycopeptides with NeuGc from the Fc region in Infliximab. **(A)** EEQY^300^ **N**STYR_4_3_1_1_0 (m/z 962.38, 3+) and **(B)** EEQY^300^ **N**STYR_4_3_1_0_1 (m/z 967.71, 3+).

In a previous report, the N-glycopeptide was only located at the N300 position of the Fc region (Pisupati et al., 2017). Herein, we identified the glycopeptide from not only the N300 site, but also the N41 site (Table 1, Supplementary Table S1 and 2). A total of 49 and 54 glycopeptides were identified in biologics and biosimilar at two sites, respectively. At the N41 site, 5 and 7 N-glycopeptides (32 and 149 spectra) were identified in biologics and biosimilar, respectively, and core1 type O-glycosylation at THTCPPCPAPELLGGPSVFLFPPKPK (amino acids of 226–251) was identified only in biosimilar. (Supplementary Figure S2) We also identified 12 and 18 glycopeptides (618 and 678 spectra) with NeuGc (Supplementary Table S5), and 4 and 3 glycopeptides (23 and 34 spectra) containing NeuAc in biologics and biosimilar, respectively. Figure 2 shows representative tandem spectra of N-glycopeptide with NeuAc, EEQY^300^NSTYR_4_3_1_1_0, and N-glycopeptide with NeuGc, EEQY^300^NSTYR_4_3_1_0_1. Those peptide sequence is the same, but the glycan differs in only sialic acid. We confirmed that fragment ions were clearly matched as follows: EEQY^300^NSTYR_4_3_1_1_0 has peaks assigned to NeuAc (*m/z* 292.1027) and NeuAc-H_2_O (*m/z* 274.0921) in the HCD spectrum (Figure 2A). On the other hand, EEQY^300^NSTYR_4_3_1_0_1 has its fragment ions of NeuGc (*m/z* 308.0976) and NeuGc-H_2_O (*m/z* 290.0870) in the HCD spectrum (Figure 2B). These oxonium ions were used for diagnosis to determine which sialic acid were included. Because the peptide sequences and the glycan structures without sialic acid of the two glycopeptides are same, the Y ions produced by glycosidic cleavage were almost identical in the CID spectra. Few glycan fragment ions were better observed in the CID spectra than in the HCD spectra, where they were oxonium ions with large molecular weight containing sialic acid such as *m/z* 657.236 and *m/z* 673.232.

**TABLE 1:** Identified N-glycopeptides and glycosylation sites from tandem spectra of Infliximab.

Glycosylation type	Part of chain	Glycopeptide number (amino acid position)	Glycosylation siteGlycosylation site	Glycopeptide sequence	Biologics		Biosimilar	
					Glyco-peptides	GSMs	Glyco-peptides	GSMs
				EEQYNSTYR		785		968
	Heavy	AA 296–304	300	EEQYNSTYRVVSVLTVLHQDWLNGK	51	4	55	1
N				TKPREEQYNSTYR		348		284
	Light	AA 40–45	41	TNGSPR	3	13	7	99
O	Heavy	AA 226–251	n.d	THTCPPCPAPELLGGPSVFLFPPKPK	0	0	1	3
Total					54	1,150	63	1,355

**FIGURE 3:**
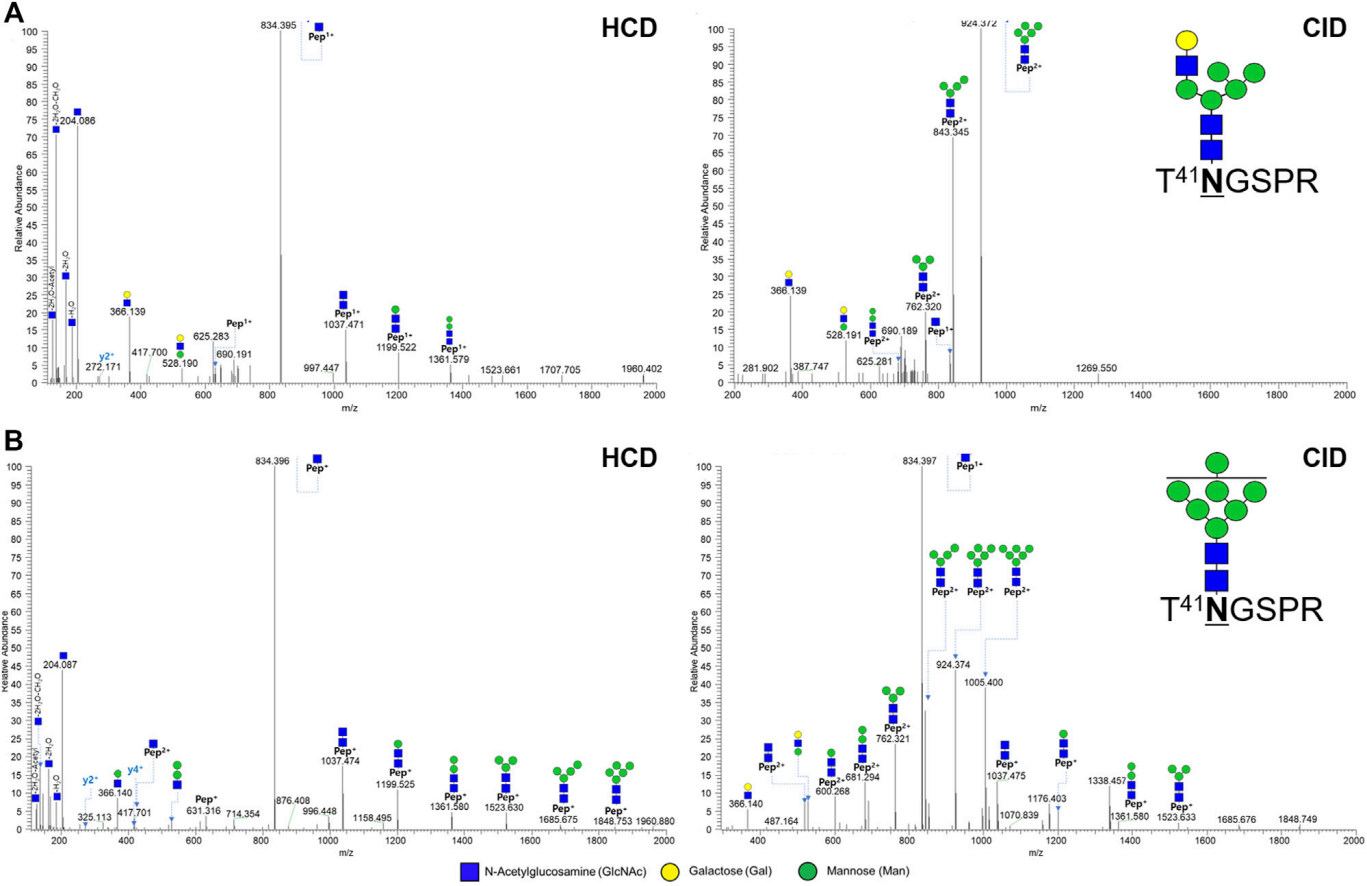
Representative tandem spectra of novel N-glycopeptide of the hybrid type and the high-mannose type from Infliximab. **(A)** T^41^NGSPR_6_3_0_0_0 (m/z 738.63, 2+) of the hybrid type N-glycopeptide and **(B)** T^41^NGSPR_7_2_0_0_0 (m/z 1,086.93, 2+) of the high-mannose type N-glycopeptide.

At N41 site, only hybrid type and high mannose type glycosylation were expressed, unlike the N300 site (Supplementary Figure S1, S2). Two out of 10 glycopeptides at N41 located in the light chain were shown in Figure 3. In the HCD spectrum, seven oxonium ions, five Y ions, and 1 y ion presents T^41^ **N**GSPR_6_3_0_0_0, a hybrid type N-glycopeptide (Figure 3A). Also, in the HCD spectrum, seven oxonium ions, nine Y ions, and 2 y ions clearly demonstrate a glycopeptide fragmentation pattern as high mannose type N-glycopeptide T^41^ **N**GSPR_7_2_0_0_0 (Figure 3B). Y ions were sequentially matched in both CID spectra.

### Quantitative analysis and evaluation of similarity between the originator and biosimilar

**FIGURE 4:**
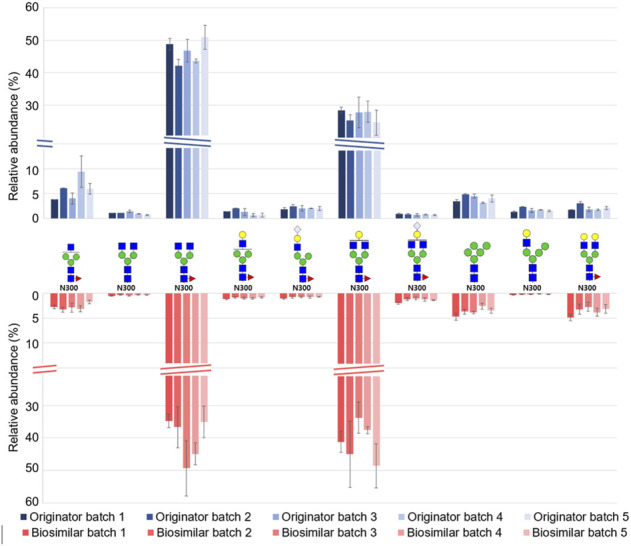
Relative abundance distribution of the top 10 abundant N-glycopeptides from five batches of Remicade® and Remsima®.

For a label-free quantitative analysis of a total of 60 glycopeptides from Infliximab, we used an in-house program (coded by Python 3.8) that extracts and sums the intensities of M, M + 1, and M + 2 from the corresponding isotope distribution in their chromatogram. (Supplementary Table S6, S7) Based on the label free quantification result, Figure 4 shows the relative abundance of the top 10 abundant glycopeptides from five batches of biologics and biosimilar. All of the top 10 glycopeptides were expressed at the N300 site. The overall qualitative profiles for both the originator and biosimilar seems similar. The most frequently expressed glycan were G0F (3_4_1_0_0) and G1F (4_4_1_0_0) in N300, and it is in agreement with the results of previous studies. (Fang et al., 2016; Pisupati et al., 2017). 8 out of 10 glycopeptides were fucosylated, and 4 out of 10 sialylated glycopeptides were attached with NeuGc.

**FIGURE 5:**
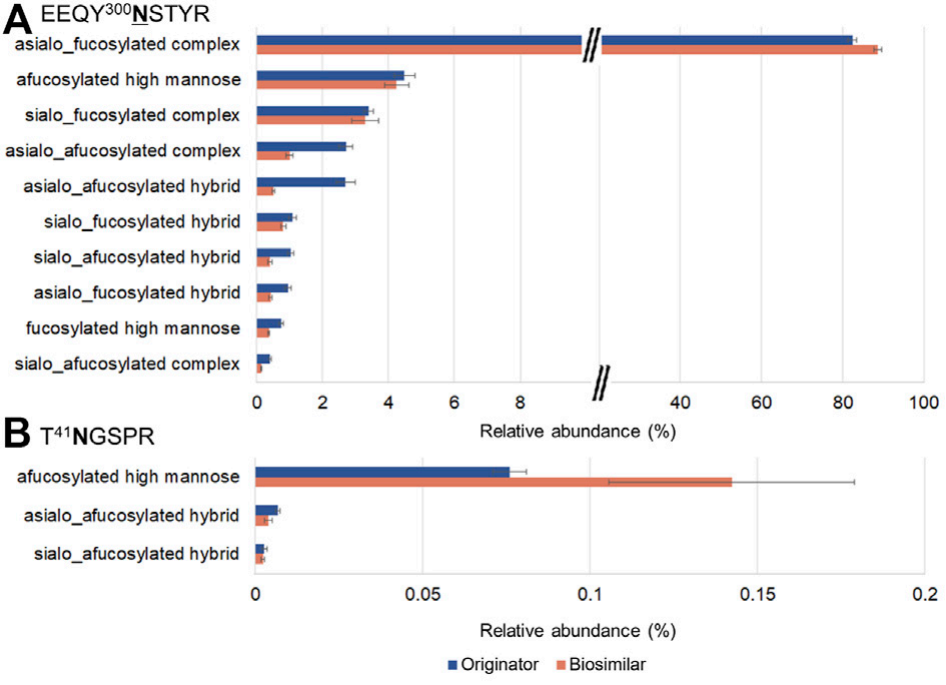
Glycoform profiles of site-specific N-glycopeptides from the originator (Remicade®) and biosimilar (Remsima®). Bar graphs showed the comparison of the glycoform profiles in Remicade® and Remsima® based on the number of GSMs of EEQY^300^NSTYR **(A)** and T^41^NGSPR **(B)**. The error bar indicates standard error.

EEQY^300^NSTYR and T^41^NGSPR contain a total of 10 and 3 glycoforms, respectively. (Figures 5A,B). Trace levels of glycopeptides abundance for EEQY^300^NSTYR were similar in both mAbs. At the N300 site, the most abundant were asialo-fucosylated complex glycopeptides (82.4%, originator and 88.6%, biosimilar), afucosylated high mannose glycopeptides (4.5%, originator and 4.2% biosimilar), and sialo-fucosylated complex glycopeptides (3.4%, originator and 3.3%, biosimilar). On the other hand, at the N41 site, the afucosylated high mannose glycopeptides from the biosimilar were remarkably higher.

**FIGURE 6:**
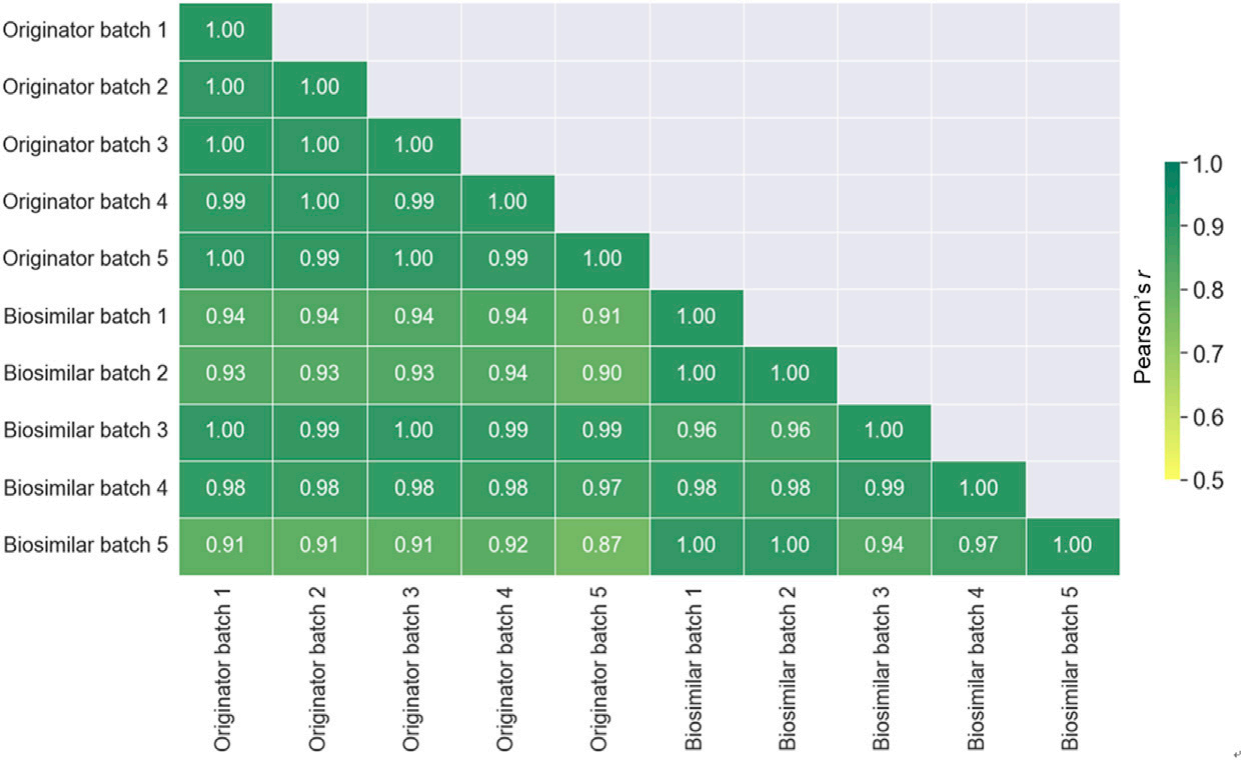
A Heat map displaying the spectra similarity between the originator (Remicade®) and biosimilar (Remsima®) batches. The numbers displayed in the heat map represent the Pearson correlation coefficient values.

To evaluate the similarity between all batches of the originator and biosimilar, the Pearson correlation coefficients was calculated by the relative abundance (Figure 6). A value closer to +1.0 indicates a better correlation. The similarities between batches in the same mAbs (0.93–0.97) were higher than those between originator and biosimilar (0.84–0.94). Therefore, these results presented that the similarity between different batches is better than that between the originator and biosimilar of infliximab.

## Discussion

Infliximab, therapeutic monoclonal antibodies targeting tumor necrosis factor-alpha (TNFα), is used to treat inflammatory diseases. Infliximab has been found to have one N-glycosylation in the Fc region (Pisupati et al., 2017), and glycosylation is a very important target to be analyzed in therapeutic mAbs because it affects the PK, PD, effector functions, solubility, and stability. (Arnold et al., 2007; Liu, 2015; Higel et al., 2016). In this study, the development of workflow analysis for site-specific glycopeptides from therapeutic mAbs was reported. In terms of usefulness of the LC-MS/MS method, a comparative analysis was conducted through the identification and the relative quantification of the site-specific glycopeptide for infliximab and its biosimilar.

For analysis of Infliximab’s glycosylation, previous studies examined by native mass spectrometry, ion mobility, and quantitative glycan. Jing Fang et al. (2016) showed that a released N-glycan analysis was performed using UPLC-MS to evaluate the similarities and possible differences between the originator and biosimilar. (Fang et al., 2016). A total of 23 and 21 glycans were identified on Infliximabs and its biosimilar. Similarly, Pisupati et al. identified approximately 25 glycoforms for Remicade® and Remsima® and observed glycoform population differences. (Pisupati et al., 2017). Those studies reported only N-glycosylation on Fc region. In our study, we identified 49 and 54 N- and O-glycopeptides for biologics and biosimilar, where we found approximately double number of N-glycopeptide than the Pisupati study. In addition, we first discovered one novel N-glycosylation site at N41 site from light chain of both Infliximabs and one O-glycopeptide AA 226–251 of Fc region in only biosimilar. At N41 site, five and four hybrid type and high mannose type N-glycopeptides were newly identified, respectively. The site-specific information on glycosylation at N41 site could not be obtained with conventional approach using glycan analysis released from the protein or top-down analysis of intact protein. In addition, evaluation of NeuGc from therapeutic mAbs is important. Numerous animals, including cattle, horses, mice, and rats, produce NeuGc (Muchmore et al., 1998). Since the human cannot synthesize NeuGc due to deletion of the cytidine monophospho 5′-N-acetylneuraminic acid (CMP-NeuAc) hydroxylase gene (Chou et al., 1998). In this study, more N-glycopeptides with NeuGc were identified than N-glycopeptides with NeuAc in both Infliximab, and four of the top 10 glycopeptides were N-glycopeptides with NeuGc.

From the quantitative comparison between the biologics and biosimilar, the distribution of glycopeptides appeared to be similar pattern, but the structure of major glycan were different depending on the glycosylation sites. Therefore, evaluation of glycosylation through glycopeptide analysis would be appropriate for therapeutic mAbs with multiple glycosylated sites. In addition, the similarity of glycosylation of mAbs was calculated on the basis of quantitative data of glycopeptides using the Pearson correlation coefficient. It showed a higher correlation between batches of the same mAbs than between different mAbs. We showed that the site-specific analysis of glycopeptides using tandem mass spectrometry is useful for providing more sophisticated information in biopharmaceutical evaluation.

## Data Availability

The datasets presented in this study can be found in online repositories. The names of the repository/repositories and accession number(s) can be found below: http://proteomecentral.proteomexchange.org/cgi/GetDataset?ID=pxd035788.
