# Supplementary material for: Advanced assessment through intact glycopeptide analysis of Infliximab’s biologics and biosimilar
Source: Front Mol Biosci. 2022 Nov 29;9:1006866. doi: 10.3389/fmolb.2022.1006866 (PMC9745114; doi:10.3389/fmolb.2022.1006866)

Glycan_Types	Glycopeptides	HCD_scan	CID_scan	Raw File	Page
N	EEQYNSTYR_3_2_0_0_0	4227	4230	Remsima_Batch2_03	2
N	EEQYNSTYR_3_2_1_0_0	4415	4417	Remsima_Batch3_03	3
N	EEQYNSTYR_3_3_0_0_0	4419	4422	Remsima_Batch1_01	4
N	EEQYNSTYR_3_3_1_0_0	4446	4448	Remsima_Batch1_02	5
N	EEQYNSTYR_3_4_0_0_0	4495	4497	Remsima_Batch1_03	6
N	EEQYNSTYR_3_4_1_0_0	4088	4090	Remsima_Batch4_02	7
N	EEQYNSTYR_4_2_0_0_0	4280	4282	Remsima_Batch1_03	8
N	EEQYNSTYR_4_2_1_0_0	7006	7008	Remsima_Batch4_03	9
N	EEQYNSTYR_4_3_0_0_0	4446	4449	Remsima_Batch4_02	10
N	EEQYNSTYR_4_3_0_0_1	6934	6936	Remsima_Batch1_02	11
N	EEQYNSTYR_4_3_1_0_0	4188	4191	Remsima_Batch3_01	12
N	EEQYNSTYR_4_3_1_0_1	6818	6820	Remsima_Batch4_01	13
N	EEQYNSTYR_4_3_1_1_0	6933	6936	Remsima_Batch2_03	14
N	EEQYNSTYR_4_4_0_0_0	4396	4398	Remsima_Batch2_03	15
N	EEQYNSTYR_4_4_0_0_1	6959	6961	Remsima_Batch1_01	16
N	EEQYNSTYR_4_4_1_0_0	4455	4457	Remsima_Batch1_02	17
N	EEQYNSTYR_4_4_1_0_1	7076	7078	Remsima_Batch5_03	18
N	EEQYNSTYR_4_4_1_1_0	6937	6939	Remsima_Batch3_03	19
N	EEQYNSTYR_4_5_0_0_0	4433	4435	Remsima_Batch3_01	20
N	EEQYNSTYR_4_5_1_0_0	4810	4812	Remsima_Batch5_03	21
N	EEQYNSTYR_5_2_0_0_0	4062	4064	Remsima_Batch2_01	22
N	EEQYNSTYR_5_2_1_0_0	4465	4468	Remsima_Batch5_03	23
N	EEQYNSTYR_5_3_0_0_0	4211	4213	Remsima_Batch4_01	24
N	EEQYNSTYR_5_3_0_0_1	6811	6813	Remsima_Batch3_03	25
N	EEQYNSTYR_5_3_1_0_0	4352	4354	Remsima_Batch1_01	26
N	EEQYNSTYR_5_3_1_0_1	6944	6947	Remsima_Batch4_03	27
N	EEQYNSTYR_5_4_1_0_0	4373	4375	Remsima_Batch3_02	28
N	EEQYNSTYR_5_4_1_0_1	6180	6182	Remsima_Batch3_02	29
N	EEQYNSTYR_5_4_1_0_2	8784	8786	Remsima_Batch4_01	30
N	EEQYNSTYR_5_4_1_1_0	7157	7159	Remsima_Batch4_03	31
N	EEQYNSTYR_5_5_1_0_0	4432	4435	Remsima_Batch2_01	32
N	EEQYNSTYR_5_5_1_0_1	6954	6956	Remsima_Batch4_03	33
N	EEQYNSTYR_6_2_0_0_0	4108	4111	Remsima_Batch1_02	34
N	EEQYNSTYR_6_3_0_0_0	4180	4183	Remsima_Batch1_03	35
N	EEQYNSTYR_6_3_0_0_1	6655	6658	Remsima_Batch2_01	36
N	EEQYNSTYR_6_3_1_0_0	4166	4168	Remsima_Batch1_03	37
N	EEQYNSTYR_6_3_1_0_1	6858	6860	Remsima_Batch4_03	38
N	EEQYNSTYR_6_4_1_0_0	4064	4066	Remsima_Batch1_02	39
N	EEQYNSTYR_6_4_1_0_1	6724	6726	Remsima_Batch4_02	40
N	EEQYNSTYR_6_5_1_0_1	6679	6682	Remsima_Batch4_01	41
N	EEQYNSTYR_6_5_1_0_2	9031	9033	Remsima_Batch5_03	42
N	EEQYNSTYR_7_2_0_0_0	4116	4118	Remsima_Batch4_03	43
N	EEQYNSTYR_7_4_1_0_0	4220	4223	Remsima_Batch5_01	44
N	EEQYNSTYR_7_5_1_0_1	6413	6416	Remsima_Batch2_01	45
N	EEQYNSTYR_8_2_0_0_0	3814	3816	Remsima_Batch2_01	46
N	EEQYNSTYR_9_2_0_0_0	3874	3877	Remsima_Batch5_01	47
Core1	THTCPPCPAPELLGGPSVFLFPPKPK_1_1_0_0_1	18457	18459	Remsima_Batch4_03	48
N	TNGSPR_4_2_0_0_0	1893	1896	Remsima_Batch1_01	49
N	TNGSPR_5_2_0_0_0	2092	2093	Remsima_Batch5_02	50
N	TNGSPR_5_3_0_0_1	1999	2000	Remsima_Batch1_02	51
N	TNGSPR_6_2_0_0_0	1711	1712	Remsima_Batch4_03	52
N	TNGSPR_6_3_0_0_1	2093	2095	Remsima_Batch4_03	53
N	TNGSPR_7_2_0_0_0	1400	1401	Remsima_Batch5_02	54
N	TNGSPR_8_2_0_0_0	1276	1278	Remsima_Batch5_02	55

EEQYNSTYR(=PEP)_3_2_0_0_0, m/z:694.6162(3+), RT:22.53, HCD-score:78.51, Y-score:89.52, P-score:88.89,
HCD-MS/MS Scan:4227, SNR=0.8, Base Peak Intensity=7047417

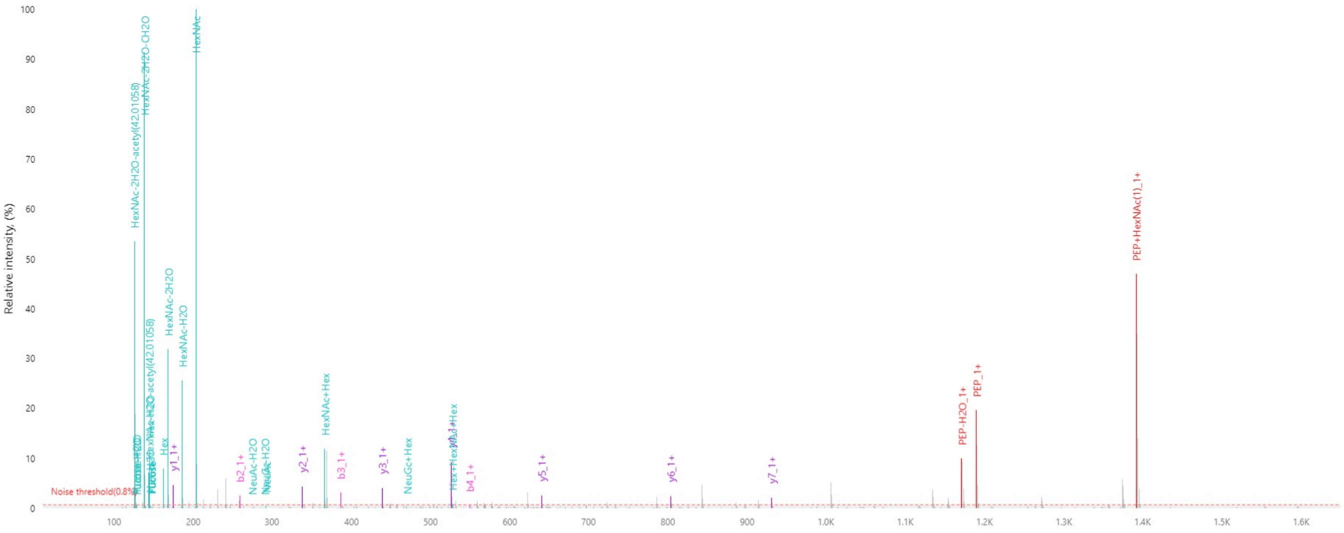

EEQYNSTYR(=PEP)_3_2_0_0_0, m/z:694.6162(3+), RT:22.55, HCD-score:78.51, Y-score:89.52, P-score:88.89,
CID-MS/MS Scan:4230, SNR=0.8, Base Peak Intensity=5055399

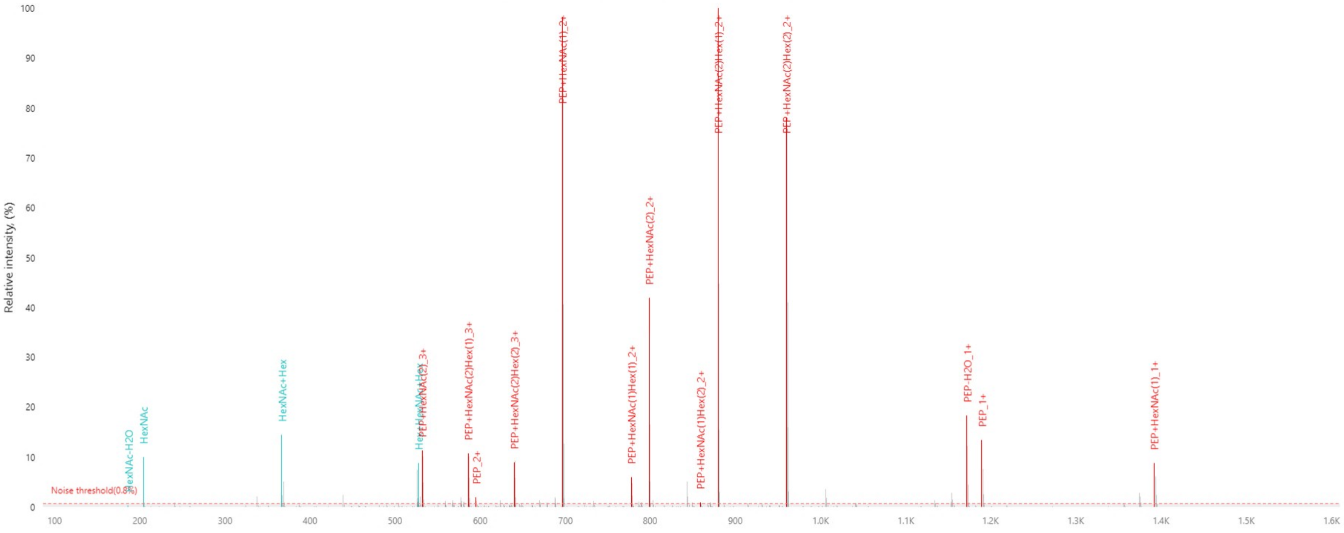

EEQYNSTYR(=PEP)_3_2_1_0_0, m/z:1114.4470(2+), RT:23.35, HCD-score:87.89, Y-score:86.89, P-score:22.22, HCD-MS/MS Scan:4415, SNR=0.8, Base Peak Intensity=1214472.9

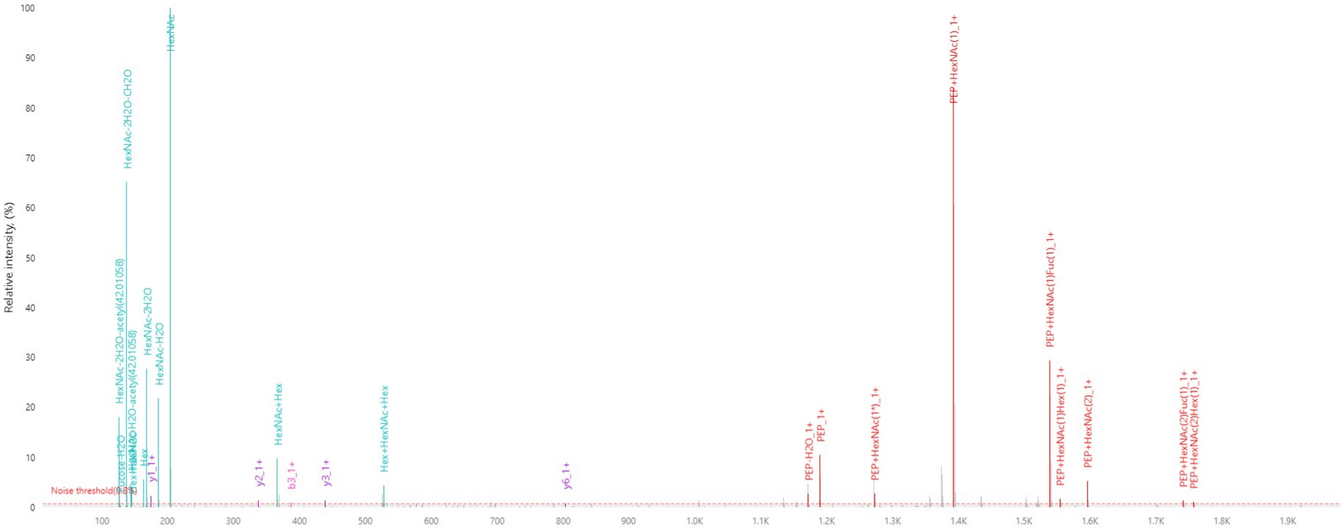

EEQYNSTYR(=PEP)_3_2_1_0_0, m/z:1114.4470(2+), RT:23.36, HCD-score:87.89, Y-score:86.89, P-score:22.22, CID-MS/MS Scan:4417, SNR=0.8, Base Peak Intensity=1403713.5

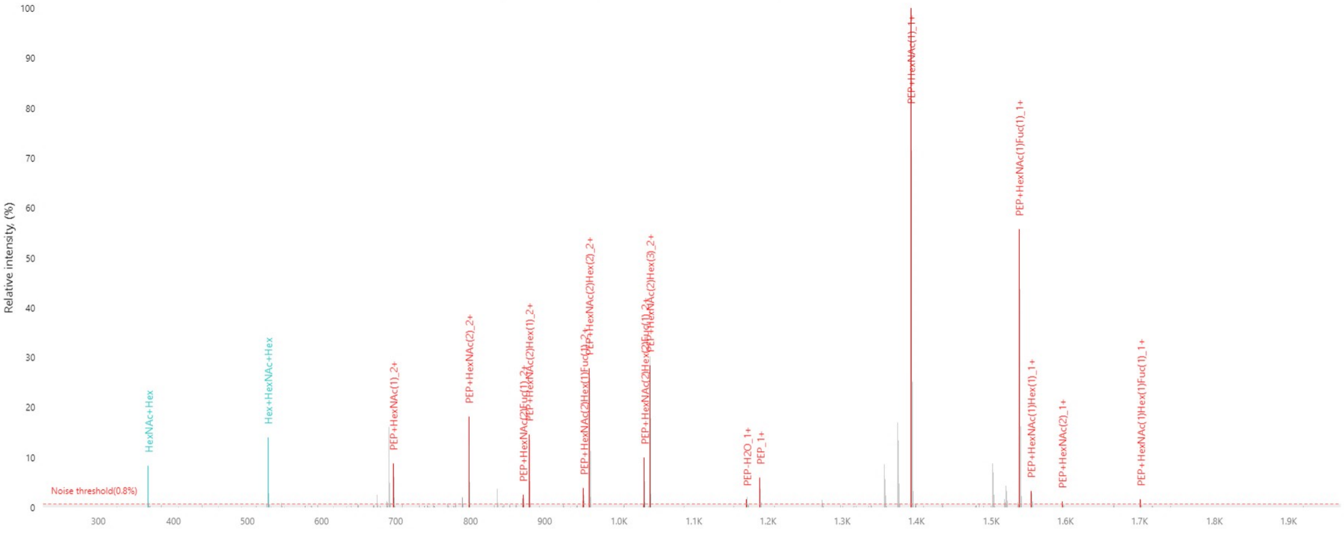

EEQYNSTYR(=PEP)_3_3_0_0_0, m/z:762.3081(3+), RT:23.15, HCD-score:89.56, Y-score:96.87, P-score:66.67,
HCD-MS/MS Scan:4419, SNR=0.8, Base Peak Intensity=4857929

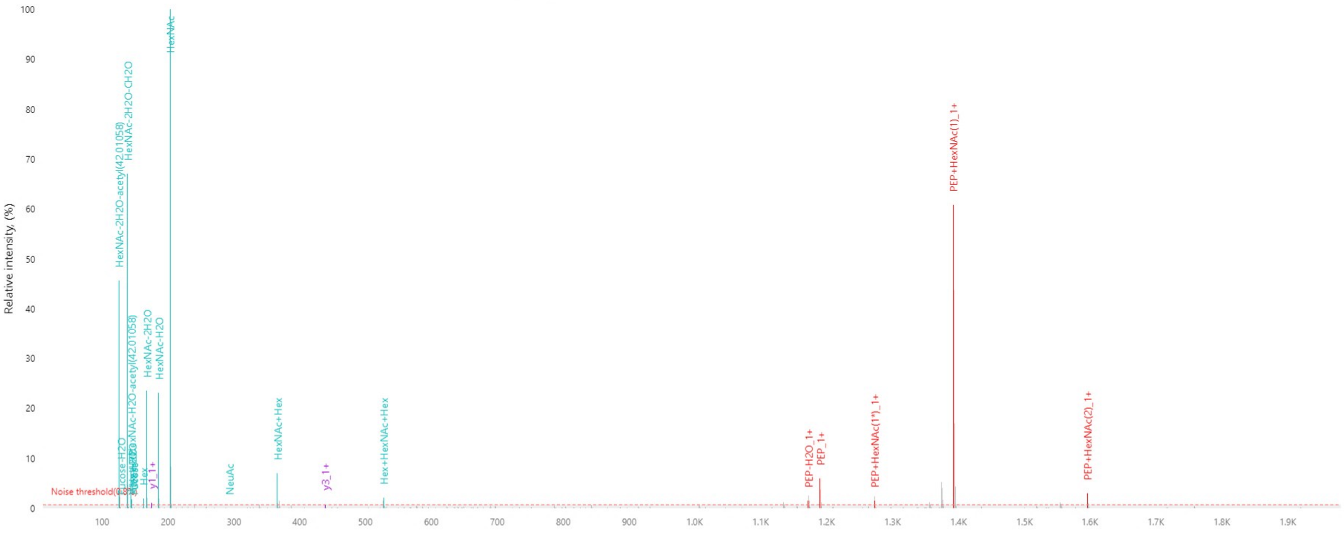

EEQYNSTYR(=PEP)_3_3_0_0_0, m/z:762.3081(3+), RT:23.16, HCD-score:89.56, Y-score:96.87, P-score:66.67,
CID-MS/MS Scan:4422, SNR=0.8, Base Peak Intensity=9691342

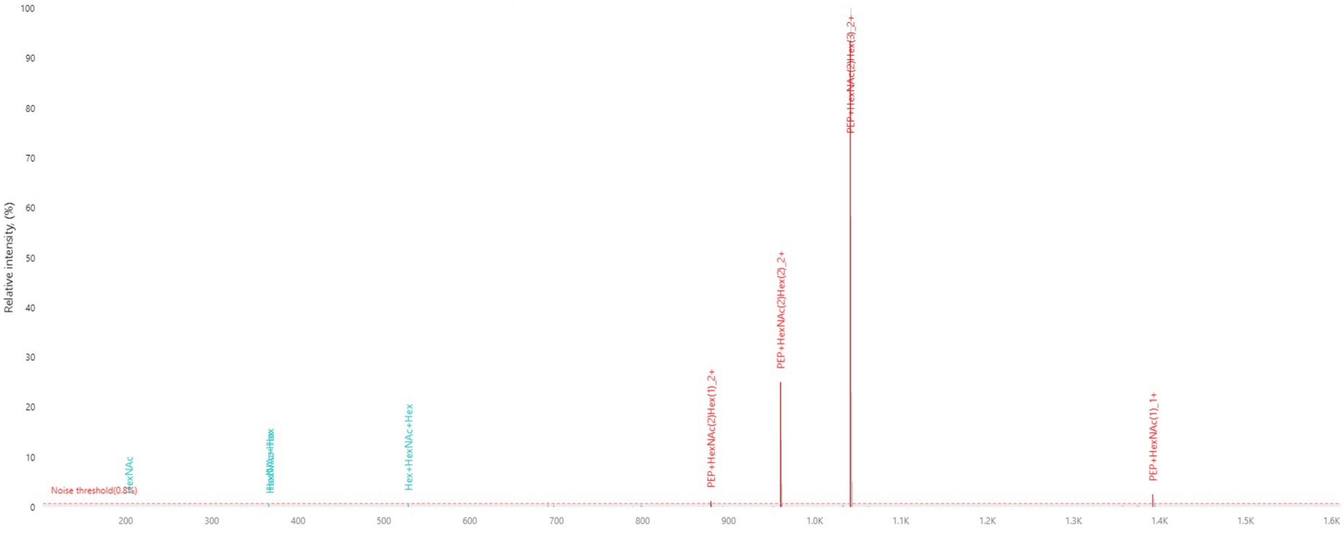

EEQYNSTYR(=PEP)_3_3_1_0_0, m/z:810.9948(3+), RT:23.27, HCD-score:91.50, Y-score:97.45, P-score:22.22,
HCD-MS/MS Scan:4446, SNR=0.8, Base Peak Intensity=6487715.5

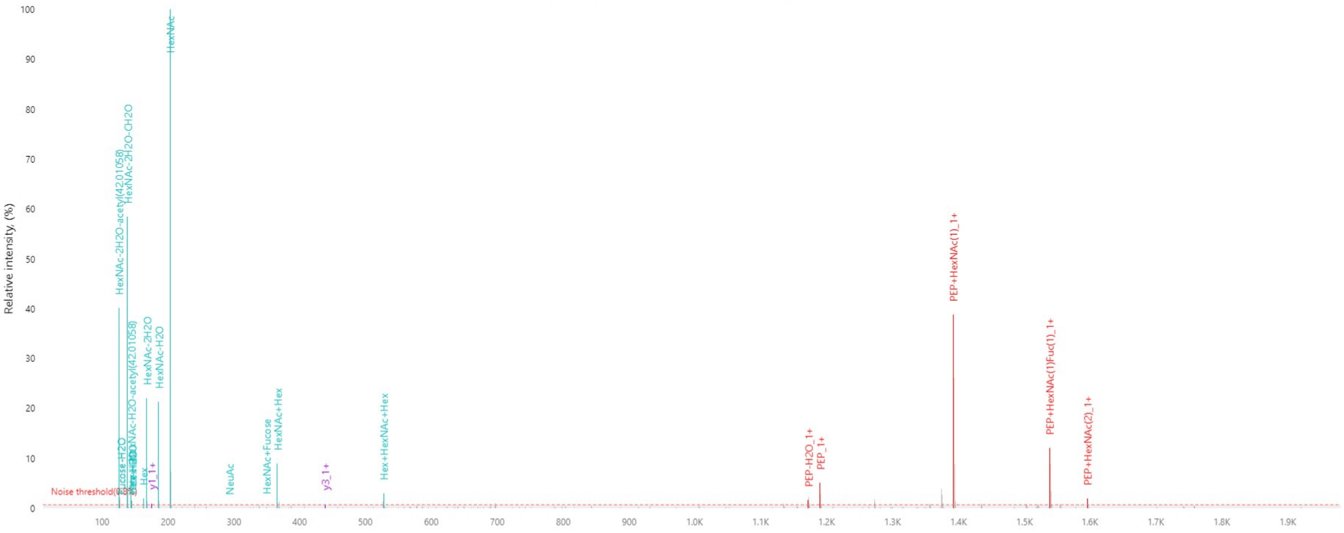

EEQYNSTYR(=PEP)_3_3_1_0_0, m/z:810.9948(3+), RT:23.27, HCD-score:91.50, Y-score:97.45, P-score:22.22,
CID-MS/MS Scan:4448, SNR=0.8, Base Peak Intensity=11181842

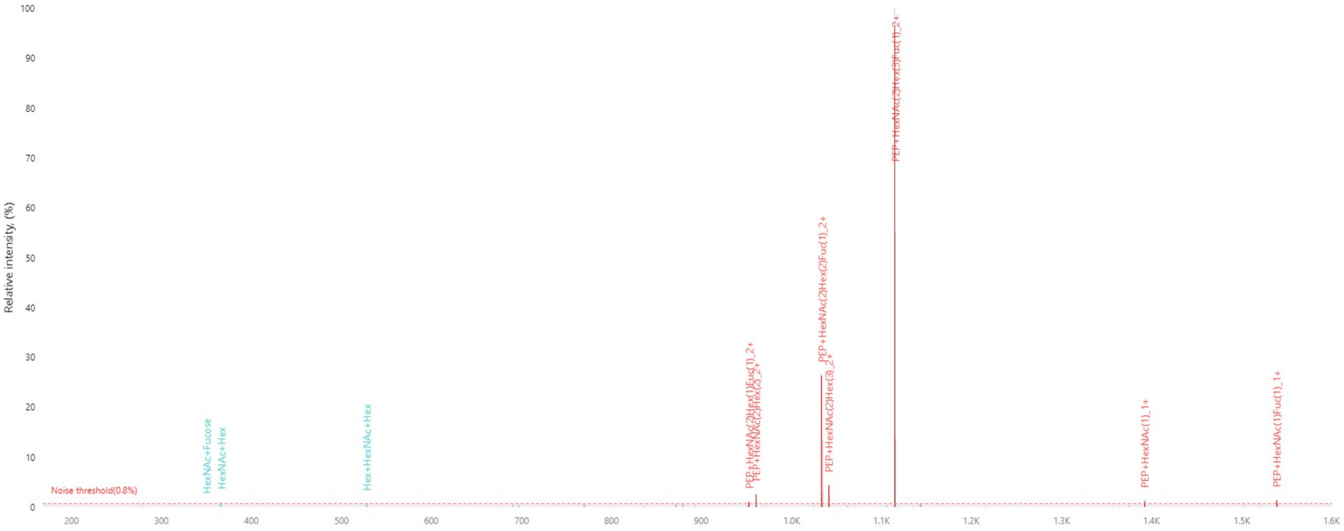

EEQYNSTYR(=PEP)_3_4_0_0_0, m/z:830.0023(3+), RT:23.48, HCD-score:95.14, Y-score:98.54, P-score:33.33,
HCD-MS/MS Scan:4495, SNR=0.8, Base Peak Intensity=10467155

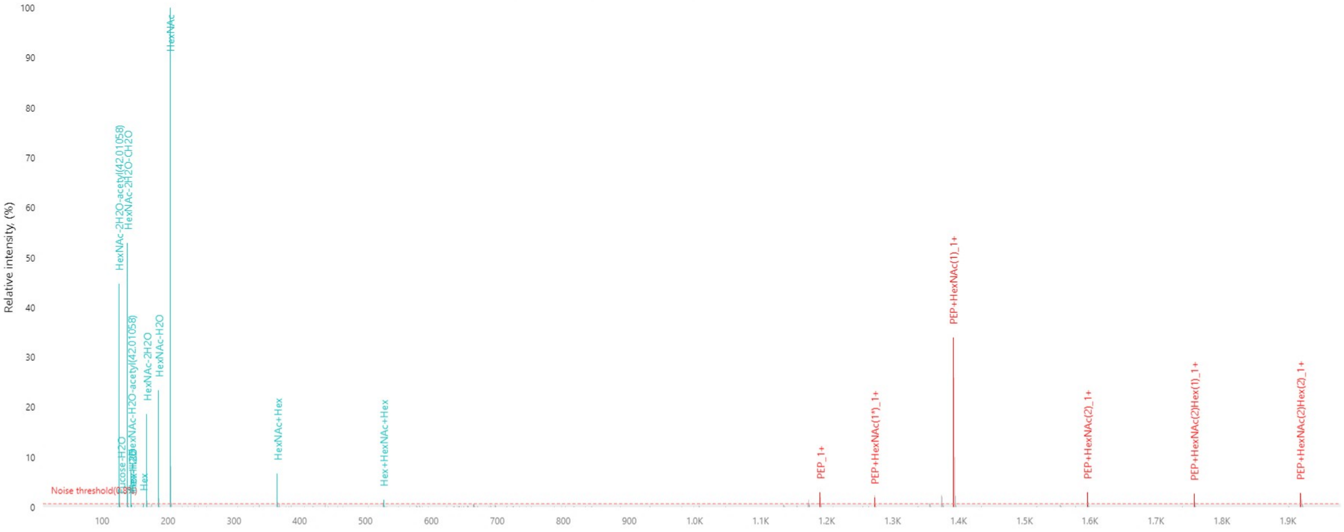

EEQYNSTYR(=PEP)_3_4_0_0_0, m/z:830.0023(3+), RT:23.48, HCD-score:95.14, Y-score:98.54, P-score:33.33,
CID-MS/MS Scan:4497, SNR=0.8, Base Peak Intensity=12732569

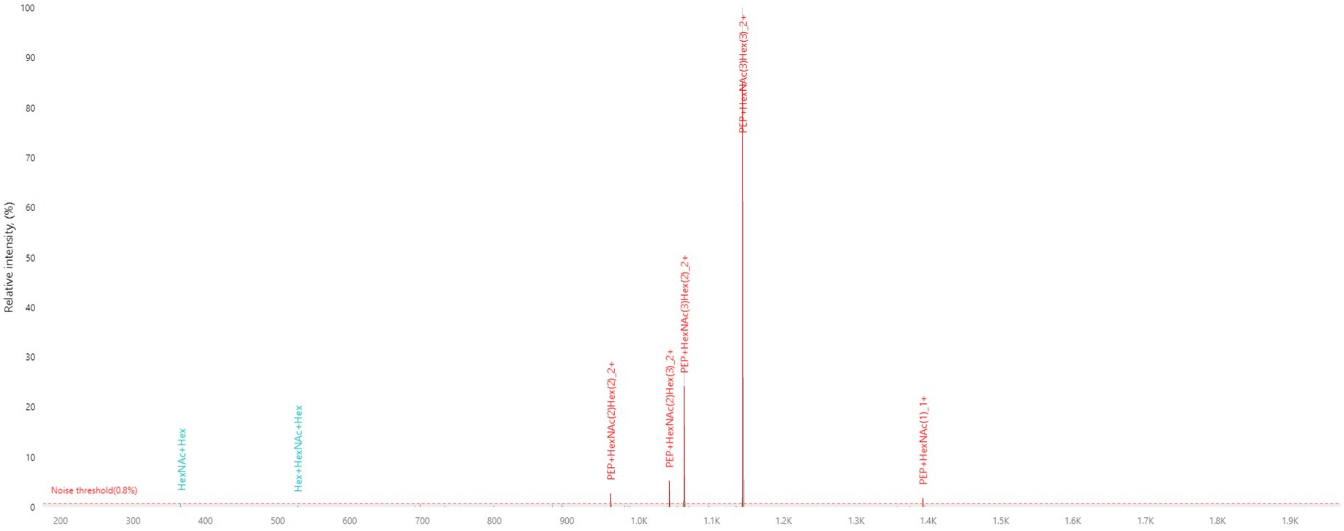

EEQYNSTYR(=PEP)_3_4_1_0_0, m/z:878.6887(3+), RT:21.73, HCD-score:90.79, Y-score:97.24, P-score:11.11,
HCD-MS/MS Scan:4088, SNR=0.8, Base Peak Intensity=8307928

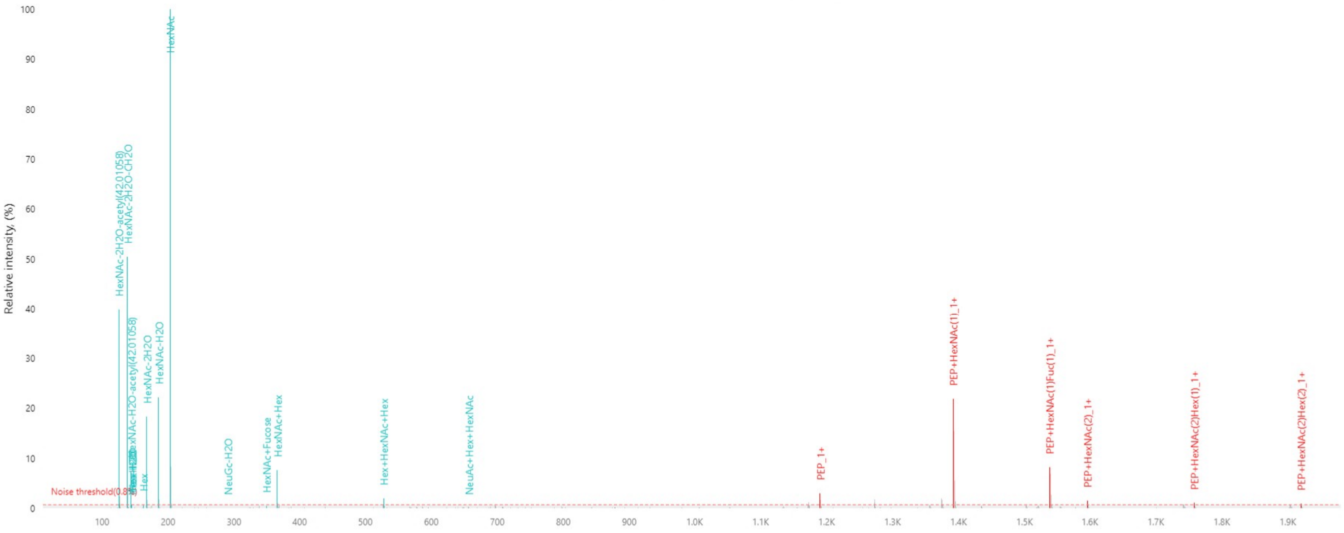

EEQYNSTYR(=PEP)_3_4_1_0_0, m/z:878.6887(3+), RT:21.73, HCD-score:90.79, Y-score:97.24, P-score:11.11,
CID-MS/MS Scan:4090, SNR=0.8, Base Peak Intensity=11036349

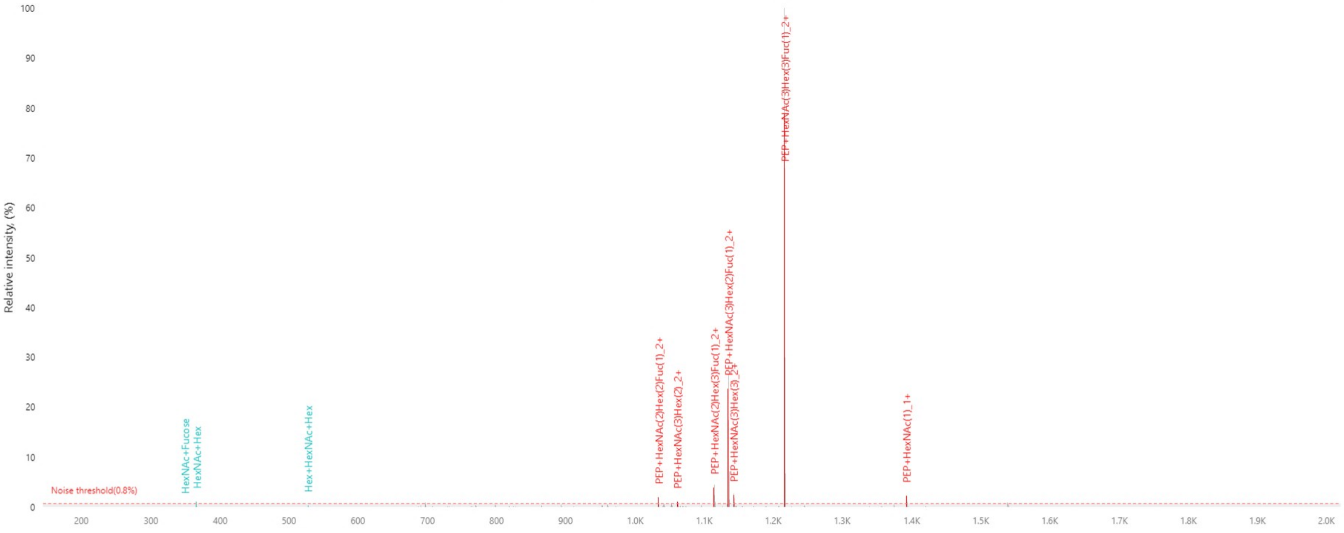

EEQYNSTYR(=PEP)_4_2_0_0_0, m/z:748.6360(3+), RT:22.72, HCD-score:82.13, Y-score:89.25, P-score:88.89,
HCD-MS/MS Scan:4280, SNR=0.8, Base Peak Intensity=18620888

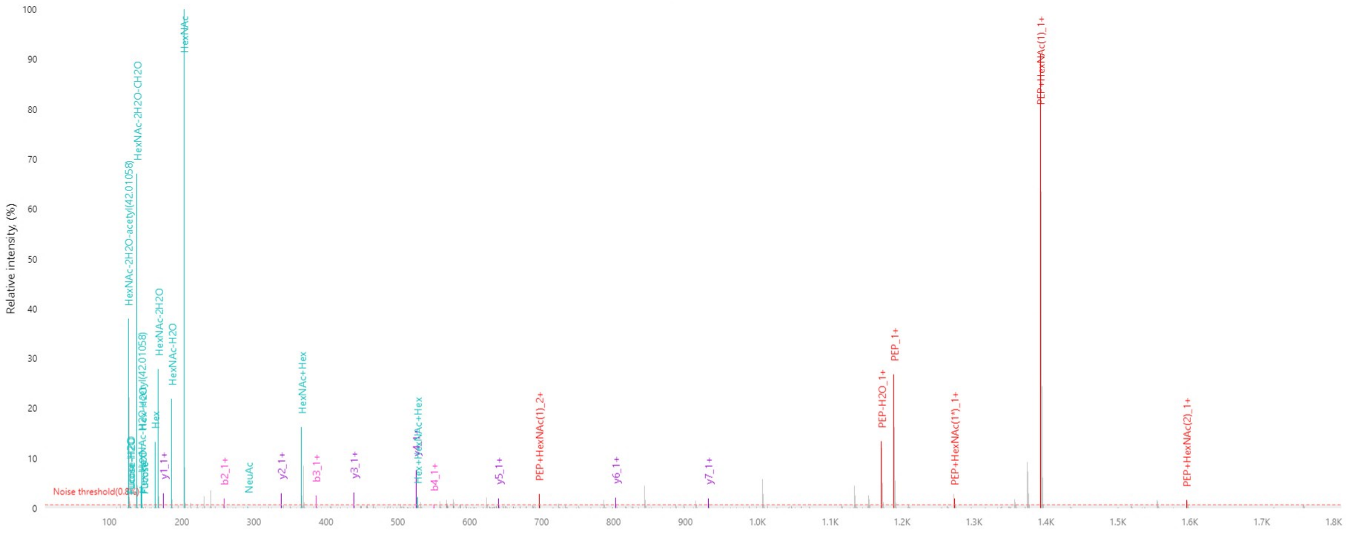

EEQYNSTYR(=PEP)_4_2_0_0_0, m/z:748.6360(3+), RT:22.72, HCD-score:82.13, Y-score:89.25, P-score:88.89,
CID-MS/MS Scan:4282, SNR=0.8, Base Peak Intensity=10240485

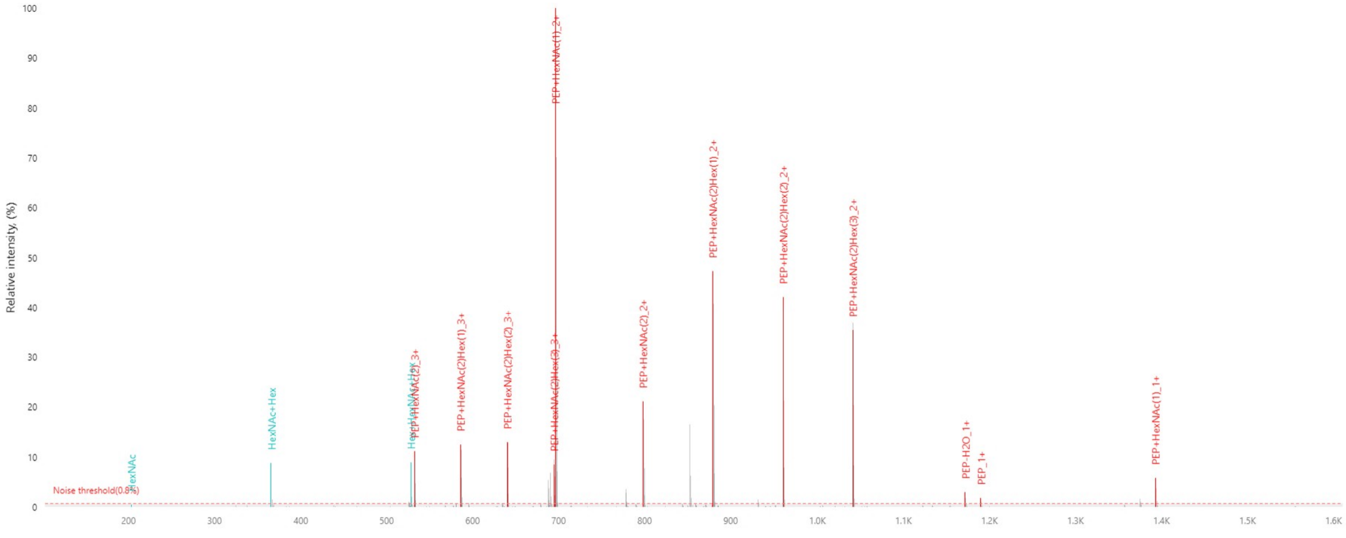

EEQYNSTYR(=PEP)_4_2_1_0_0, m/z:1195.4750(2+), RT:31.41, HCD-score:87.86, Y-score:82.84, P-score:0.00,
HCD-MS/MS Scan:7006, SNR=0.8, Base Peak Intensity=380650.9

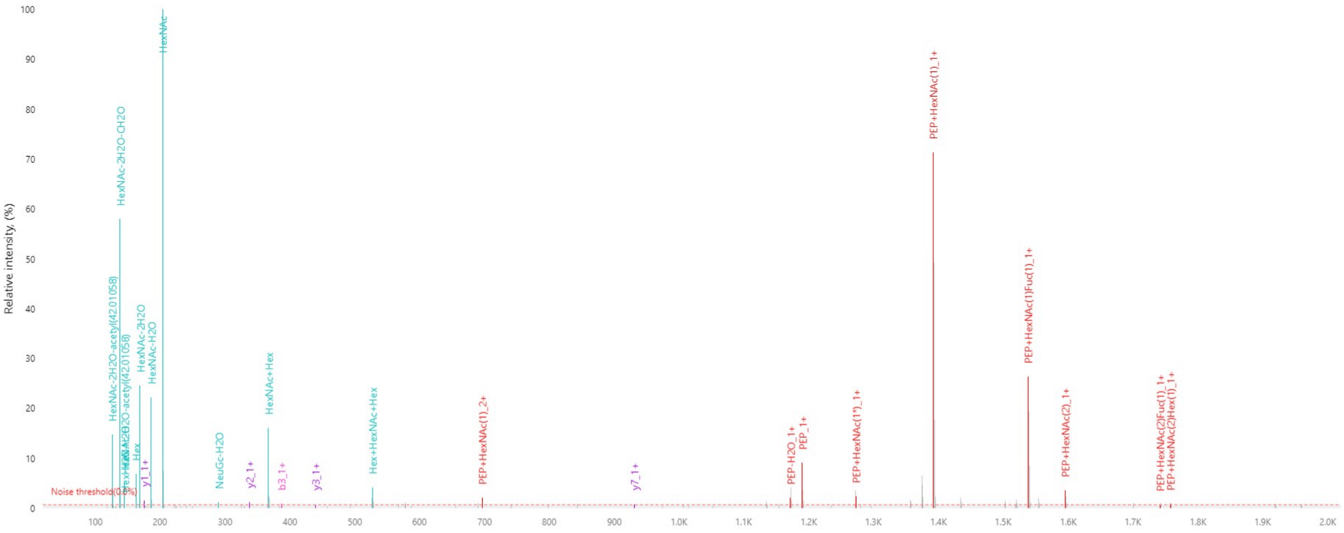

EEQYNSTYR(=PEP)_4_2_1_0_0, m/z:1195.4750(2+), RT:31.41, HCD-score:87.86, Y-score:82.84, P-score:0.00,
CID-MS/MS Scan:7008, SNR=0.8, Base Peak Intensity=280115.5

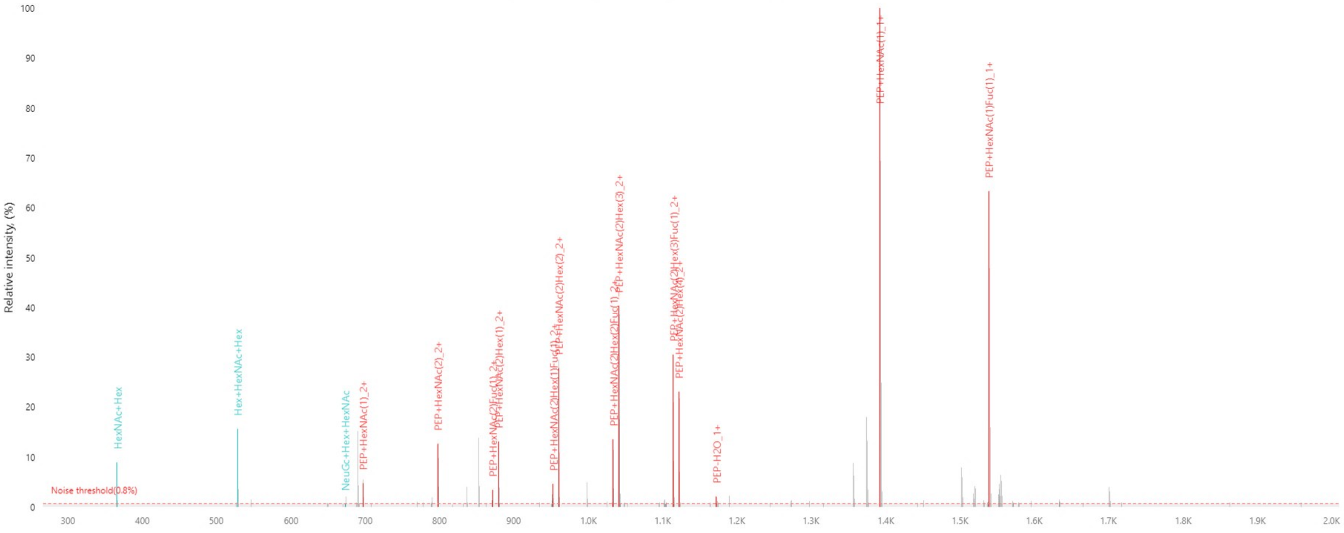

EEQYNSTYR(=PEP)_4_3_0_0_0, m/z:1223.9835(2+), RT:23.02, HCD-score:92.01, Y-score:83.62, P-score:0.00, HCD-MS/MS Scan:4446, SNR=0.8, Base Peak Intensity=1948945.8

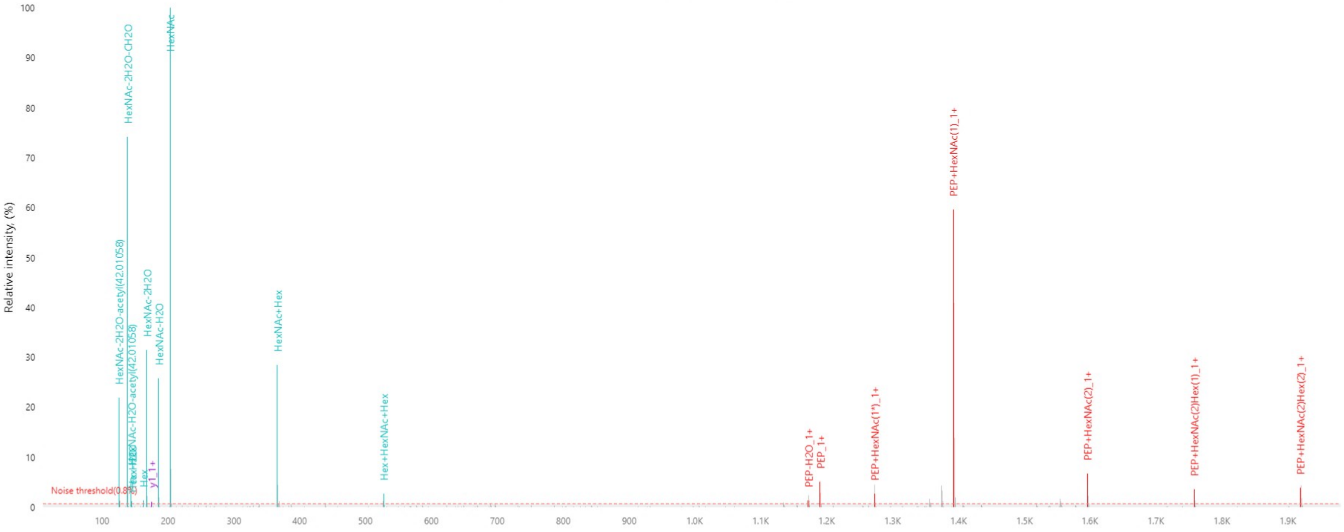

EEQYNSTYR(=PEP)_4_3_0_0_0, m/z:1223.9835(2+), RT:23.03, HCD-score:92.01, Y-score:83.62, P-score:0.00, CID-MS/MS Scan:4449, SNR=0.8, Base Peak Intensity=2179572

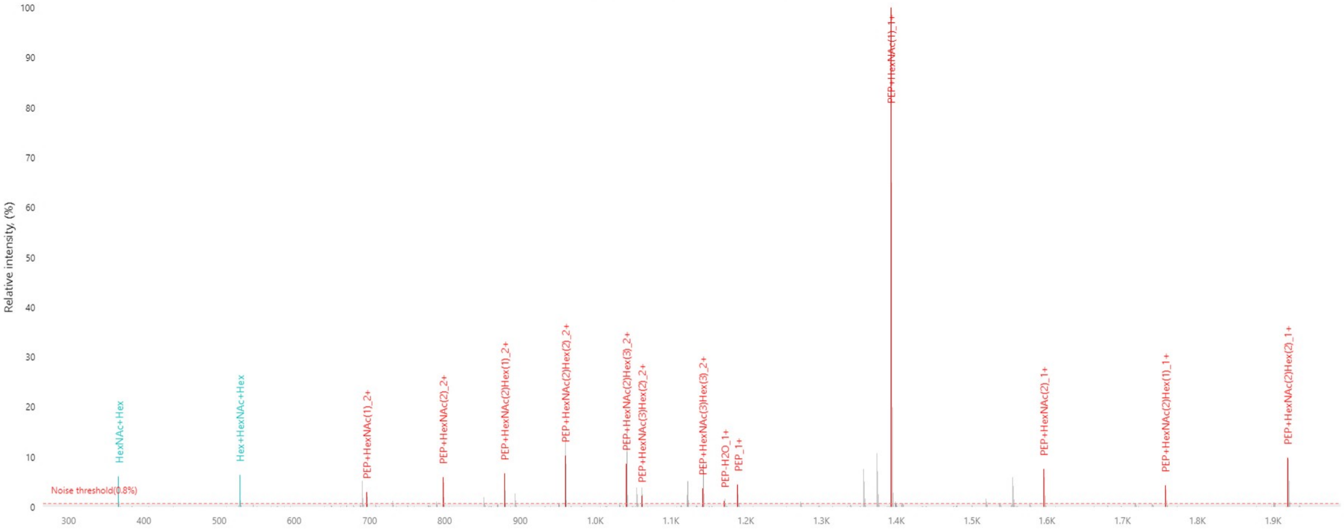

EEQYNSTYR(=PEP)_4_3_0_0_1, m/z:918.6904(3+), RT:31.68, HCD-score:91.50, Y-score:94.72, P-score:22.22,
HCD-MS/MS Scan:6934, SNR=0.8, Base Peak Intensity=4222162.5

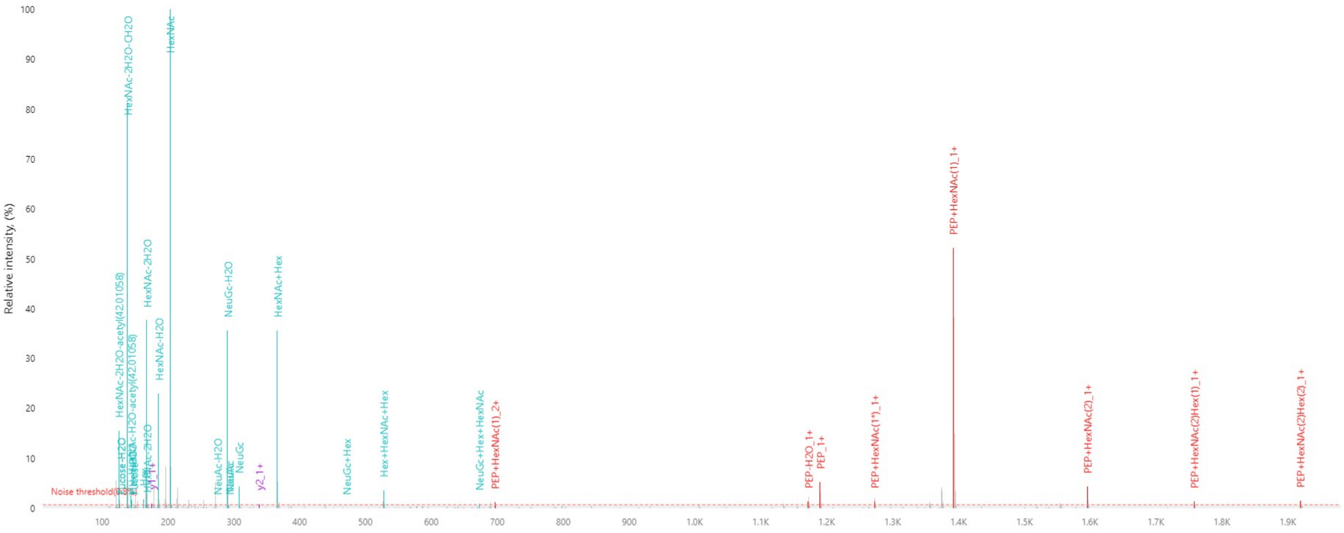

EEQYNSTYR(=PEP)_4_3_0_0_1, m/z:918.6904(3+), RT:31.69, HCD-score:91.50, Y-score:94.72, P-score:22.22,
CID-MS/MS Scan:6936, SNR=0.8, Base Peak Intensity=6012656

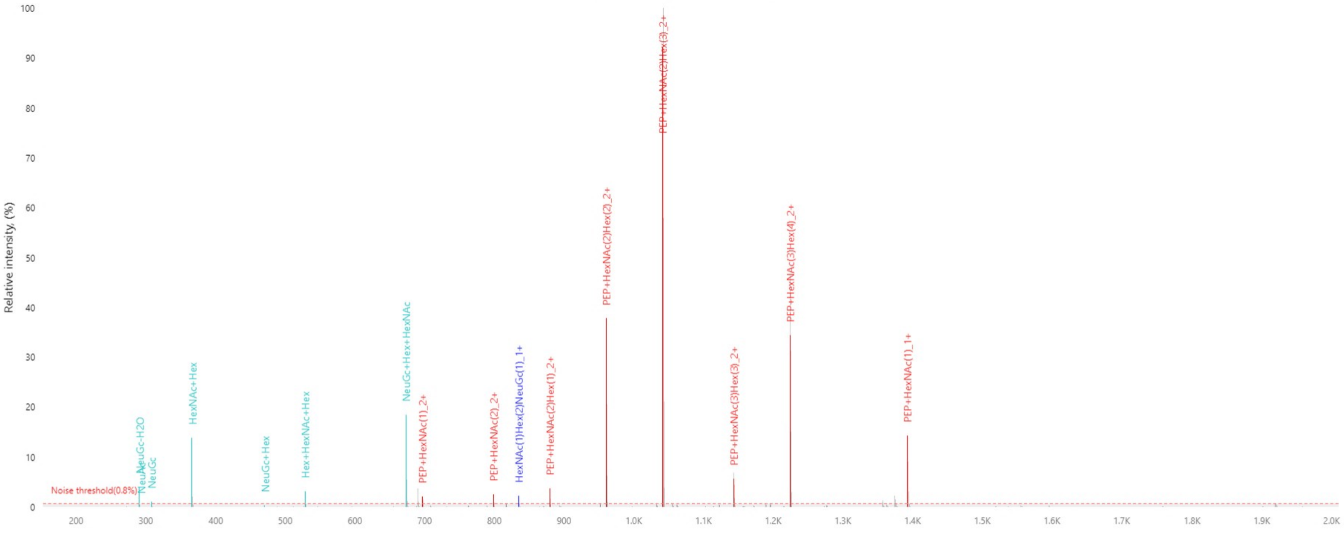

EEQYNSTYR(=PEP)_4_3_1_0_0, m/z:1297.0132(2+), RT:22.65, HCD-score:96.06, Y-score:86.97, P-score:0.00,
HCD-MS/MS Scan:4188, SNR=0.8, Base Peak Intensity=2329617

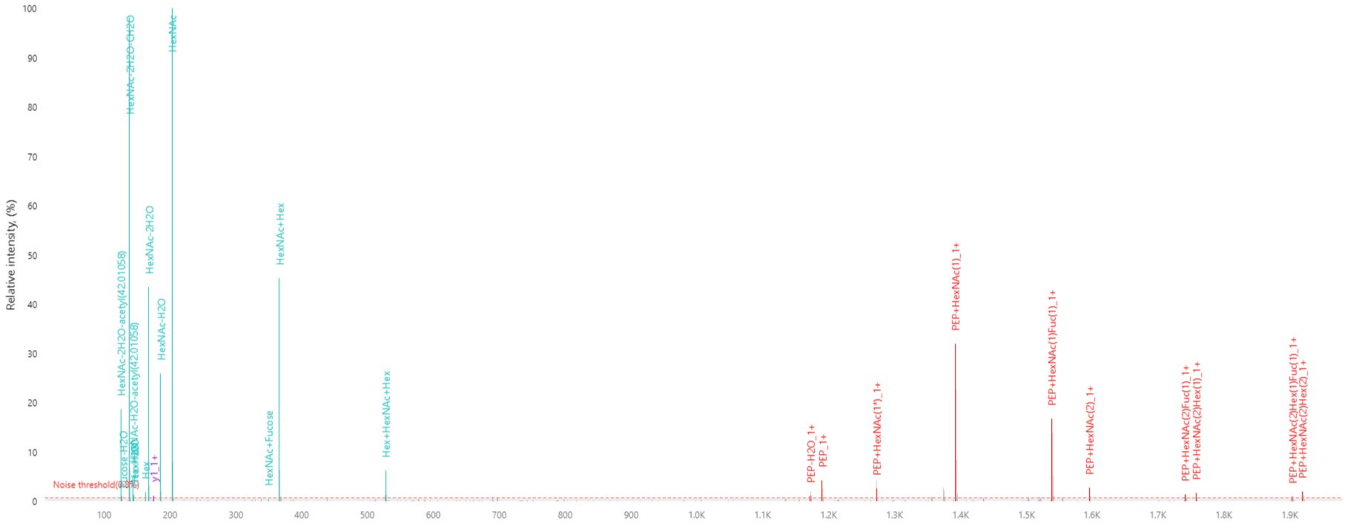

EEQYNSTYR(=PEP)_4_3_1_0_0, m/z:1297.0132(2+), RT:22.66, HCD-score:96.06, Y-score:86.97, P-score:0.00,
CID-MS/MS Scan:4191, SNR=0.8, Base Peak Intensity=1811612.6

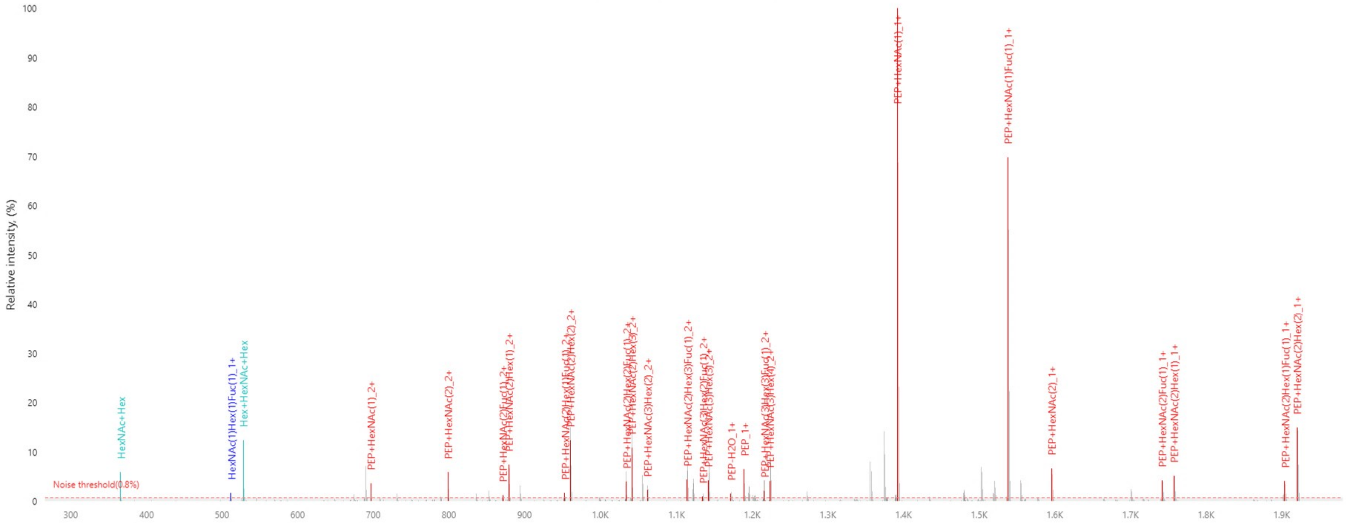

EEQYNSTYR(=PEP)_4_3_1_0_1, m/z:967.3807(3+), RT:31.43, HCD-score:90.77, Y-score:95.46, P-score:22.22,
HCD-MS/MS Scan:6818, SNR=0.8, Base Peak Intensity=18819862

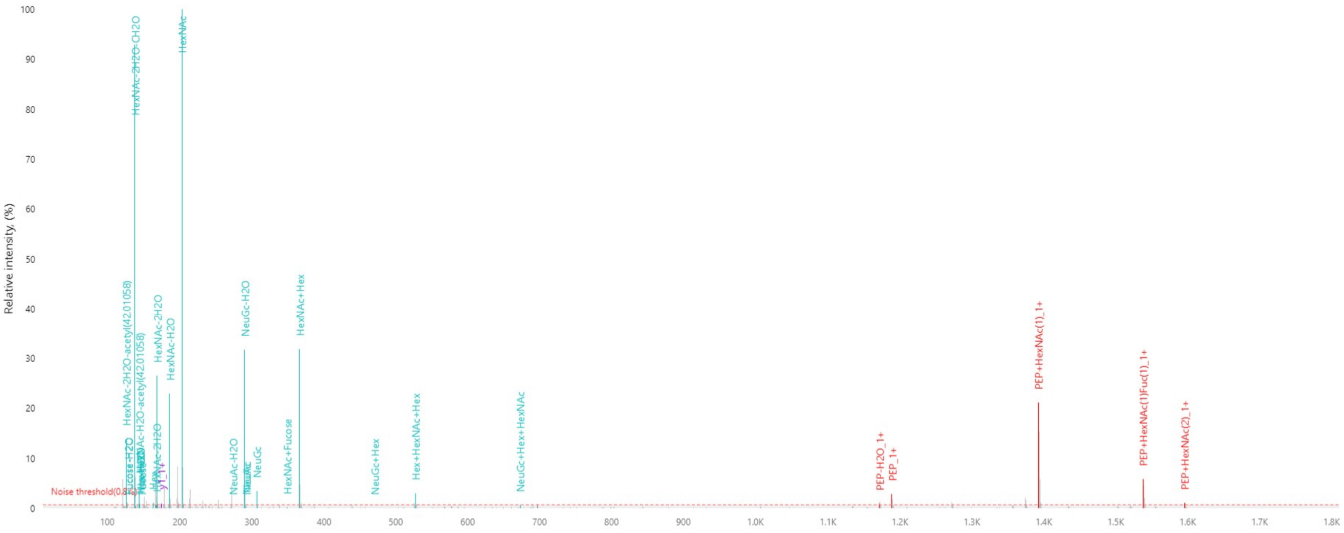

EEQYNSTYR(=PEP)_4_3_1_0_1, m/z:967.3807(3+), RT:31.44, HCD-score:90.77, Y-score:95.46, P-score:22.22,
CID-MS/MS Scan:6820, SNR=0.8, Base Peak Intensity=27603172

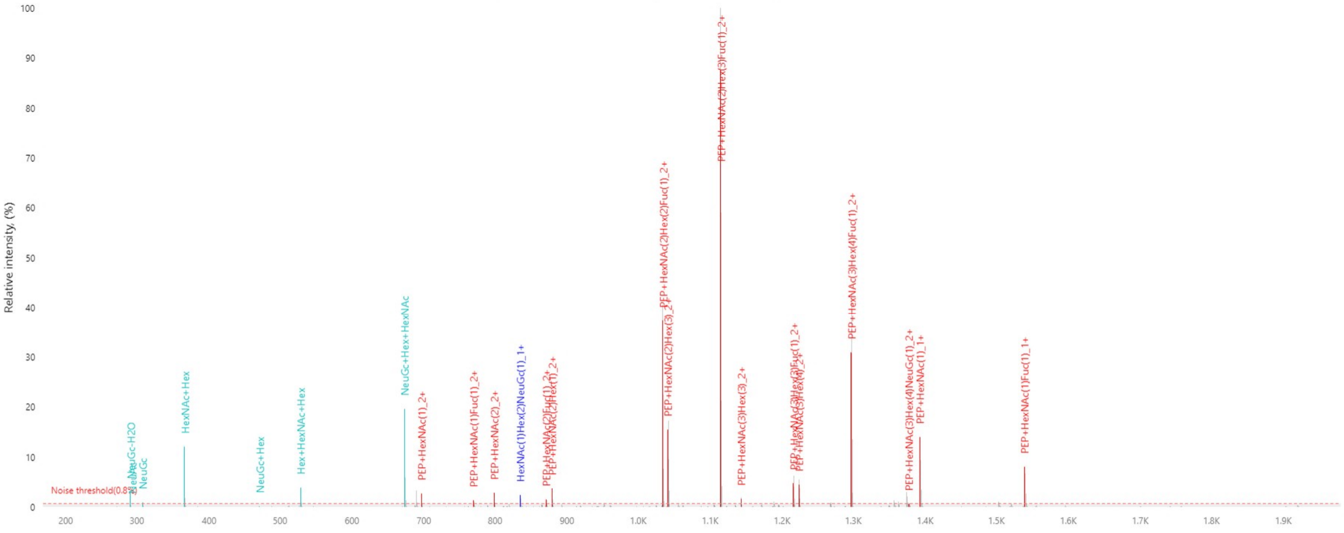

EEQYNSTYR(=PEP)_4_3_1_1_0, m/z:962.0452(3+), RT:31.89, HCD-score:94.34, Y-score:97.57, P-score:11.11,
HCD-MS/MS Scan:6933, SNR=0.8, Base Peak Intensity=306889.3

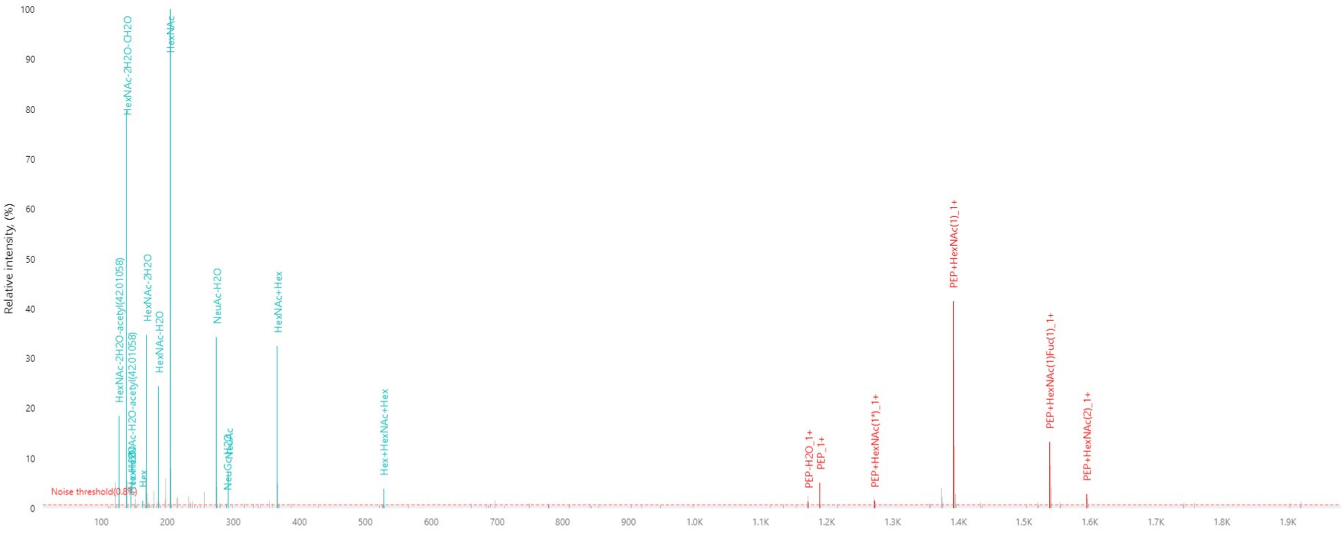

EEQYNSTYR(=PEP)_4_3_1_1_0, m/z:962.0452(3+), RT:31.90, HCD-score:94.34, Y-score:97.57, P-score:11.11,
CID-MS/MS Scan:6936, SNR=0.8, Base Peak Intensity=332781.3

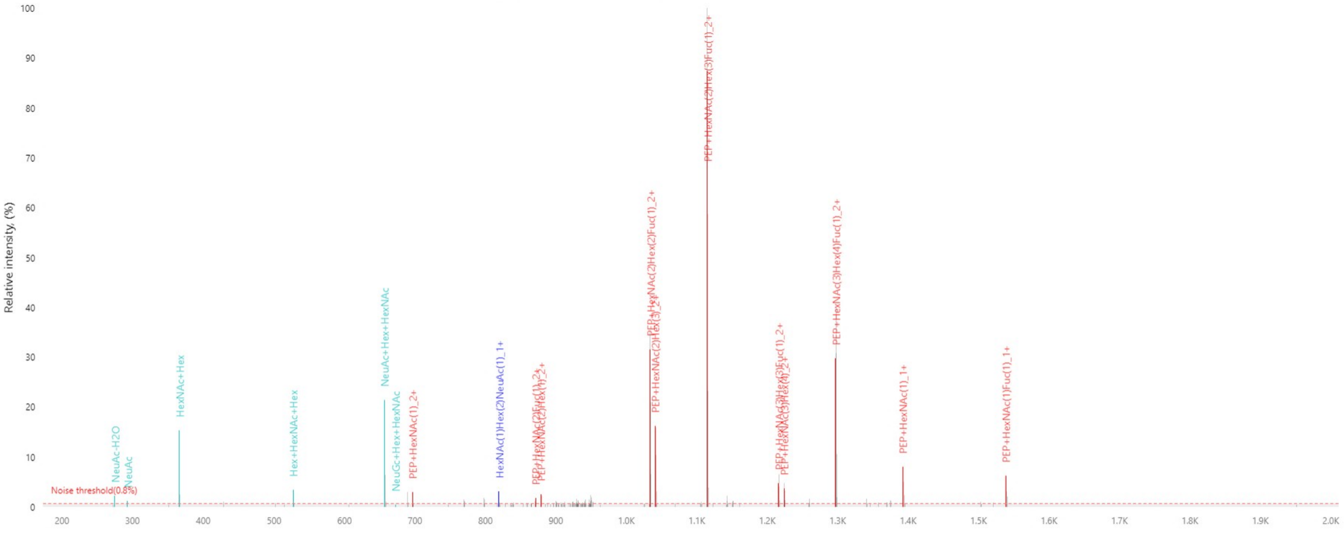

EEQYNSTYR(=PEP)_4_4_0_0_0, m/z:884.0192(3+), RT:23.18, HCD-score:95.44, Y-score:98.63, P-score:22.22,
HCD-MS/MS Scan:4396, SNR=0.8, Base Peak Intensity=3375866.2

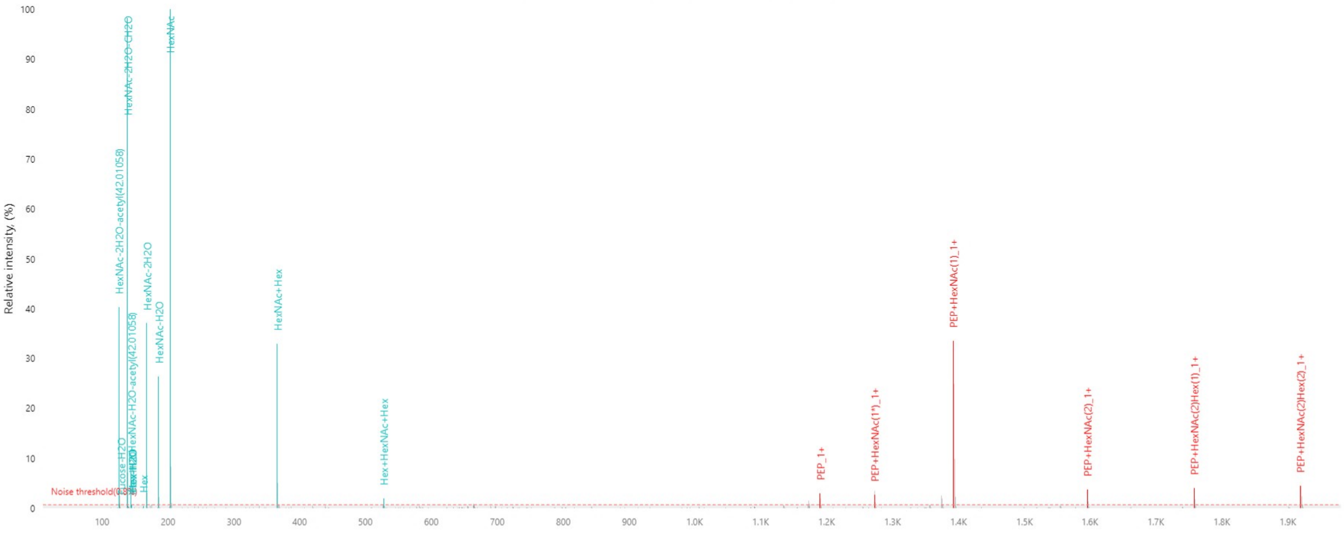

EEQYNSTYR(=PEP)_4_4_0_0_0, m/z:884.0192(3+), RT:23.19, HCD-score:95.44, Y-score:98.63, P-score:22.22,
CID-MS/MS Scan:4398, SNR=0.8, Base Peak Intensity=4798524

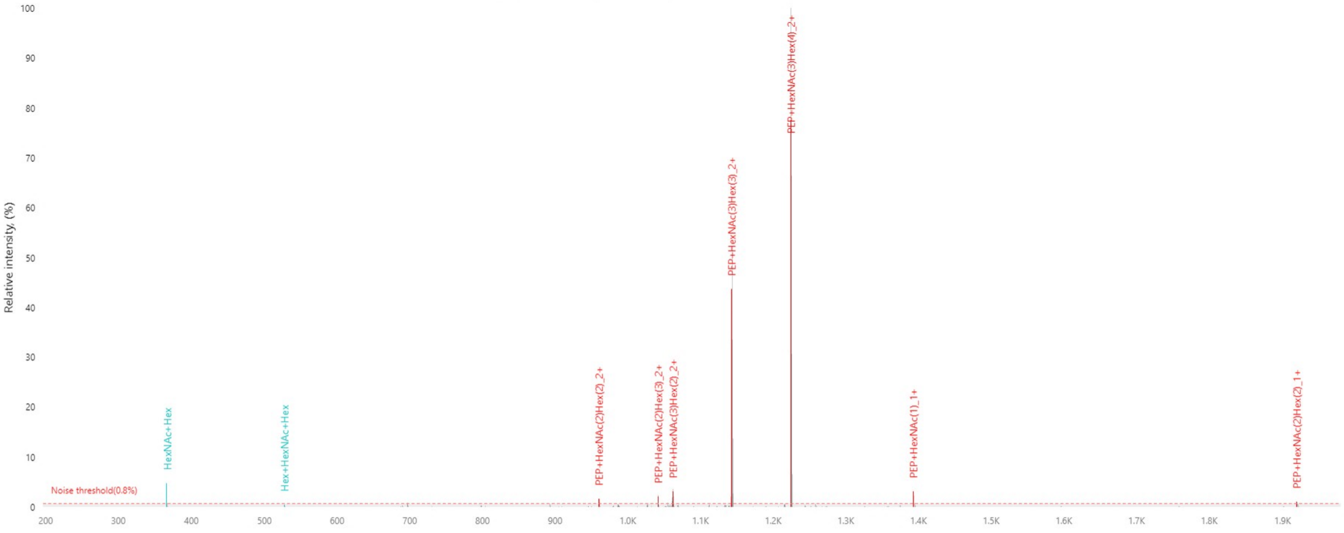

EEQYNSTYR(=PEP)_4_4_0_0_1, m/z:986.3831(3+), RT:31.71, HCD-score:80.83, Y-score:91.37, P-score:11.11,
HCD-MS/MS Scan:6959, SNR=0.8, Base Peak Intensity=1571781.5

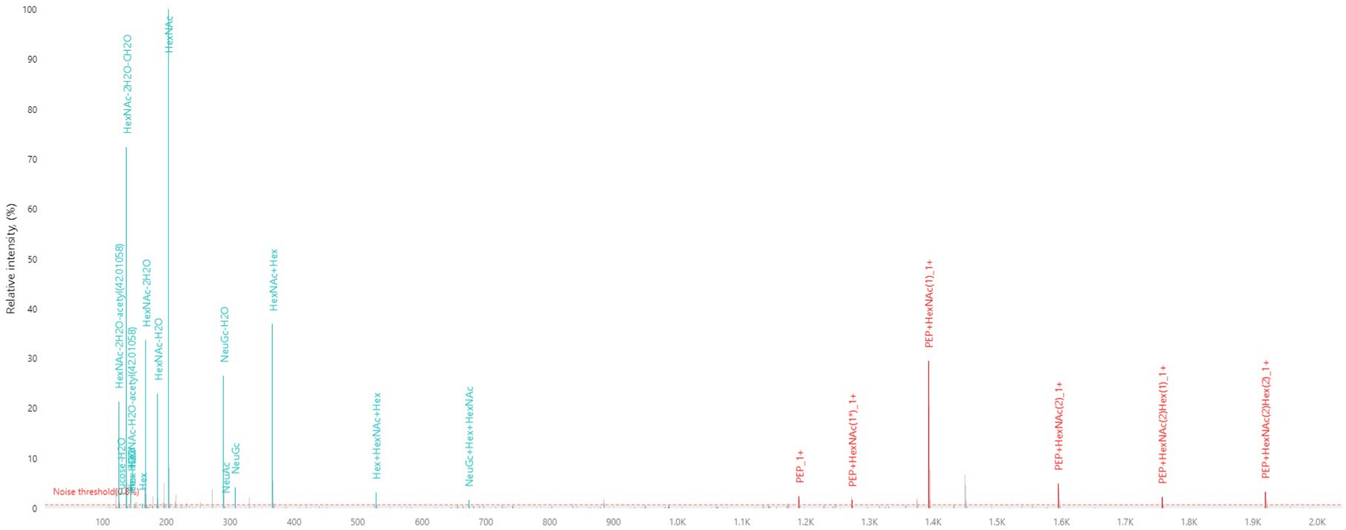

EEQYNSTYR(=PEP)_4_4_0_0_1, m/z:986.3831(3+), RT:31.71, HCD-score:80.83, Y-score:91.37, P-score:11.11,
CID-MS/MS Scan:6961, SNR=0.8, Base Peak Intensity=1048699.9

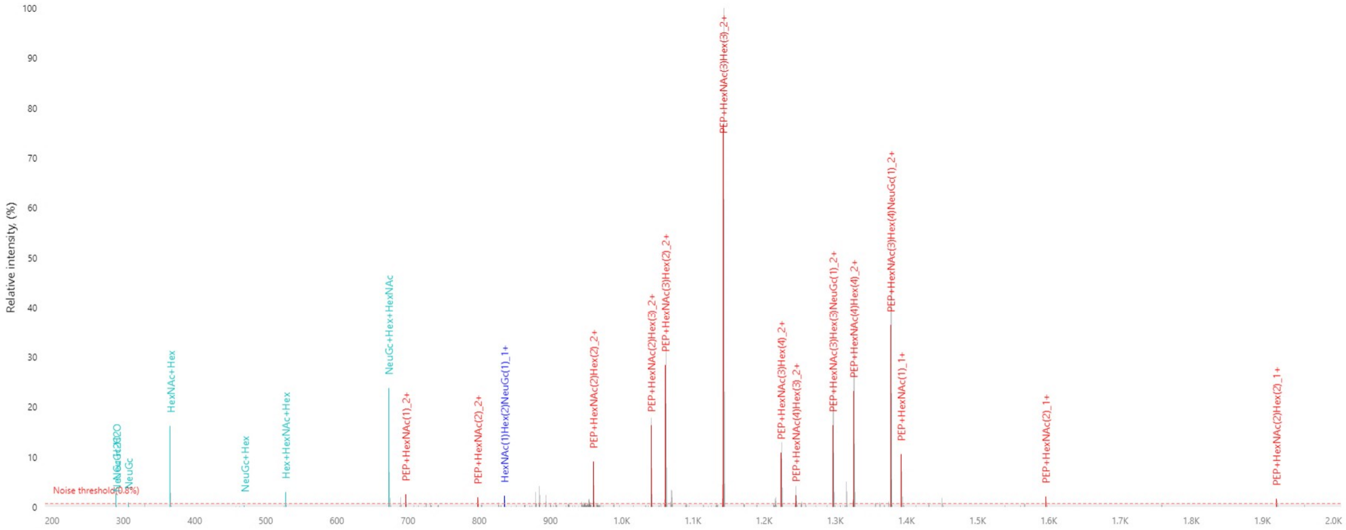

EEQYNSTYR(=PEP)_4_4_1_0_0, m/z:932.7054(3+), RT:23.30, HCD-score:96.23, Y-score:98.87, P-score:44.44,
HCD-MS/MS Scan:4455, SNR=0.8, Base Peak Intensity=577870720

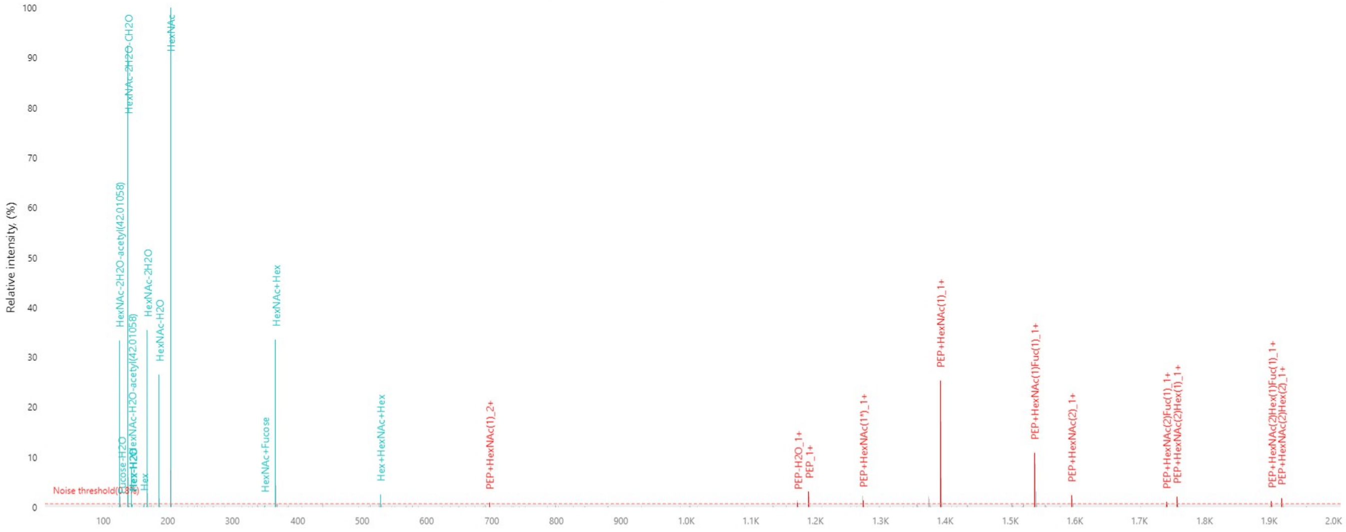

EEQYNSTYR(=PEP)_4_4_1_0_0, m/z:932.7054(3+), RT:23.30, HCD-score:96.23, Y-score:98.87, P-score:44.44,
CID-MS/MS Scan:4457, SNR=0.8, Base Peak Intensity=315926272

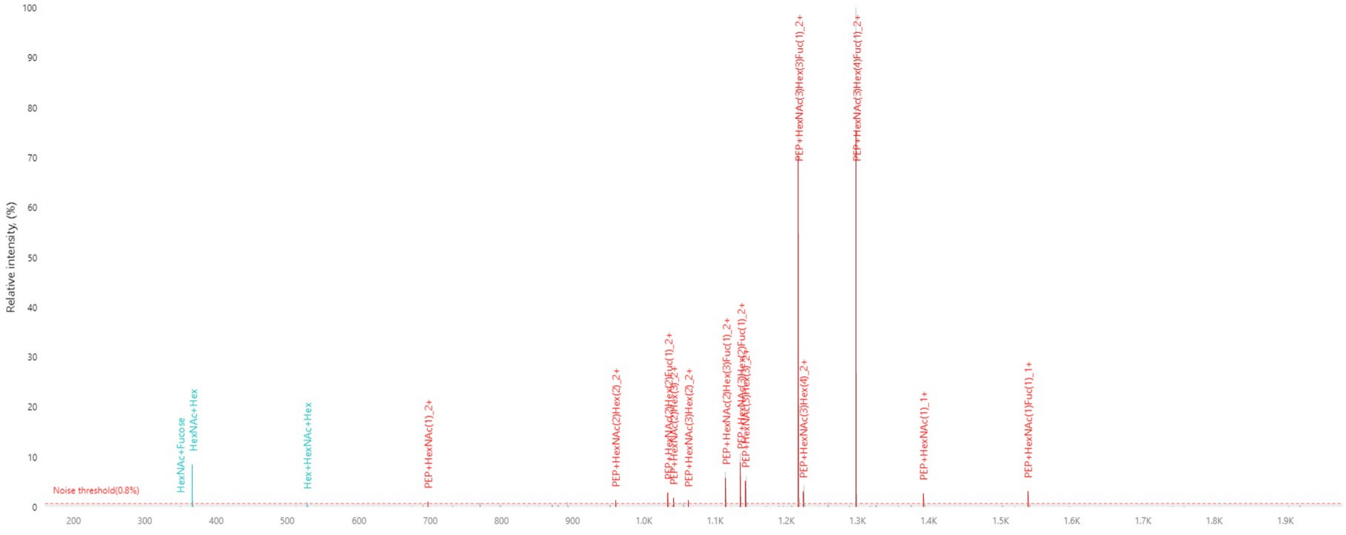

EEQYNSTYR(=PEP)_4_4_1_0_1, m/z:1035.0702(3+), RT:31.51, HCD-score:100.00, Y-score:99.42, P-score:11.11,
HCD-MS/MS Scan:7076, SNR=0.8, Base Peak Intensity=43990812

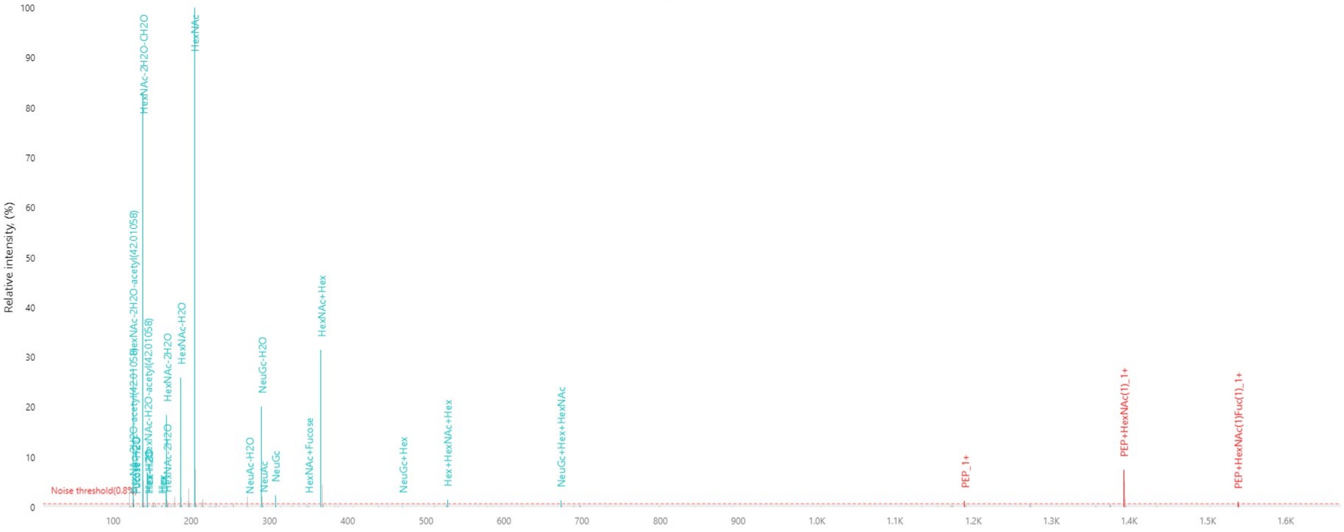

EEQYNSTYR(=PEP)_4_4_1_0_1, m/z:1035.0702(3+), RT:31.51, HCD-score:100.00, Y-score:99.42, P-score:11.11,
CID-MS/MS Scan:7078, SNR=0.8, Base Peak Intensity=52632212

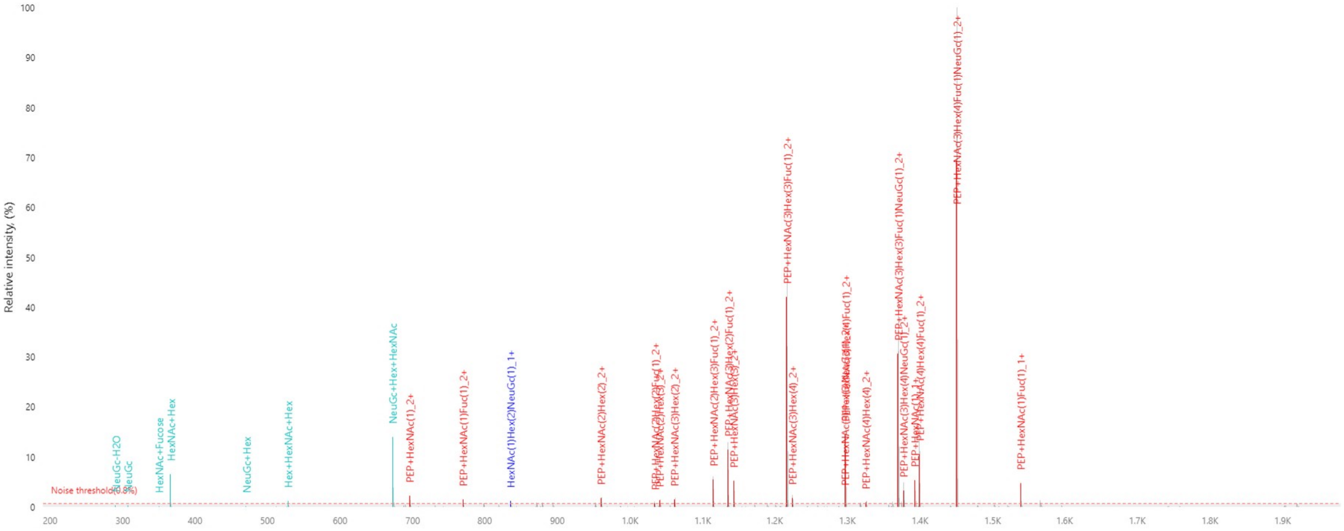

EEQYNSTYR(=PEP)_4_4_1_1_0, m/z:1029.7379(3+), RT:32.02, HCD-score:95.28, Y-score:96.98, P-score:22.22,
HCD-MS/MS Scan:6937, SNR=0.8, Base Peak Intensity=1392513.9

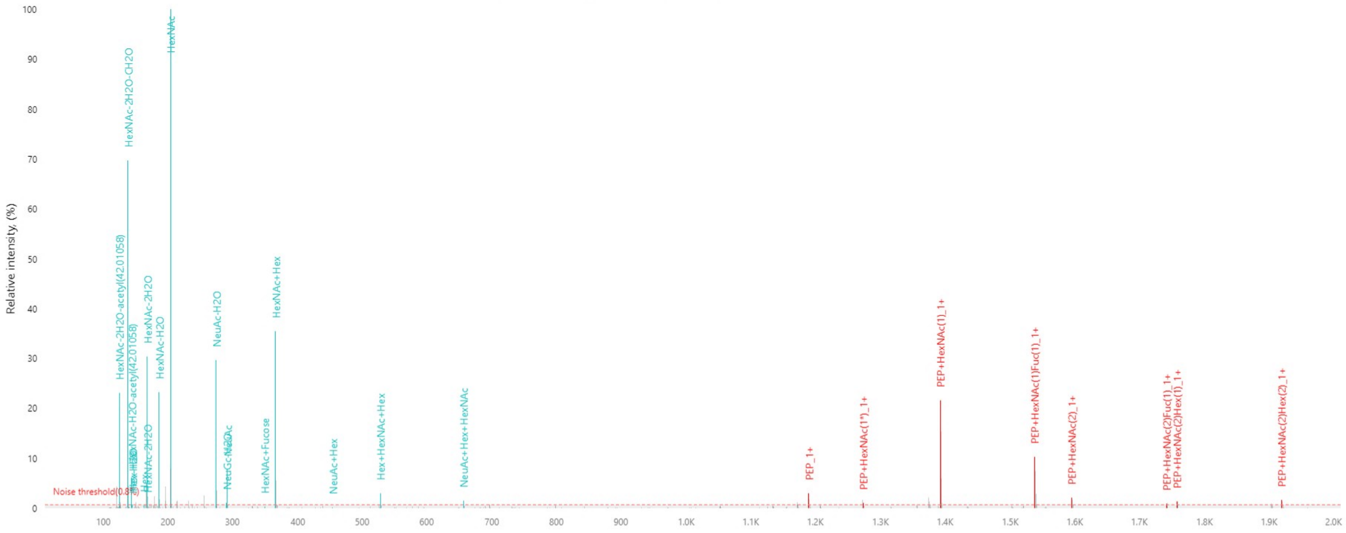

EEQYNSTYR(=PEP)_4_4_1_1_0, m/z:1029.7379(3+), RT:32.02, HCD-score:95.28, Y-score:96.98, P-score:22.22,
CID-MS/MS Scan:6939, SNR=0.8, Base Peak Intensity=770891.8

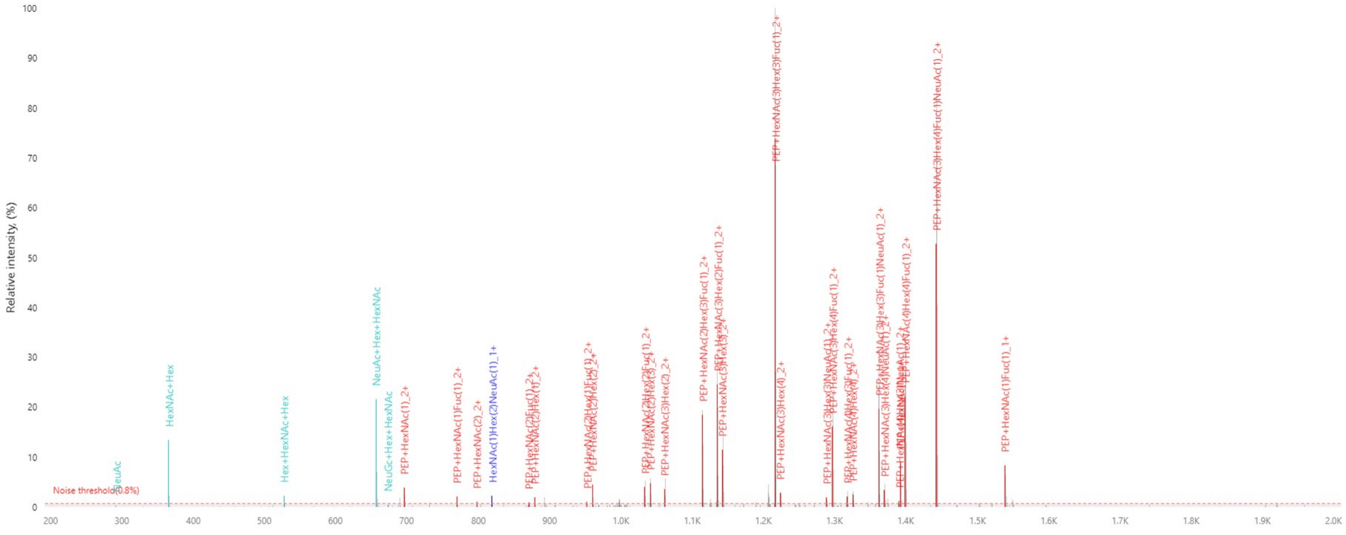

EEQYNSTYR(=PEP)_4_5_0_0_0, m/z:951.7128(3+), RT:23.52, HCD-score:27.28, Y-score:72.77, P-score:0.00,
HCD-MS/MS Scan:4433, SNR=0.8, Base Peak Intensity=1823989.9

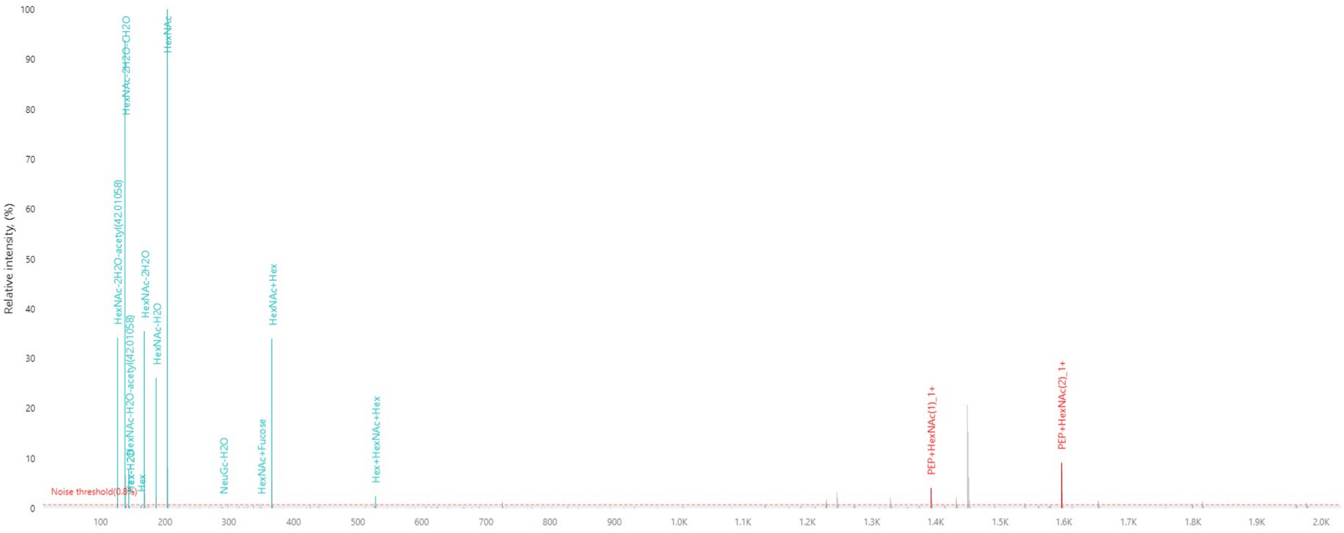

EEQYNSTYR(=PEP)_4_5_0_0_0, m/z:951.7128(3+), RT:23.53, HCD-score:27.28, Y-score:72.77, P-score:0.00,
CID-MS/MS Scan:4435, SNR=0.8, Base Peak Intensity=1743233.6

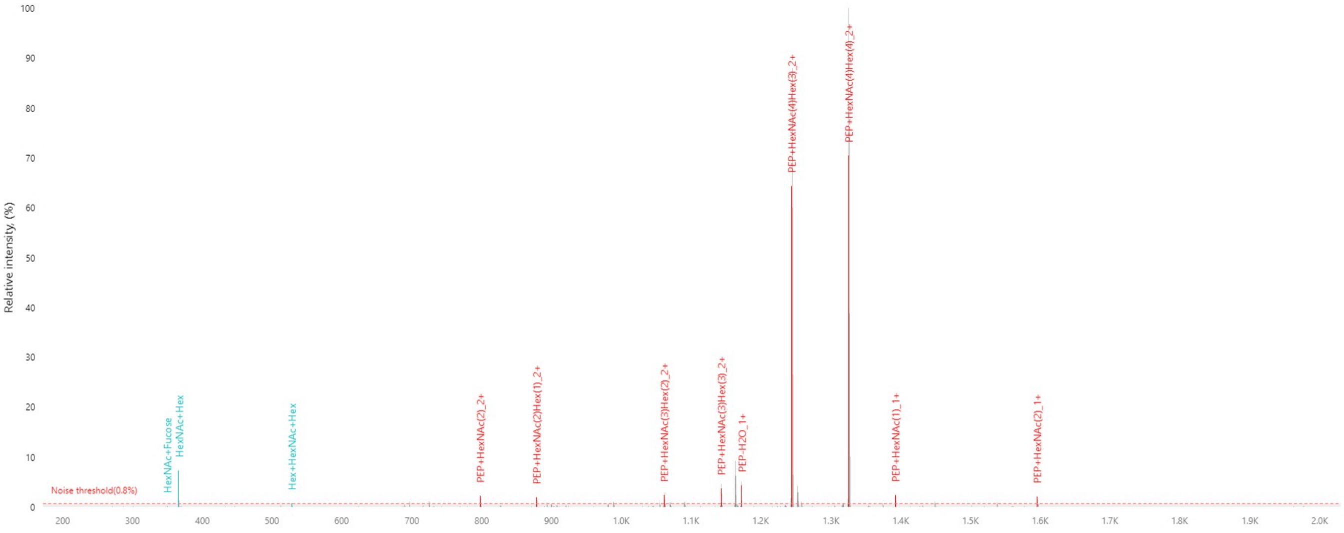

EEQYNSTYR(=PEP)_4_5_1_0_0, m/z:1000.3970(3+), RT:23.77, HCD-score:96.27, Y-score:98.45, P-score:44.44,
HCD-MS/MS Scan:4810, SNR=0.8, Base Peak Intensity=2544092.2

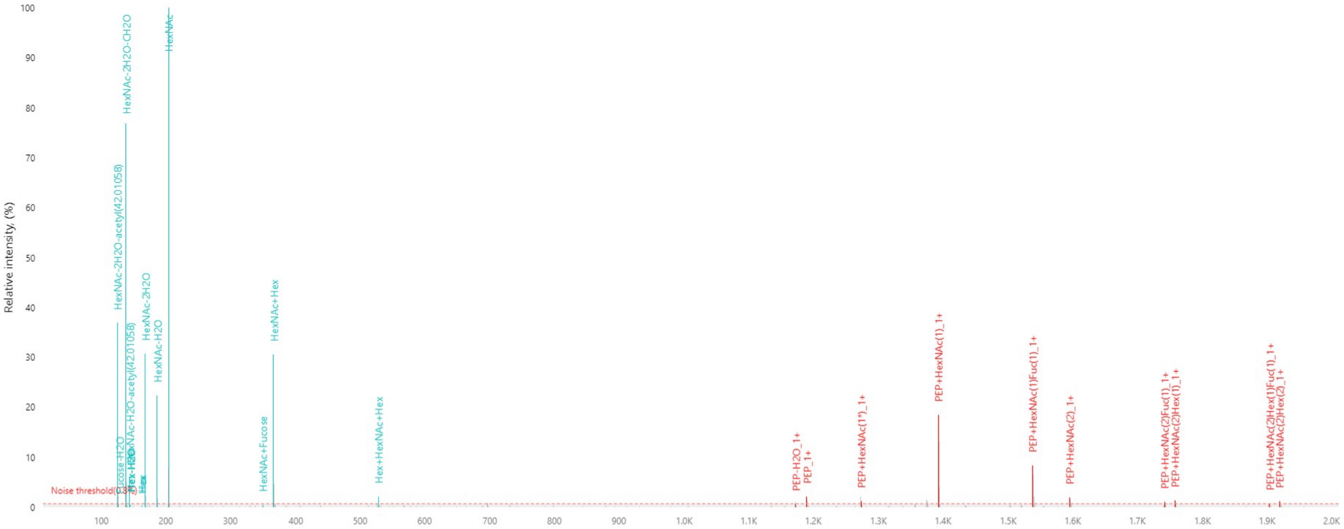

EEQYNSTYR(=PEP)_4_5_1_0_0, m/z:1000.3970(3+), RT:23.78, HCD-score:96.27, Y-score:98.45, P-score:44.44,
CID-MS/MS Scan:4812, SNR=0.8, Base Peak Intensity=2555129.5

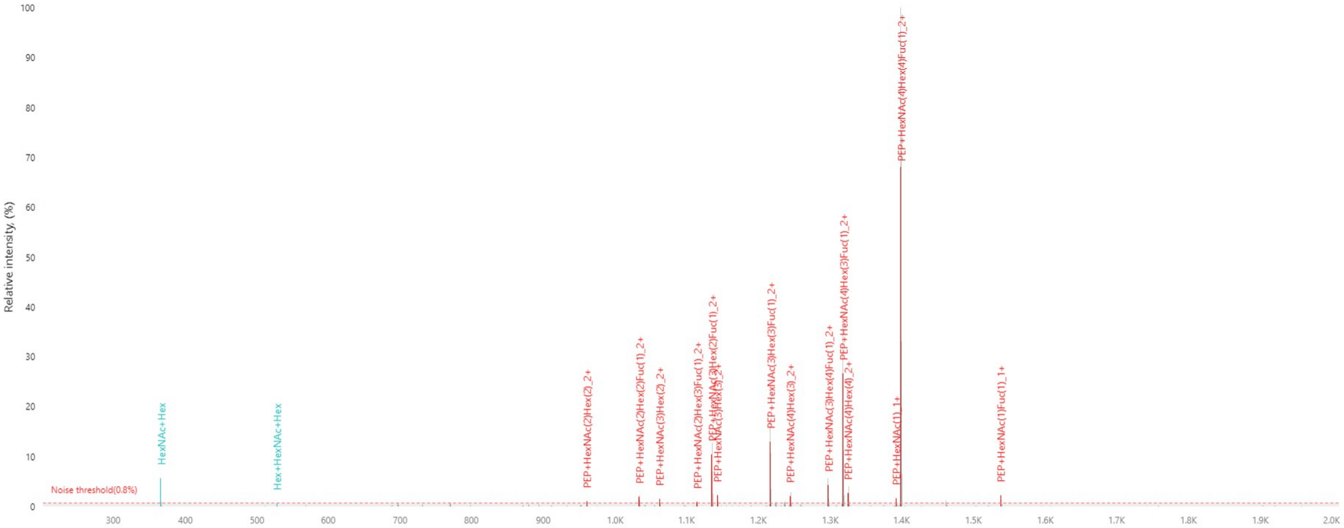

EEQYNSTYR(=PEP)_5_2_0_0_0, m/z:802.6557(3+), RT:22.02, HCD-score:85.66, Y-score:91.08, P-score:55.56,
HCD-MS/MS Scan:4062, SNR=0.8, Base Peak Intensity=717223.2

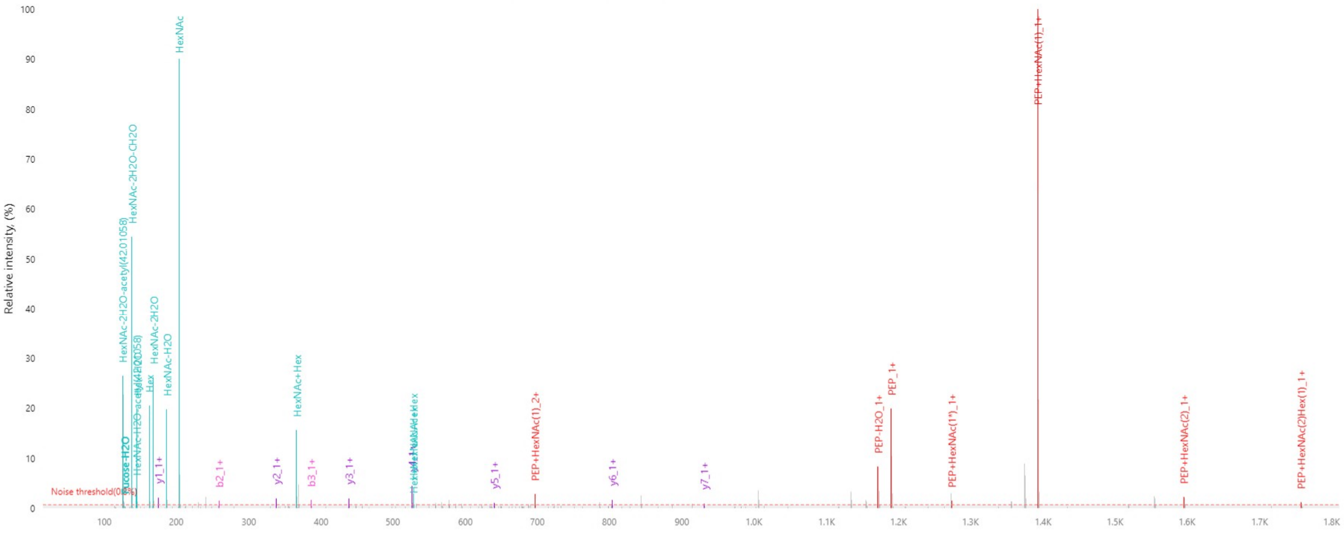

EEQYNSTYR(=PEP)_5_2_0_0_0, m/z:802.6557(3+), RT:22.02, HCD-score:85.66, Y-score:91.08, P-score:55.56,
CID-MS/MS Scan:4064, SNR=0.8, Base Peak Intensity=609551

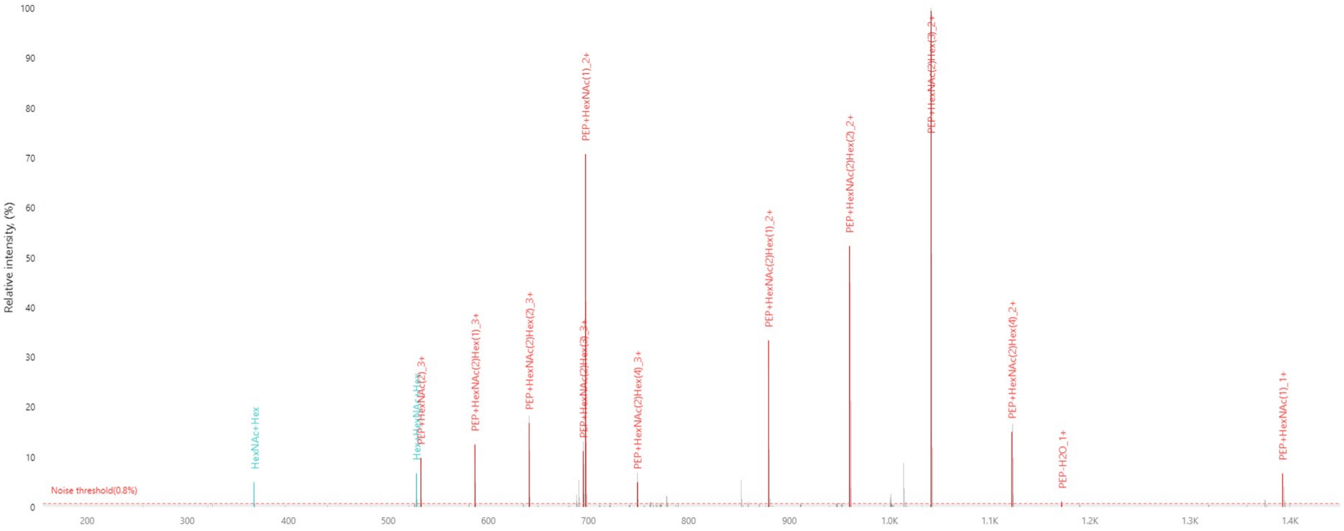

EEQYNSTYR(=PEP)_5_2_1_0_0, m/z:1276.5027(2+), RT:22.56, HCD-score:87.78, Y-score:79.90, P-score:0.00,
HCD-MS/MS Scan:4465, SNR=0.8, Base Peak Intensity=1371223.9

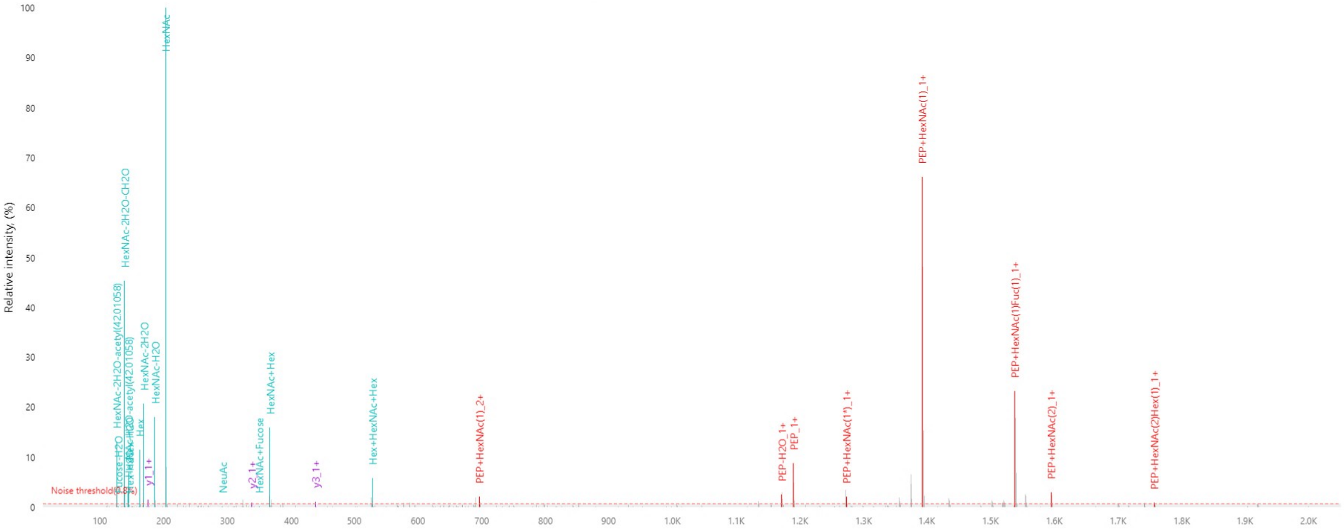

EEQYNSTYR(=PEP)_5_2_1_0_0, m/z:1276.5027(2+), RT:22.57, HCD-score:87.78, Y-score:79.90, P-score:0.00,
CID-MS/MS Scan:4468, SNR=0.8, Base Peak Intensity=1225096.1

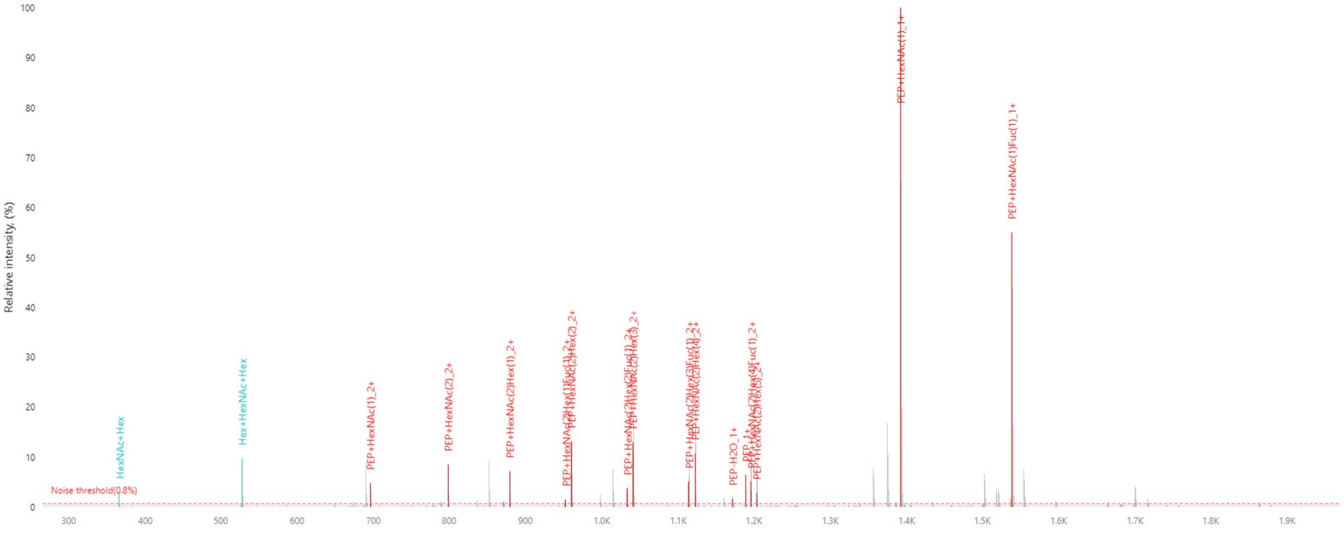

EEQYNSTYR(=PEP)_5_3_0_0_0, m/z:870.3453(3+), RT:22.47, HCD-score:91.02, Y-score:97.31, P-score:0.00,
HCD-MS/MS Scan:4211, SNR=0.8, Base Peak Intensity=18378344

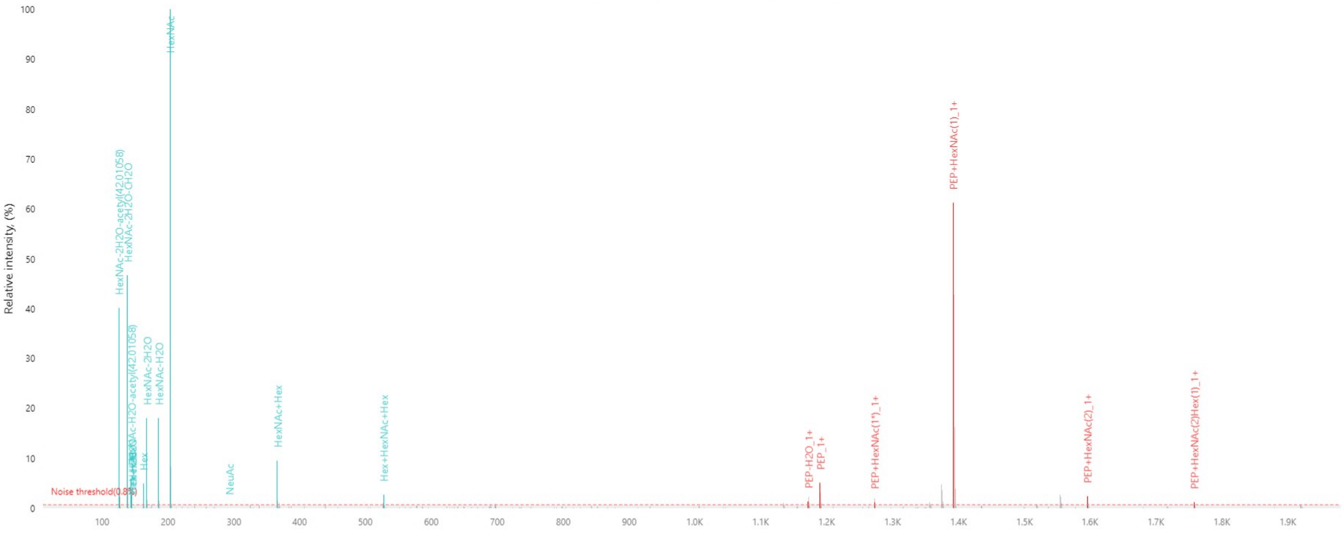

EEQYNSTYR(=PEP)_5_3_0_0_0, m/z:870.3453(3+), RT:22.48, HCD-score:91.02, Y-score:97.31, P-score:0.00,
CID-MS/MS Scan:4213, SNR=0.8, Base Peak Intensity=17629560

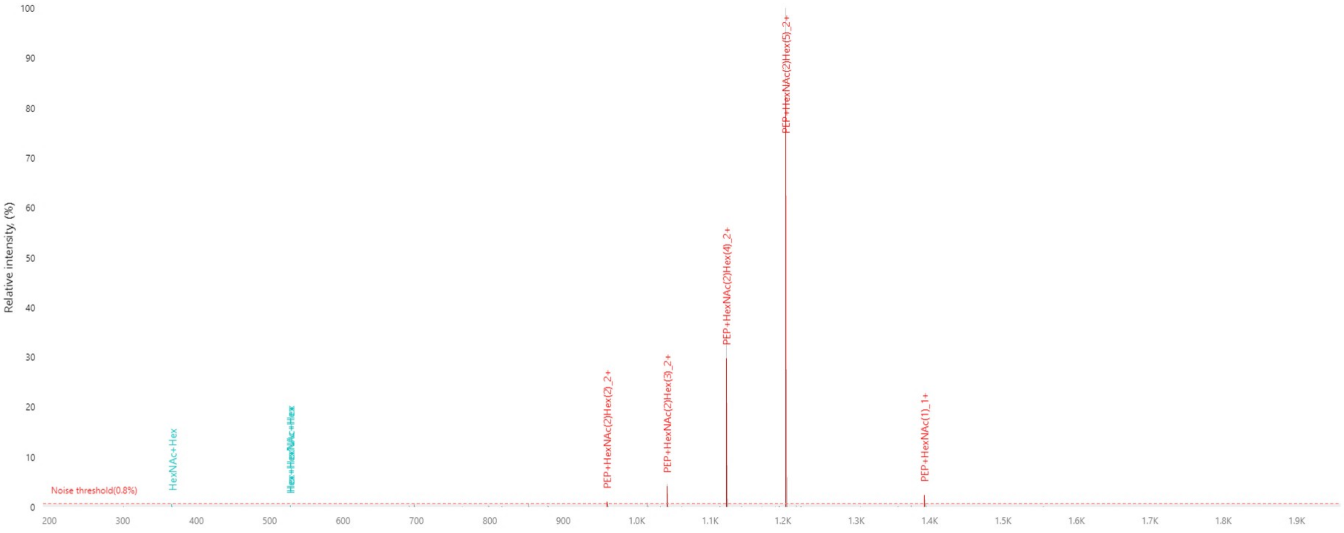

EEQYNSTYR(=PEP)_5_3_0_0_1, m/z:972.7076(3+), RT:31.58, HCD-score:90.71, Y-score:92.24, P-score:22.22,
HCD-MS/MS Scan:6811, SNR=0.8, Base Peak Intensity=3861600

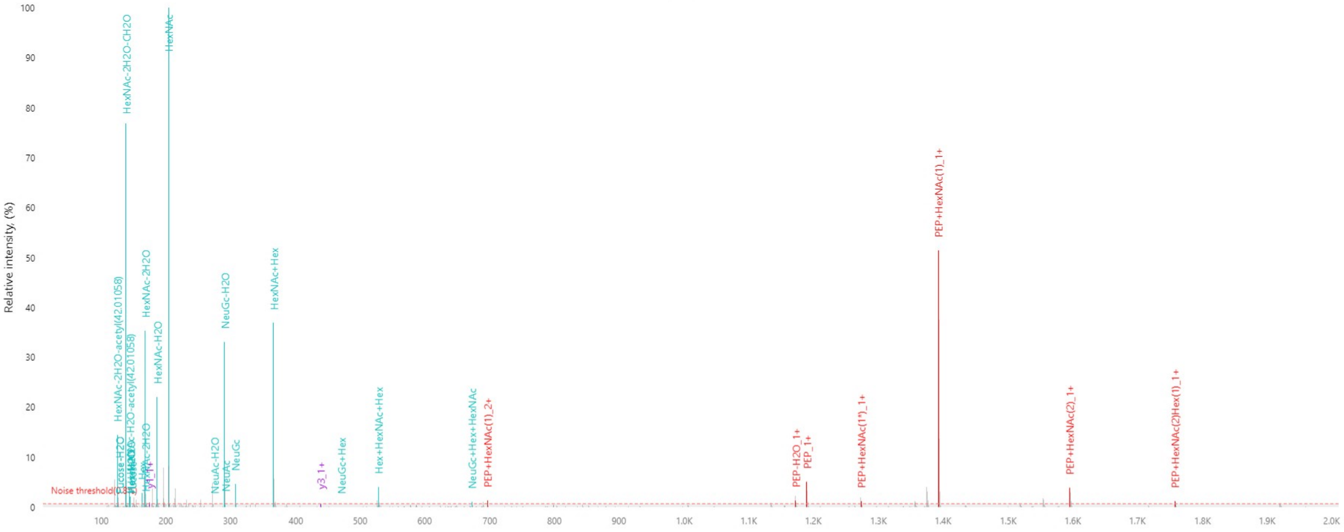

EEQYNSTYR(=PEP)_5_3_0_0_1, m/z:972.7076(3+), RT:31.59, HCD-score:90.71, Y-score:92.24, P-score:22.22,
CID-MS/MS Scan:6813, SNR=0.8, Base Peak Intensity=4894903

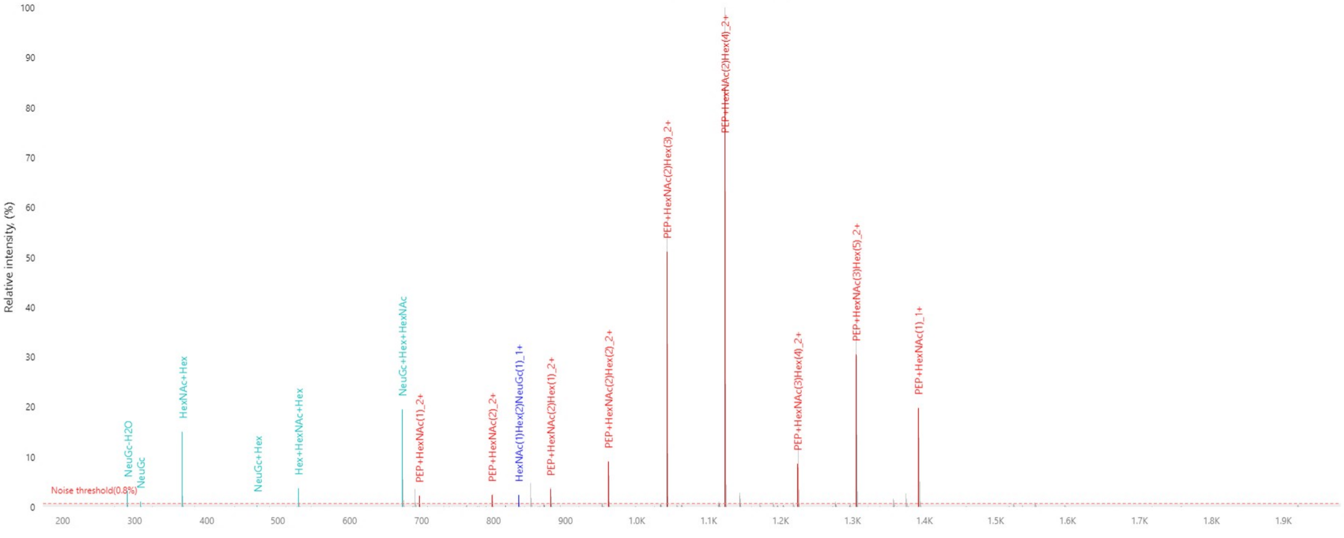

Supplement: Supplementary file 1 [file Image2.pdf]
