# Supplementary material for: Advanced assessment through intact glycopeptide analysis of Infliximab’s biologics and biosimilar
Source: Front Mol Biosci. 2022 Nov 29;9:1006866. doi: 10.3389/fmolb.2022.1006866 (PMC9745114; doi:10.3389/fmolb.2022.1006866)

EEQYNSTYR(=PEP)_5_3_1_0_0, m/z:919.0322(3+), RT:22.91, HCD-score:90.25, Y-score:94.84, P-score:44.44, HCD-MS/MS Scan:4352, SNR=0.8, Base Peak Intensity=2122037.5

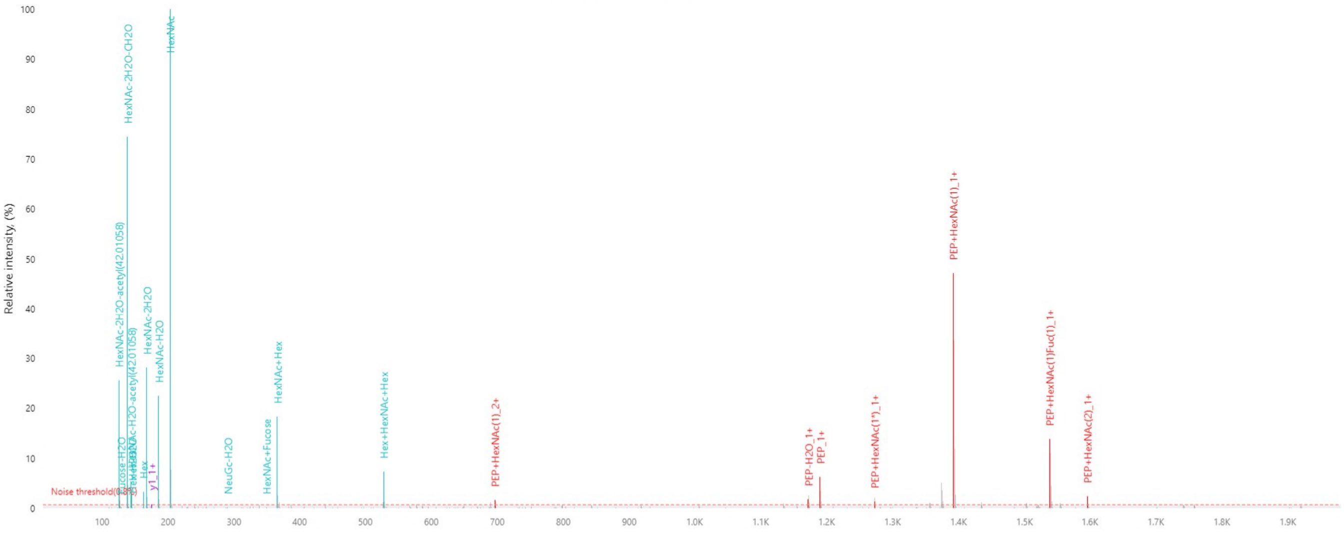

EEQYNSTYR(=PEP)_5_3_1_0_0, m/z:919.0322(3+), RT:22.92, HCD-score:90.25, Y-score:94.84, P-score:44.44, CID-MS/MS Scan:4354, SNR=0.8, Base Peak Intensity=1640042.4

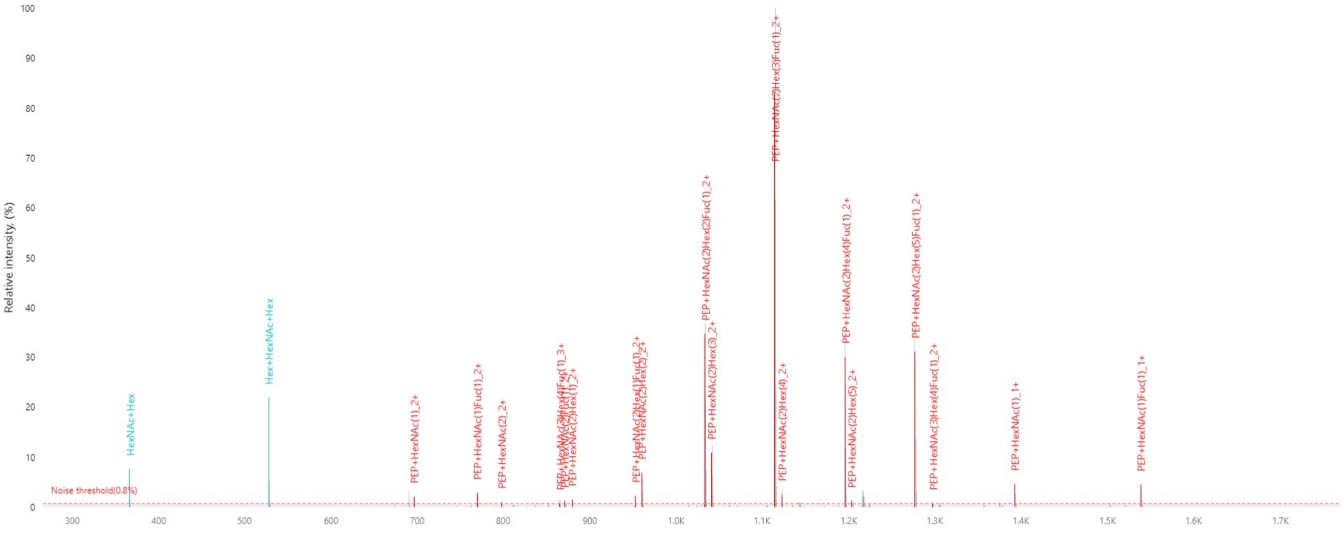

EEQYNSTYR(=PEP)_5_3_1_0_1, m/z:1021.3958(3+), RT:31.21, HCD-score:92.03, Y-score:94.27, P-score:0.00,
HCD-MS/MS Scan:6944, SNR=0.8, Base Peak Intensity=5152256

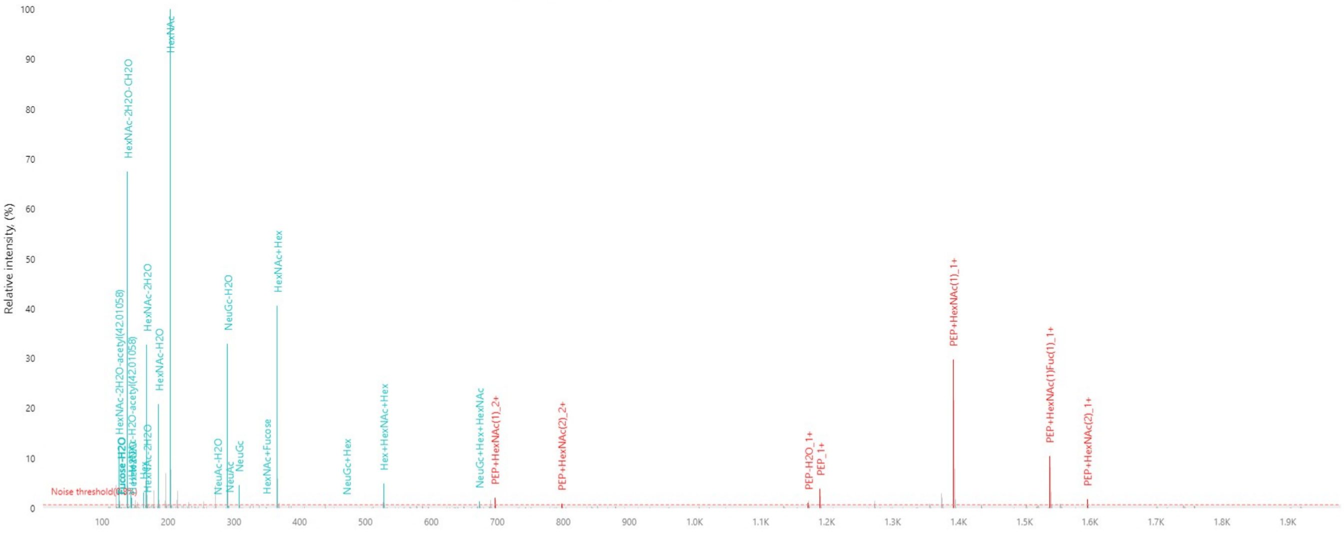

EEQYNSTYR(=PEP)_5_3_1_0_1, m/z:1021.3958(3+), RT:31.22, HCD-score:92.03, Y-score:94.27, P-score:0.00,
CID-MS/MS Scan:6947, SNR=0.8, Base Peak Intensity=5229559.5

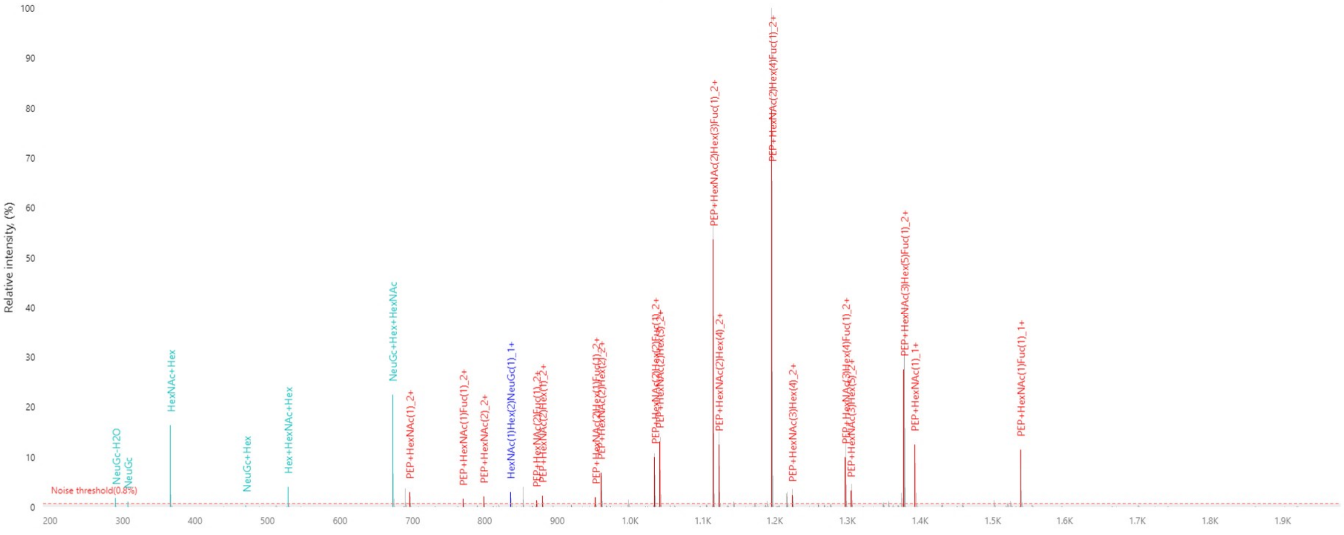

EEQYNSTYR(=PEP)_5_4_1_0_0, m/z:986.7244(3+), RT:23.22, HCD-score:96.46, Y-score:97.97, P-score:33.33,
HCD-MS/MS Scan:4373, SNR=0.8, Base Peak Intensity=87666368

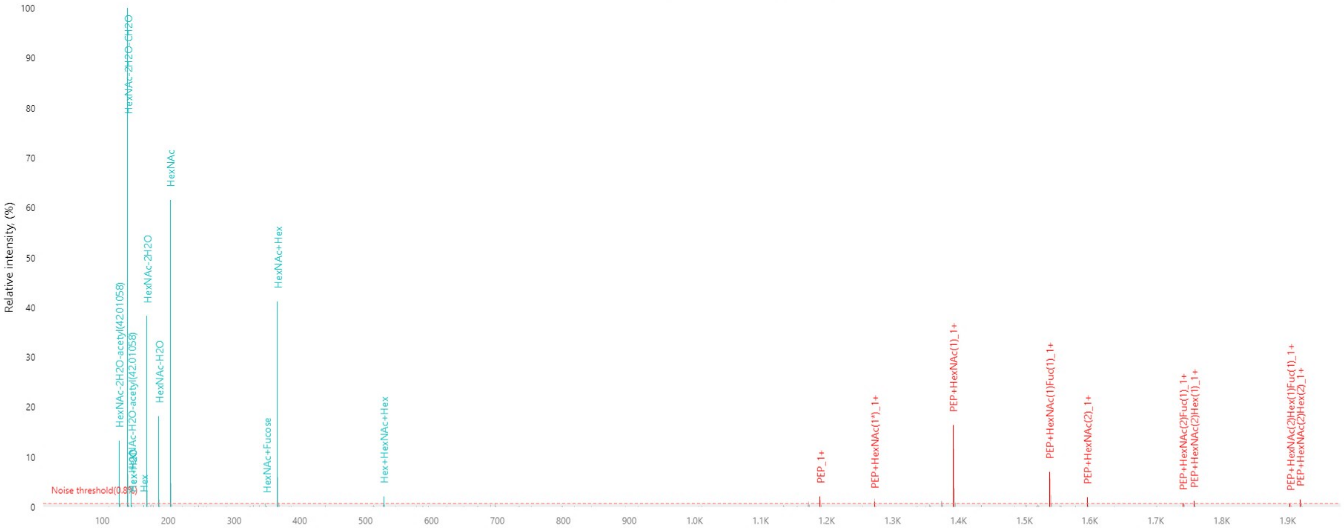

EEQYNSTYR(=PEP)_5_4_1_0_0, m/z:986.7244(3+), RT:23.23, HCD-score:96.46, Y-score:97.97, P-score:33.33,
CID-MS/MS Scan:4375, SNR=0.8, Base Peak Intensity=59404668

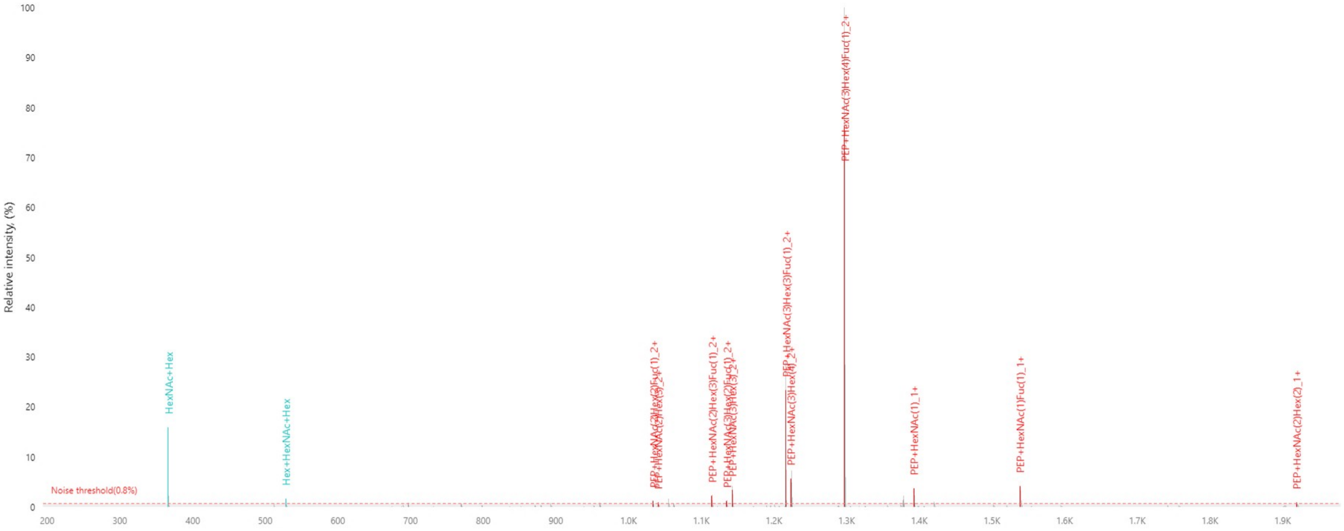

EEQYNSTYR(=PEP)_5_4_1_0_1, m/z:1089.0903(3+), RT:29.43, HCD-score:100.00, Y-score:100.00, P-score:11.11,
HCD-MS/MS Scan:6180, SNR=0.8, Base Peak Intensity=338406

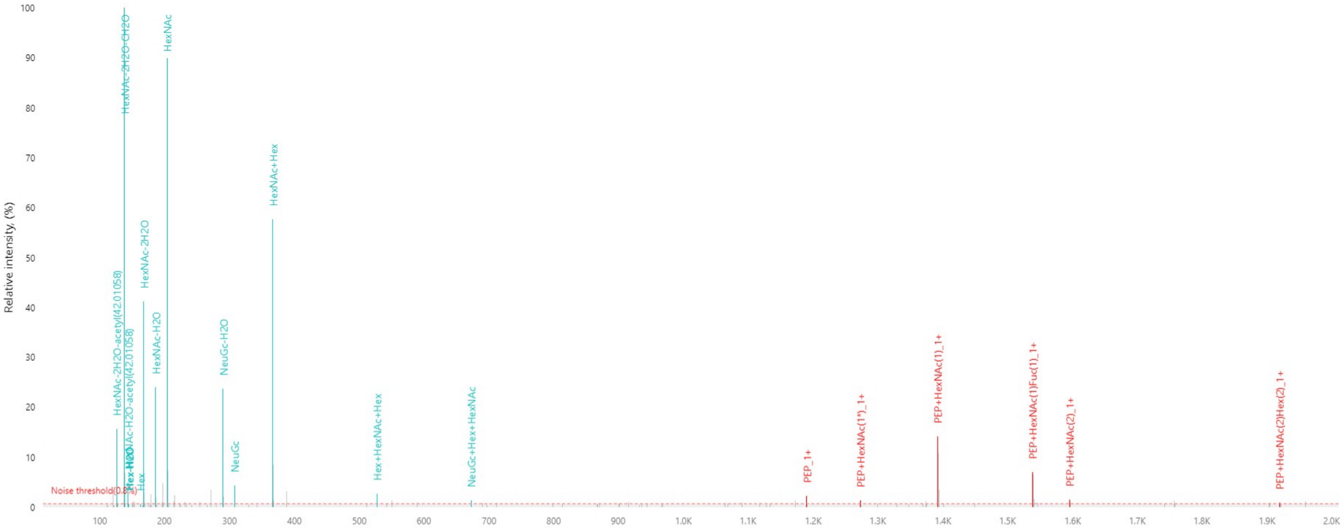

EEQYNSTYR(=PEP)_5_4_1_0_1, m/z:1089.0903(3+), RT:29.43, HCD-score:100.00, Y-score:100.00, P-score:11.11,
CID-MS/MS Scan:6182, SNR=0.8, Base Peak Intensity=261022

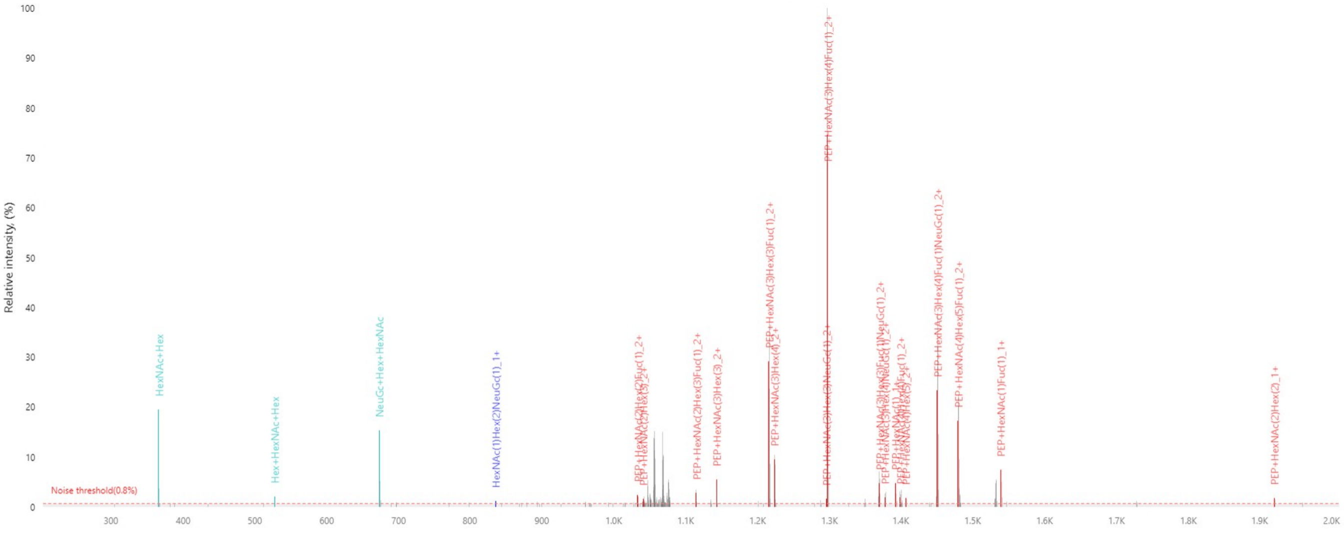

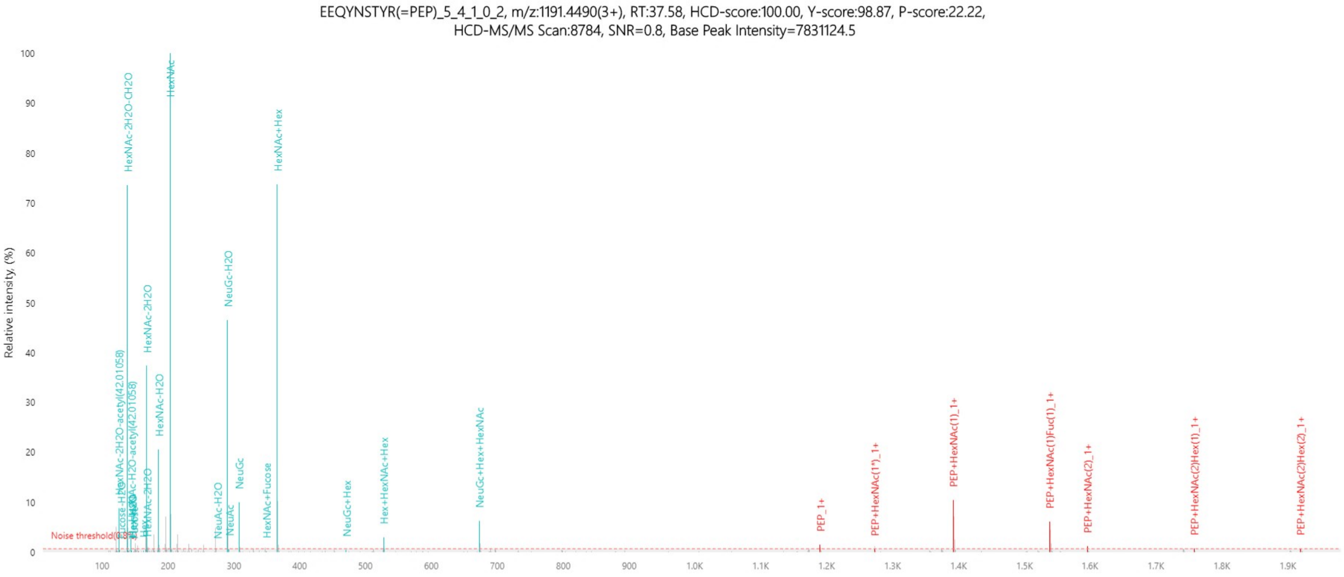
EEQYNSTYR(=PEP)_5_4_1_0_2, m/z:1191.4490(3+), RT:37.58, HCD-score:100.00, Y-score:98.87, P-score:22.22,
HCD-MS/MS Scan:8784, SNR=0.8, Base Peak Intensity=7831124.5
Relative intensity, (%)
Noise threshold(0.8%)

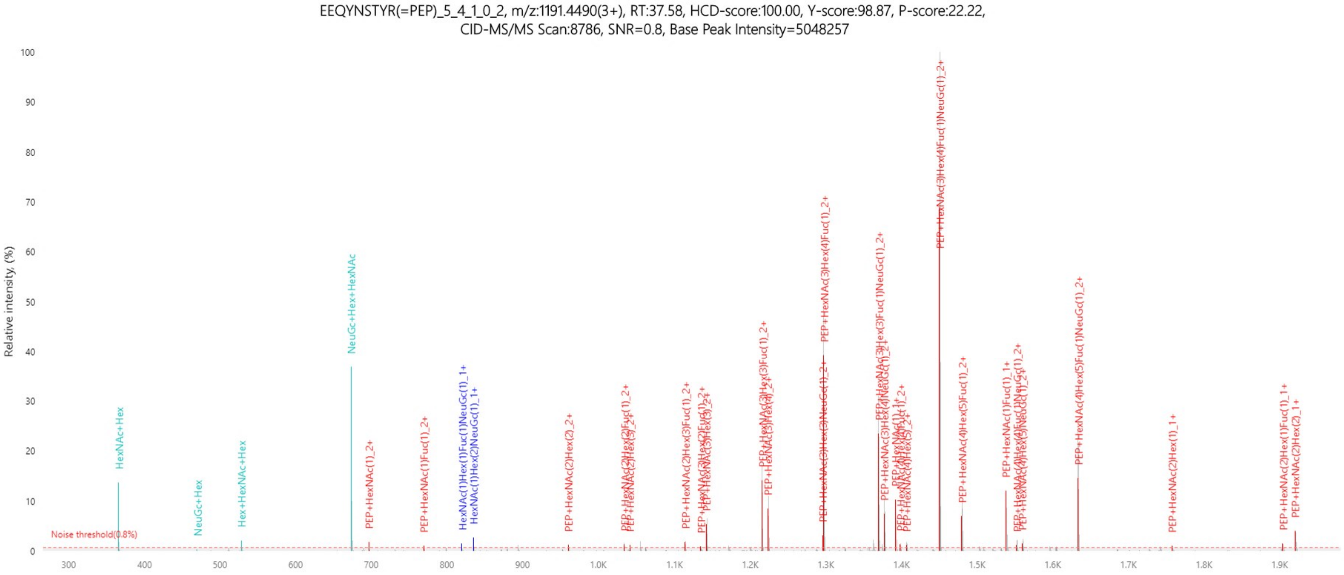
EEQYNSTYR(=PEP)_5_4_1_0_2, m/z:1191.4490(3+), RT:37.58, HCD-score:100.00, Y-score:98.87, P-score:22.22,
CID-MS/MS Scan:8786, SNR=0.8, Base Peak Intensity=5048257
Relative intensity, (%)
Noise threshold(0.8%)

EEQYNSTYR(=PEP)_5_4_1_1_0, m/z:1083.7549(3+), RT:31.90, HCD-score:96.14, Y-score:97.05, P-score:22.22,
HCD-MS/MS Scan:7157, SNR=0.8, Base Peak Intensity=898575.1

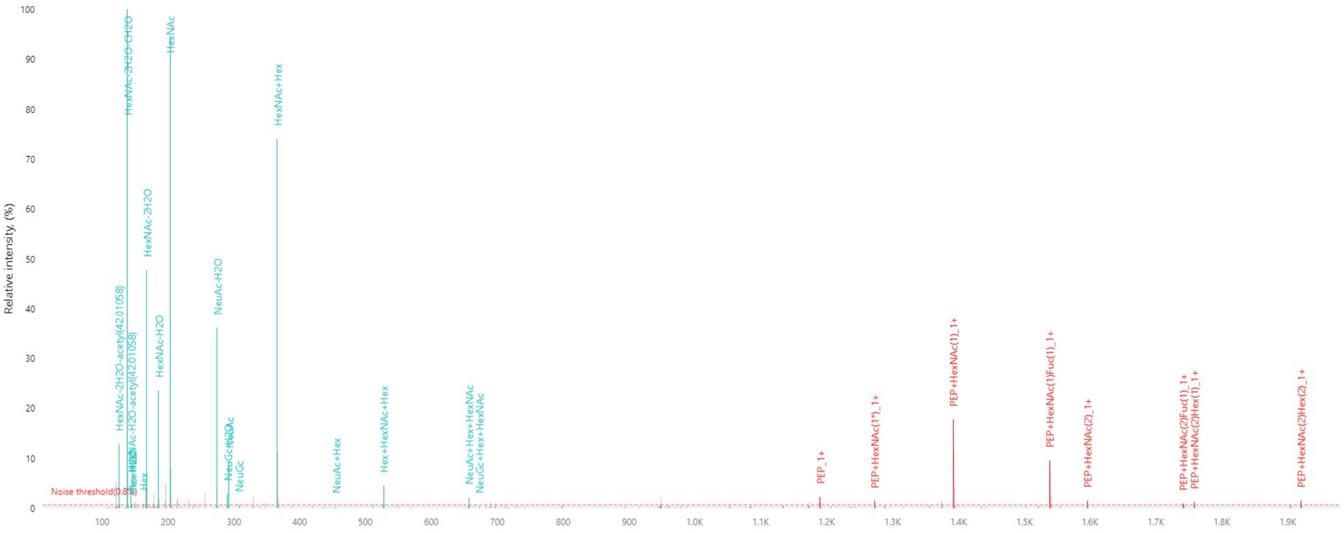

EEQYNSTYR(=PEP)_5_4_1_1_0, m/z:1083.7549(3+), RT:31.90, HCD-score:96.14, Y-score:97.05, P-score:22.22,
CID-MS/MS Scan:7159, SNR=0.8, Base Peak Intensity=719028.9

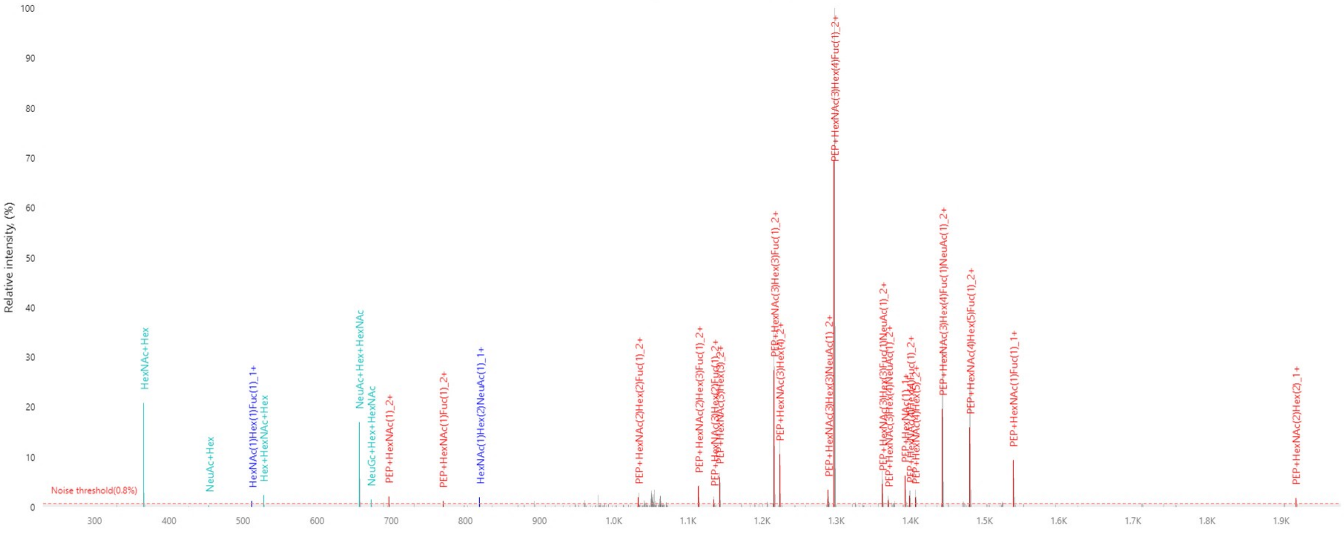

EEQYNSTYR(=PEP)_5_5_1_0_0, m/z:1054.4164(3+), RT:23.39, HCD-score:95.98, Y-score:98.79, P-score:33.33,
HCD-MS/MS Scan:4432, SNR=0.8, Base Peak Intensity=1589516.5

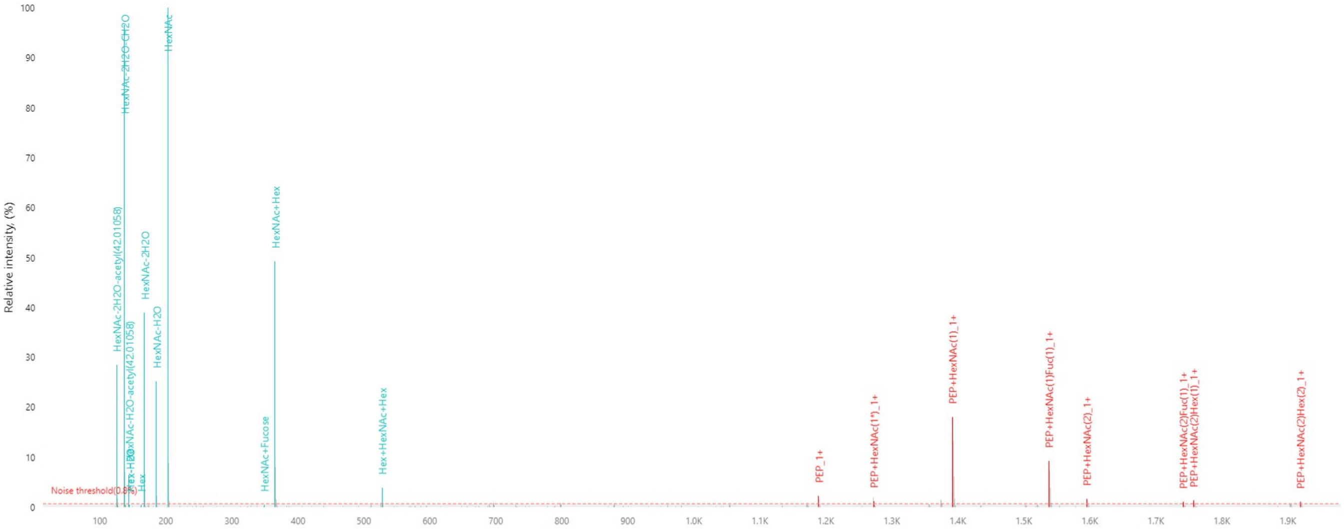

EEQYNSTYR(=PEP)_5_5_1_0_0, m/z:1054.4164(3+), RT:23.40, HCD-score:95.98, Y-score:98.79, P-score:33.33,
CID-MS/MS Scan:4435, SNR=0.8, Base Peak Intensity=953563.4

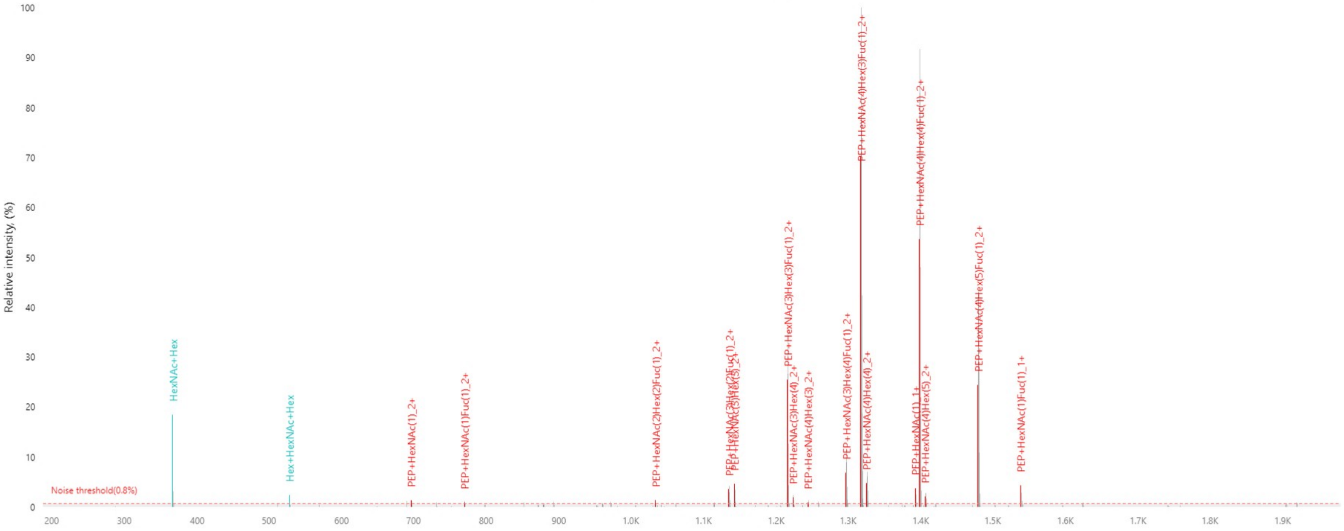

EEQYNSTYR(=PEP)_5_5_1_0_1, m/z:1156.7793(3+), RT:31.24, HCD-score:96.76, Y-score:97.90, P-score:11.11,
HCD-MS/MS Scan:6954, SNR=0.8, Base Peak Intensity=772883.2

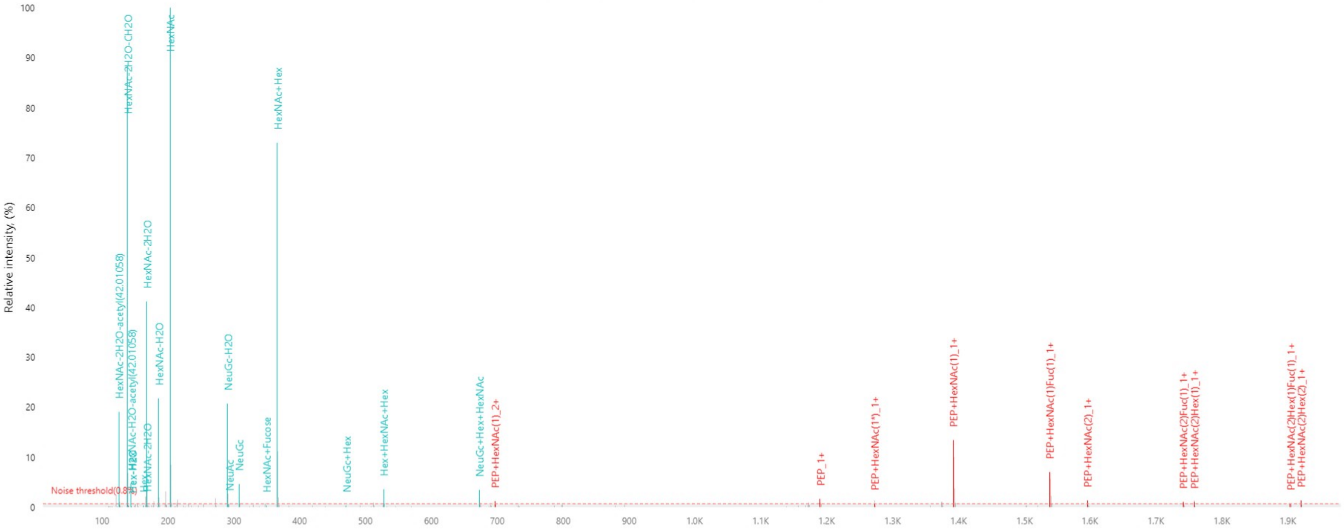

EEQYNSTYR(=PEP)_5_5_1_0_1, m/z:1156.7793(3+), RT:31.24, HCD-score:96.76, Y-score:97.90, P-score:11.11,
CID-MS/MS Scan:6956, SNR=0.8, Base Peak Intensity=288078.2

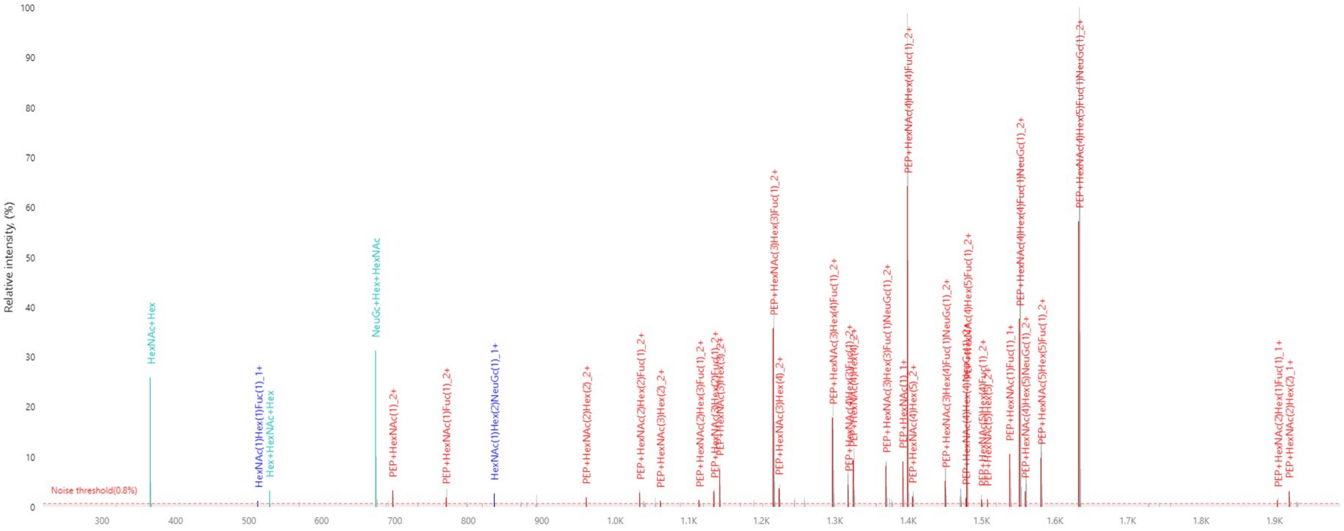

EEQYNSTYR(=PEP)_6_2_0_0_0, m/z:856.6714(3+), RT:22.01, HCD-score:86.62, Y-score:93.40, P-score:11.11,
HCD-MS/MS Scan:4108, SNR=0.8, Base Peak Intensity=1209472.5

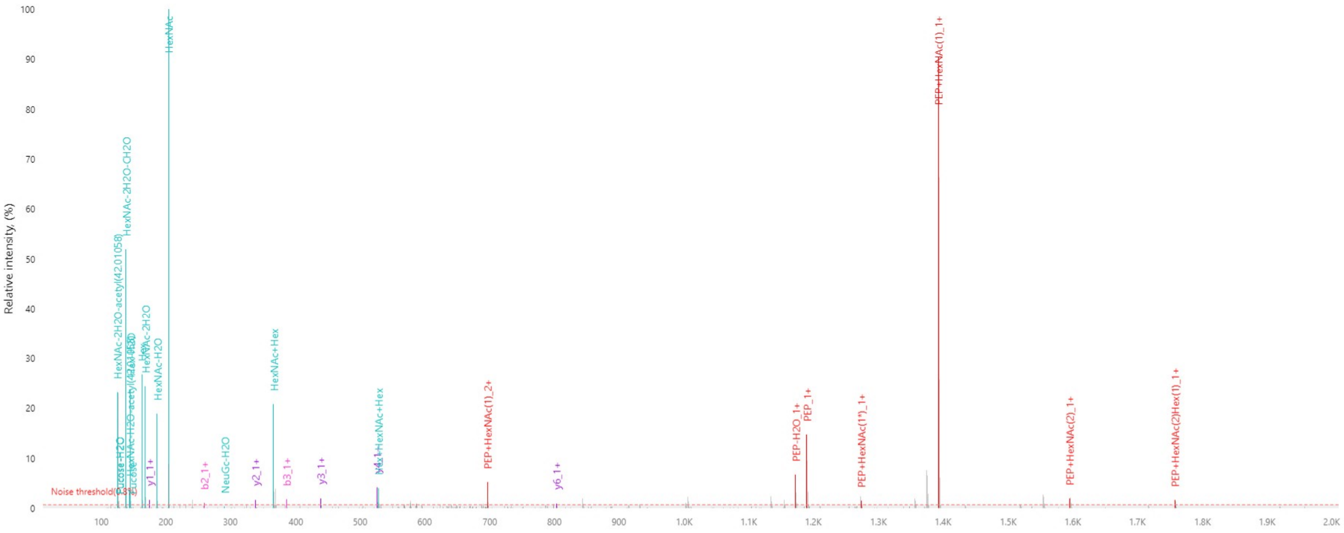

EEQYNSTYR(=PEP)_6_2_0_0_0, m/z:856.6714(3+), RT:22.03, HCD-score:86.62, Y-score:93.40, P-score:11.11,
CID-MS/MS Scan:4111, SNR=0.8, Base Peak Intensity=1113987.6

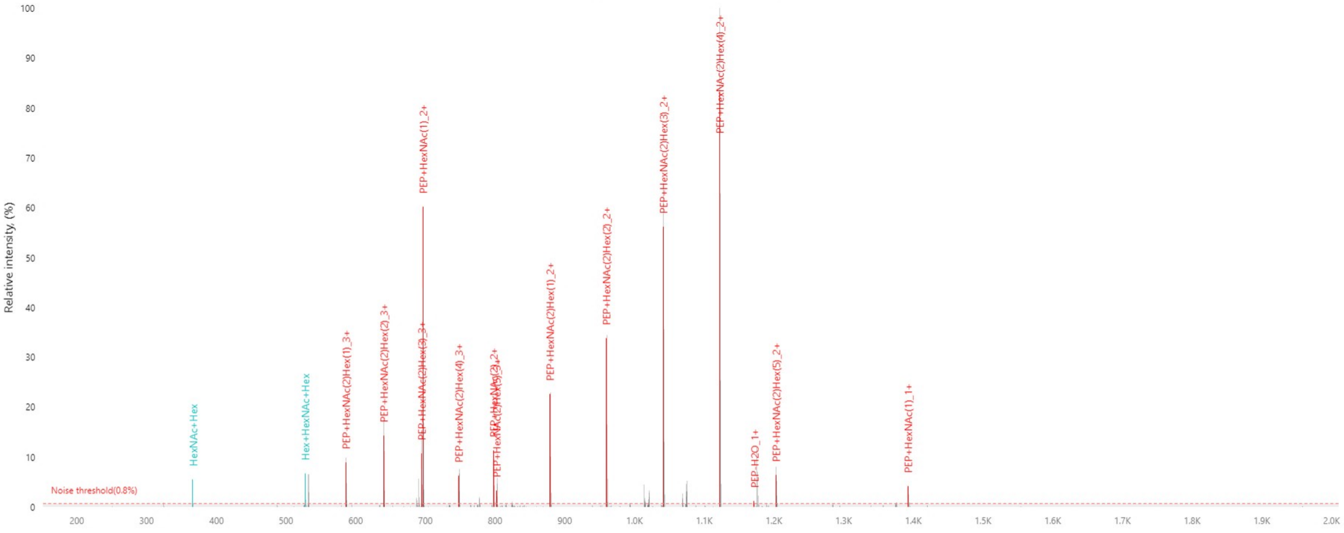

EEQYNSTYR(=PEP)_6_3_0_0_0, m/z:924.3655(3+), RT:22.35, HCD-score:89.21, Y-score:94.03, P-score:0.00,
HCD-MS/MS Scan:4180, SNR=0.8, Base Peak Intensity=4049626.2

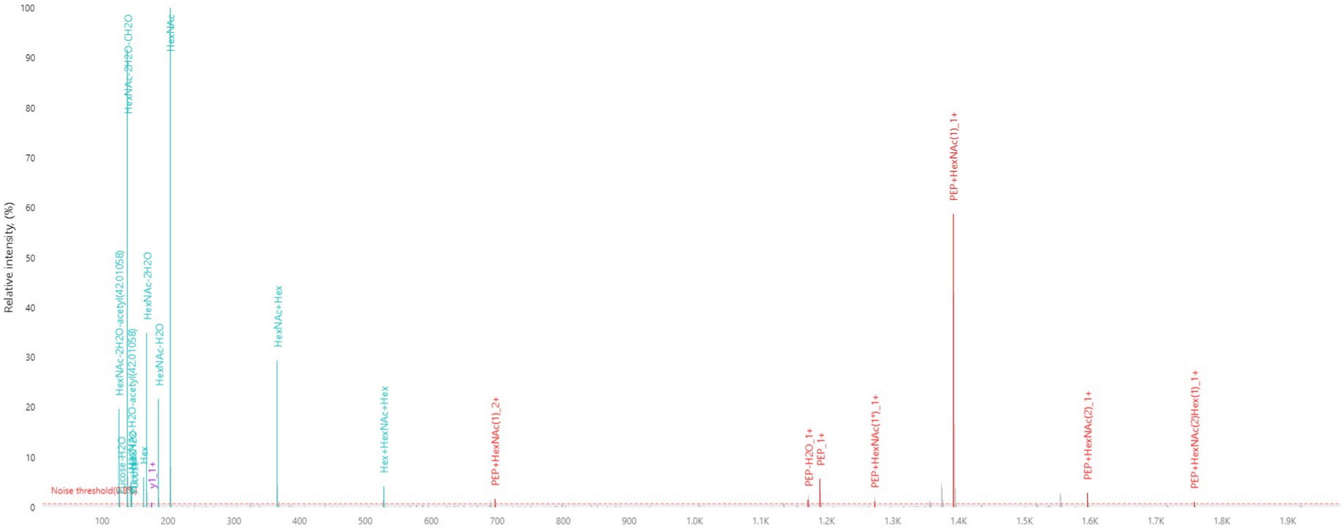

EEQYNSTYR(=PEP)_6_3_0_0_0, m/z:924.3655(3+), RT:22.36, HCD-score:89.21, Y-score:94.03, P-score:0.00,
CID-MS/MS Scan:4183, SNR=0.8, Base Peak Intensity=7324936

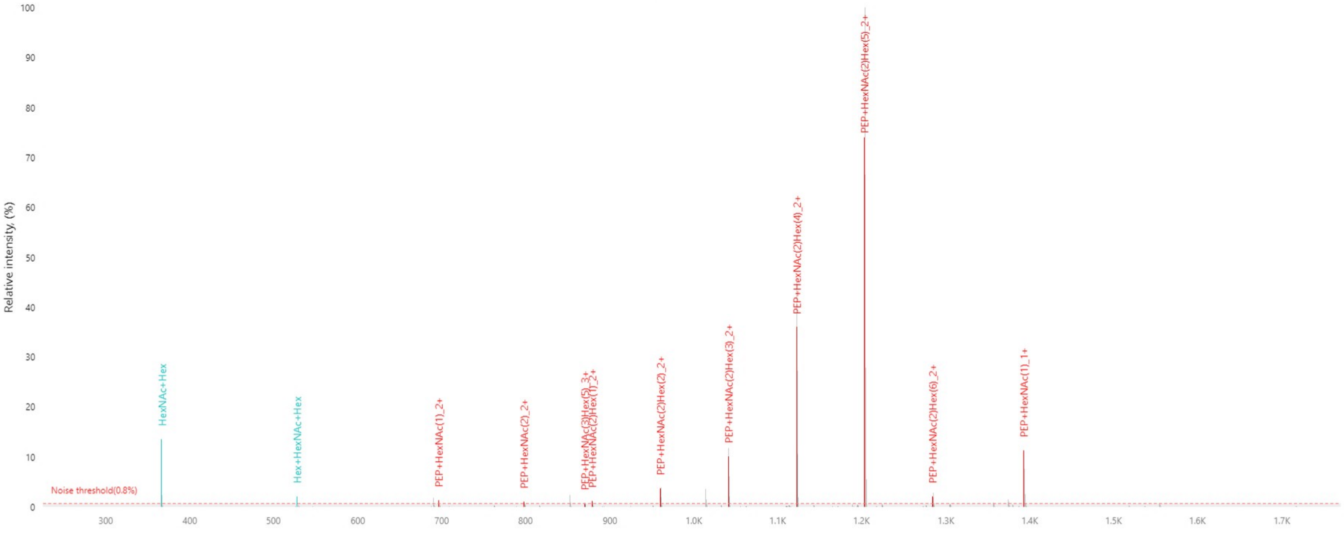

EEQYNSTYR(=PEP)_6_3_0_0_1, m/z:770.2958(4+), RT:31.04, HCD-score:82.64, Y-score:93.54, P-score:11.11, HCD-MS/MS Scan:6655, SNR=0.8, Base Peak Intensity=696881.6

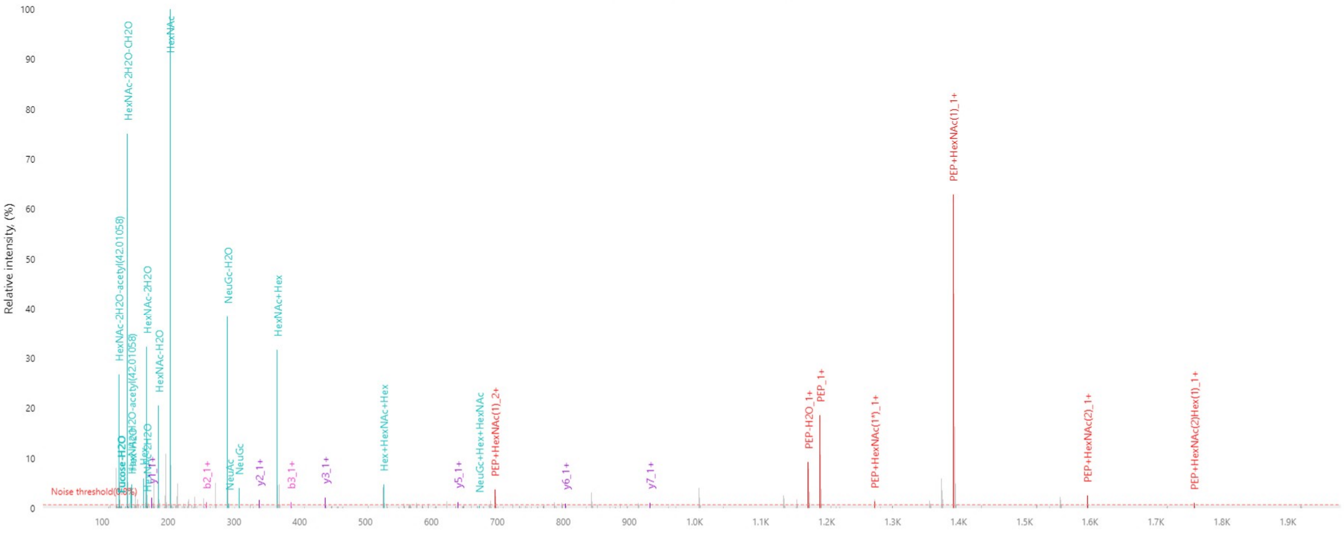

EEQYNSTYR(=PEP)_6_3_0_0_1, m/z:770.2958(4+), RT:31.05, HCD-score:82.64, Y-score:93.54, P-score:11.11, CID-MS/MS Scan:6658, SNR=0.8, Base Peak Intensity=431944.3

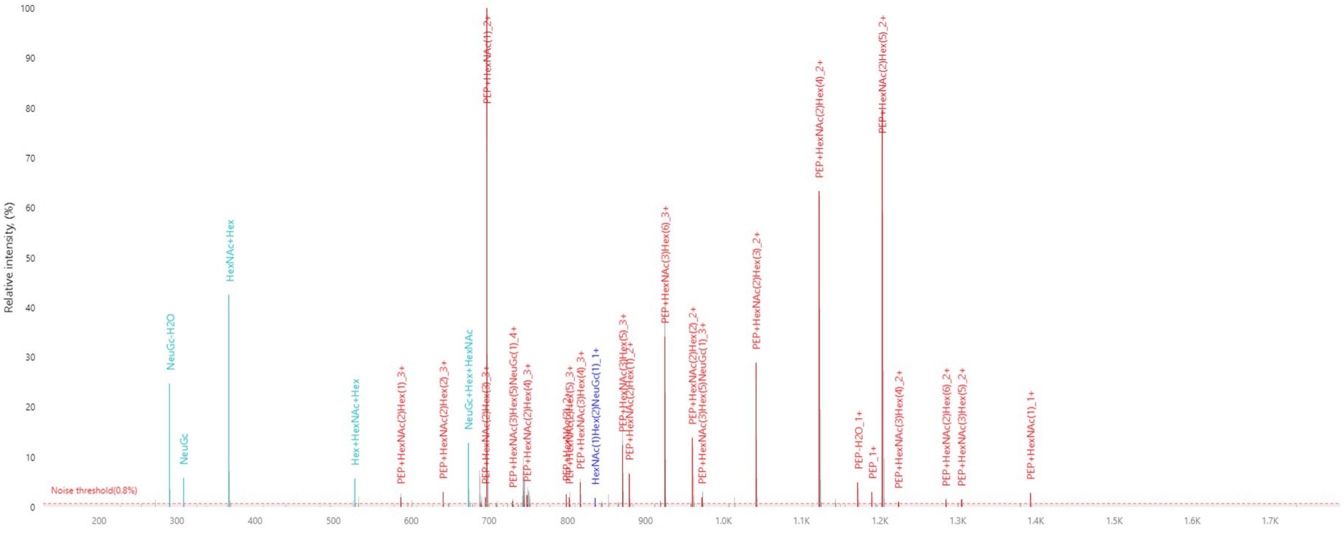

EEQYNSTYR(=PEP)_6_3_1_0_0, m/z:973.0523(3+), RT:22.30, HCD-score:90.56, Y-score:95.81, P-score:44.44,
HCD-MS/MS Scan:4166, SNR=0.8, Base Peak Intensity=5566105.5

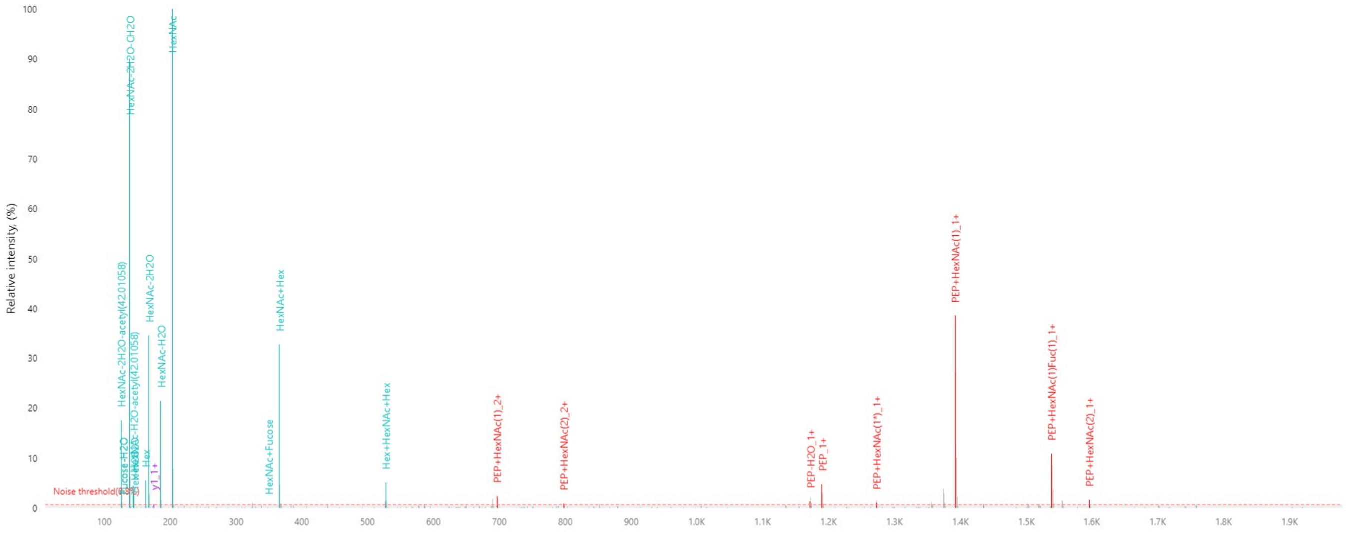

EEQYNSTYR(=PEP)_6_3_1_0_0, m/z:973.0523(3+), RT:22.30, HCD-score:90.56, Y-score:95.81, P-score:44.44,
CID-MS/MS Scan:4168, SNR=0.8, Base Peak Intensity=7279610.5

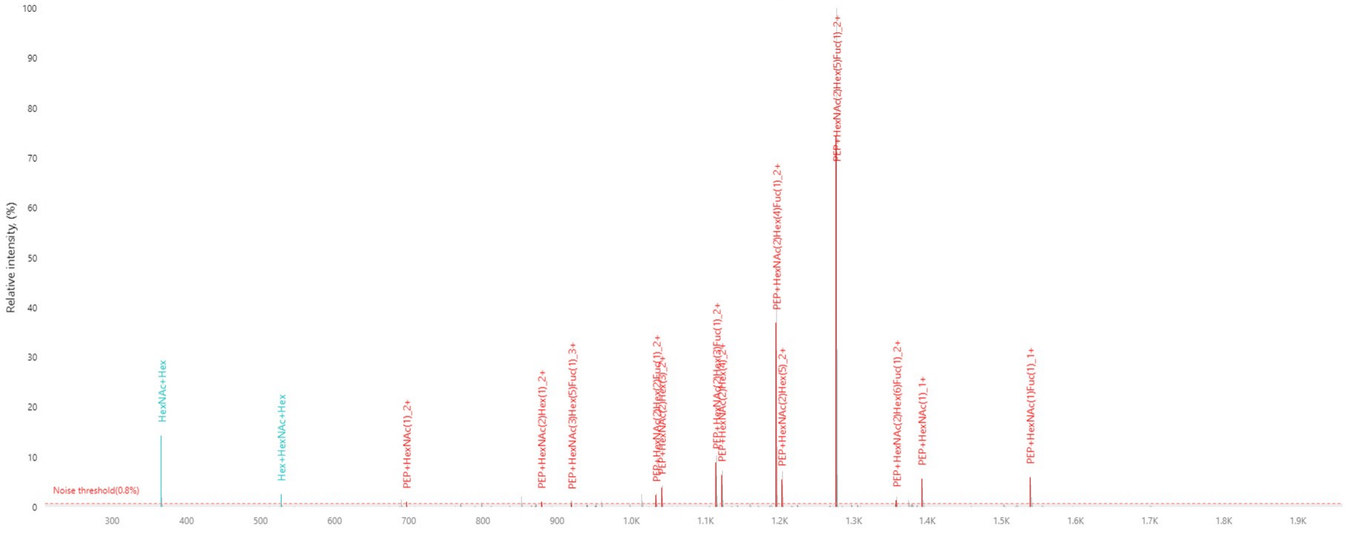

EEQYNSTYR(=PEP)_6_3_1_0_1, m/z:806.8114(4+), RT:30.92, HCD-score:90.81, Y-score:95.55, P-score:44.44,
HCD-MS/MS Scan:6858, SNR=0.8, Base Peak Intensity=945111.1

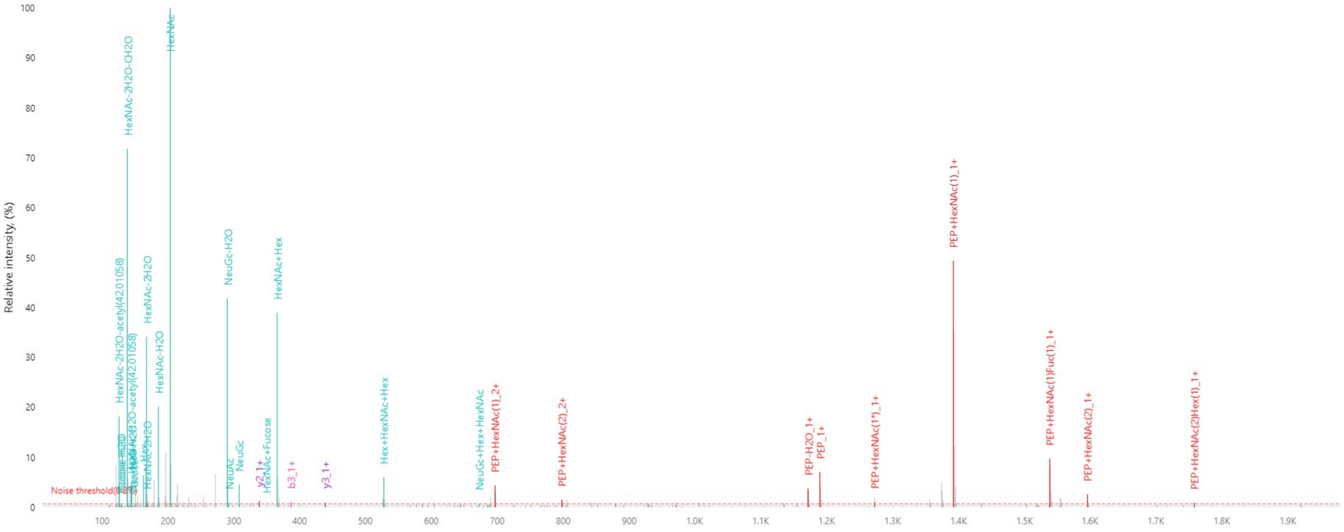

EEQYNSTYR(=PEP)_6_3_1_0_1, m/z:806.8114(4+), RT:30.92, HCD-score:90.81, Y-score:95.55, P-score:44.44,
CID-MS/MS Scan:6860, SNR=0.8, Base Peak Intensity=365473.4

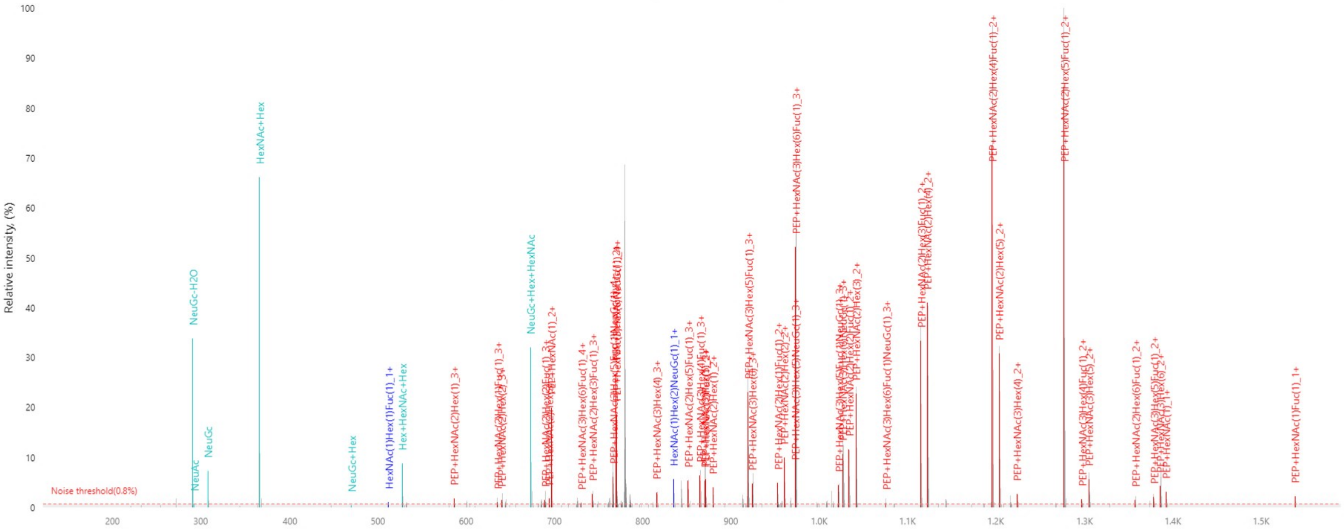

EEQYNSTYR(=PEP)_6_4_1_0_0, m/z:1040.7422(3+), RT:21.85, HCD-score:94.85, Y-score:93.66, P-score:0.00,
HCD-MS/MS Scan:4064, SNR=0.8, Base Peak Intensity=579279.9

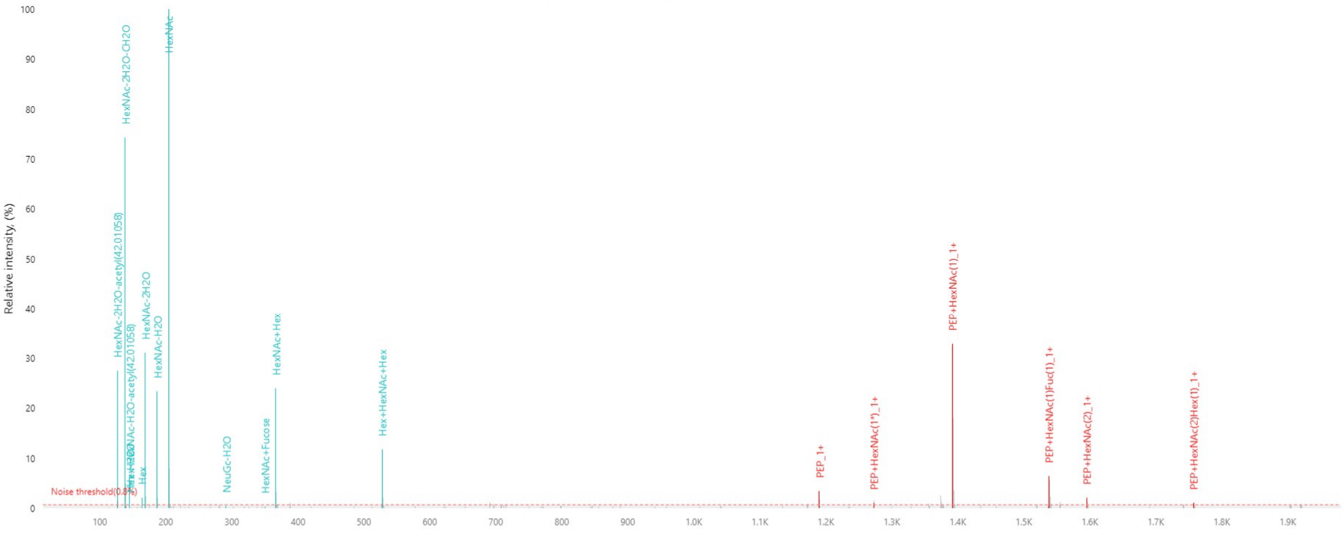

EEQYNSTYR(=PEP)_6_4_1_0_0, m/z:1040.7422(3+), RT:21.85, HCD-score:94.85, Y-score:93.66, P-score:0.00,
CID-MS/MS Scan:4066, SNR=0.8, Base Peak Intensity=286111.4

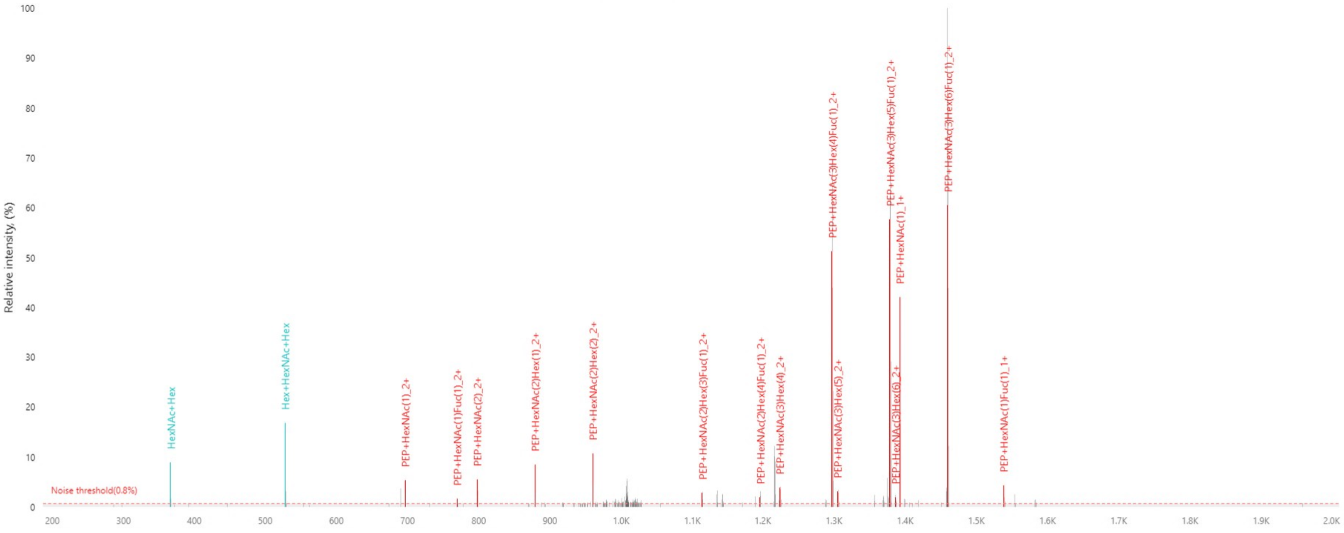

EEQYNSTYR(=PEP)_6_4_1_0_1, m/z:857.5818(4+), RT:30.83, HCD-score:95.47, Y-score:89.61, P-score:77.78,
HCD-MS/MS Scan:6724, SNR=0.8, Base Peak Intensity=3558771.2

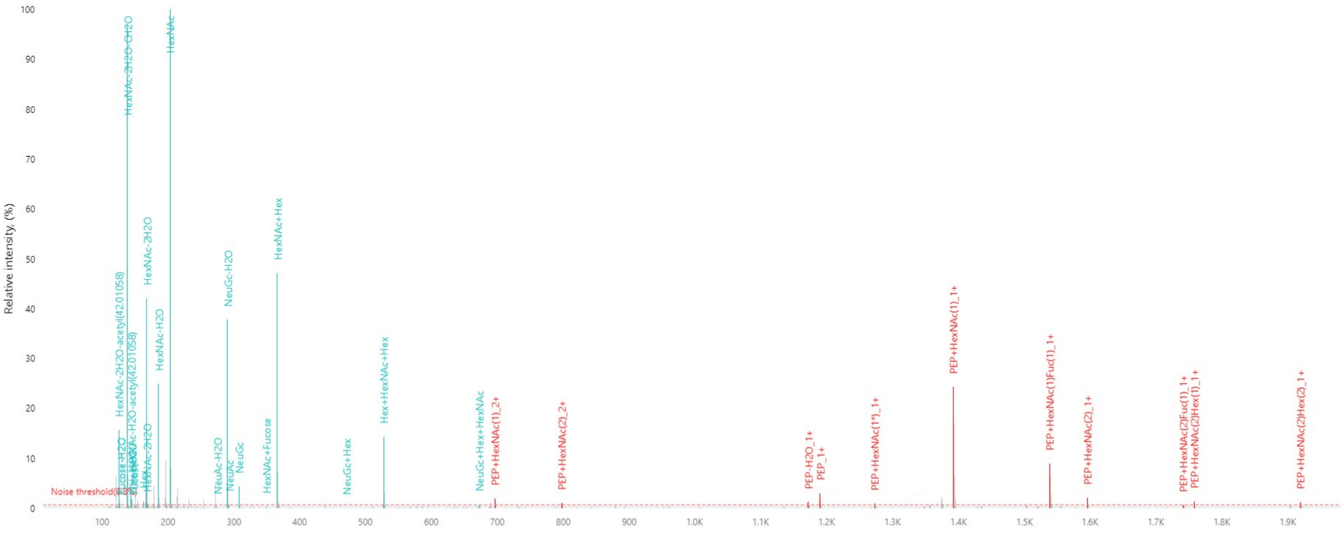

EEQYNSTYR(=PEP)_6_4_1_0_1, m/z:857.5818(4+), RT:30.83, HCD-score:95.47, Y-score:89.61, P-score:77.78,
CID-MS/MS Scan:6726, SNR=0.8, Base Peak Intensity=1592878.6

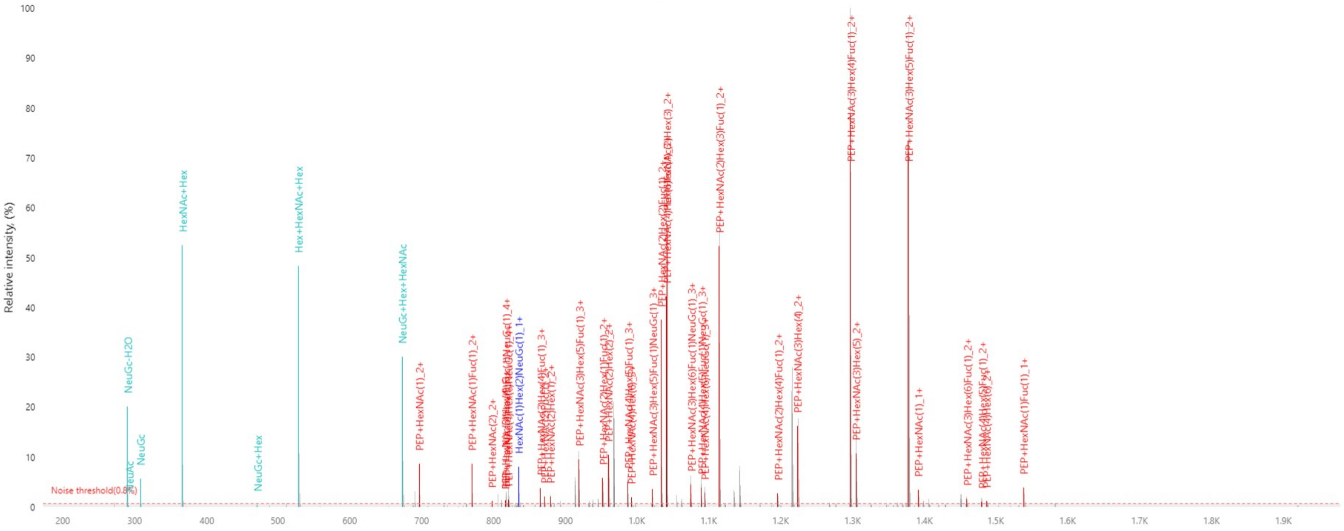

EEQYNSTYR(=PEP)_6_5_1_0_1, m/z:1210.7966(3+), RT:31.01, HCD-score:97.39, Y-score:98.31, P-score:22.22,
HCD-MS/MS Scan:6679, SNR=0.8, Base Peak Intensity=1479856.8

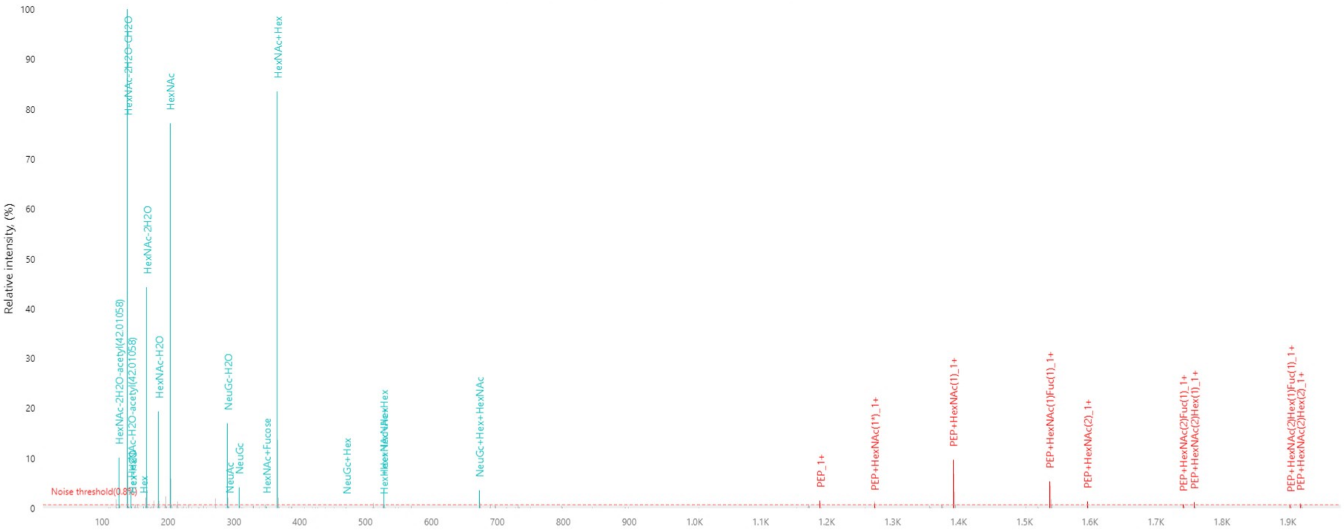

EEQYNSTYR(=PEP)_6_5_1_0_1, m/z:1210.7966(3+), RT:31.02, HCD-score:97.39, Y-score:98.31, P-score:22.22,
CID-MS/MS Scan:6682, SNR=0.8, Base Peak Intensity=751758.5

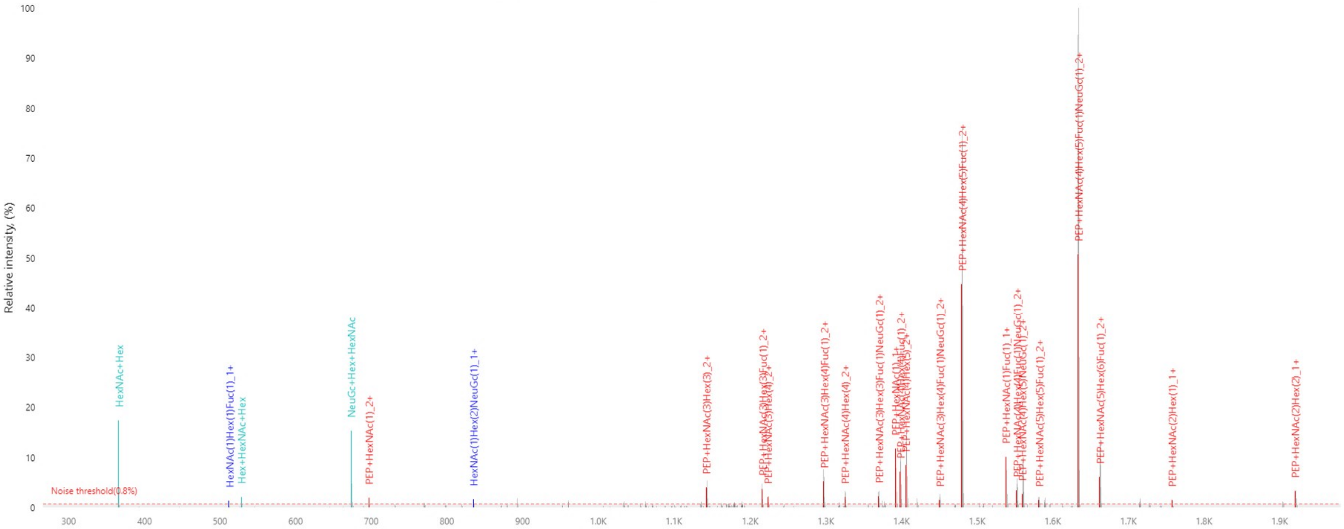

EEQYNSTYR(=PEP)_6_5_1_0_2, m/z:1313.1582(3+), RT:37.52, HCD-score:57.51, Y-score:80.94, P-score:0.00,
HCD-MS/MS Scan:9031, SNR=0.8, Base Peak Intensity=402950.5

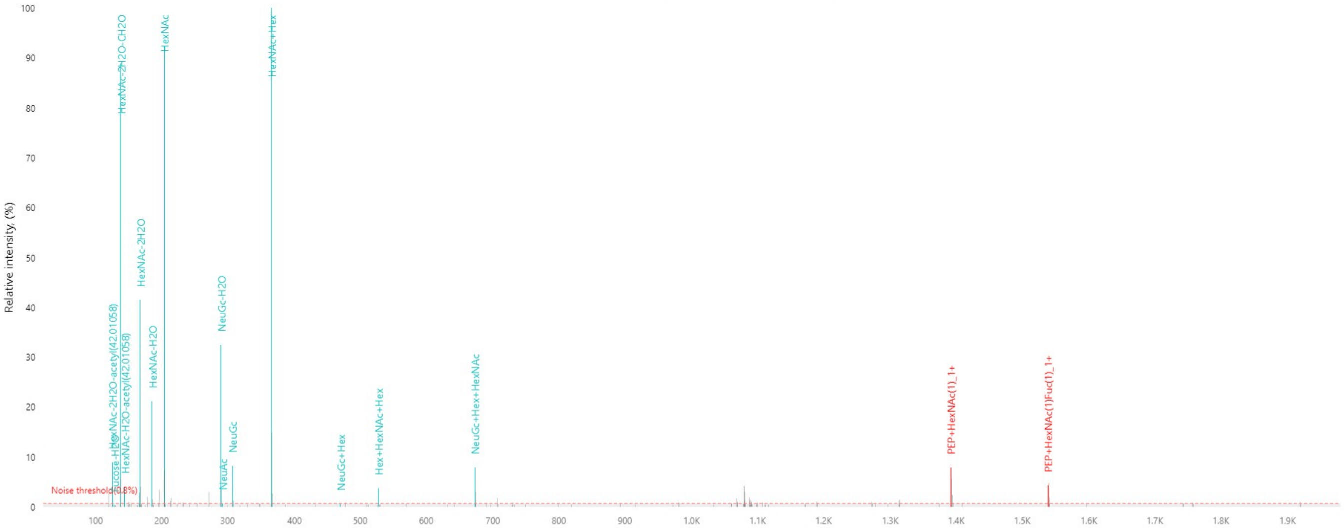

EEQYNSTYR(=PEP)_6_5_1_0_2, m/z:1313.1582(3+), RT:37.52, HCD-score:57.51, Y-score:80.94, P-score:0.00,
CID-MS/MS Scan:9033, SNR=0.8, Base Peak Intensity=243288.7

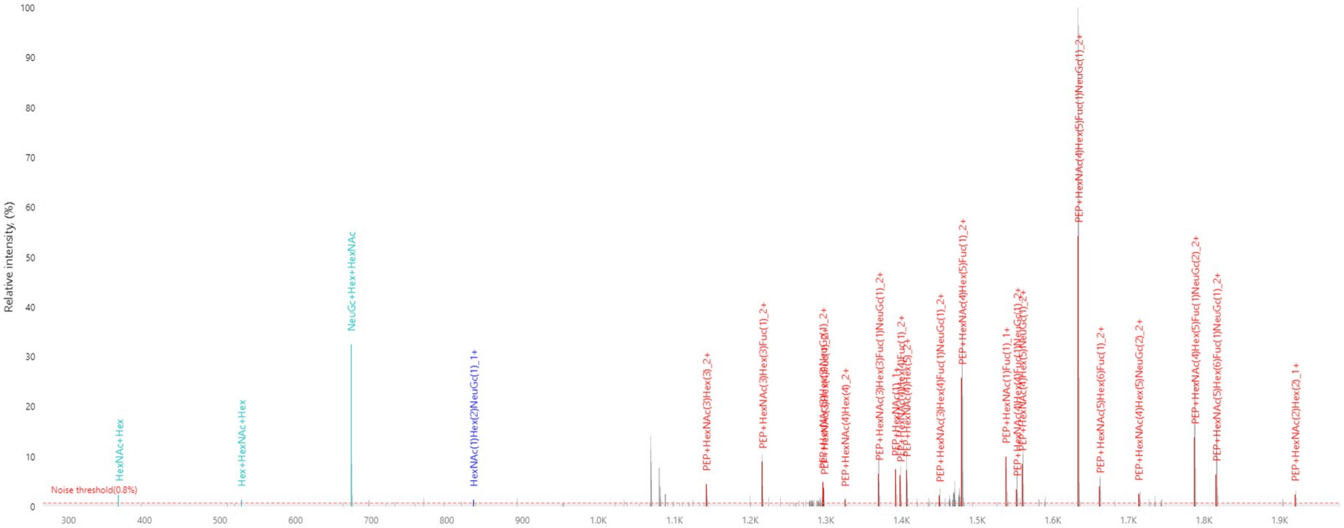

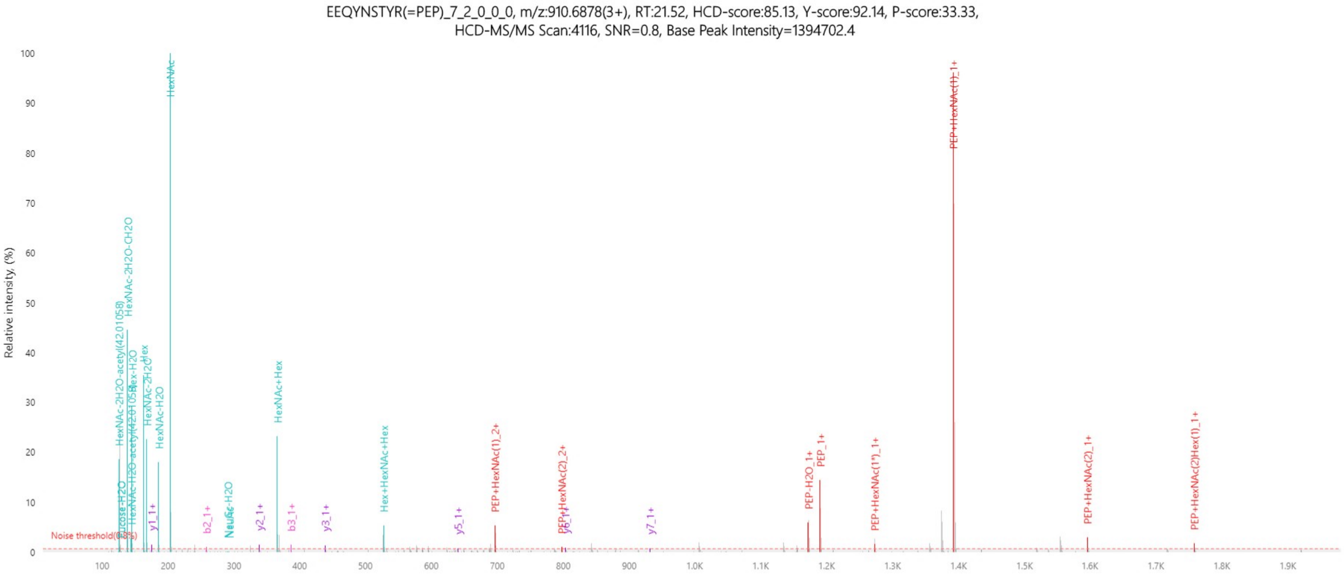
EEQYNSTYR(=PEP)_7_2_0_0_0, m/z:910.6878(3+), RT:21.52, HCD-score:85.13, Y-score:92.14, P-score:33.33,
HCD-MS/MS Scan:4116, SNR=0.8, Base Peak Intensity=1394702.4
Relative intensity, (%)
HexNAc
PEP+HexNAc(1)_1+
HexNAc+Hex
Hex+HexNAc+Hex
PEP+HexNAc(1)_2+
PEP+HexNAc(2)_2+
PEP-H2O_1+
PEP_1+
PEP+HexNAc(1*)_1+
PEP+HexNAc(2)_1+
PEP+HexNAc(2)Hex(1)_1+
Noise threshold(0.8%)

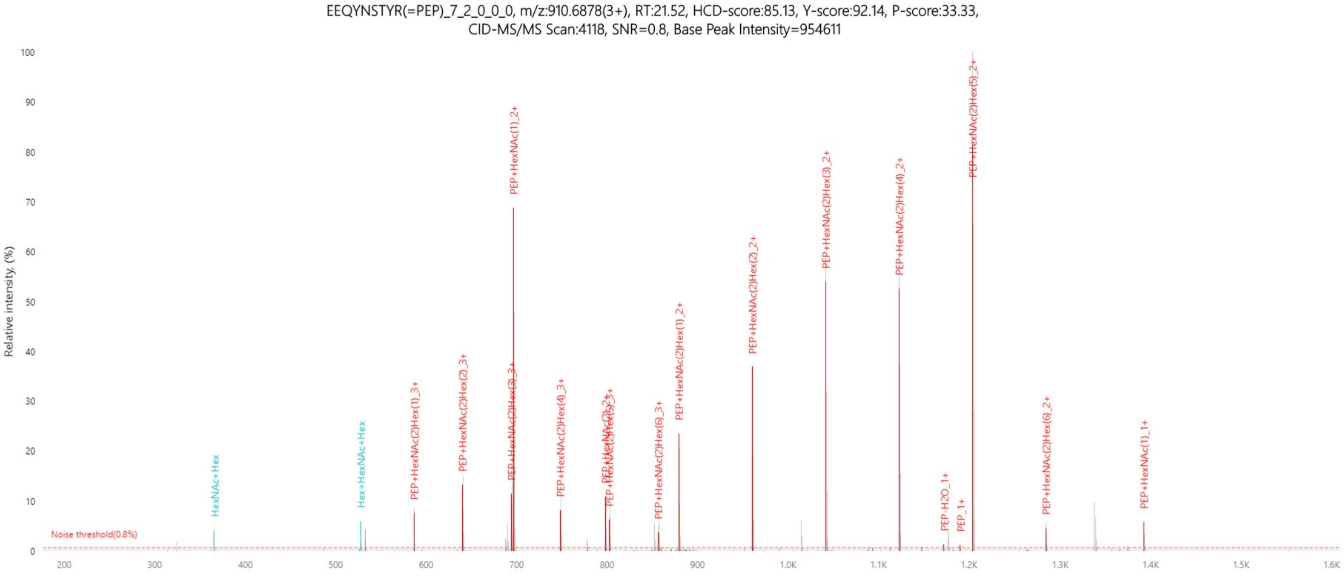
EEQYNSTYR(=PEP)_7_2_0_0_0, m/z:910.6878(3+), RT:21.52, HCD-score:85.13, Y-score:92.14, P-score:33.33,
CID-MS/MS Scan:4118, SNR=0.8, Base Peak Intensity=954611
Relative intensity, (%)
HexNAc+Hex
Hex+HexNAc+Hex
PEP+HexNAc(2)Hex(1)_3+
PEP+HexNAc(2)Hex(2)_3+
PEP+HexNAc(1)_2+
PEP+HexNAc(2)Hex(4)_3+
PEP+HexNAc(2)Hex(6)_3+
PEP+HexNAc(2)Hex(1)_2+
PEP+HexNAc(2)Hex(2)_2+
PEP+HexNAc(2)Hex(3)_2+
PEP+HexNAc(2)Hex(4)_2+
PEP+HexNAc(2)Hex(5)_2+
PEP-H2O_1+
PEP_1+
PEP+HexNAc(2)Hex(6)_2+
PEP+HexNAc(1)_1+
Noise threshold(0.8%)

EEQYNSTYR(=PEP)_7_4_1_0_0, m/z:1094.7588(3+), RT:22.37, HCD-score:96.66, Y-score:95.38, P-score:44.44,
HCD-MS/MS Scan:4220, SNR=0.8, Base Peak Intensity=1741562.8

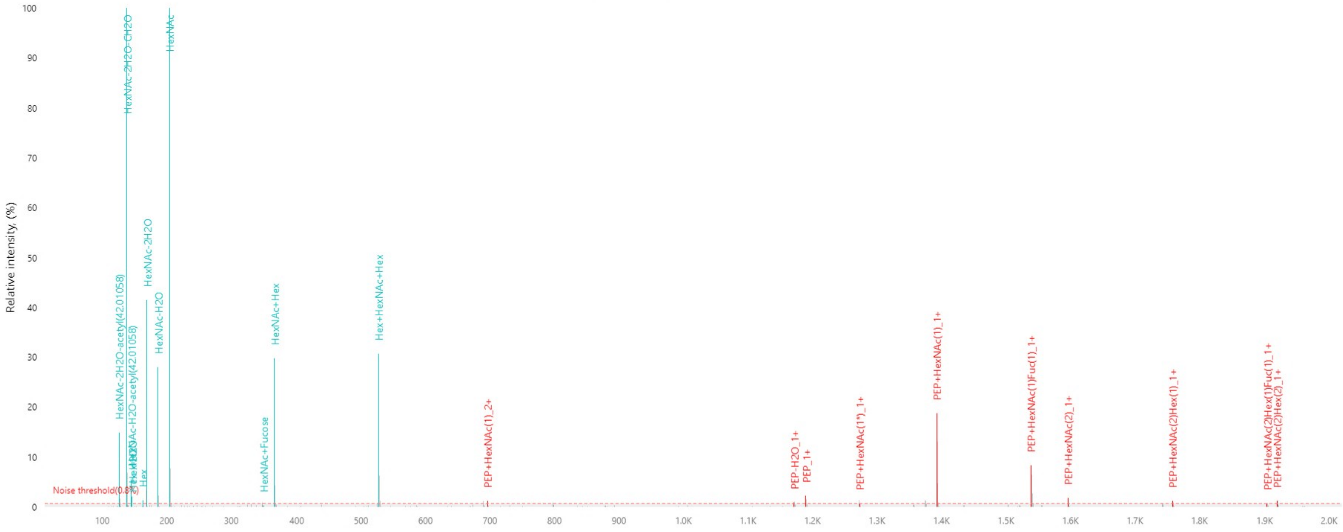

EEQYNSTYR(=PEP)_7_4_1_0_0, m/z:1094.7588(3+), RT:22.38, HCD-score:96.66, Y-score:95.38, P-score:44.44,
CID-MS/MS Scan:4223, SNR=0.8, Base Peak Intensity=1946275

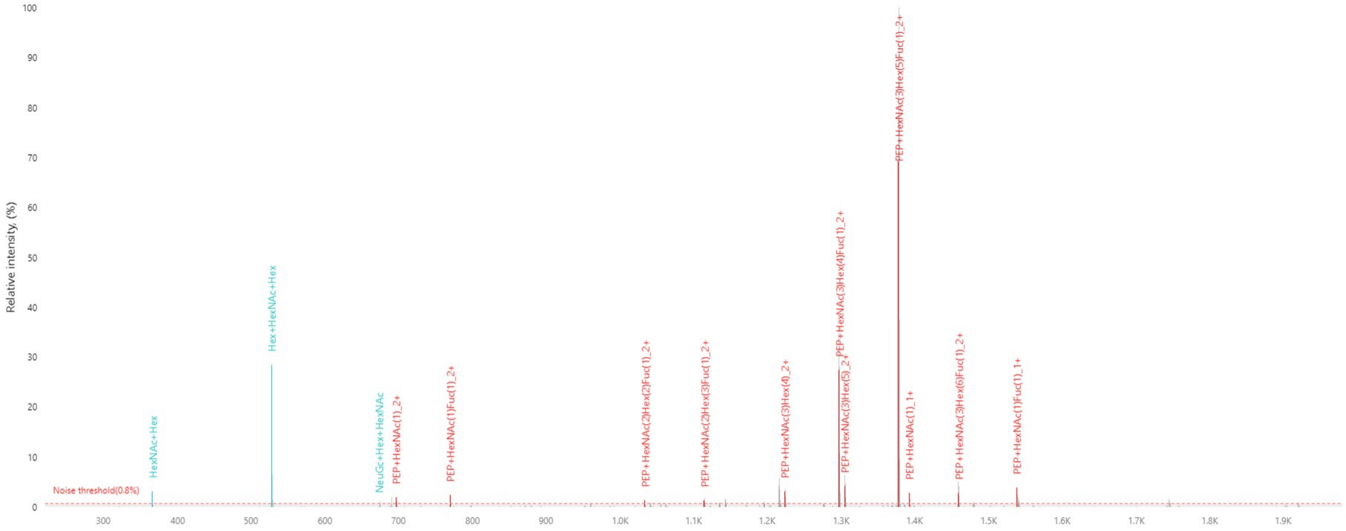

EEQYNSTYR(=PEP)_7_5_1_0_1, m/z:1264.8151(3+), RT:30.20, HCD-score:100.00, Y-score:95.44, P-score:0.00,
HCD-MS/MS Scan:6413, SNR=0.8, Base Peak Intensity=253338.3

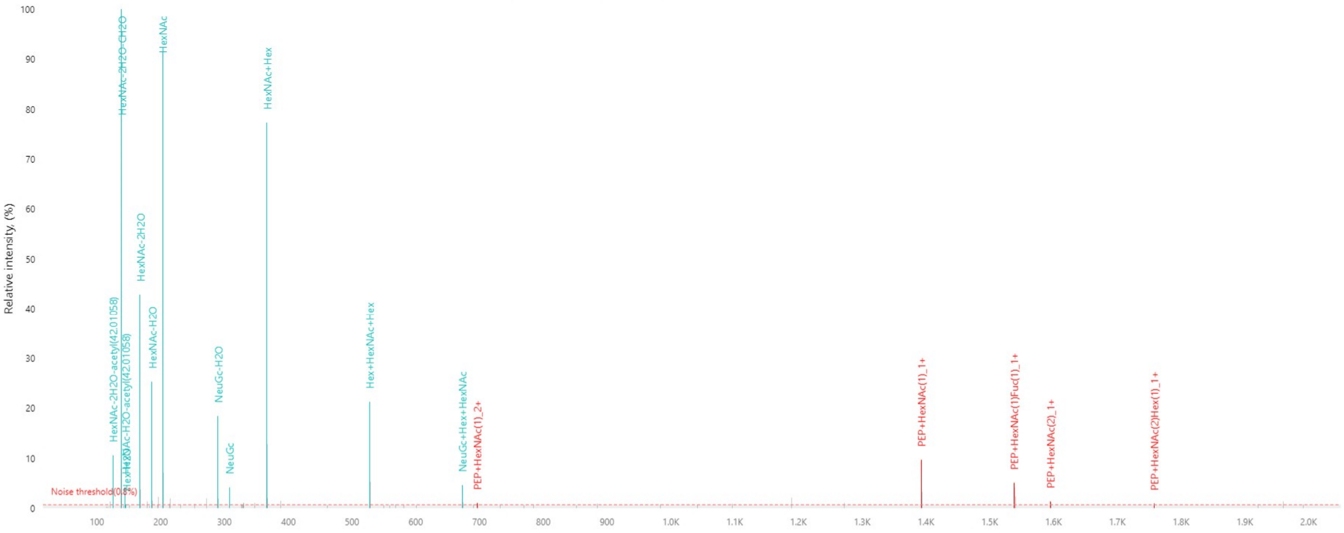

EEQYNSTYR(=PEP)_7_5_1_0_1, m/z:1264.8151(3+), RT:30.22, HCD-score:100.00, Y-score:95.44, P-score:0.00,
CID-MS/MS Scan:6416, SNR=0.8, Base Peak Intensity=103403.8

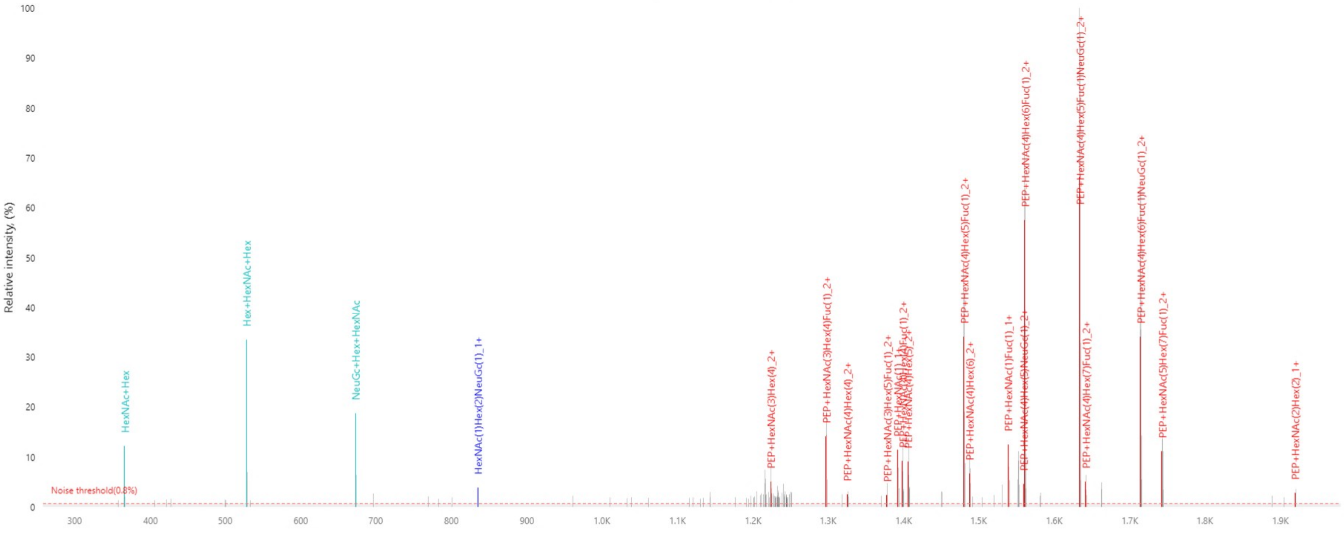

EEQYNSTYR(=PEP)_8_2_0_0_0, m/z:964.7035(3+), RT:21.12, HCD-score:88.04, Y-score:92.77, P-score:11.11,
HCD-MS/MS Scan:3814, SNR=0.8, Base Peak Intensity=570309.6

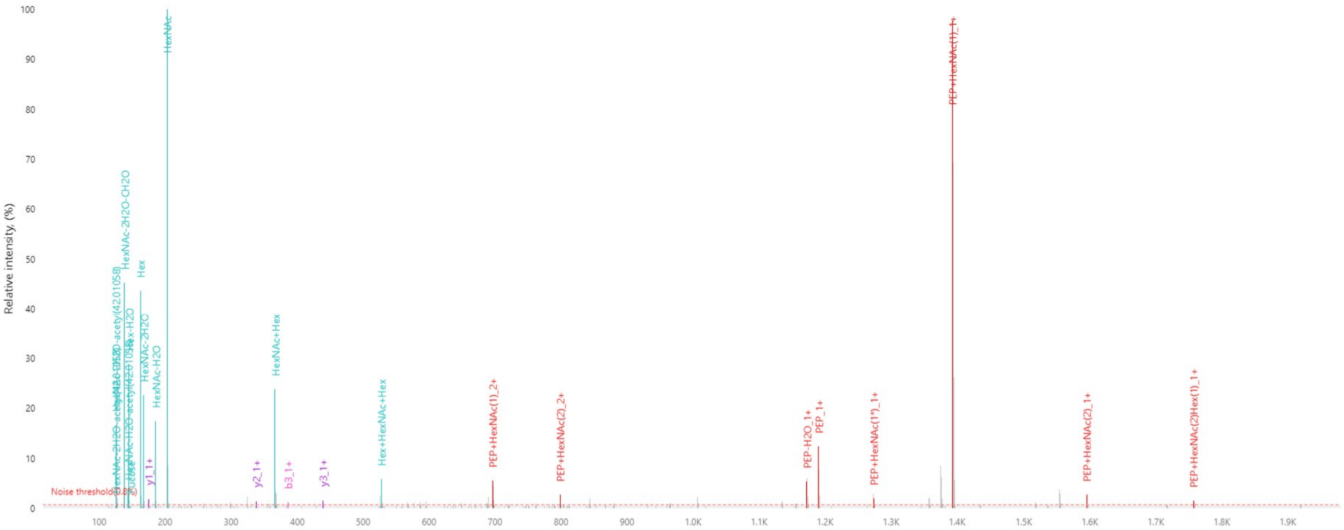

EEQYNSTYR(=PEP)_8_2_0_0_0, m/z:964.7035(3+), RT:21.13, HCD-score:88.04, Y-score:92.77, P-score:11.11,
CID-MS/MS Scan:3816, SNR=0.8, Base Peak Intensity=477653.1

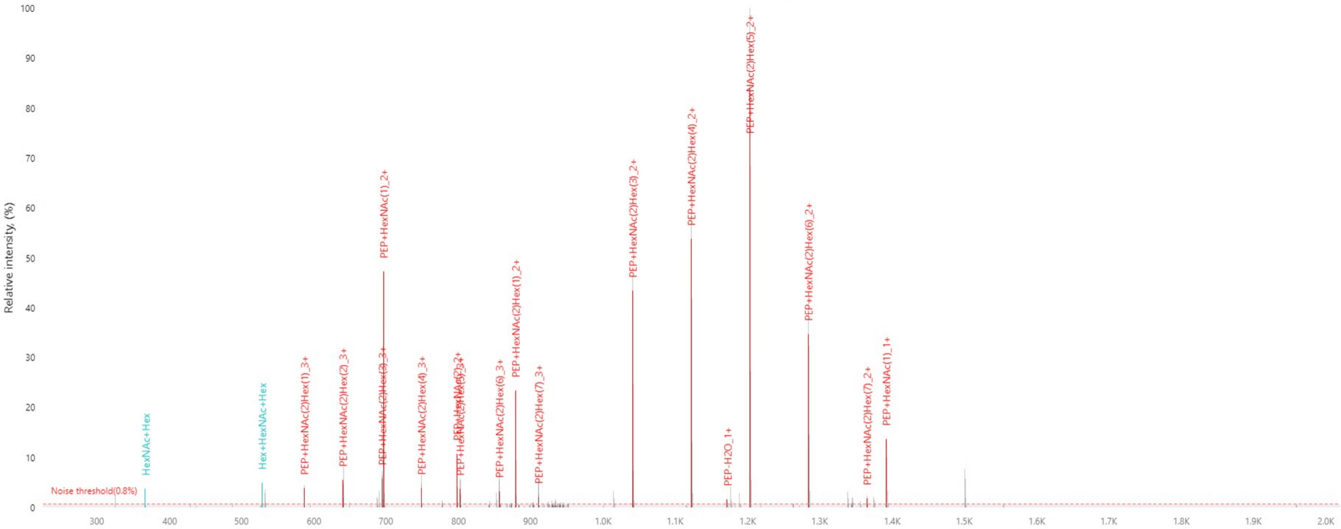

EEQYNSTYR(=PEP)_9_2_0_0_0, m/z:1018.7227(3+), RT:21.15, HCD-score:87.26, Y-score:92.46, P-score:11.11,
HCD-MS/MS Scan:3874, SNR=0.8, Base Peak Intensity=406569.4

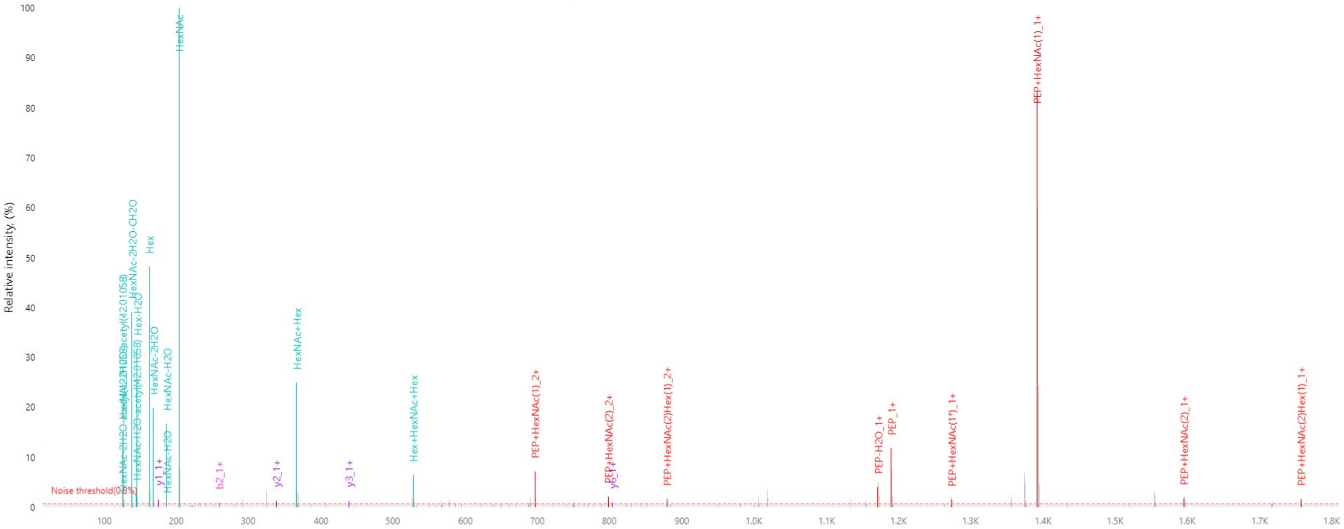

EEQYNSTYR(=PEP)_9_2_0_0_0, m/z:1018.7227(3+), RT:21.16, HCD-score:87.26, Y-score:92.46, P-score:11.11,
CID-MS/MS Scan:3877, SNR=0.8, Base Peak Intensity=263529.2

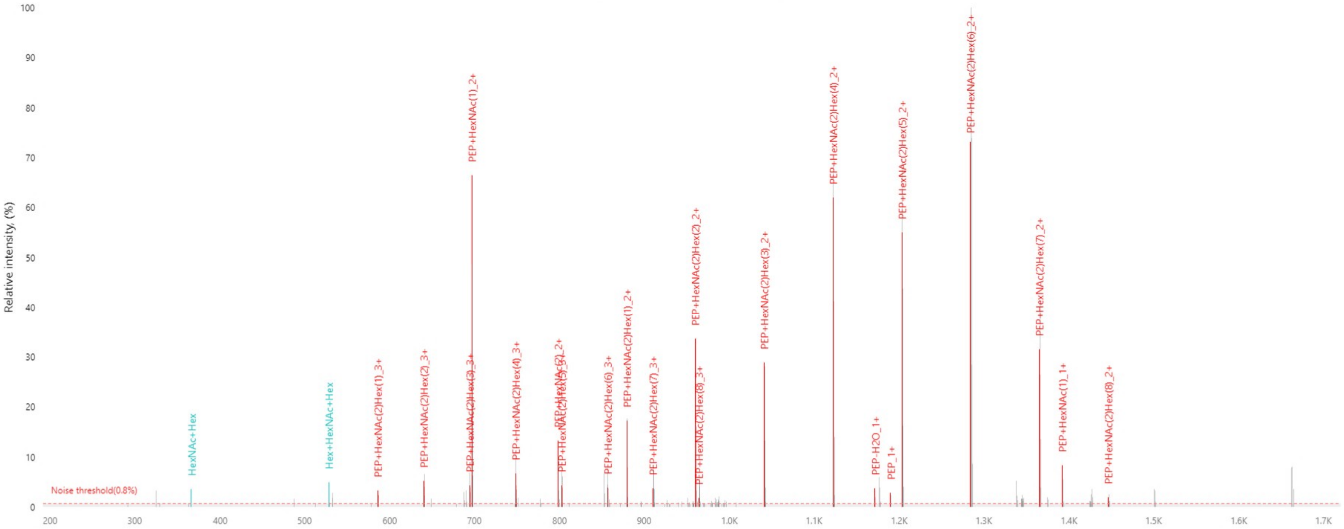

THTCPPCPAPELLGGPSVFLFPPKPK(=PEP)_1_1_0_0_1, m/z:879.9266(4+), RT:66.93, HCD-score:71.38, Y-score:72.21, P-score:,
HCD-MS/MS Scan:18457, SNR=0.8, Base Peak Intensity=14617.4

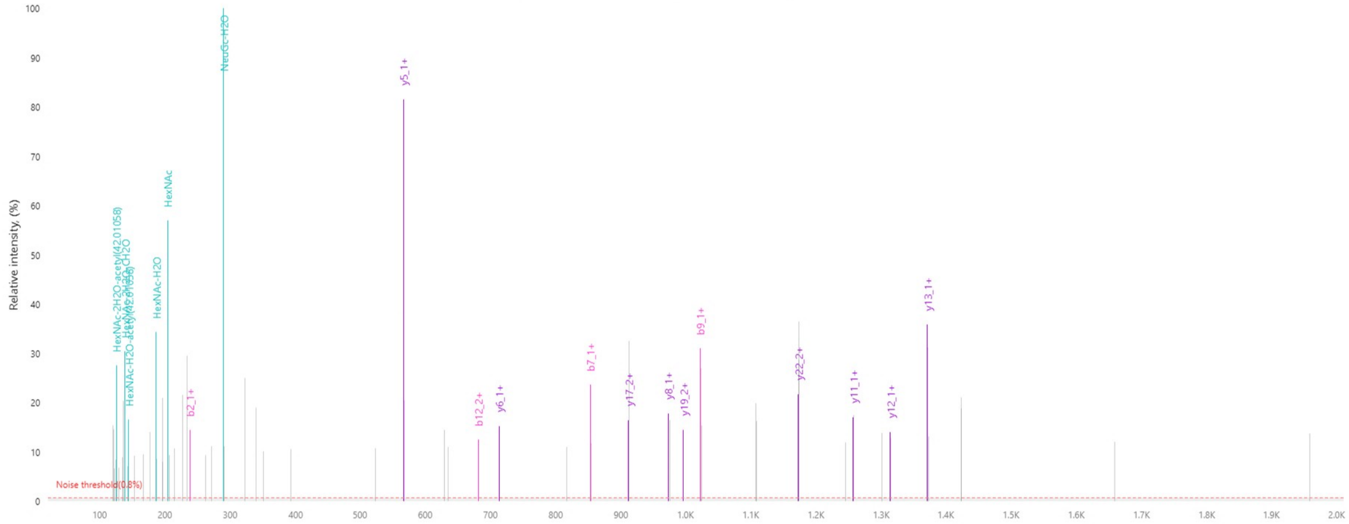

THTCPPCPAPELLGGPSVFLFPPKPK(=PEP)_1_1_0_0_1, m/z:879.9266(4+), RT:66.93, HCD-score:71.38, Y-score:72.21, P-score:,
CID-MS/MS Scan:18459, SNR=0.8, Base Peak Intensity=44041.8

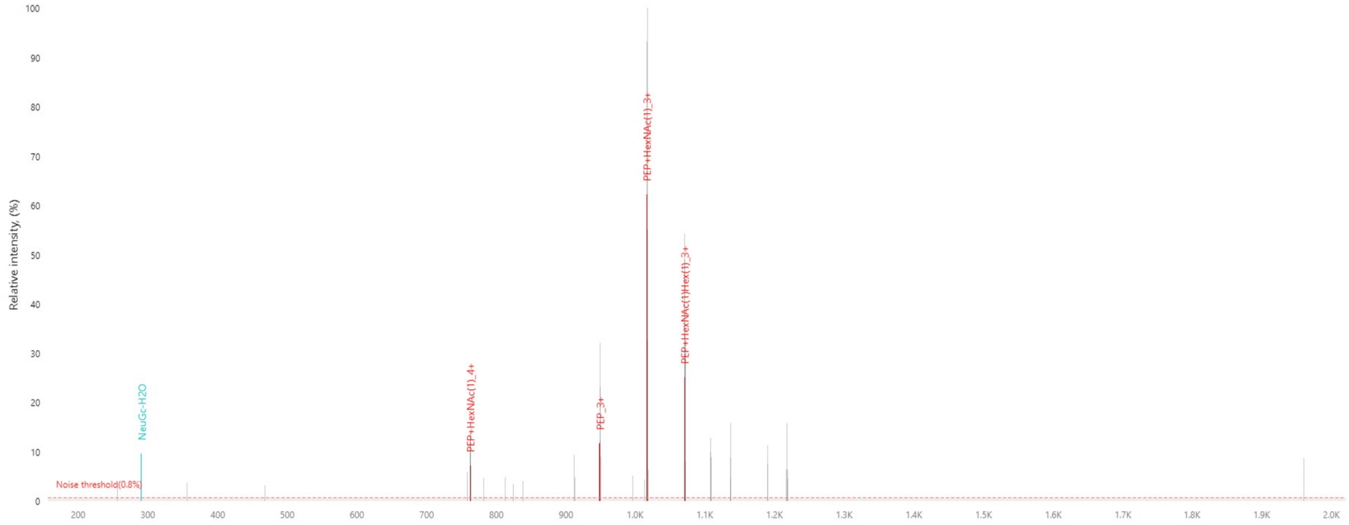

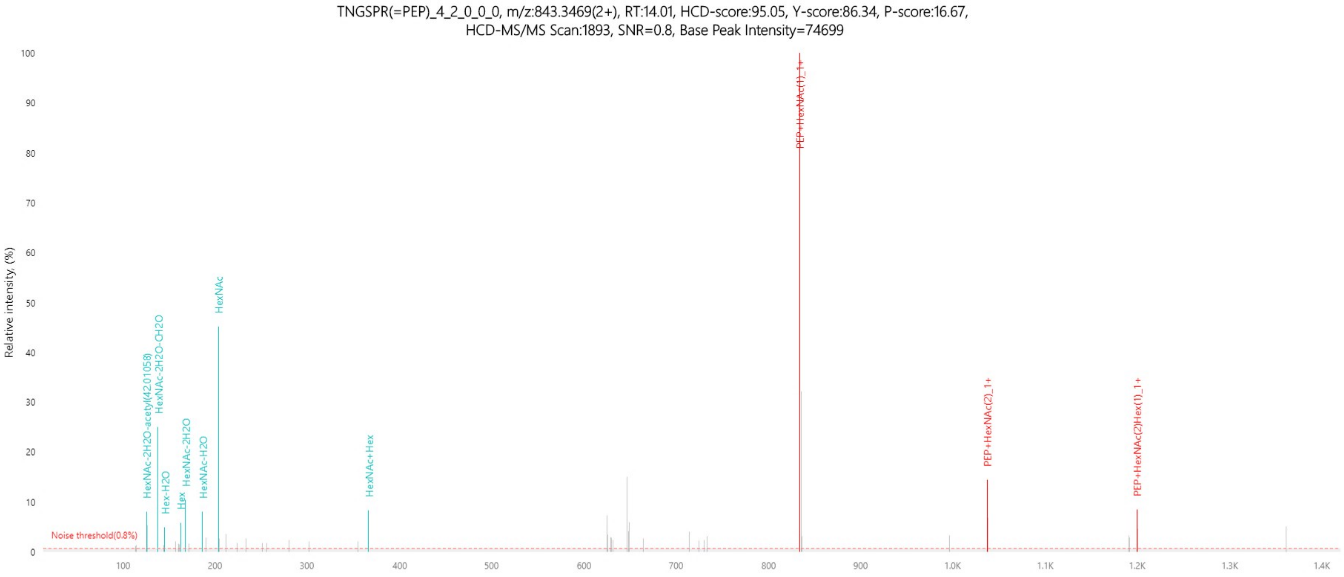
TNGSPR(=PEP)_4_2_0_0_0, m/z:843.3469(2+), RT:14.01, HCD-score:95.05, Y-score:86.34, P-score:16.67,
HCD-MS/MS Scan:1893, SNR=0.8, Base Peak Intensity=74699
Relative intensity, (%)
Noise threshold(0.8%)
HexNAc-2H2O-acetyl(42.01058)
HexNAc-2H2O-CH2O
Hex-H2O
Hex
HexNAc-2H2O
HexNAc-H2O
HexNAc
HexNAc+Hex
PEP+HexNAc(1)_1+
PEP+HexNAc(2)_1+
PEP+HexNAc(2)Hex(1)_1+

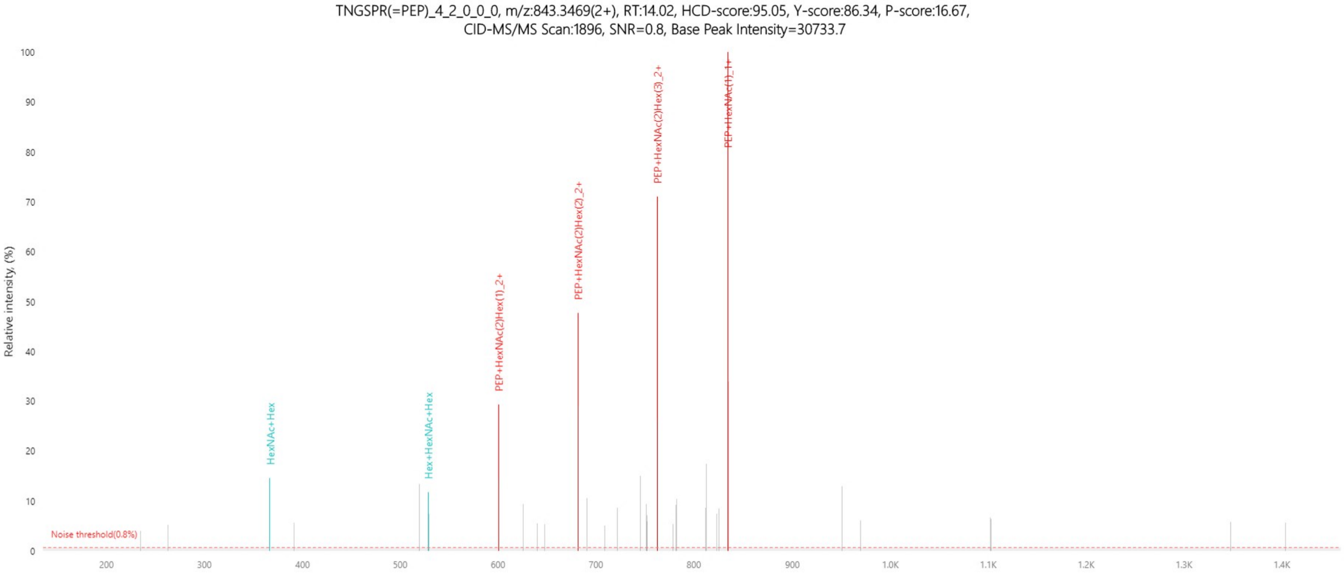
TNGSPR(=PEP)_4_2_0_0_0, m/z:843.3469(2+), RT:14.02, HCD-score:95.05, Y-score:86.34, P-score:16.67,
CID-MS/MS Scan:1896, SNR=0.8, Base Peak Intensity=30733.7
Relative intensity, (%)
Noise threshold(0.8%)
HexNAc+Hex
Hex+HexNAc+Hex
PEP+HexNAc(2)Hex(1)_2+
PEP+HexNAc(2)Hex(2)_2+
PEP+HexNAc(2)Hex(3)_2+
PEP+HexNAc(1)_1+

TNGSPR(=PEP)_5_2_0_0_0, m/z:924.3737(2+), RT:13.98, HCD-score:94.56, Y-score:88.92, P-score:16.67, HCD-MS/MS Scan:2092, SNR=0.8, Base Peak Intensity=15063989

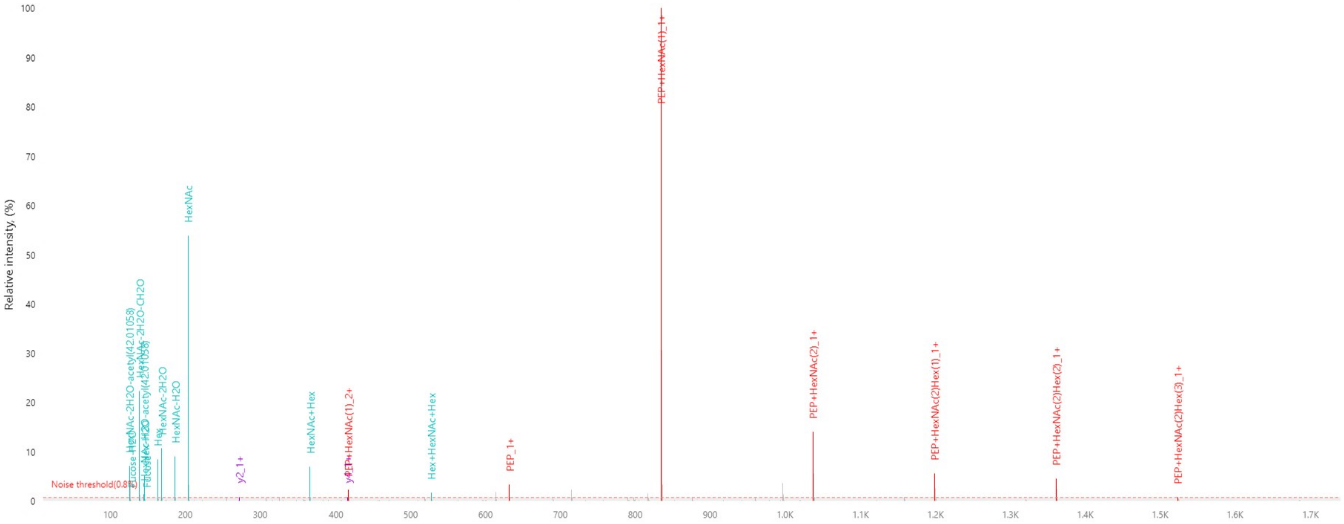

TNGSPR(=PEP)_5_2_0_0_0, m/z:924.3737(2+), RT:13.99, HCD-score:94.56, Y-score:88.92, P-score:16.67, CID-MS/MS Scan:2093, SNR=0.8, Base Peak Intensity=19555514

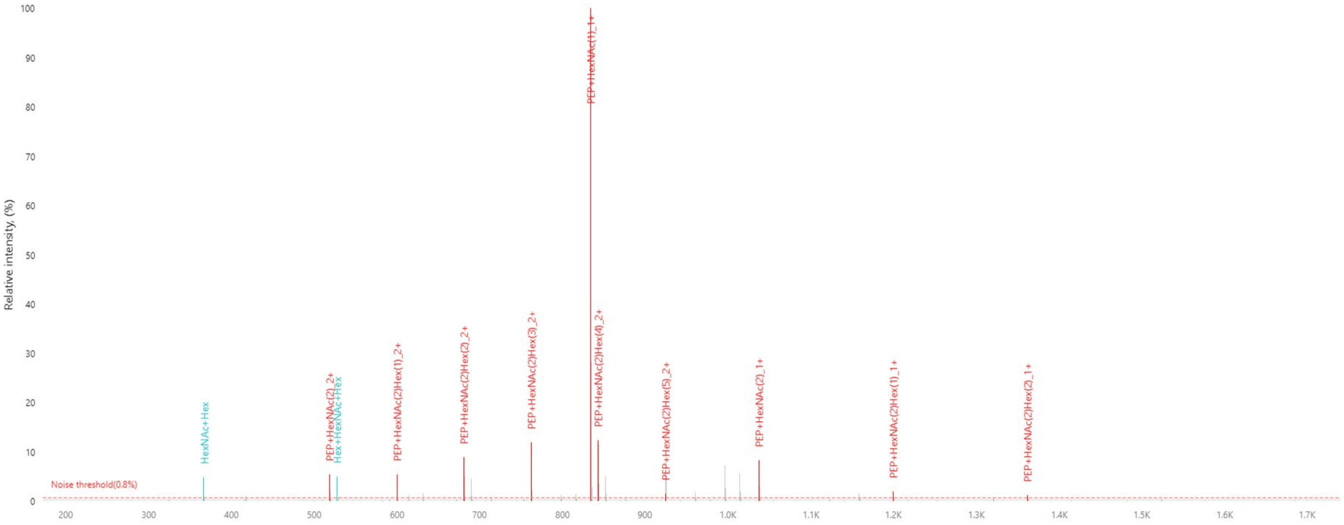

TNGSPR(=PEP)_5_3_0_0_1, m/z:786.6422(3+), RT:14.19, HCD-score:100.00, Y-score:62.53, P-score:,
HCD-MS/MS Scan:1999, SNR=0.8, Base Peak Intensity=15269.4

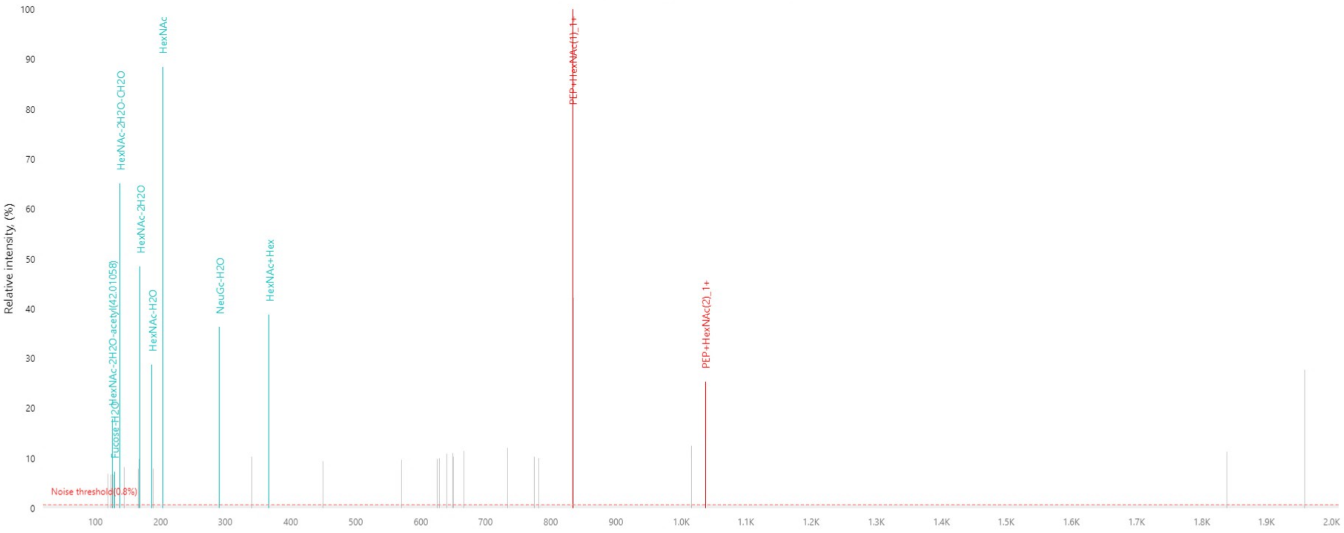

TNGSPR(=PEP)_5_3_0_0_1, m/z:786.6422(3+), RT:14.20, HCD-score:100.00, Y-score:62.53, P-score:,
CID-MS/MS Scan:2000, SNR=0.8, Base Peak Intensity=18974.5

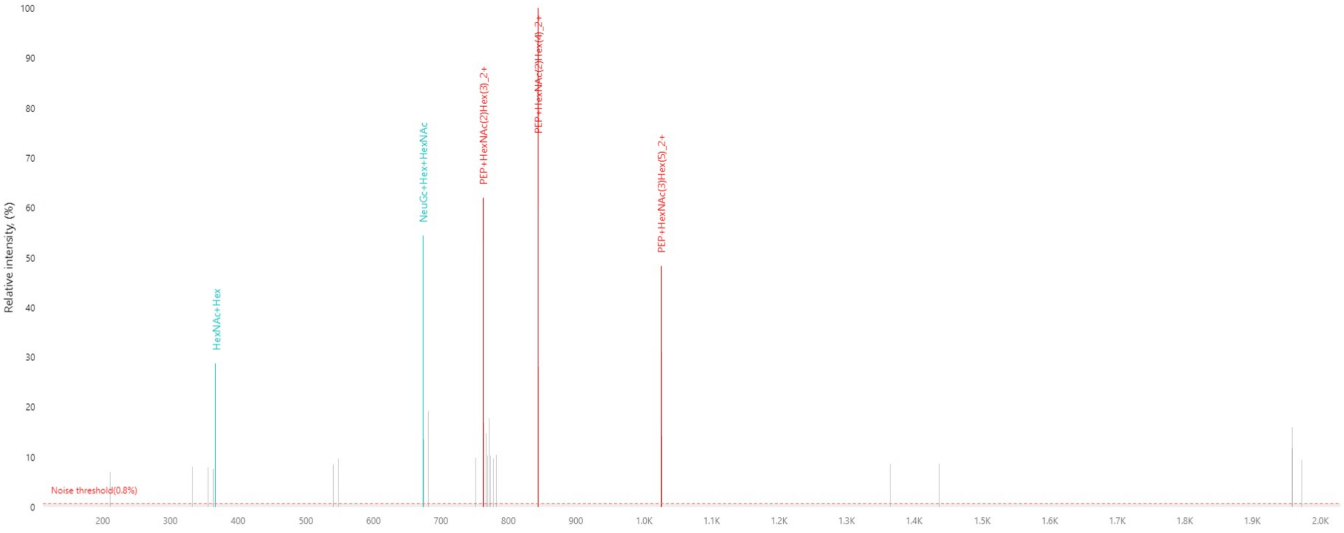

TNGSPR(=PEP)_6_2_0_0_0, m/z:1005.4003(2+), RT:12.63, HCD-score:100.00, Y-score:100.00, P-score:0.00,
HCD-MS/MS Scan:1712, SNR=0.8, Base Peak Intensity=38416.7

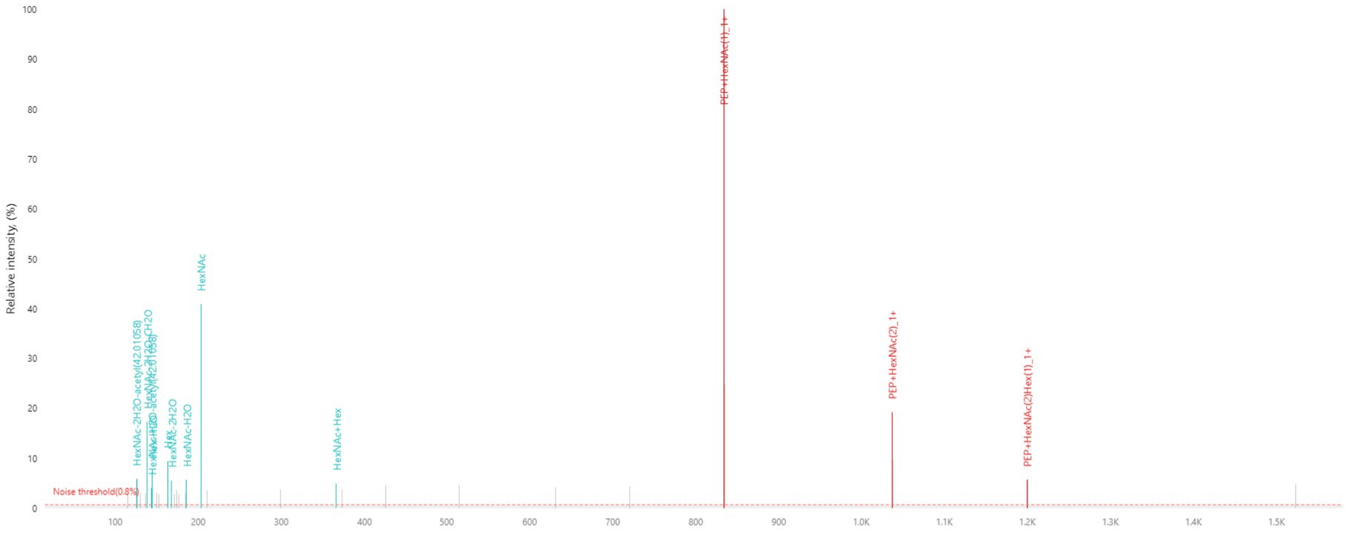

TNGSPR(=PEP)_6_2_0_0_0, m/z:1005.4003(2+), RT:12.63, HCD-score:100.00, Y-score:100.00, P-score:0.00,
CID-MS/MS Scan:1713, SNR=0.8, Base Peak Intensity=27238.7

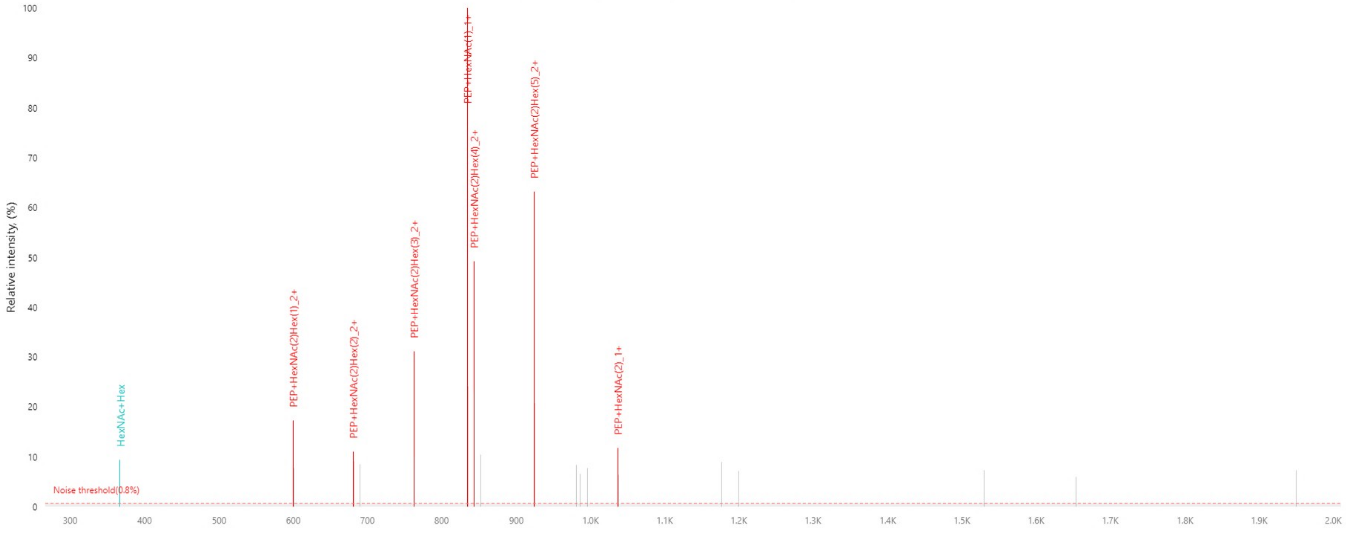

TNGSPR(=PEP)_6_3_0_0_1, m/z:840.6601(3+), RT:14.08, HCD-score:97.87, Y-score:95.83, P-score:,
HCD-MS/MS Scan:2093, SNR=0.8, Base Peak Intensity=100072.8

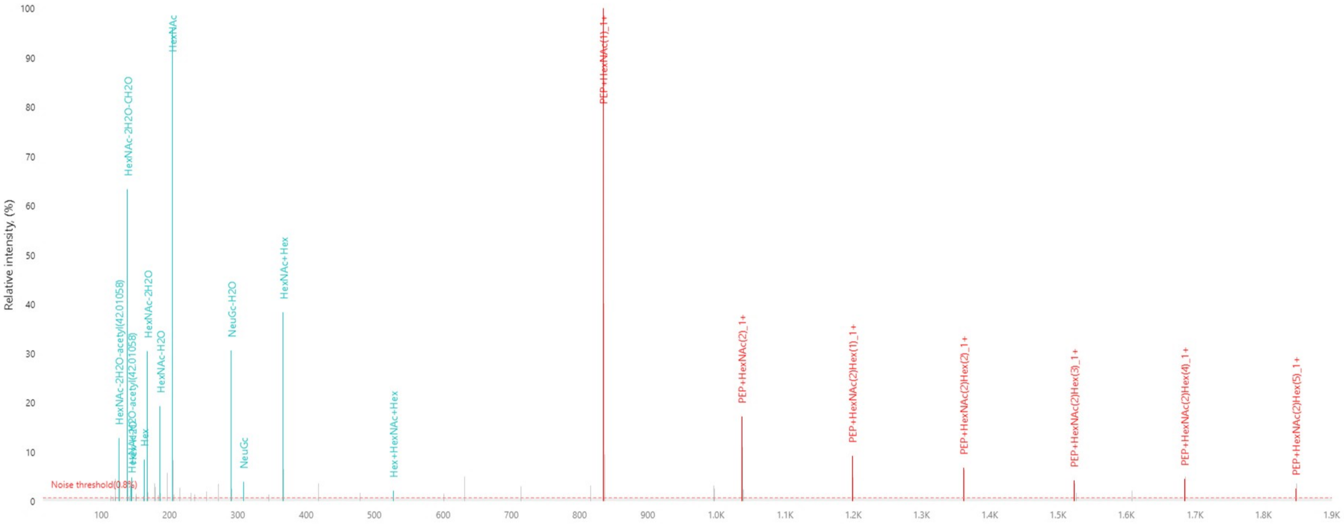

TNGSPR(=PEP)_6_3_0_0_1, m/z:840.6601(3+), RT:14.09, HCD-score:97.87, Y-score:95.83, P-score:,
CID-MS/MS Scan:2095, SNR=0.8, Base Peak Intensity=88006.7

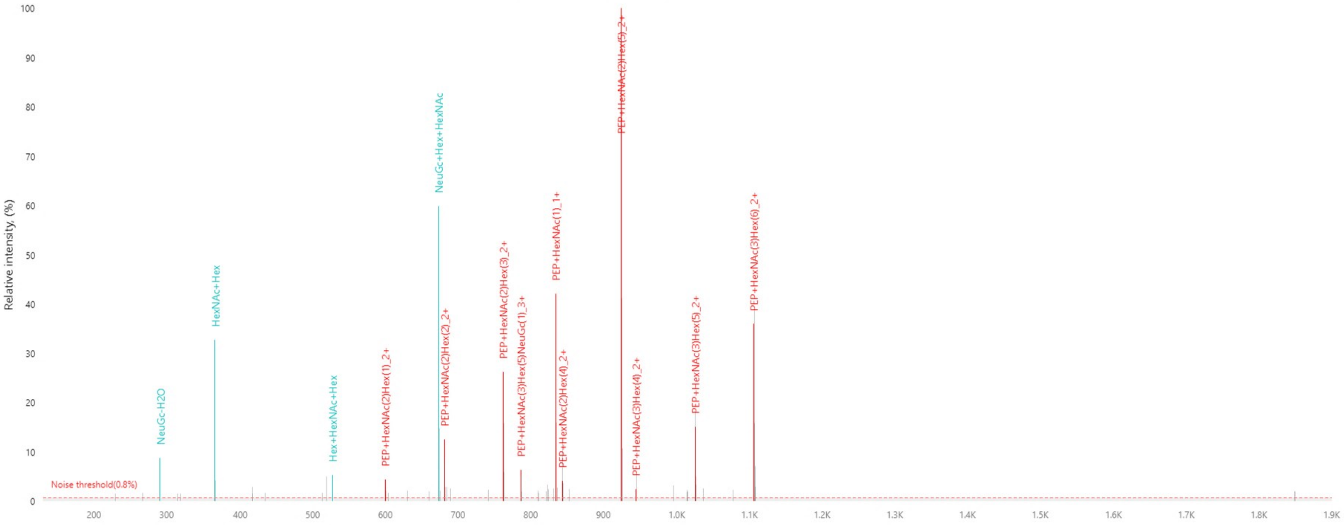

TNGSPR(=PEP)_7_2_0_0_0, m/z:1086.4259(2+), RT:8.81, HCD-score:94.81, Y-score:88.21, P-score:0.00,
HCD-MS/MS Scan:1400, SNR=0.8, Base Peak Intensity=398523.8

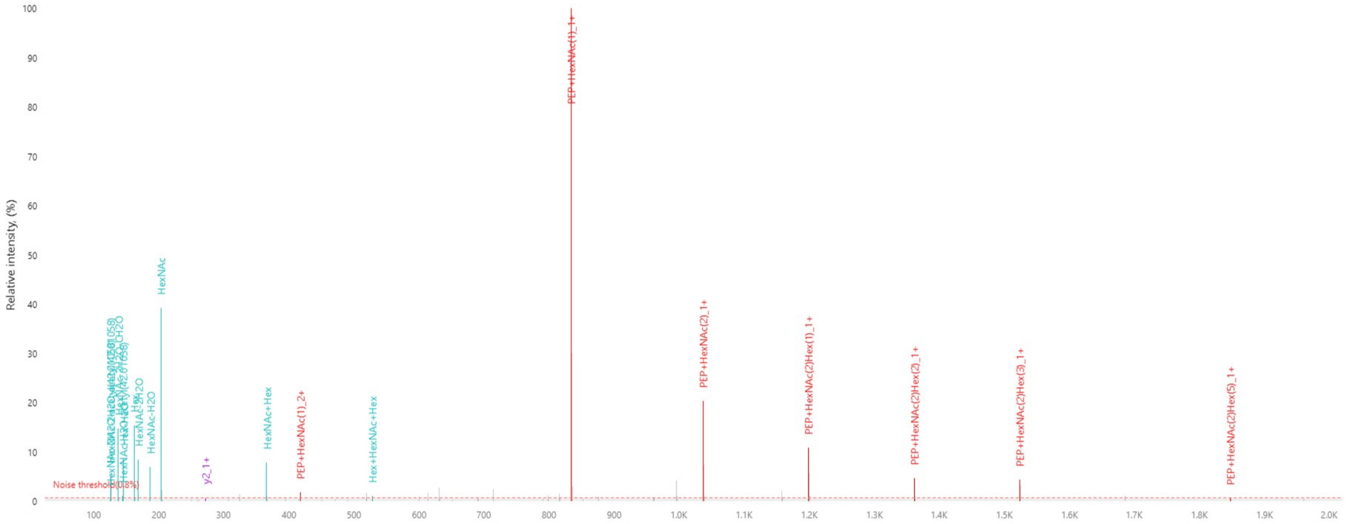

TNGSPR(=PEP)_7_2_0_0_0, m/z:1086.4259(2+), RT:8.82, HCD-score:94.81, Y-score:88.21, P-score:0.00,
CID-MS/MS Scan:1401, SNR=0.8, Base Peak Intensity=308976.9

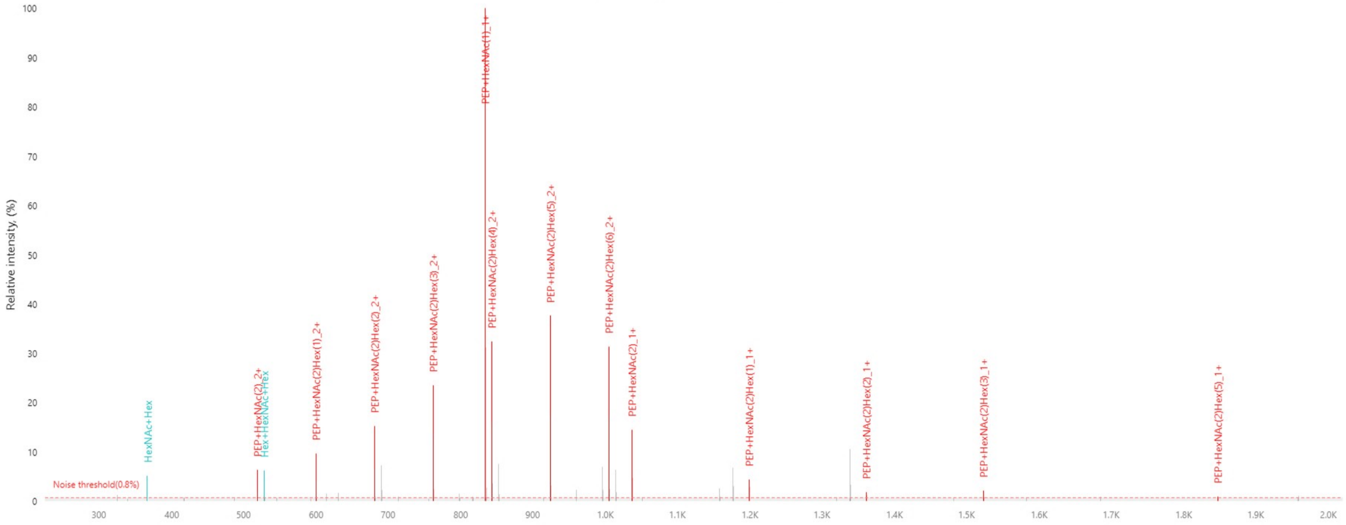

TNGSPR(=PEP)_8_2_0_0_0, m/z:1167.4531(2+), RT:8.05, HCD-score:100.00, Y-score:96.98, P-score:0.00,
HCD-MS/MS Scan:1276, SNR=0.8, Base Peak Intensity=63757.2

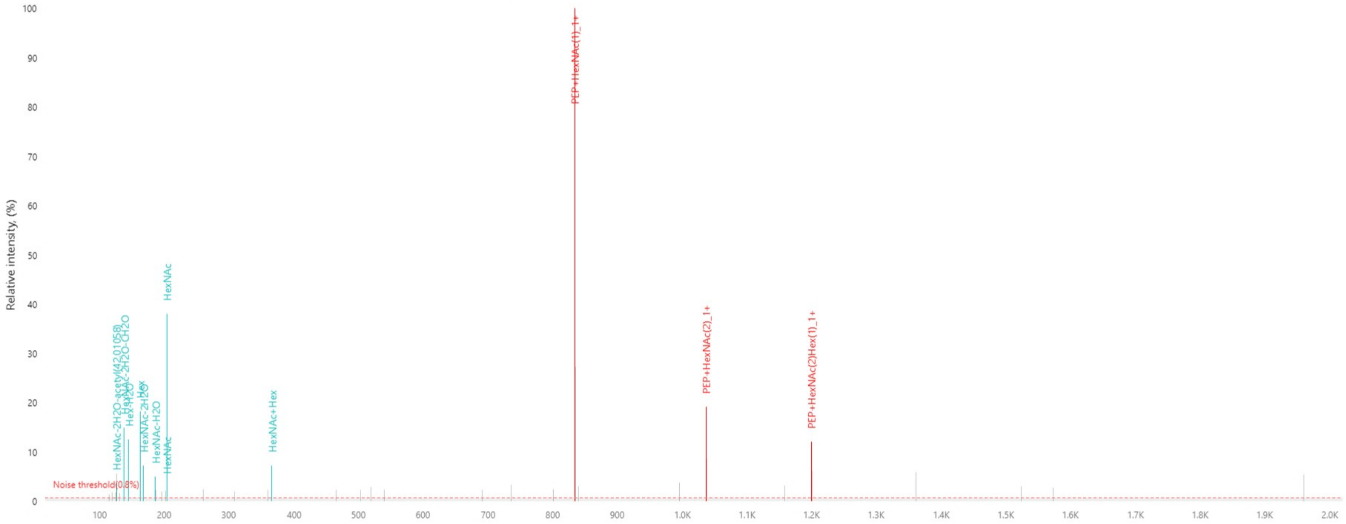

TNGSPR(=PEP)_8_2_0_0_0, m/z:1167.4531(2+), RT:8.06, HCD-score:100.00, Y-score:96.98, P-score:0.00,
CID-MS/MS Scan:1278, SNR=0.8, Base Peak Intensity=45870.1

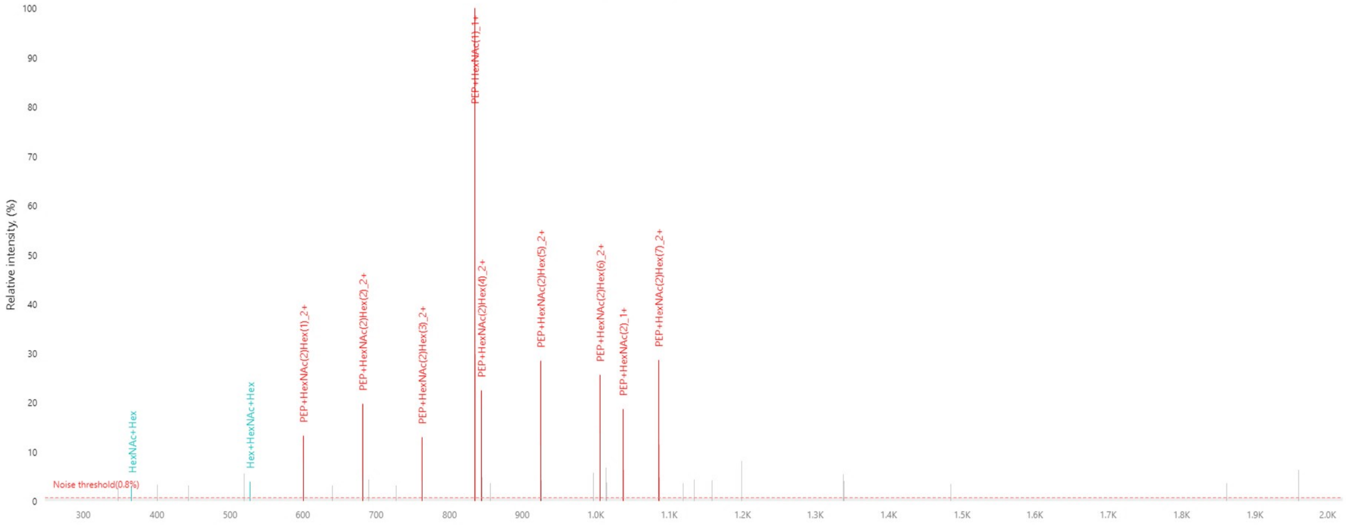

Supplement: Supplementary file 2 [file Image3.pdf]
