# Supplementary material for: Advanced assessment through intact glycopeptide analysis of Infliximab’s biologics and biosimilar
Source: Front Mol Biosci. 2022 Nov 29;9:1006866. doi: 10.3389/fmolb.2022.1006866 (PMC9745114; doi:10.3389/fmolb.2022.1006866)

Glycan_Types	Glycopeptides	HCD_scan	CID_scan	Raw File	Page
N	EEQYNSTYR_3_2_0_0_0	4308	4311	Remicade_Batch1_01	2
N	EEQYNSTYR_3_2_1_0_0	4523	4526	Remicade_Batch1_01	3
N	EEQYNSTYR_3_3_0_0_0	4435	4437	Remicade_Batch1_01	4
N	EEQYNSTYR_3_3_1_0_0	4357	4362	Remicade_Batch1_01	5
N	EEQYNSTYR_3_4_0_0_0	4461	4463	Remicade_Batch1_01	6
N	EEQYNSTYR_3_4_1_0_0	5098	5100	Remicade_Batch1_01	7
N	EEQYNSTYR_3_5_1_0_0	4477	4480	Remicade_Batch2_02	8
N	EEQYNSTYR_4_2_0_0_0	4253	4255	Remicade_Batch1_01	9
N	EEQYNSTYR_4_2_1_0_0	4228	4231	Remicade_Batch1_01	10
N	EEQYNSTYR_4_3_0_0_0	4342	4344	Remicade_Batch1_01	11
N	EEQYNSTYR_4_3_0_0_1	6848	6850	Remicade_Batch1_01	12
N	EEQYNSTYR_4_3_0_1_0	6941	6944	Remicade_Batch2_01	13
N	EEQYNSTYR_4_3_1_0_0	6791	6793	Remicade_Batch1_01	14
N	EEQYNSTYR_4_3_1_0_1	6781	6784	Remicade_Batch1_01	15
N	EEQYNSTYR_4_3_1_1_0	7014	7016	Remicade_Batch1_01	16
N	EEQYNSTYR_4_3_2_0_0	4563	4566	Remicade_Batch1_01	17
N	EEQYNSTYR_4_4_0_0_0	4411	4413	Remicade_Batch1_01	18
N	EEQYNSTYR_4_4_0_0_1	6932	6934	Remicade_Batch1_01	19
N	EEQYNSTYR_4_4_1_0_0	4350	4353	Remicade_Batch1_01	20
N	EEQYNSTYR_4_4_1_0_1	6812	6814	Remicade_Batch1_01	21
N	EEQYNSTYR_4_4_1_1_0	7022	7024	Remicade_Batch1_03	22
N	EEQYNSTYR_4_4_2_0_0	4593	4596	Remicade_Batch5_03	23
N	EEQYNSTYR_4_5_0_0_0	4529	4532	Remicade_Batch1_01	24
N	EEQYNSTYR_4_5_1_0_0	4618	4621	Remicade_Batch1_03	25
N	EEQYNSTYR_5_2_0_0_0	4336	4338	Remicade_Batch1_01	26
N	EEQYNSTYR_5_2_1_0_0	4255	4258	Remicade_Batch1_01	27
N	EEQYNSTYR_5_3_0_0_0	4218	4221	Remicade_Batch1_01	28
N	EEQYNSTYR_5_3_0_0_1	6700	6702	Remicade_Batch1_01	29
N	EEQYNSTYR_5_3_1_0_0	4412	4414	Remicade_Batch1_01	30
N	EEQYNSTYR_5_3_1_0_1	6642	6644	Remicade_Batch1_01	31
N	EEQYNSTYR_5_4_1_0_0	4448	4450	Remicade_Batch1_01	32
N	EEQYNSTYR_5_4_1_0_1	6607	6609	Remicade_Batch1_01	33
N	EEQYNSTYR_5_4_1_0_2	8685	8687	Remicade_Batch1_01	34
N	EEQYNSTYR_5_4_1_1_0	6822	6824	Remicade_Batch4_02	35
N	EEQYNSTYR_6_2_0_0_0	4124	4126	Remicade_Batch1_01	36
N	EEQYNSTYR_6_3_0_0_0	4247	4249	Remicade_Batch1_01	37
N	EEQYNSTYR_6_3_0_0_1	6650	6652	Remicade_Batch1_01	38
N	EEQYNSTYR_6_3_1_0_0	4173	4175	Remicade_Batch1_01	39
N	EEQYNSTYR_6_3_1_0_1	6544	6546	Remicade_Batch1_01	40
N	EEQYNSTYR_6_4_1_0_0	4260	4262	Remicade_Batch1_01	41
N	EEQYNSTYR_6_4_1_0_1	6567	6569	Remicade_Batch1_01	42
N	EEQYNSTYR_7_2_0_0_0	4018	4020	Remicade_Batch1_01	43
N	EEQYNSTYR_7_4_1_0_0	4223	4225	Remicade_Batch1_01	44
N	EEQYNSTYR_8_2_0_0_0	3951	3954	Remicade_Batch1_01	45
N	TNGSPR_5_2_0_0_0	1903	1905	Remicade_Batch1_01	46
N	TNGSPR_5_3_0_0_0	1907	1909	Remicade_Batch3_02	47
N	TNGSPR_6_3_0_0_0	1884	1886	Remicade_Batch4_01	48
N	TNGSPR_6_3_0_0_1	1923	1925	Remicade_Batch3_03	49
N	TNGSPR_7_2_0_0_0	1247	1248	Remicade_Batch5_02	50

EEQYNSTYR(=PEP)_3_2_0_0_0, m/z:694.6161(3+), RT:22.84, HCD-score:77.98, Y-score:87.53, P-score:88.89,
HCD-MS/MS Scan:4308, SNR=0.8, Base Peak Intensity=4156174.8

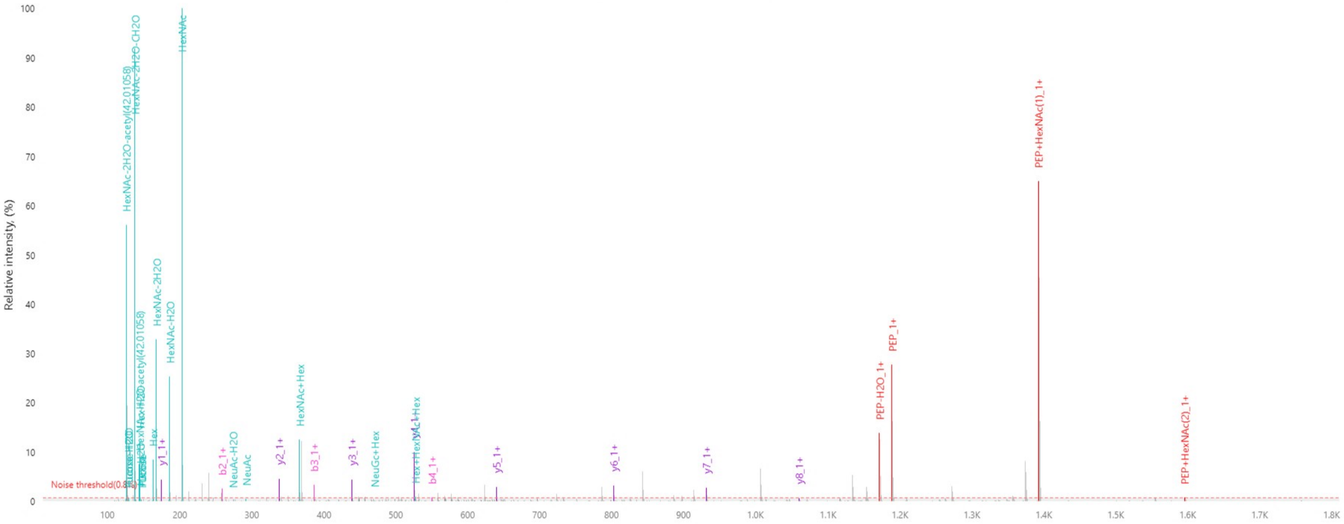

EEQYNSTYR(=PEP)_3_2_0_0_0, m/z:694.6161(3+), RT:22.85, HCD-score:77.98, Y-score:87.53, P-score:88.89,
CID-MS/MS Scan:4311, SNR=0.8, Base Peak Intensity=3714495

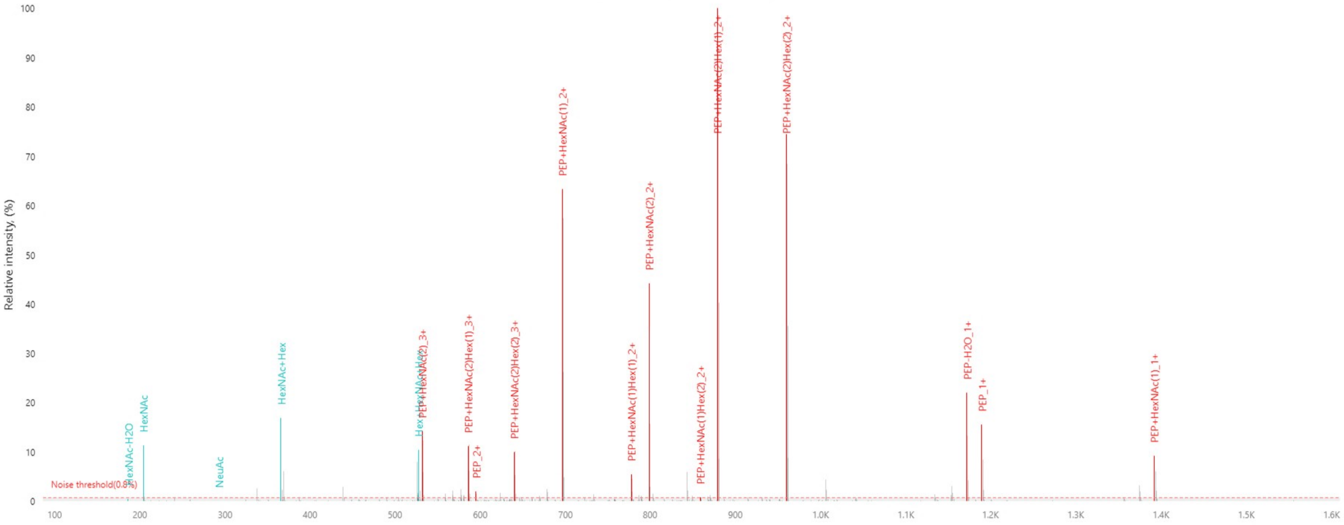

EEQYNSTYR(=PEP)_3_2_1_0_0, m/z:1114.4474(2+), RT:23.94, HCD-score:88.87, Y-score:85.71, P-score:11.11,
HCD-MS/MS Scan:4523, SNR=0.8, Base Peak Intensity=889411

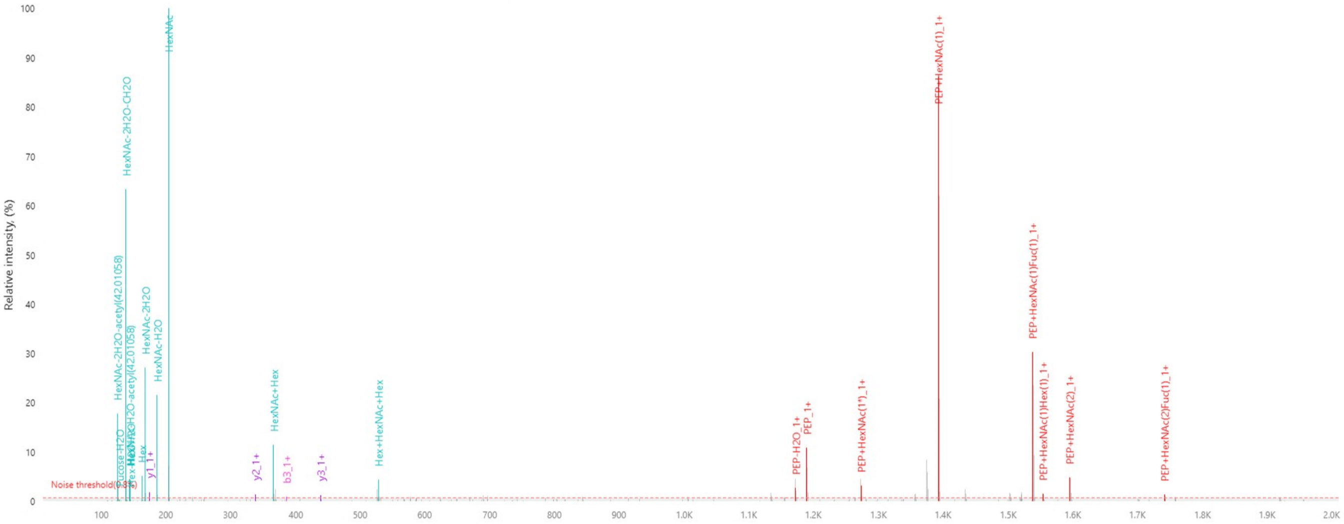

EEQYNSTYR(=PEP)_3_2_1_0_0, m/z:1114.4474(2+), RT:23.95, HCD-score:88.87, Y-score:85.71, P-score:11.11,
CID-MS/MS Scan:4526, SNR=0.8, Base Peak Intensity=788170.9

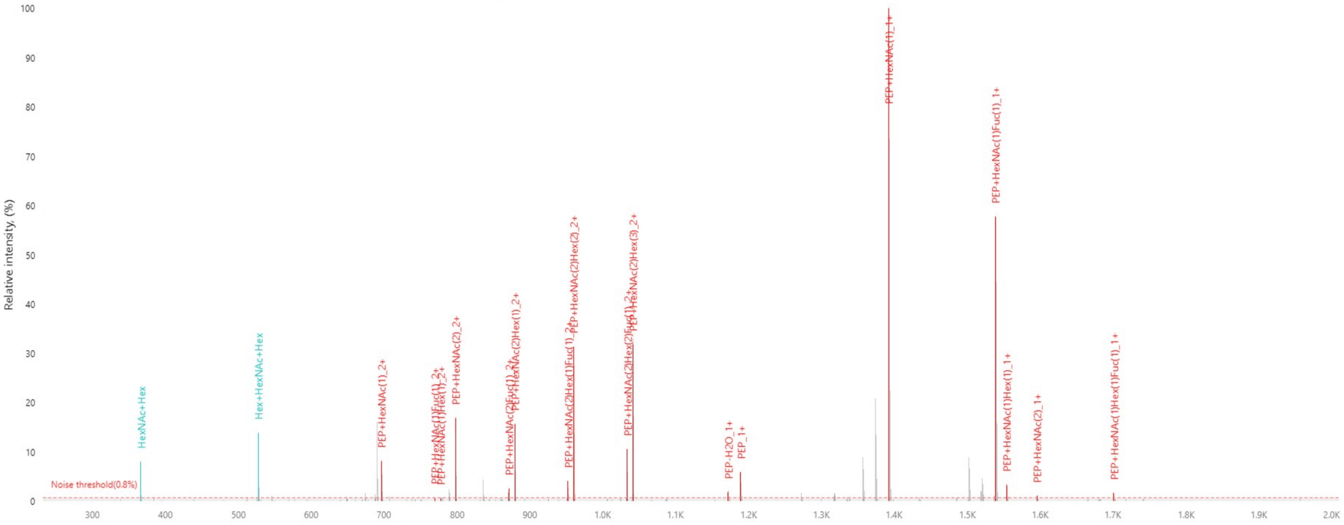

EEQYNSTYR(=PEP)_3_3_0_0_0, m/z:1142.9567(2+), RT:23.62, HCD-score:93.52, Y-score:85.70, P-score:22.22,
HCD-MS/MS Scan:4435, SNR=0.8, Base Peak Intensity=7123495.5

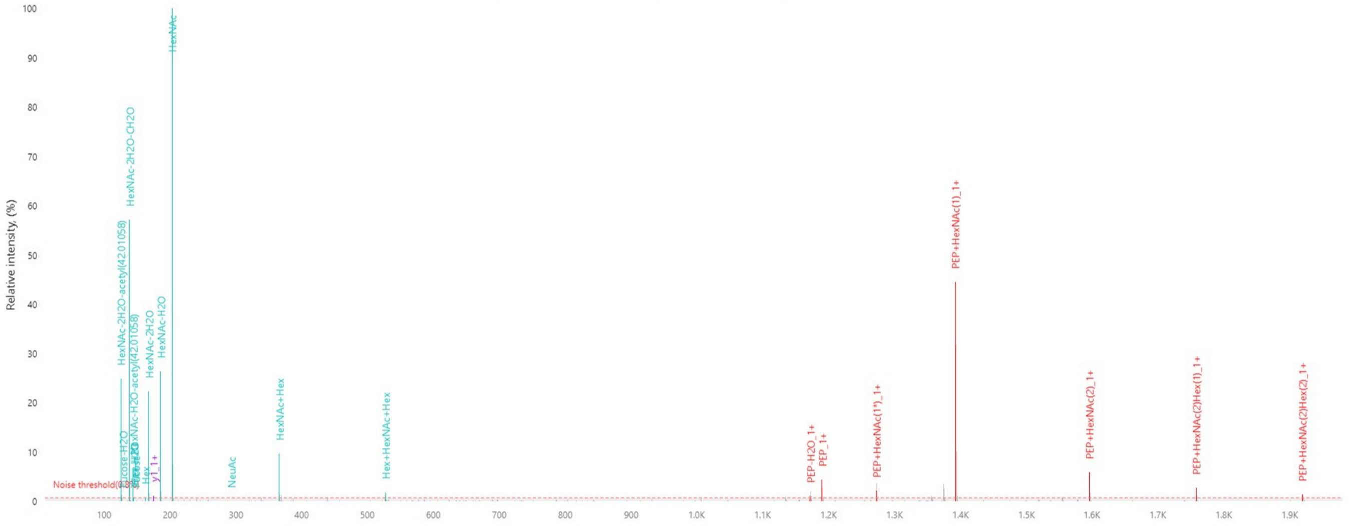

EEQYNSTYR(=PEP)_3_3_0_0_0, m/z:1142.9567(2+), RT:23.62, HCD-score:93.52, Y-score:85.70, P-score:22.22,
CID-MS/MS Scan:4437, SNR=0.8, Base Peak Intensity=10574063

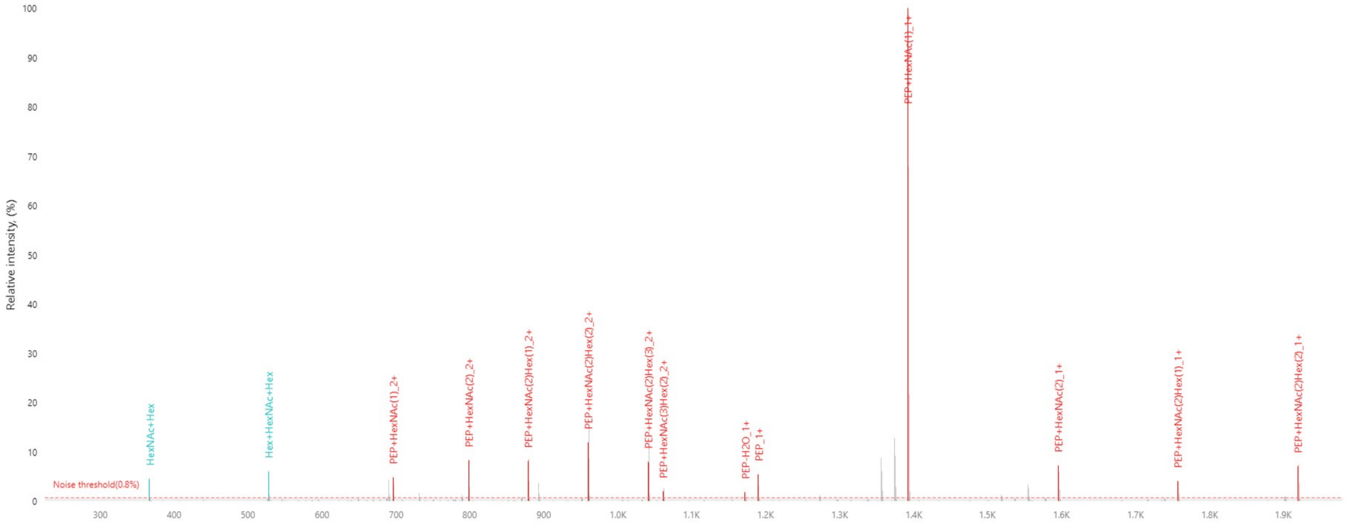

EEQYNSTYR(=PEP)_3_3_1_0_0, m/z:1215.9856(2+), RT:23.34, HCD-score:96.12, Y-score:89.35, P-score:0.00,
HCD-MS/MS Scan:4357, SNR=0.8, Base Peak Intensity=3415668.8

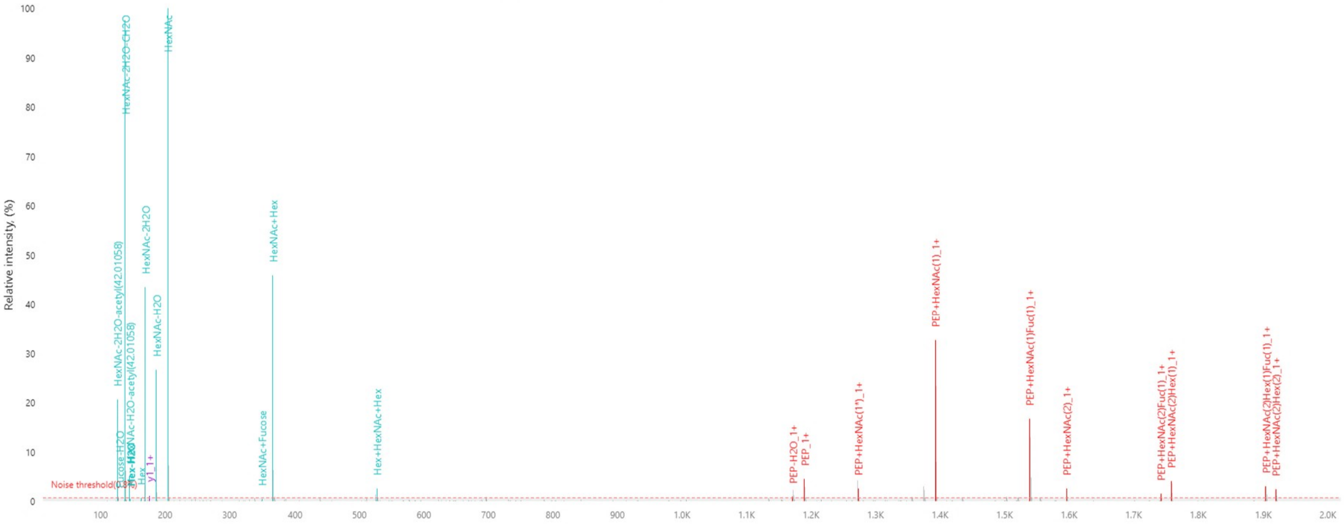

EEQYNSTYR(=PEP)_3_3_1_0_0, m/z:1215.9856(2+), RT:23.36, HCD-score:96.12, Y-score:89.35, P-score:0.00,
CID-MS/MS Scan:4362, SNR=0.8, Base Peak Intensity=3335817.2

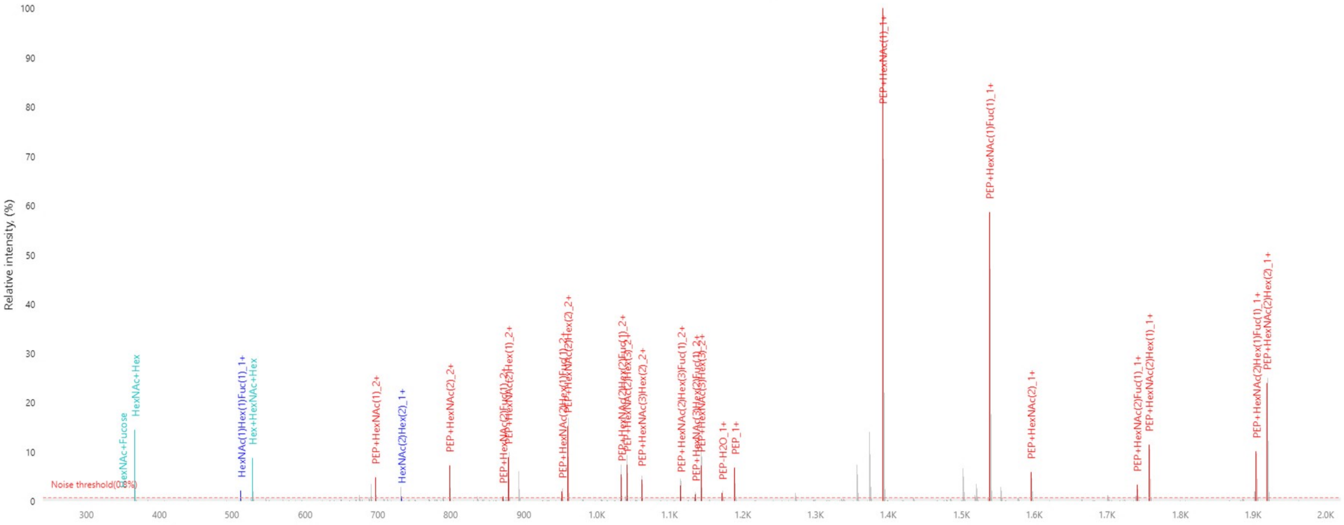

EEQYNSTYR(=PEP)_3_4_0_0_0, m/z:830.0020(3+), RT:23.72, HCD-score:94.73, Y-score:98.42, P-score:33.33,
HCD-MS/MS Scan:4461, SNR=0.8, Base Peak Intensity=14228334

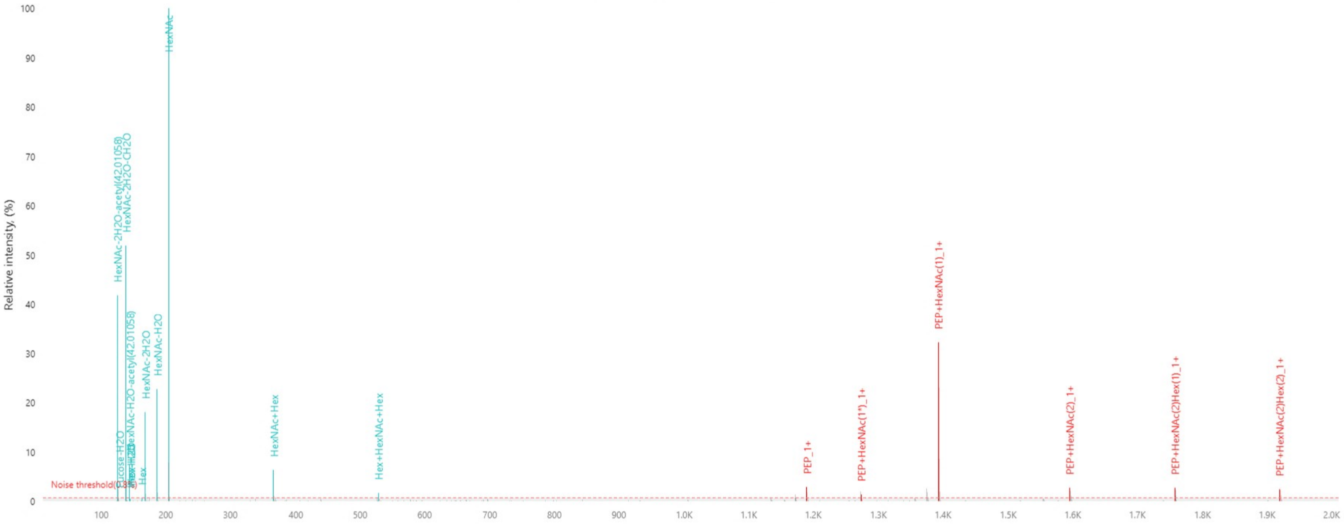

EEQYNSTYR(=PEP)_3_4_0_0_0, m/z:830.0020(3+), RT:23.73, HCD-score:94.73, Y-score:98.42, P-score:33.33,
CID-MS/MS Scan:4463, SNR=0.8, Base Peak Intensity=20184232

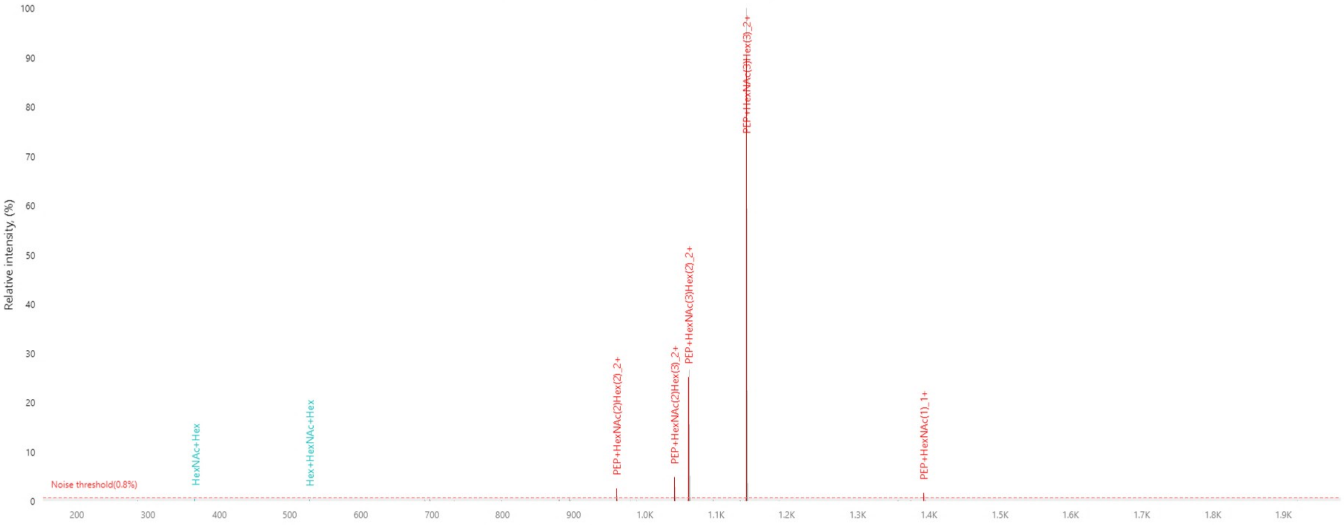

EEQYNSTYR(=PEP)_3_4_1_0_0, m/z:878.6878(3+), RT:26.04, HCD-score:95.53, Y-score:63.66, P-score:22.22,
HCD-MS/MS Scan:5098, SNR=0.8, Base Peak Intensity=1545371

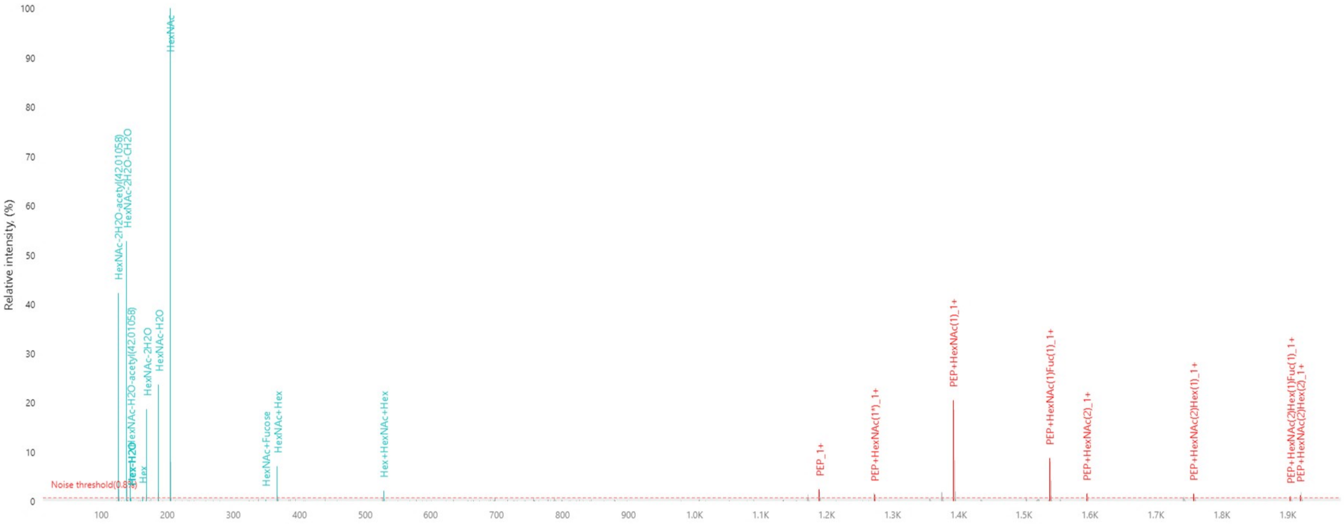

EEQYNSTYR(=PEP)_3_4_1_0_0, m/z:878.6878(3+), RT:26.04, HCD-score:95.53, Y-score:63.66, P-score:22.22,
CID-MS/MS Scan:5100, SNR=0.8, Base Peak Intensity=1778436.1

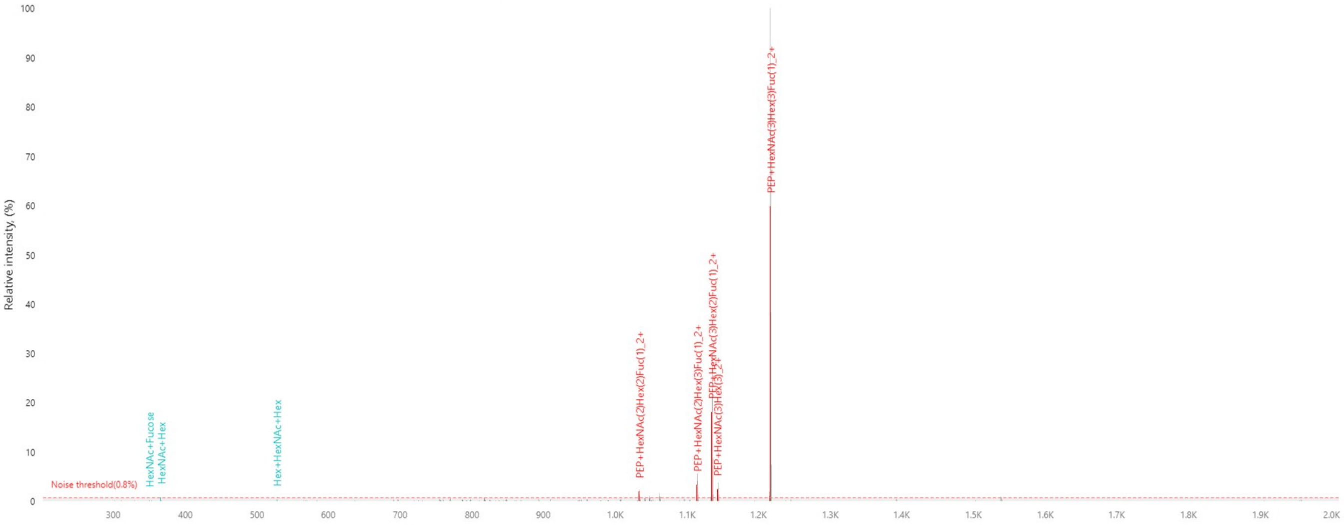

EEQYNSTYR(=PEP)_3_5_1_0_0, m/z:1419.0657(2+), RT:23.63, HCD-score:73.19, Y-score:77.75, P-score:,
HCD-MS/MS Scan:4477, SNR=0.8, Base Peak Intensity=338422.6

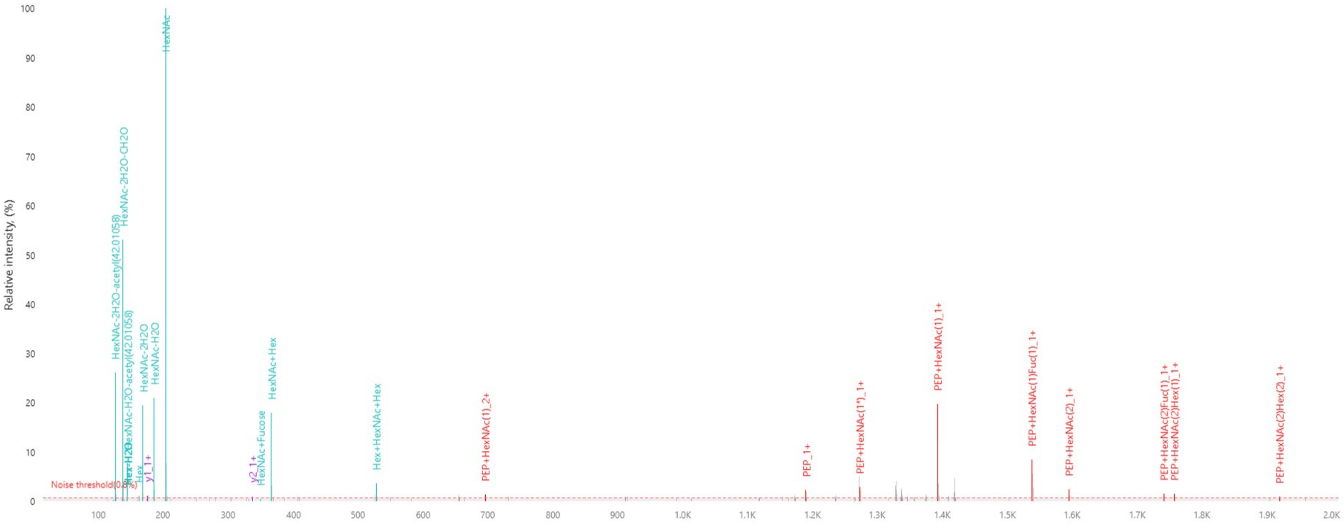

EEQYNSTYR(=PEP)_3_5_1_0_0, m/z:1419.0657(2+), RT:23.64, HCD-score:73.19, Y-score:77.75, P-score:,
CID-MS/MS Scan:4480, SNR=0.8, Base Peak Intensity=120583.7

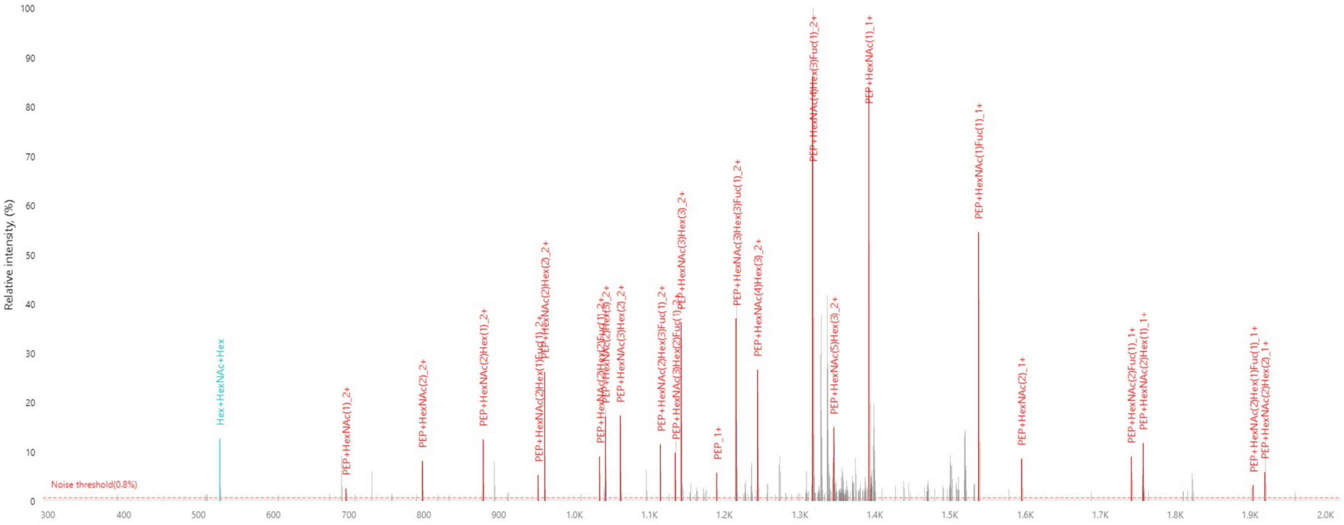

EEQYNSTYR(=PEP)_4_2_0_0_0, m/z:748.6355(3+), RT:22.97, HCD-score:81.24, Y-score:89.09, P-score:66.67, HCD-MS/MS Scan:4253, SNR=0.8, Base Peak Intensity=8209496

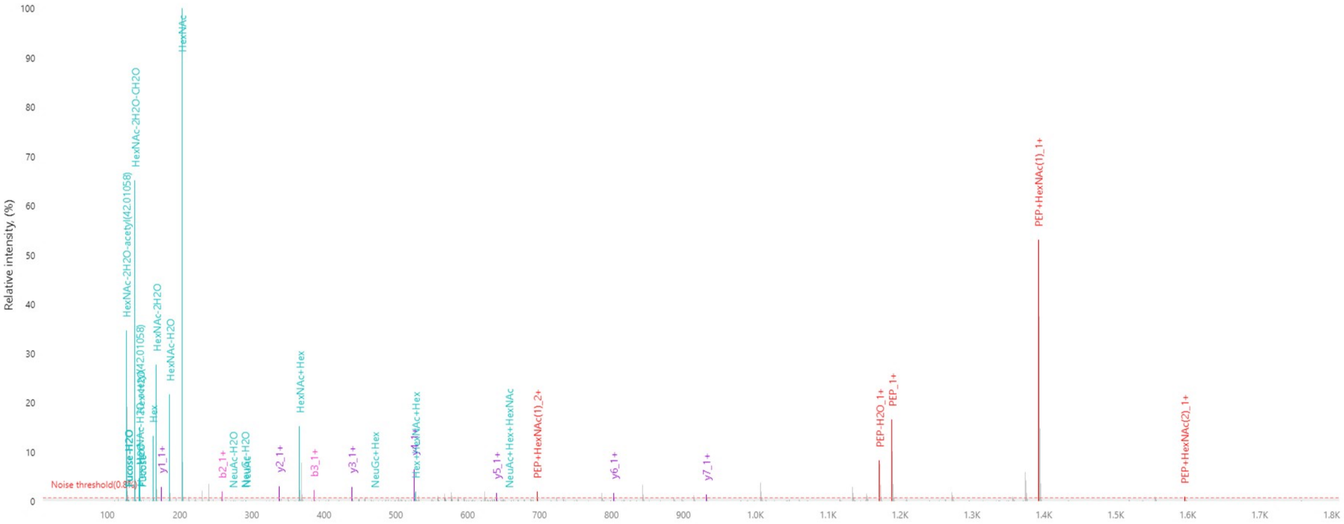

EEQYNSTYR(=PEP)_4_2_0_0_0, m/z:748.6355(3+), RT:22.98, HCD-score:81.24, Y-score:89.09, P-score:66.67, CID-MS/MS Scan:4255, SNR=0.8, Base Peak Intensity=8430549

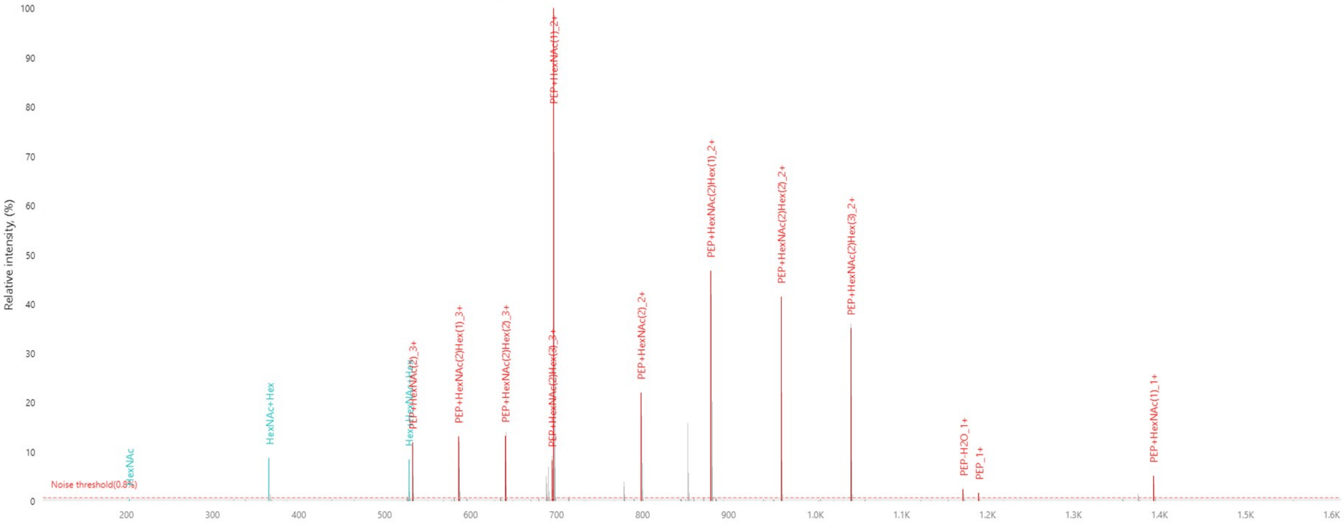

EEQYNSTYR(=PEP)_4_2_1_0_0, m/z:1195.4760(2+), RT:22.88, HCD-score:86.67, Y-score:83.10, P-score:0.00,
HCD-MS/MS Scan:4228, SNR=0.8, Base Peak Intensity=1095806

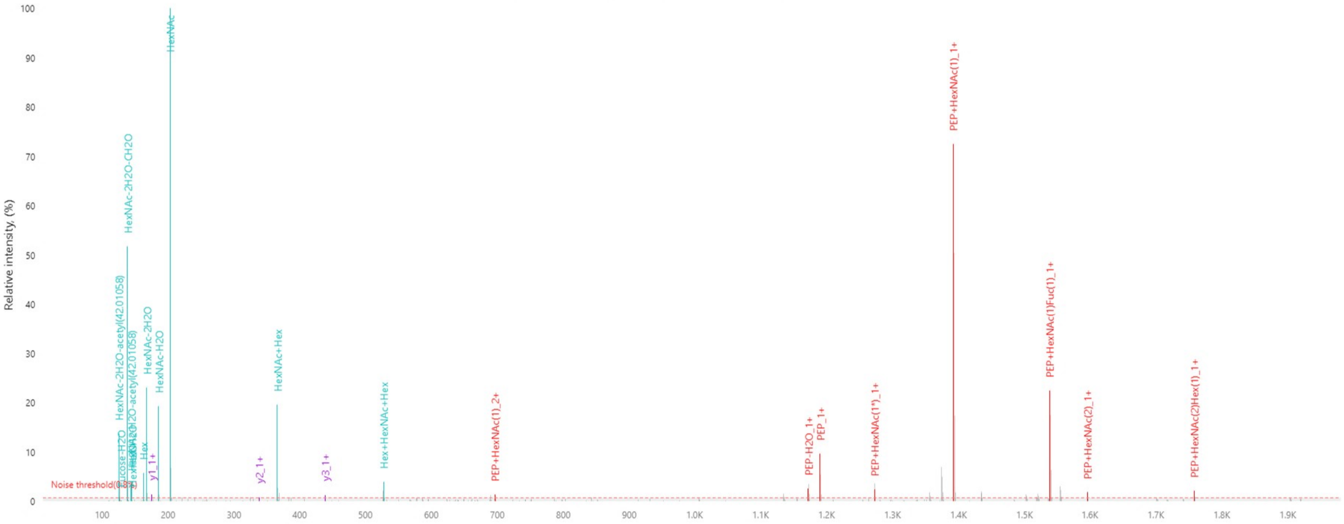

EEQYNSTYR(=PEP)_4_2_1_0_0, m/z:1195.4760(2+), RT:22.89, HCD-score:86.67, Y-score:83.10, P-score:0.00,
CID-MS/MS Scan:4231, SNR=0.8, Base Peak Intensity=884970.7

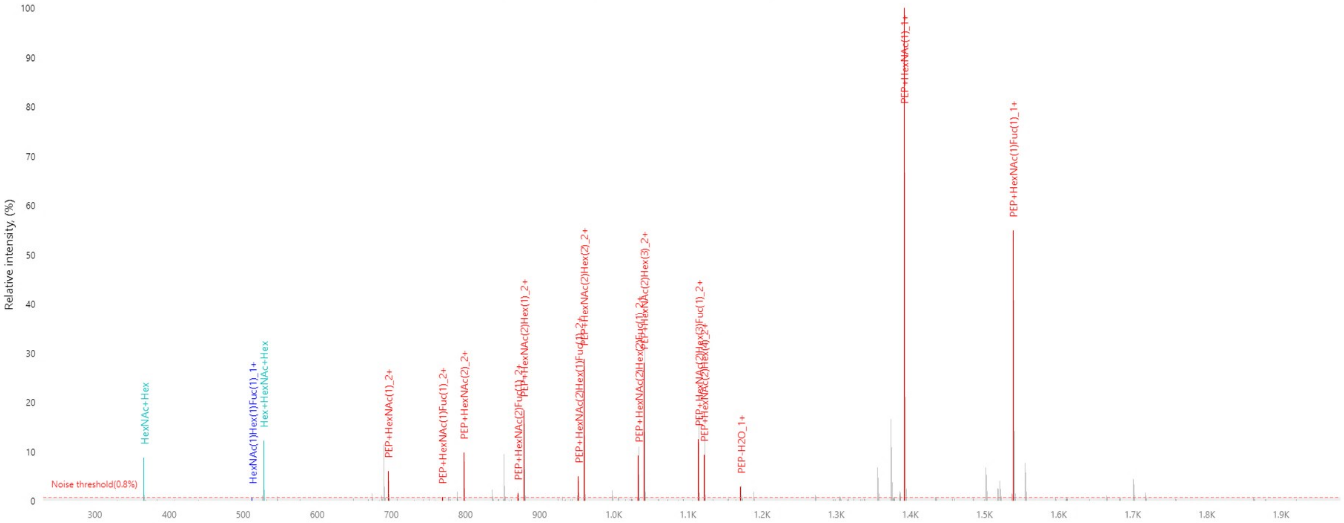

EEQYNSTYR(=PEP)_4_3_0_0_0, m/z:1223.9858(2+), RT:23.30, HCD-score:91.61, Y-score:81.22, P-score:0.00,
HCD-MS/MS Scan:4342, SNR=0.8, Base Peak Intensity=6836293

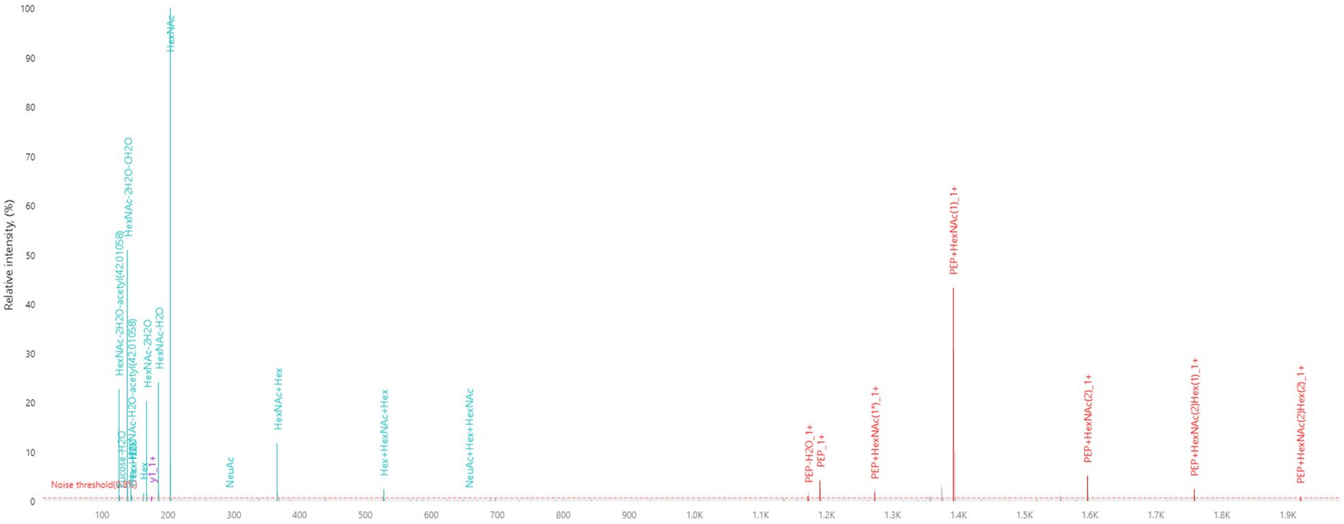

EEQYNSTYR(=PEP)_4_3_0_0_0, m/z:1223.9858(2+), RT:23.30, HCD-score:91.61, Y-score:81.22, P-score:0.00,
CID-MS/MS Scan:4344, SNR=0.8, Base Peak Intensity=8712746

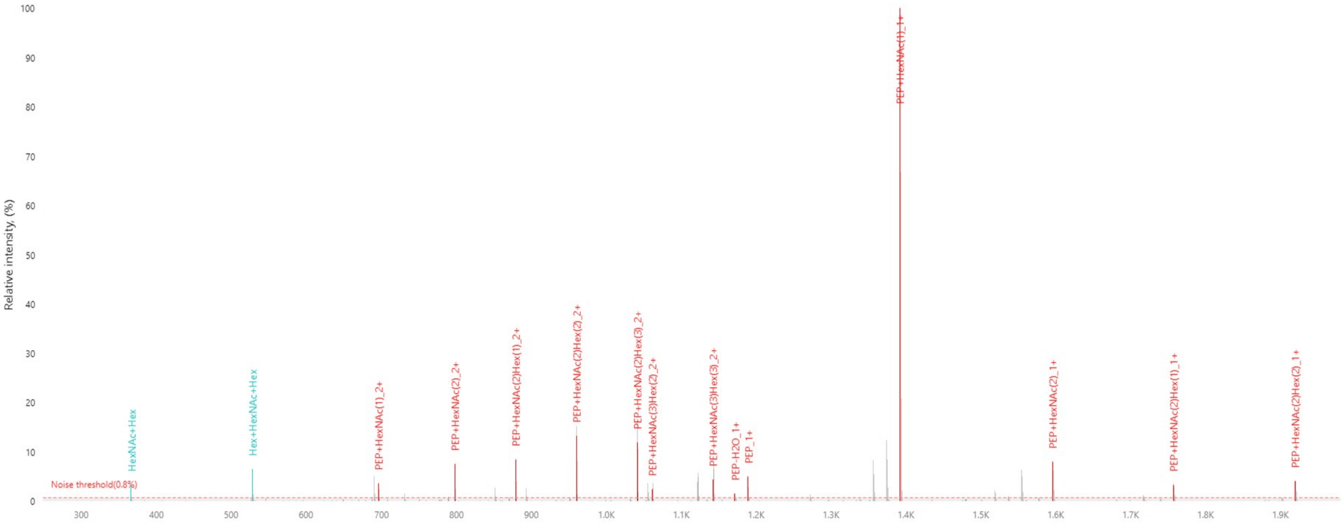

EEQYNSTYR(=PEP)_4_3_0_0_1, m/z:918.6870(3+), RT:31.94, HCD-score:90.42, Y-score:94.63, P-score:0.00,
HCD-MS/MS Scan:6848, SNR=0.8, Base Peak Intensity=1568108.8

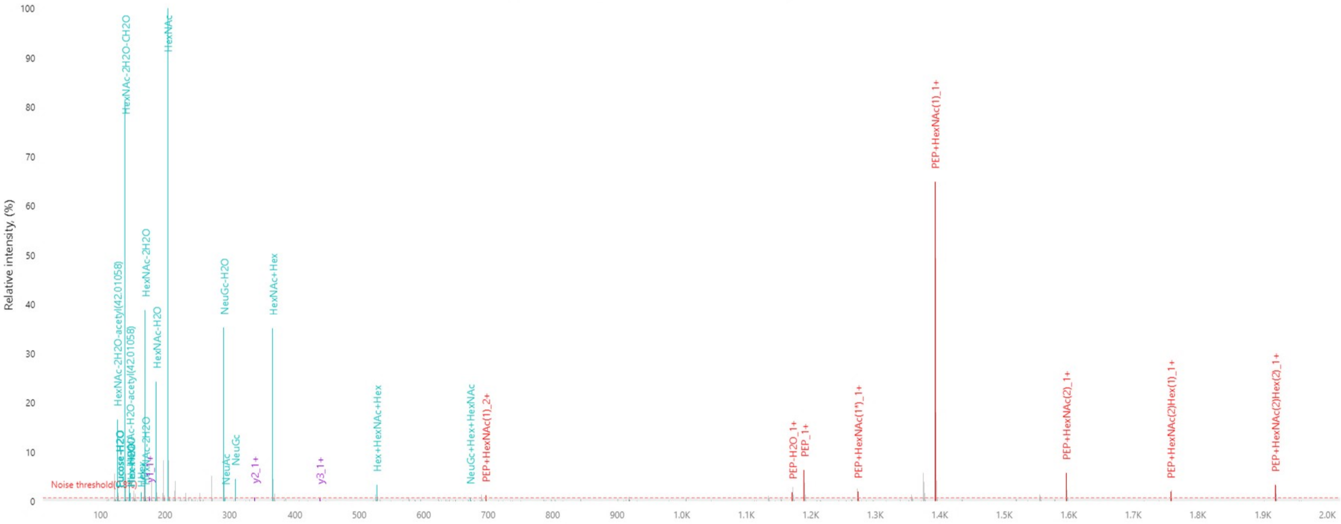

EEQYNSTYR(=PEP)_4_3_0_0_1, m/z:918.6870(3+), RT:31.94, HCD-score:90.42, Y-score:94.63, P-score:0.00,
CID-MS/MS Scan:6850, SNR=0.8, Base Peak Intensity=2165681

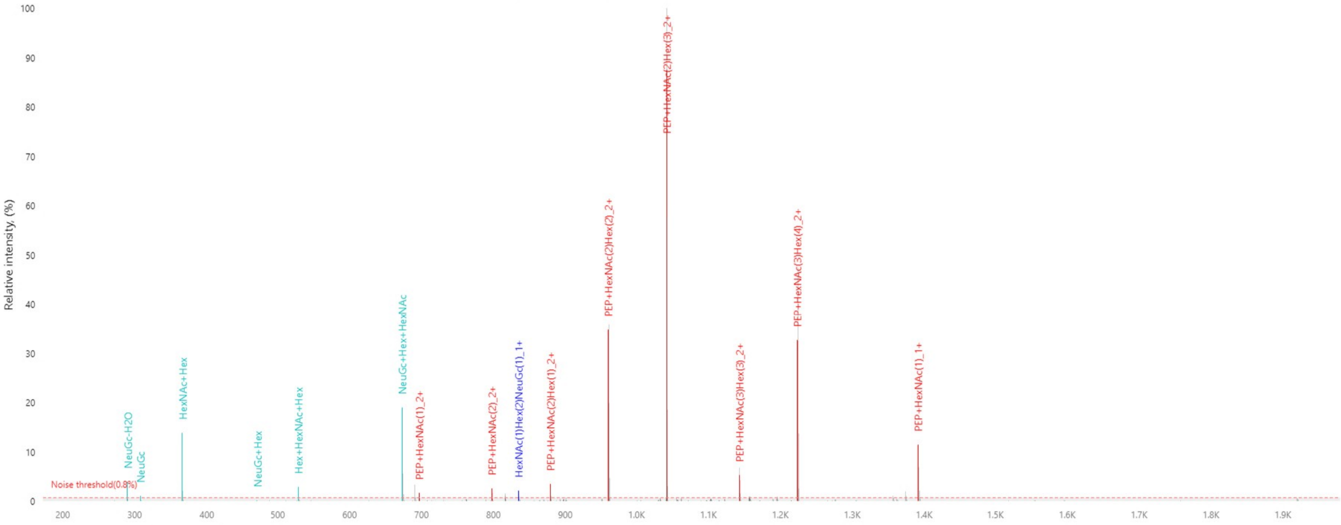

EEQYNSTYR(=PEP)_4_3_0_1_0, m/z:913.3596(3+), RT:32.25, HCD-score:59.53, Y-score:61.67, P-score:,
HCD-MS/MS Scan:6941, SNR=0.8, Base Peak Intensity=101769.8

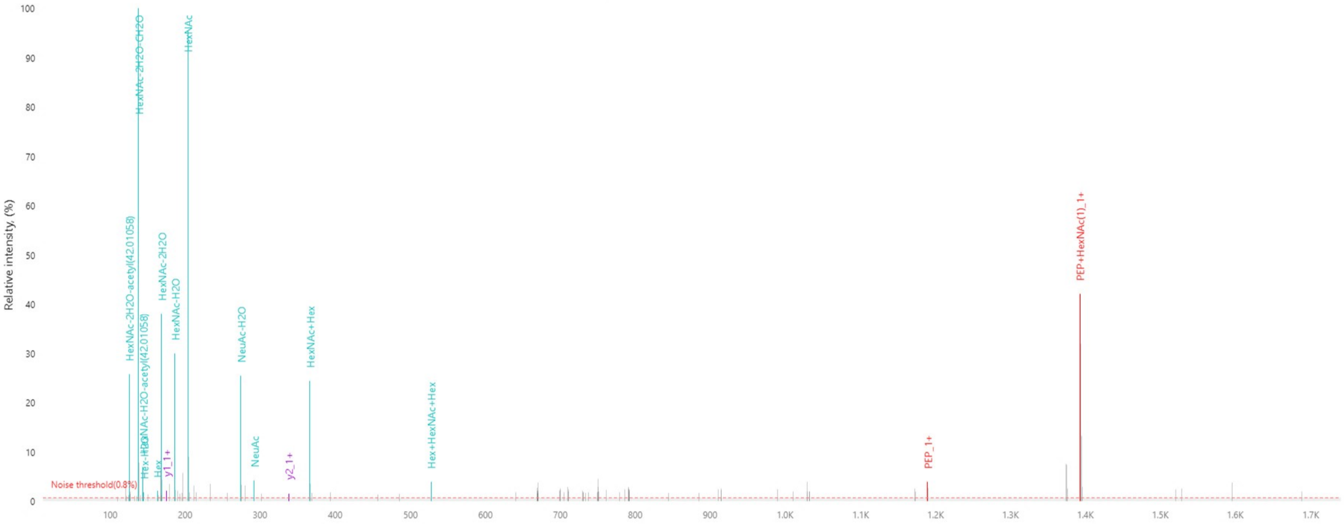

EEQYNSTYR(=PEP)_4_3_0_1_0, m/z:913.3596(3+), RT:32.26, HCD-score:59.53, Y-score:61.67, P-score:,
CID-MS/MS Scan:6944, SNR=0.8, Base Peak Intensity=85553.5

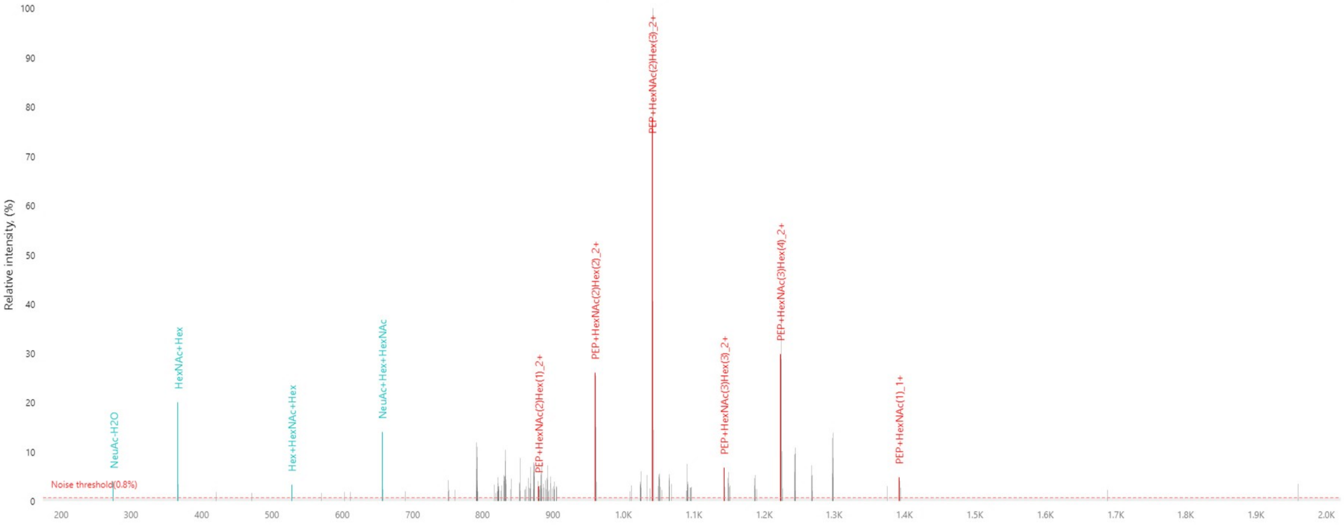

EEQYNSTYR(=PEP)_4_3_1_0_0, m/z:865.0134(3+), RT:31.73, HCD-score:88.60, Y-score:93.75, P-score:,
HCD-MS/MS Scan:6791, SNR=0.8, Base Peak Intensity=524041.9

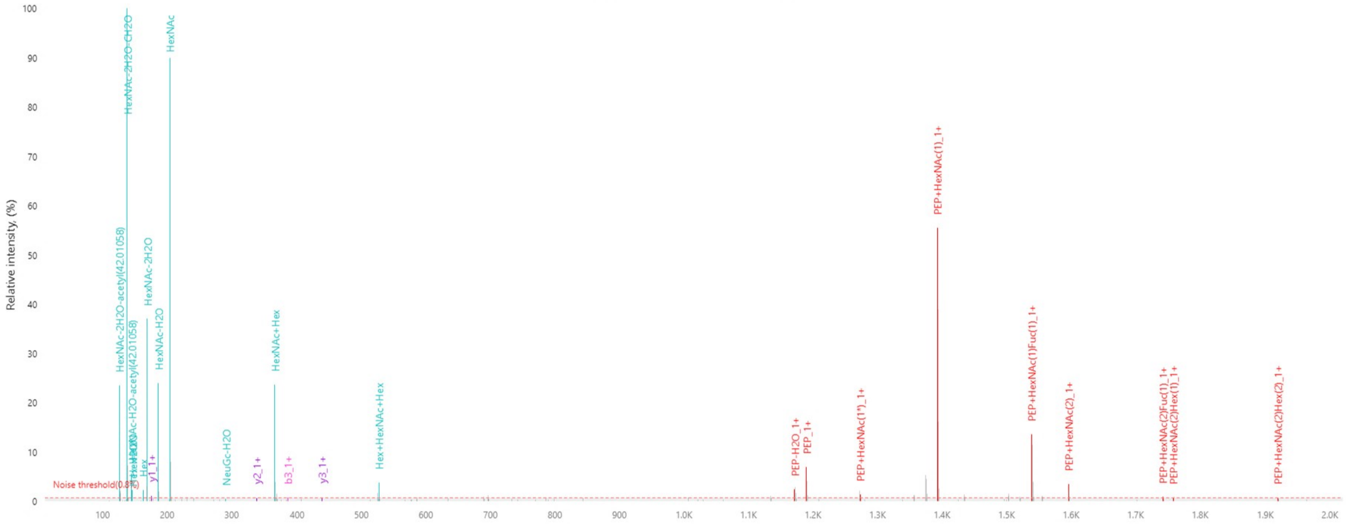

EEQYNSTYR(=PEP)_4_3_1_0_0, m/z:865.0134(3+), RT:31.74, HCD-score:88.60, Y-score:93.75, P-score:,
CID-MS/MS Scan:6793, SNR=0.8, Base Peak Intensity=787668.4

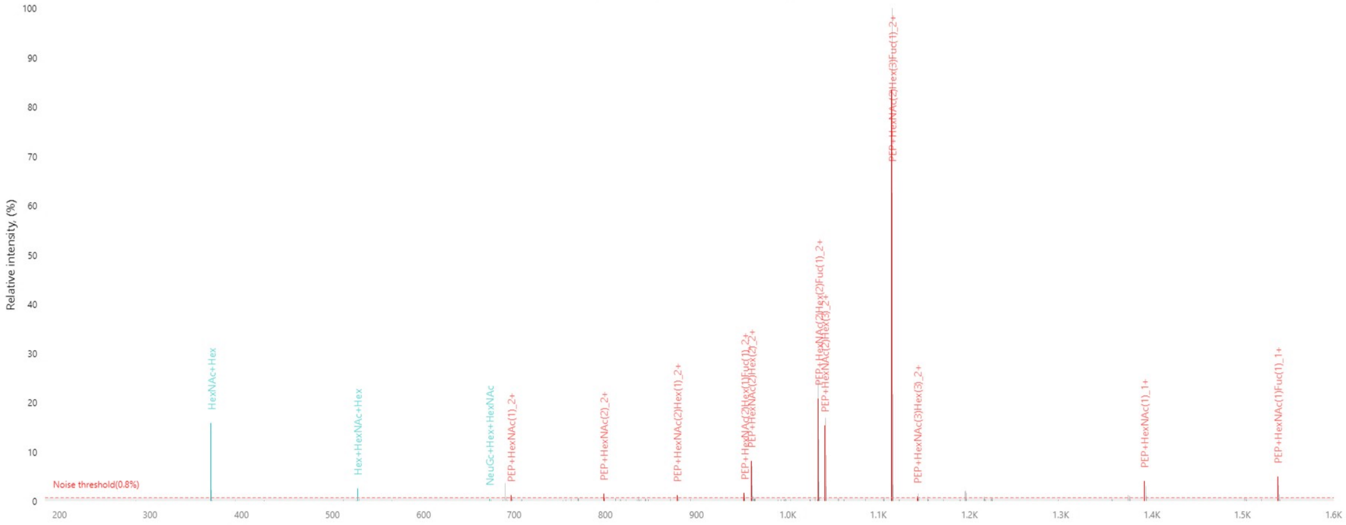

EEQYNSTYR(=PEP)_4_3_1_0_1, m/z:725.7844(4+), RT:31.69, HCD-score:94.05, Y-score:95.64, P-score:11.11,
HCD-MS/MS Scan:6781, SNR=0.8, Base Peak Intensity=338312.2

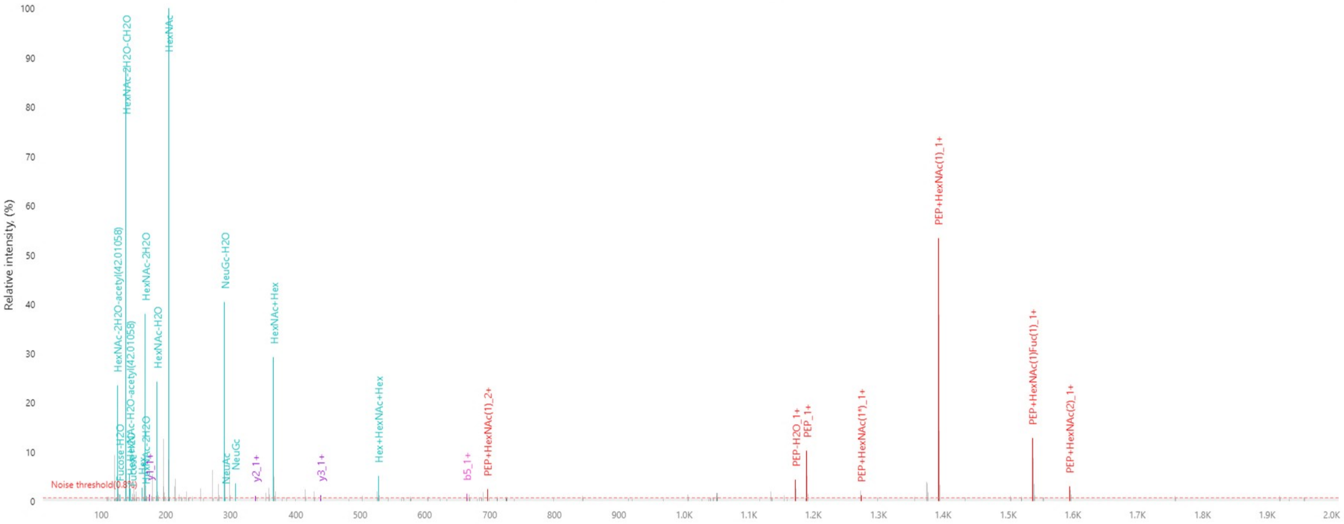

EEQYNSTYR(=PEP)_4_3_1_0_1, m/z:725.7844(4+), RT:31.71, HCD-score:94.05, Y-score:95.64, P-score:11.11,
CID-MS/MS Scan:6784, SNR=0.8, Base Peak Intensity=227232.4

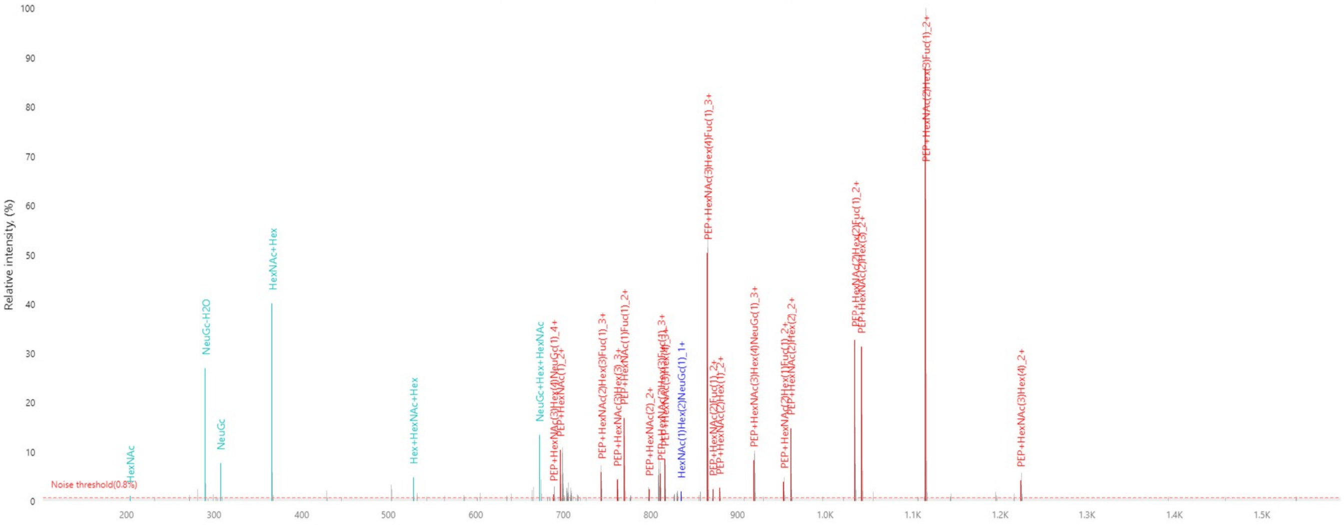

EEQYNSTYR(=PEP)_4_3_1_1_0, m/z:962.0439(3+), RT:32.48, HCD-score:88.95, Y-score:95.67, P-score:11.11,
HCD-MS/MS Scan:7014, SNR=0.8, Base Peak Intensity=847907.8

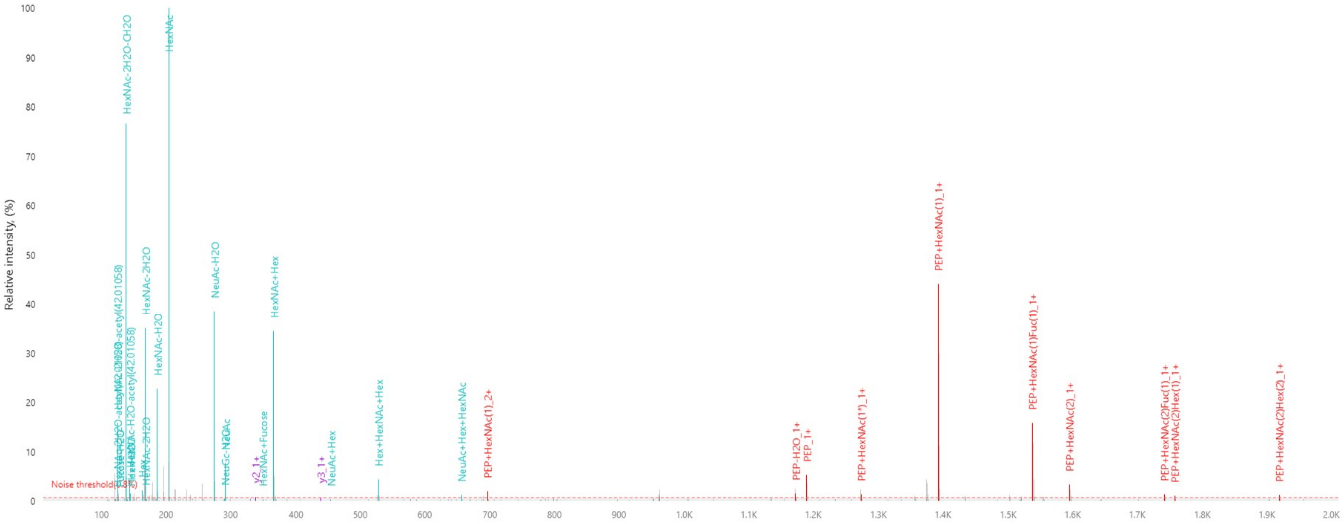

EEQYNSTYR(=PEP)_4_3_1_1_0, m/z:962.0439(3+), RT:32.49, HCD-score:88.95, Y-score:95.67, P-score:11.11,
CID-MS/MS Scan:7016, SNR=0.8, Base Peak Intensity=916357.4

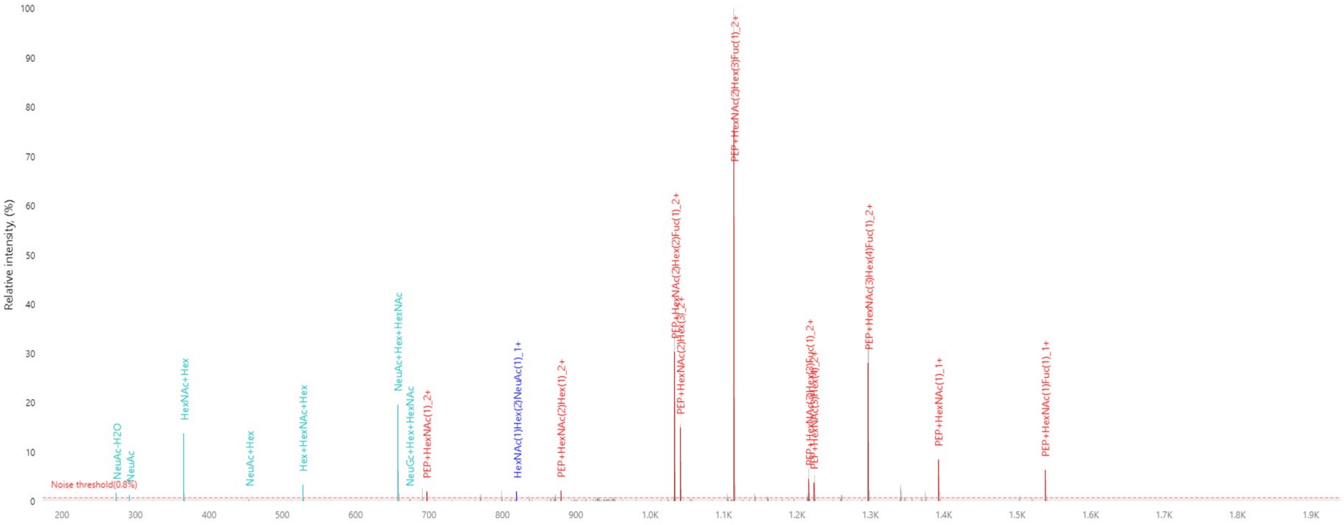

EEQYNSTYR(=PEP)_4_3_2_0_0, m/z:913.6967(3+), RT:24.07, HCD-score:82.68, Y-score:76.09, P-score:0.00,
HCD-MS/MS Scan:4563, SNR=0.8, Base Peak Intensity=519620.2

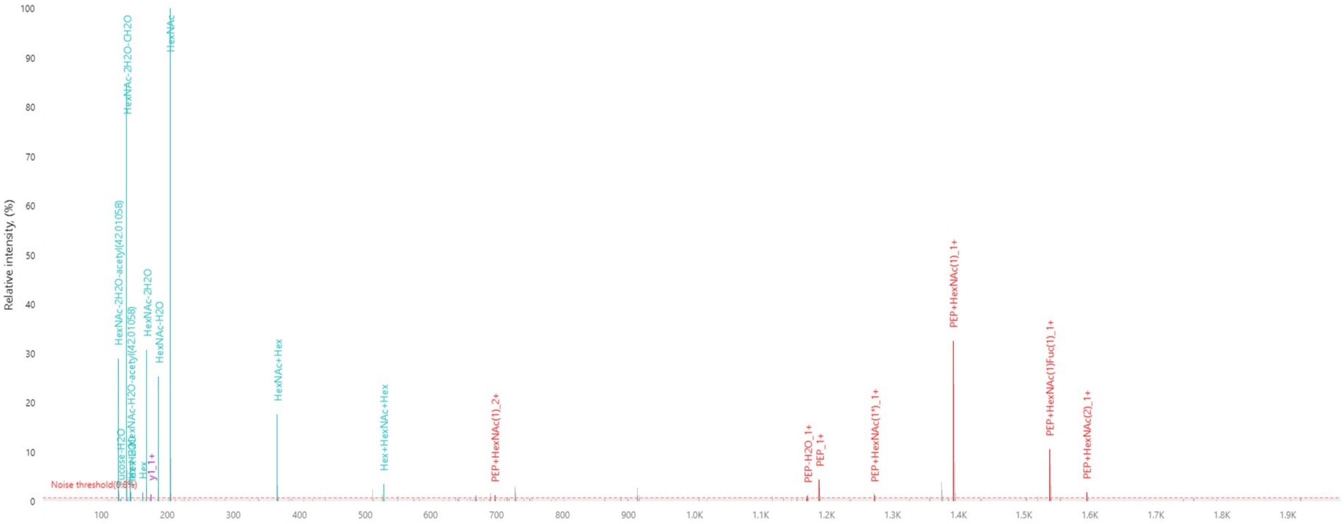

EEQYNSTYR(=PEP)_4_3_2_0_0, m/z:913.6967(3+), RT:24.09, HCD-score:82.68, Y-score:76.09, P-score:0.00,
CID-MS/MS Scan:4566, SNR=0.8, Base Peak Intensity=369961.8

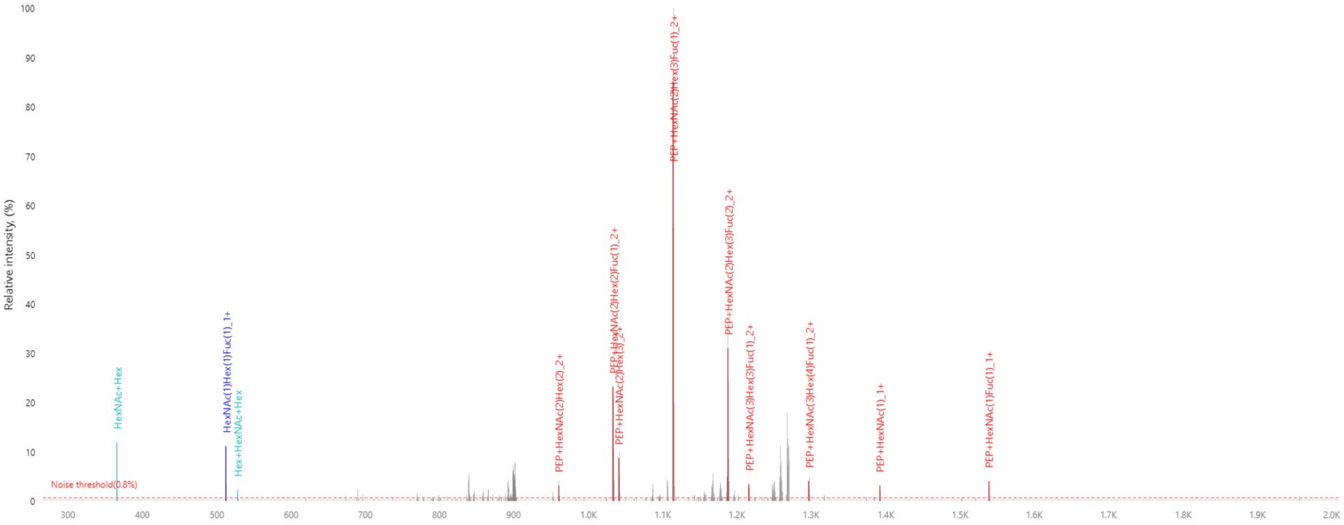

EEQYNSTYR(=PEP)_4_4_0_0_0, m/z:884.0182(3+), RT:23.54, HCD-score:95.34, Y-score:98.15, P-score:33.33,
HCD-MS/MS Scan:4411, SNR=0.8, Base Peak Intensity=7543358.5

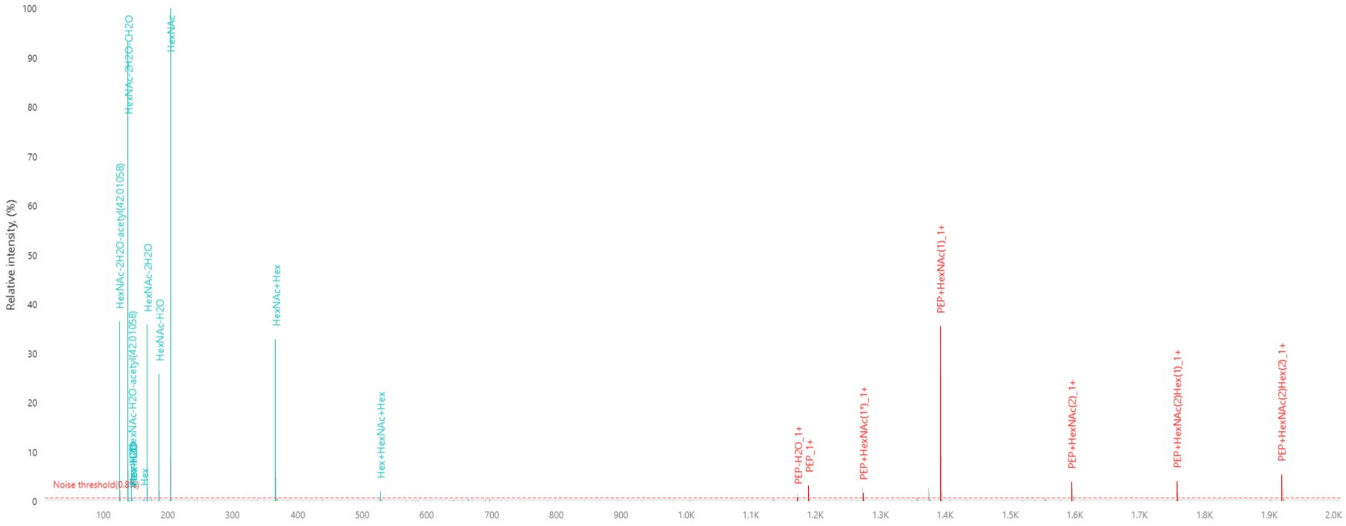

EEQYNSTYR(=PEP)_4_4_0_0_0, m/z:884.0182(3+), RT:23.55, HCD-score:95.34, Y-score:98.15, P-score:33.33,
CID-MS/MS Scan:4413, SNR=0.8, Base Peak Intensity=7944512.5

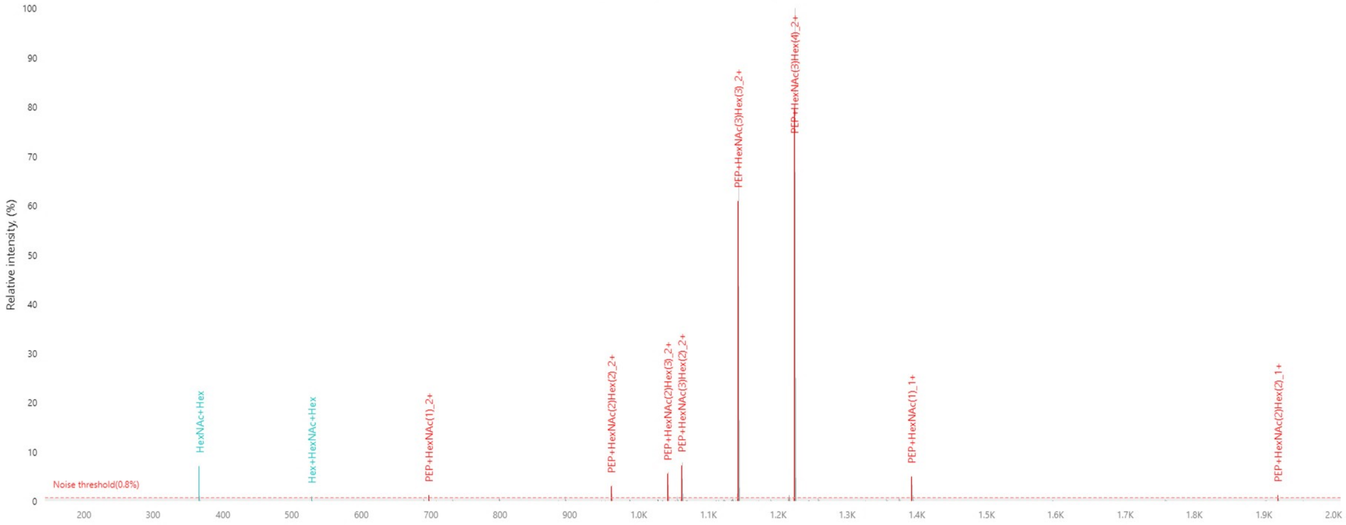

EEQYNSTYR(=PEP)_4_4_0_0_1, m/z:986.3828(3+), RT:32.21, HCD-score:57.09, Y-score:78.79, P-score:11.11,
HCD-MS/MS Scan:6932, SNR=0.8, Base Peak Intensity=734136.6

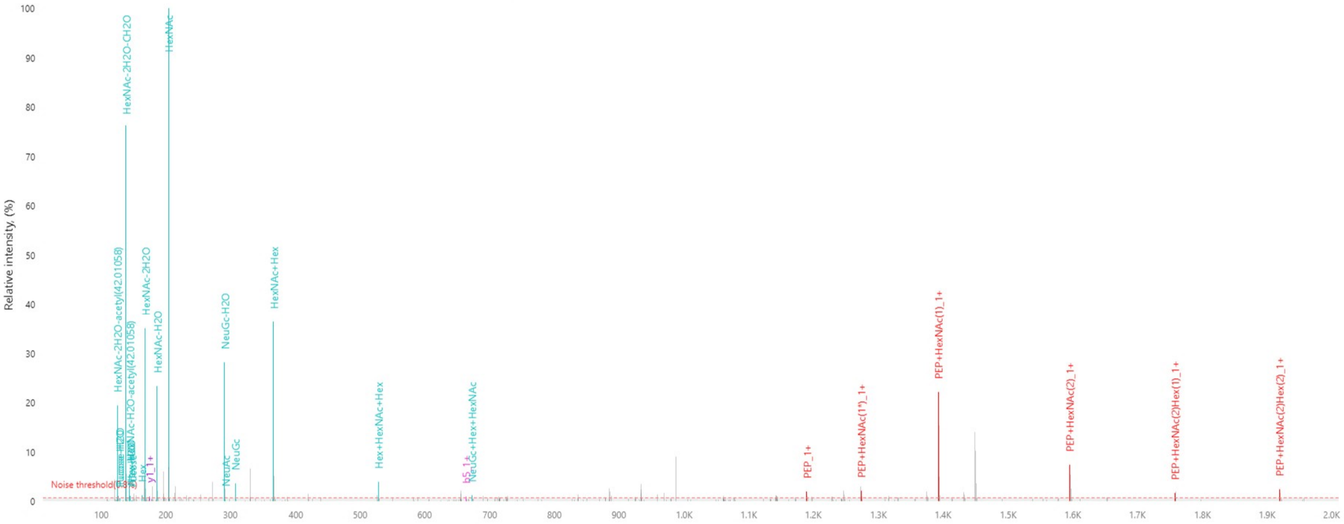

EEQYNSTYR(=PEP)_4_4_0_0_1, m/z:986.3828(3+), RT:32.22, HCD-score:57.09, Y-score:78.79, P-score:11.11,
CID-MS/MS Scan:6934, SNR=0.8, Base Peak Intensity=637626.9

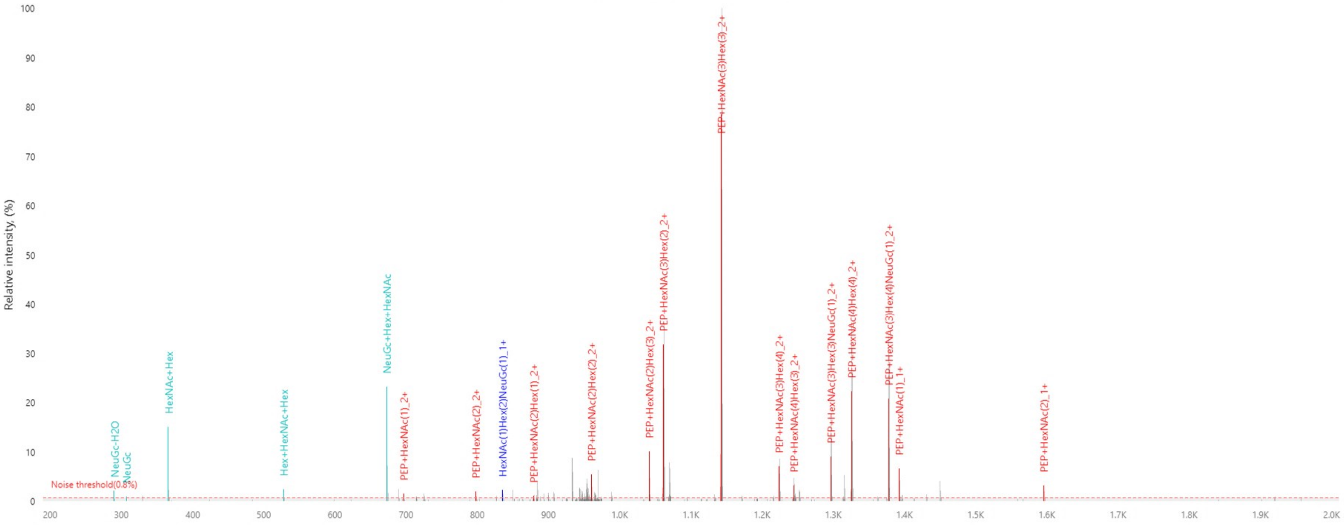

EEQYNSTYR(=PEP)_4_4_1_0_0, m/z:932.7062(3+), RT:23.33, HCD-score:100.00, Y-score:100.00, P-score:44.44,
HCD-MS/MS Scan:4350, SNR=0.8, Base Peak Intensity=31800638

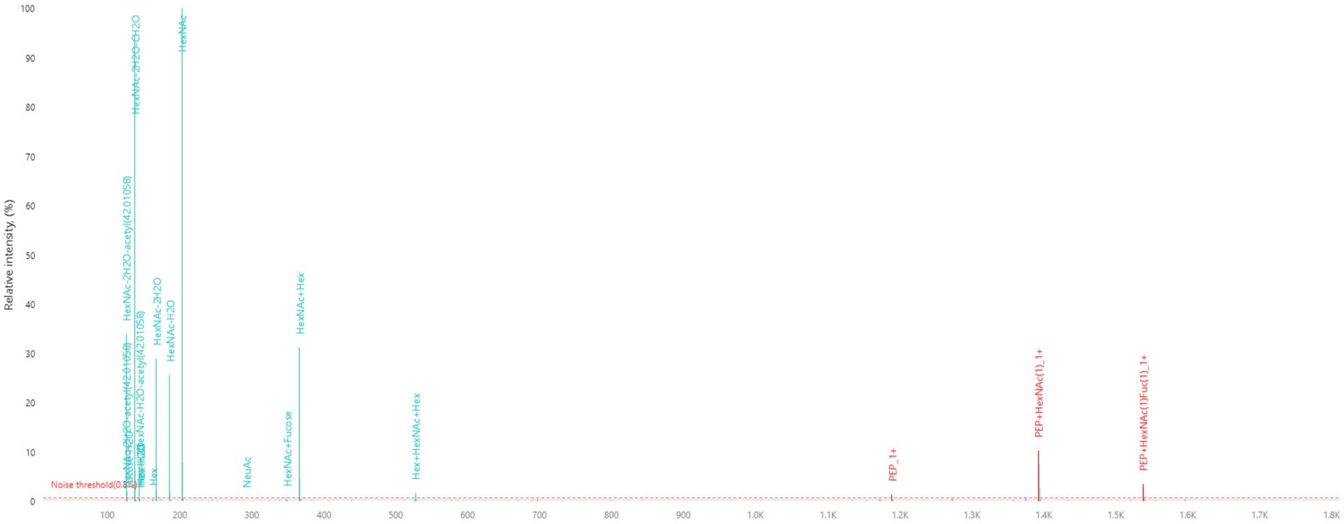

EEQYNSTYR(=PEP)_4_4_1_0_0, m/z:932.7062(3+), RT:23.34, HCD-score:100.00, Y-score:100.00, P-score:44.44,
CID-MS/MS Scan:4353, SNR=0.8, Base Peak Intensity=55842568

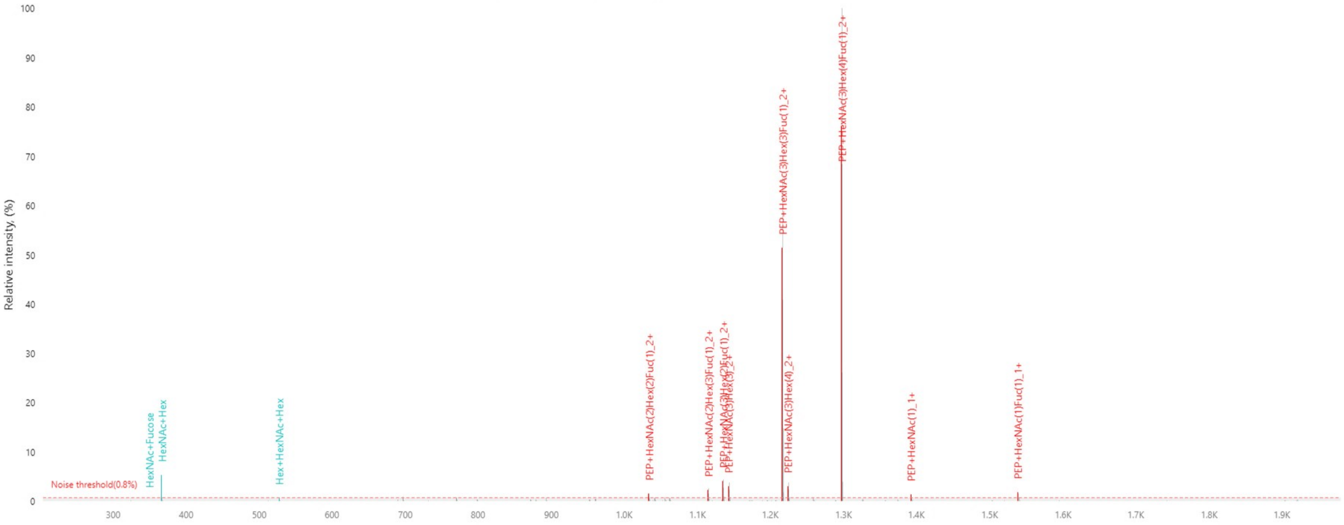

EEQYNSTYR(=PEP)_4_4_1_0_1, m/z:1035.0736(3+), RT:31.81, HCD-score:95.96, Y-score:97.68, P-score:0.00,
HCD-MS/MS Scan:6812, SNR=0.8, Base Peak Intensity=5588586.5

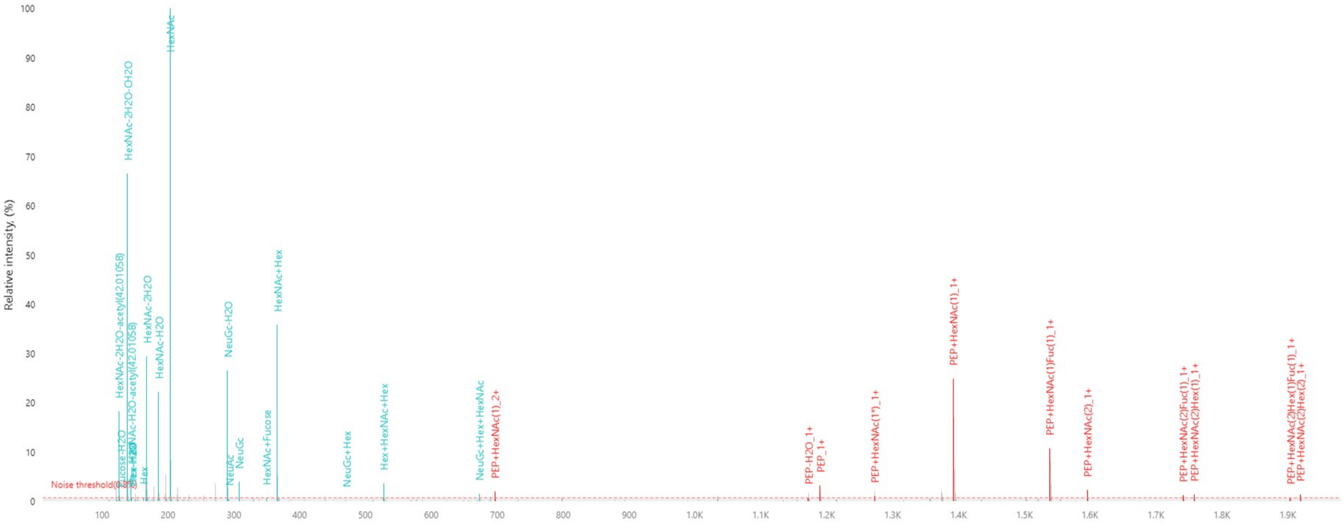

EEQYNSTYR(=PEP)_4_4_1_0_1, m/z:1035.0736(3+), RT:31.82, HCD-score:95.96, Y-score:97.68, P-score:0.00,
CID-MS/MS Scan:6814, SNR=0.8, Base Peak Intensity=2755784.5

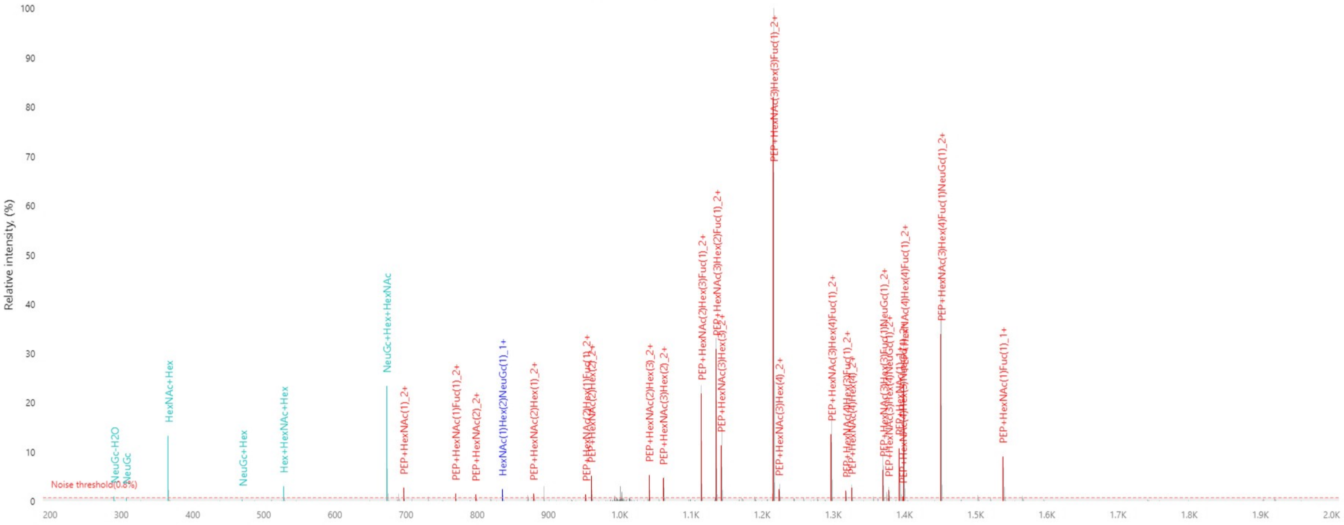

EEQYNSTYR(=PEP)_4_4_1_1_0, m/z:1029.7379(3+), RT:32.11, HCD-score:95.20, Y-score:95.27, P-score:22.22,
HCD-MS/MS Scan:7022, SNR=0.8, Base Peak Intensity=738108.9

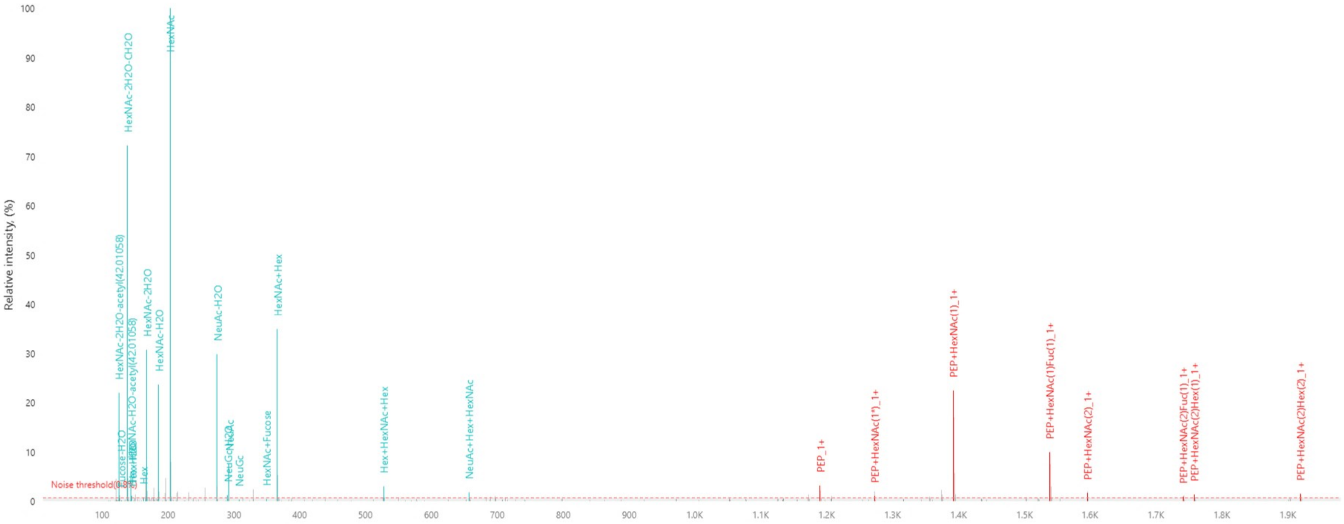

EEQYNSTYR(=PEP)_4_4_1_1_0, m/z:1029.7379(3+), RT:32.11, HCD-score:95.20, Y-score:95.27, P-score:22.22,
CID-MS/MS Scan:7024, SNR=0.8, Base Peak Intensity=447058

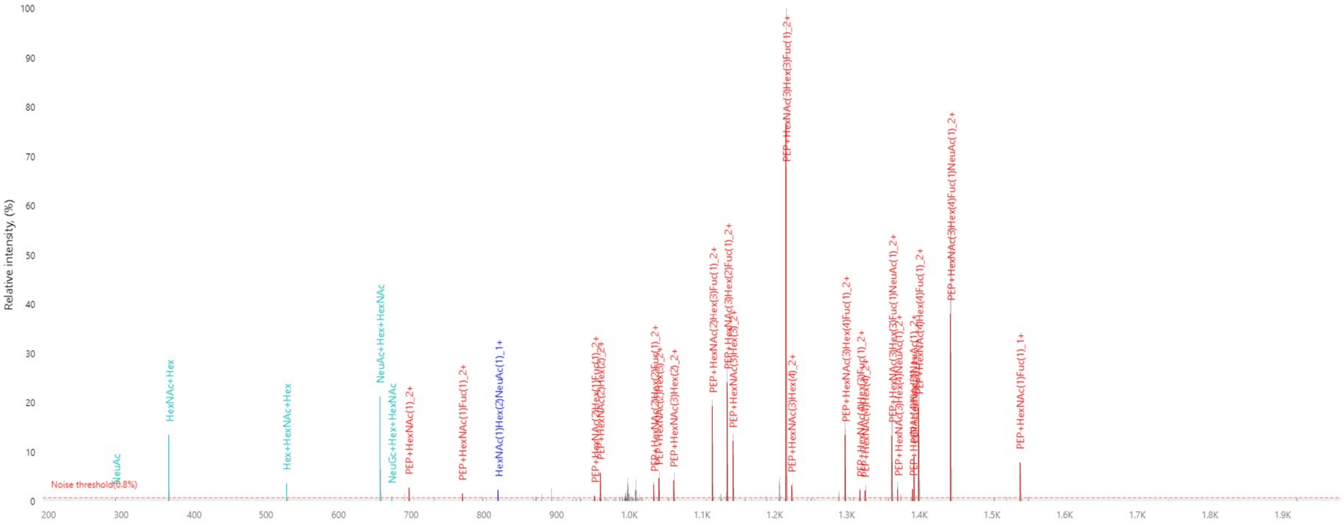

EEQYNSTYR(=PEP)_4_4_2_0_0, m/z:981.3912(3+), RT:23.72, HCD-score:90.27, Y-score:90.49, P-score:,
HCD-MS/MS Scan:4593, SNR=0.8, Base Peak Intensity=485122.2

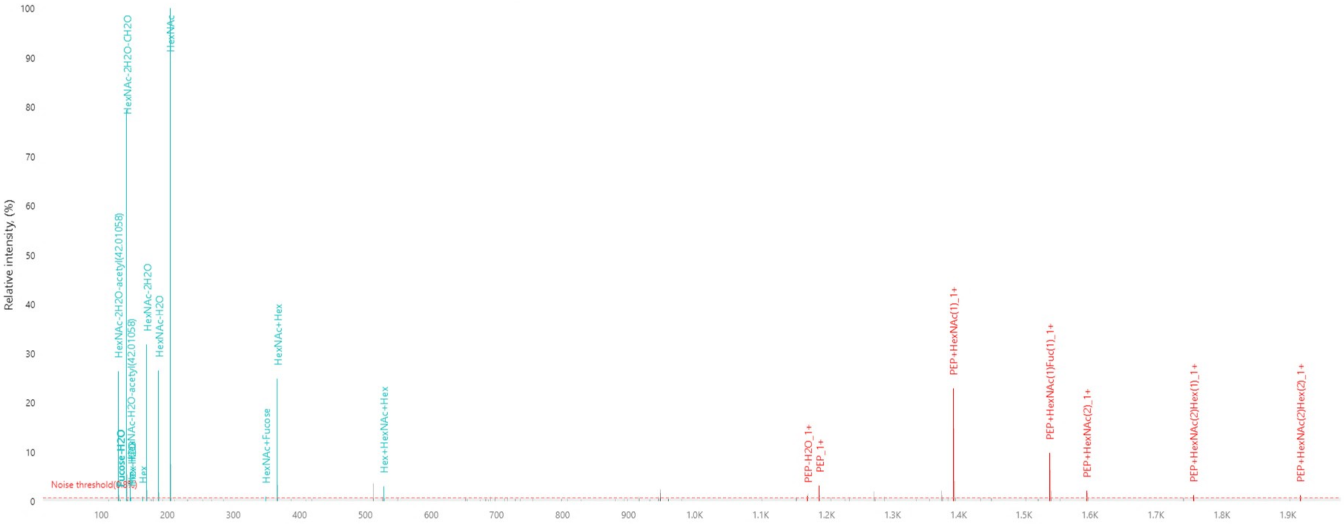

EEQYNSTYR(=PEP)_4_4_2_0_0, m/z:981.3912(3+), RT:23.74, HCD-score:90.27, Y-score:90.49, P-score:,
CID-MS/MS Scan:4596, SNR=0.8, Base Peak Intensity=337234.5

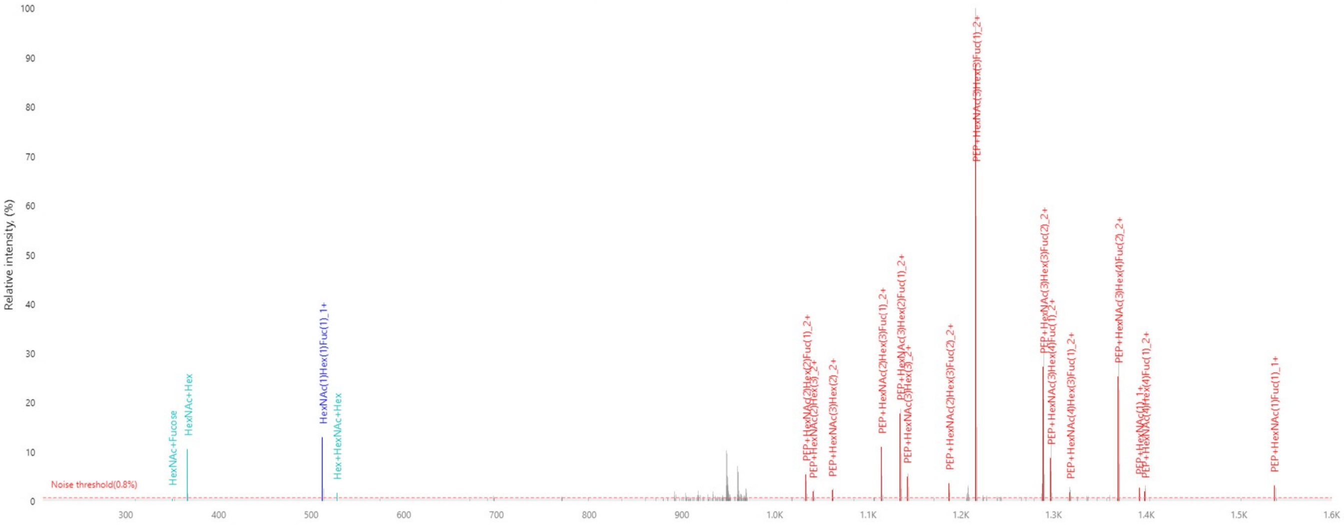

EEQYNSTYR(=PEP)_4_5_0_0_0, m/z:951.7117(3+), RT:23.96, HCD-score:26.73, Y-score:71.11, P-score:11.11,
HCD-MS/MS Scan:4529, SNR=0.8, Base Peak Intensity=1951935

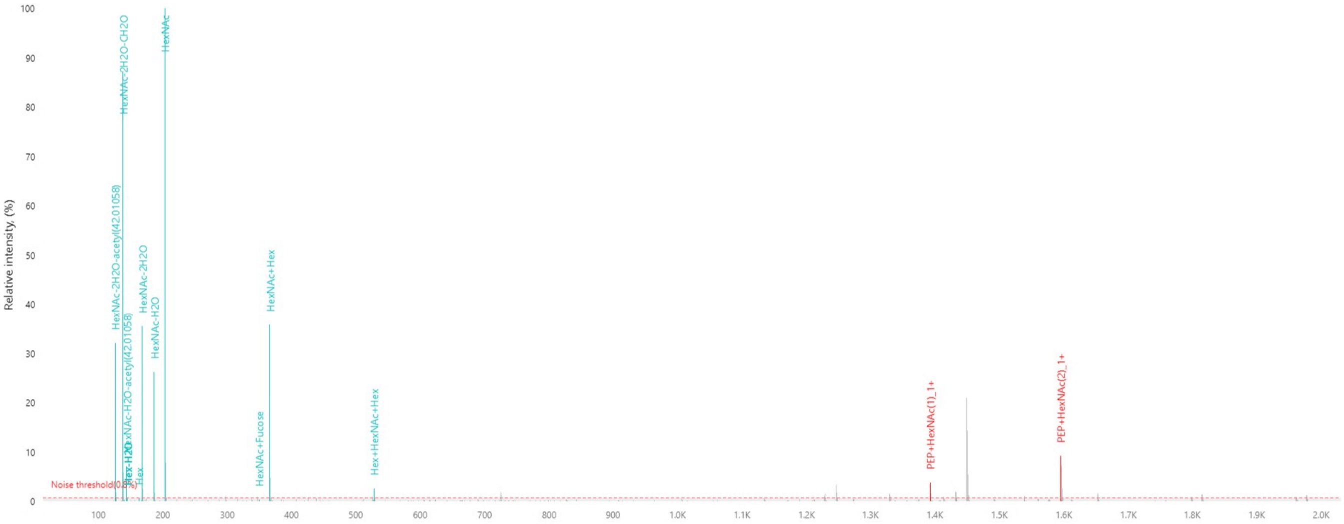

EEQYNSTYR(=PEP)_4_5_0_0_0, m/z:951.7117(3+), RT:23.97, HCD-score:26.73, Y-score:71.11, P-score:11.11,
CID-MS/MS Scan:4532, SNR=0.8, Base Peak Intensity=1795418.8

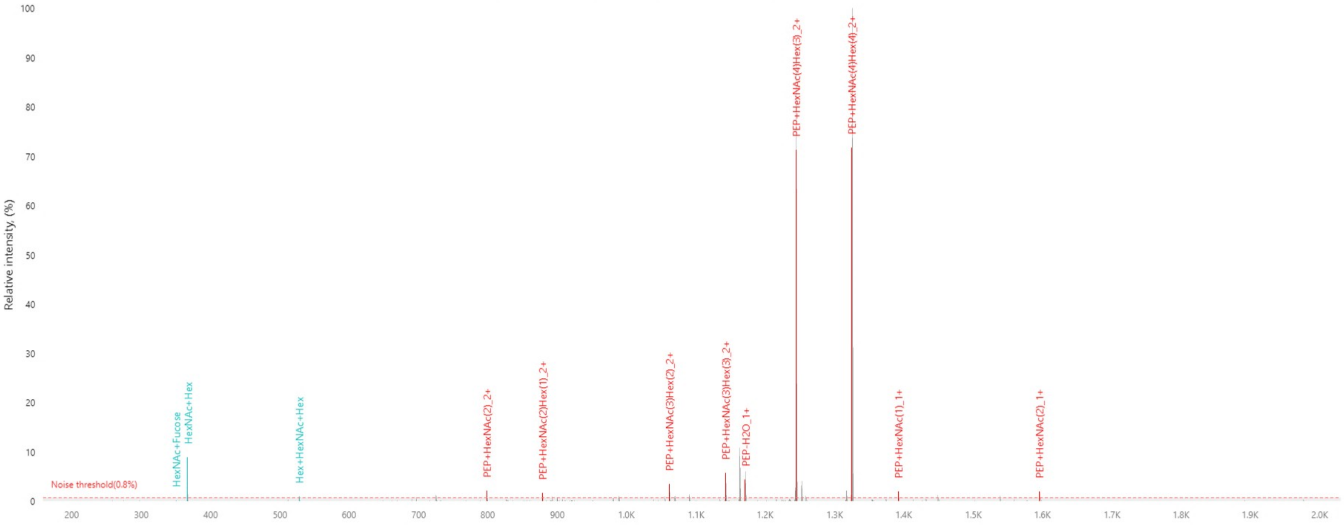

EEQYNSTYR(=PEP)_4_5_1_0_0, m/z:1000.3971(3+), RT:23.95, HCD-score:95.96, Y-score:97.79, P-score:11.11,
HCD-MS/MS Scan:4618, SNR=0.8, Base Peak Intensity=769205.9

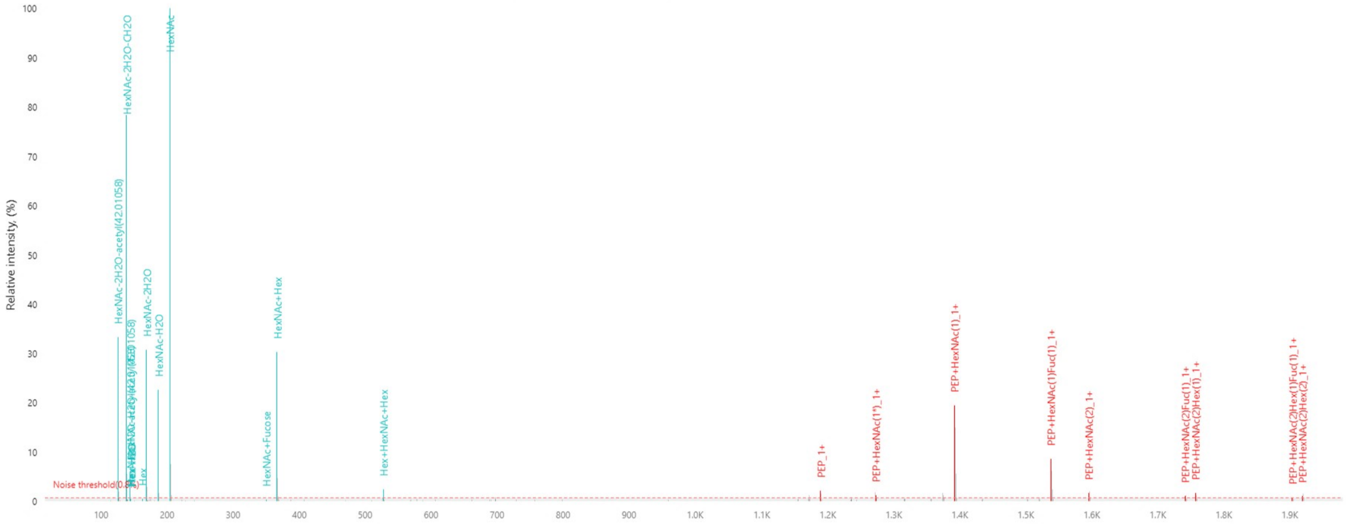

EEQYNSTYR(=PEP)_4_5_1_0_0, m/z:1000.3971(3+), RT:23.96, HCD-score:95.96, Y-score:97.79, P-score:11.11,
CID-MS/MS Scan:4621, SNR=0.8, Base Peak Intensity=605610.8

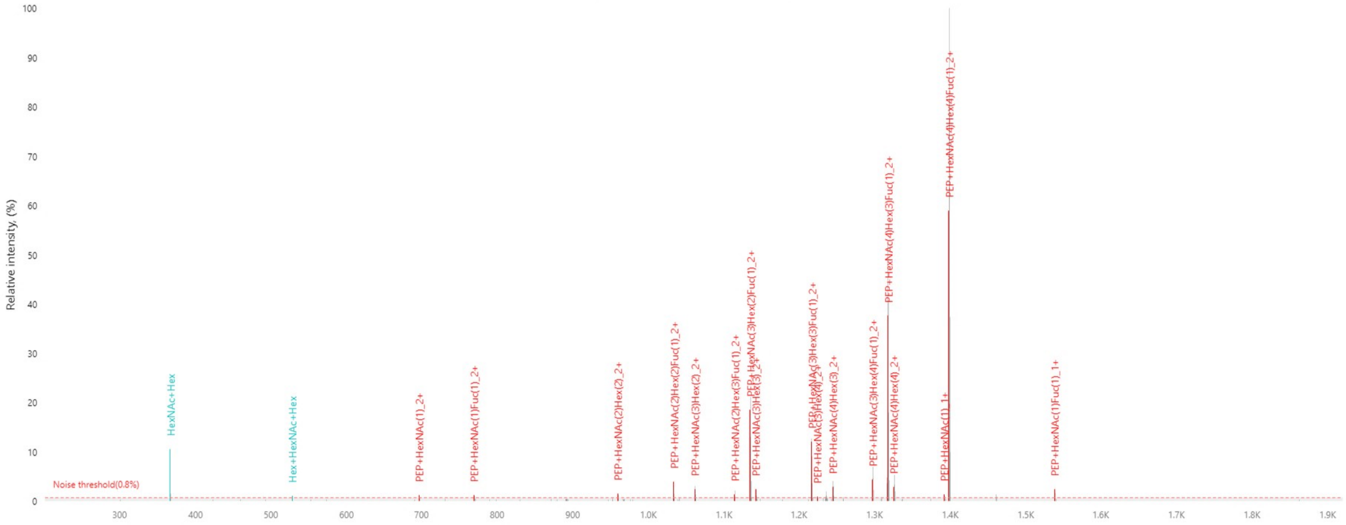

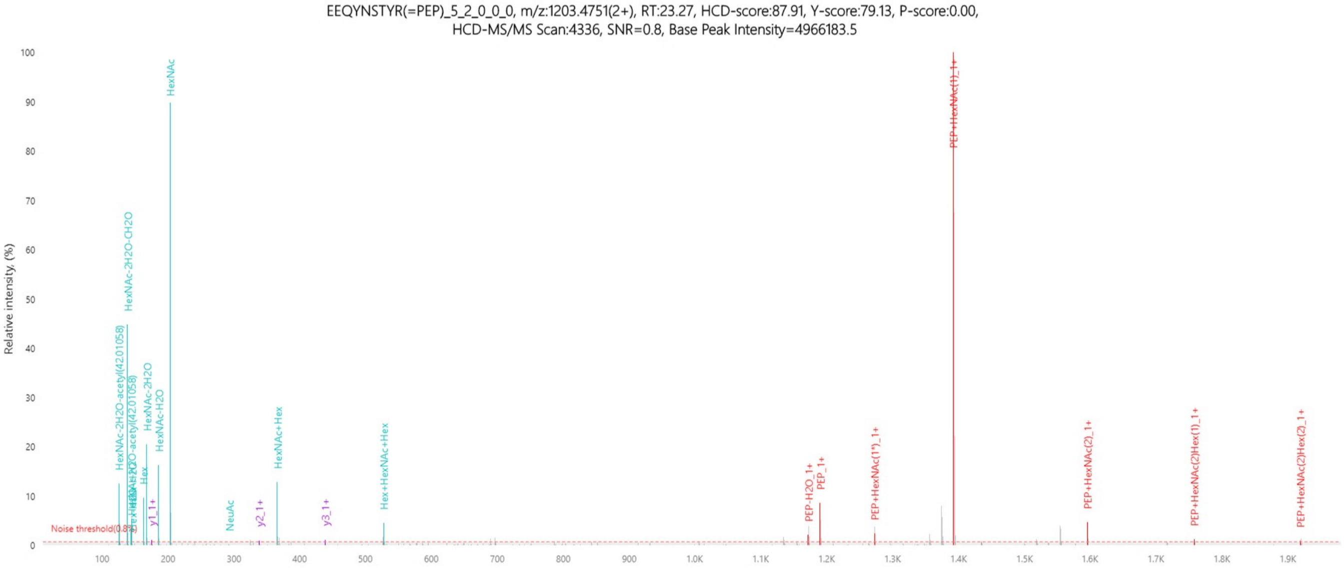
EEQYNSTYR(=PEP)_5_2_0_0_0, m/z:1203.4751(2+), RT:23.27, HCD-score:87.91, Y-score:79.13, P-score:0.00,
HCD-MS/MS Scan:4336, SNR=0.8, Base Peak Intensity=4966183.5
Relative intensity, (%)
Noise threshold(0.8%)
HexNAc
HexNAc-2H2O-CH2O
HexNAc-2H2O-acetyl(42.01058)
HexNAc-2H2O
HexNAc-H2O
Hex
y1_1+
NeuAc
y2_1+
HexNAc+Hex
y3_1+
Hex+HexNAc+Hex
PEP-H2O_1+
PEP_1+
PEP+HexNAc(1*)_1+
PEP+HexNAc(1)_1+
PEP+HexNAc(2)_1+
PEP+HexNAc(2)Hex(1)_1+
PEP+HexNAc(2)Hex(2)_1+

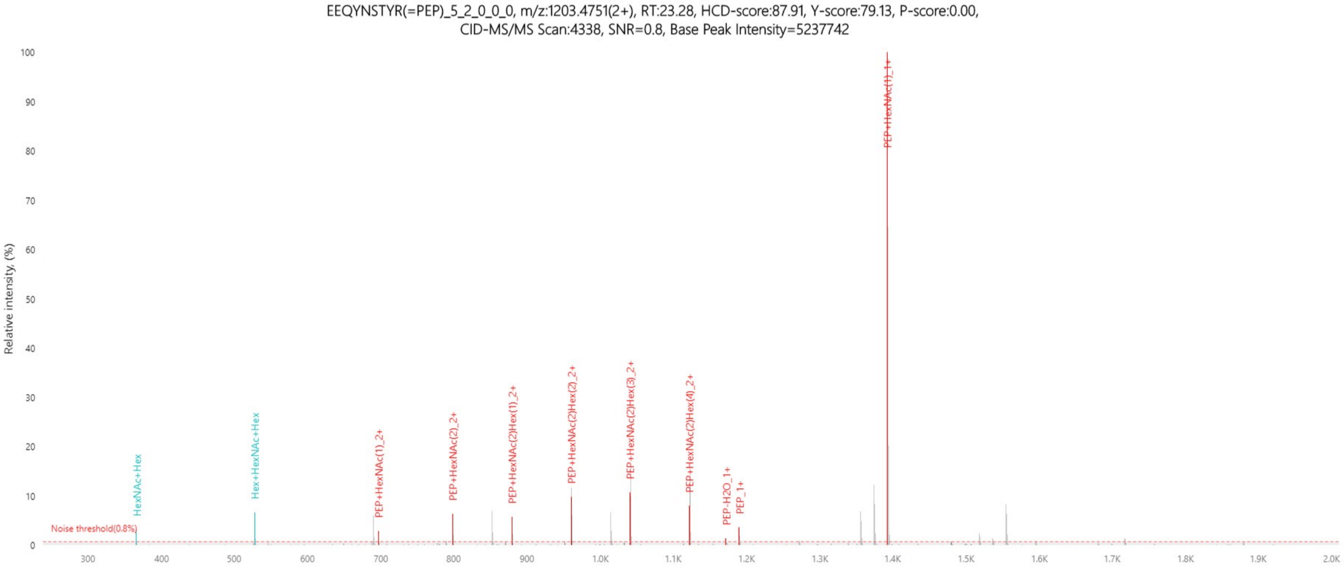
EEQYNSTYR(=PEP)_5_2_0_0_0, m/z:1203.4751(2+), RT:23.28, HCD-score:87.91, Y-score:79.13, P-score:0.00,
CID-MS/MS Scan:4338, SNR=0.8, Base Peak Intensity=5237742
Relative intensity, (%)
Noise threshold(0.8%)
HexNAc+Hex
Hex+HexNAc+Hex
PEP+HexNAc(1)_2+
PEP+HexNAc(2)_2+
PEP+HexNAc(2)Hex(1)_2+
PEP+HexNAc(2)Hex(2)_2+
PEP+HexNAc(2)Hex(3)_2+
PEP+HexNAc(2)Hex(4)_2+
PEP-H2O_1+
PEP_1+
PEP+HexNAc(1)_1+

EEQYNSTYR(=PEP)_5_2_1_0_0, m/z:1276.5023(2+), RT:22.65, HCD-score:86.82, Y-score:79.37, P-score:0.00,
HCD-MS/MS Scan:4255, SNR=0.8, Base Peak Intensity=2003005.8

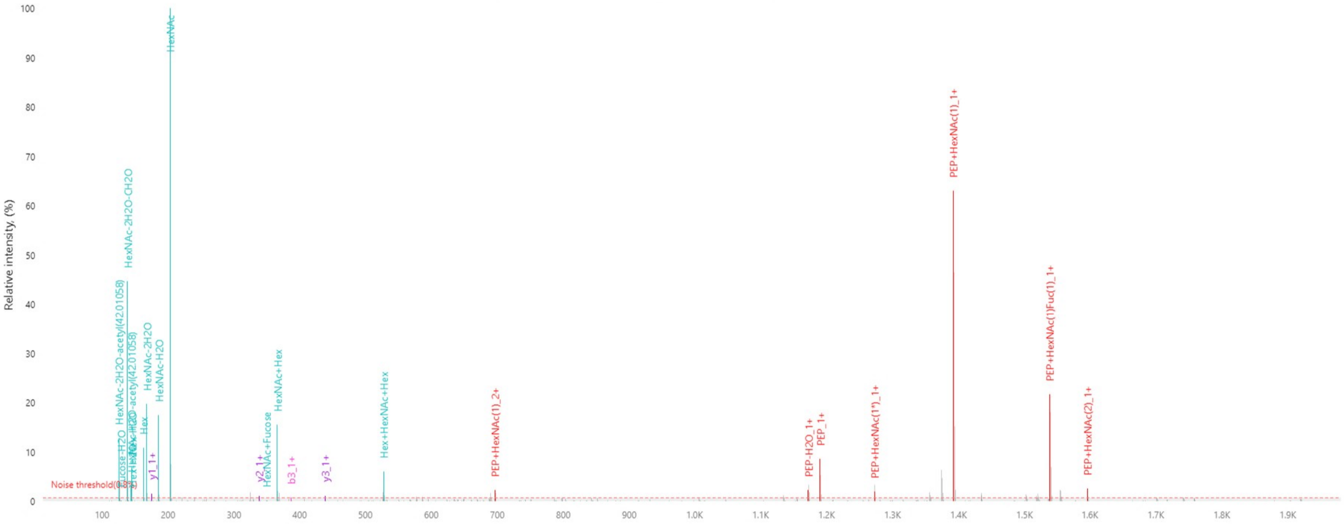

EEQYNSTYR(=PEP)_5_2_1_0_0, m/z:1276.5023(2+), RT:22.66, HCD-score:86.82, Y-score:79.37, P-score:0.00,
CID-MS/MS Scan:4258, SNR=0.8, Base Peak Intensity=2009389.5

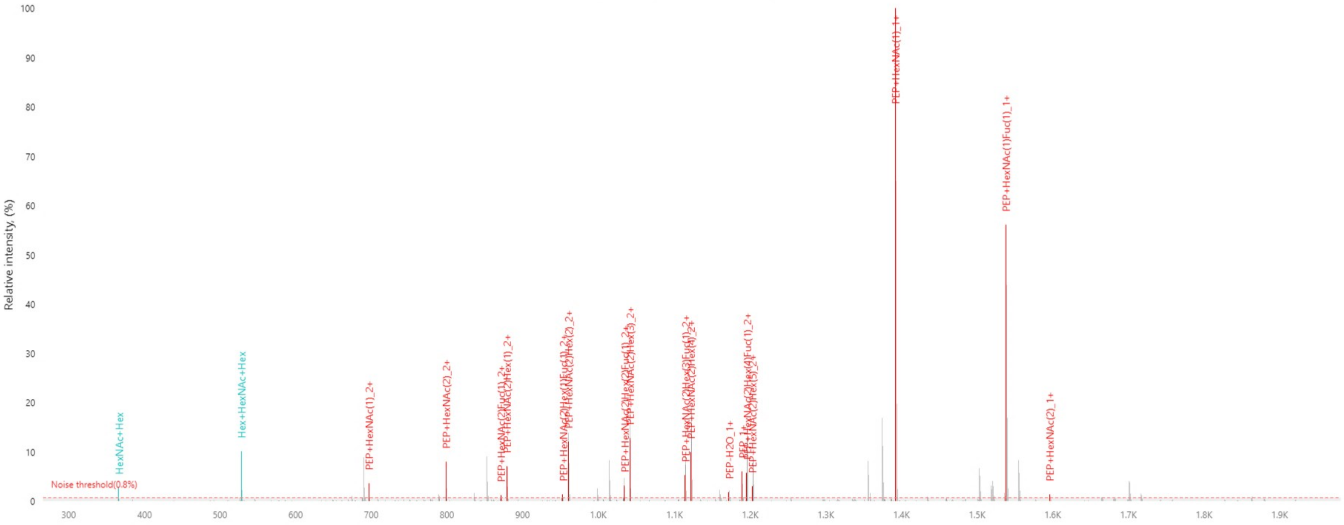

EEQYNSTYR(=PEP)_5_3_0_0_0, m/z:870.3497(3+), RT:22.85, HCD-score:90.71, Y-score:97.21, P-score:0.00,
HCD-MS/MS Scan:4218, SNR=0.8, Base Peak Intensity=12985602

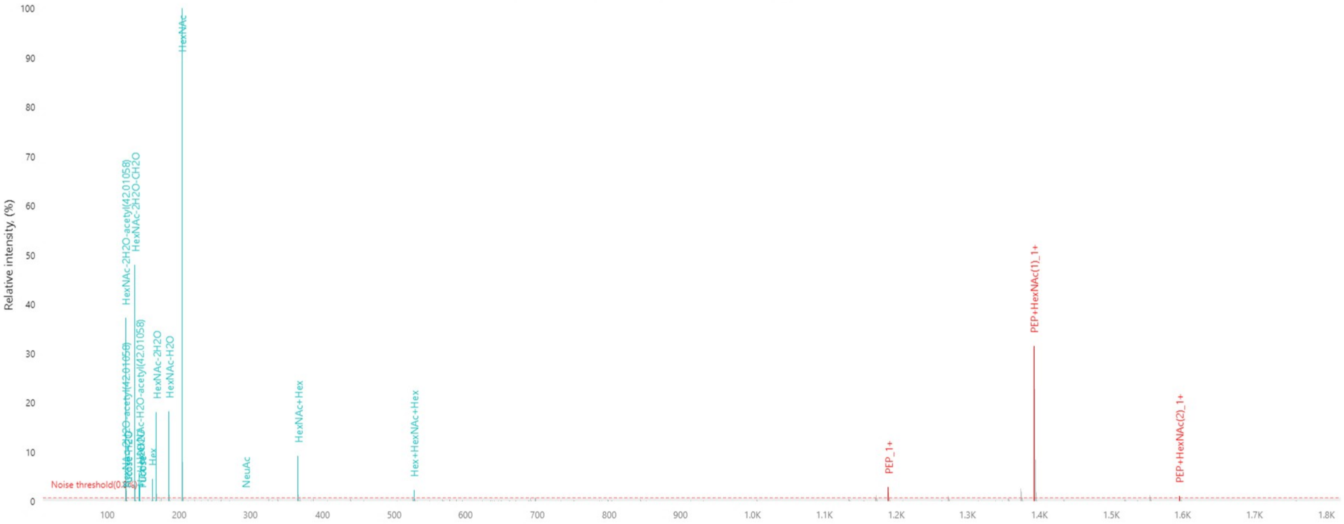

EEQYNSTYR(=PEP)_5_3_0_0_0, m/z:870.3497(3+), RT:22.86, HCD-score:90.71, Y-score:97.21, P-score:0.00,
CID-MS/MS Scan:4221, SNR=0.8, Base Peak Intensity=23707026

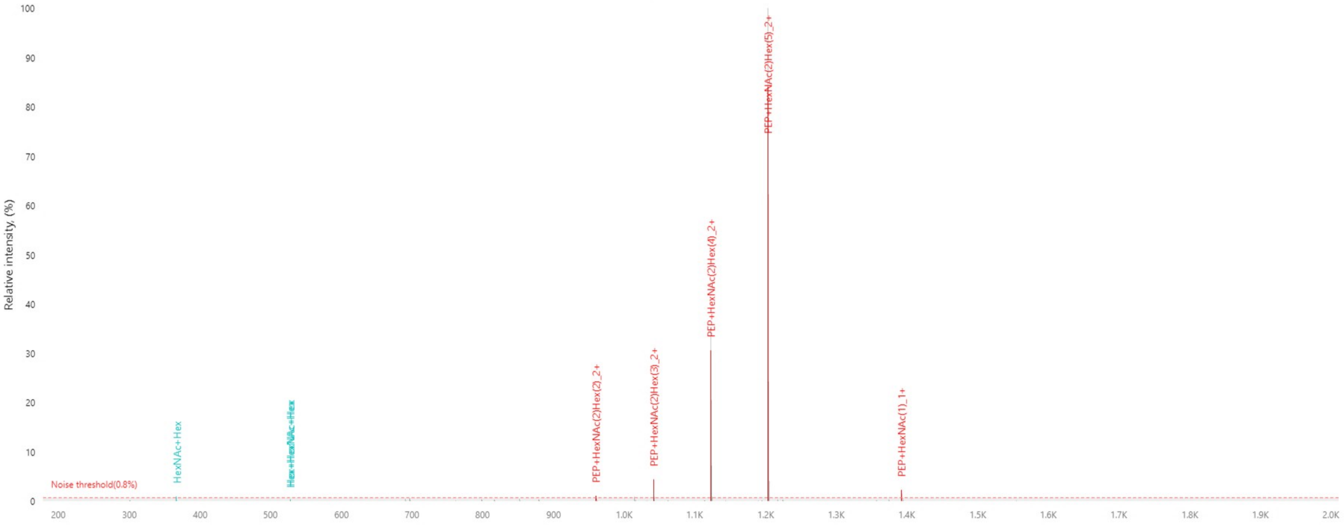

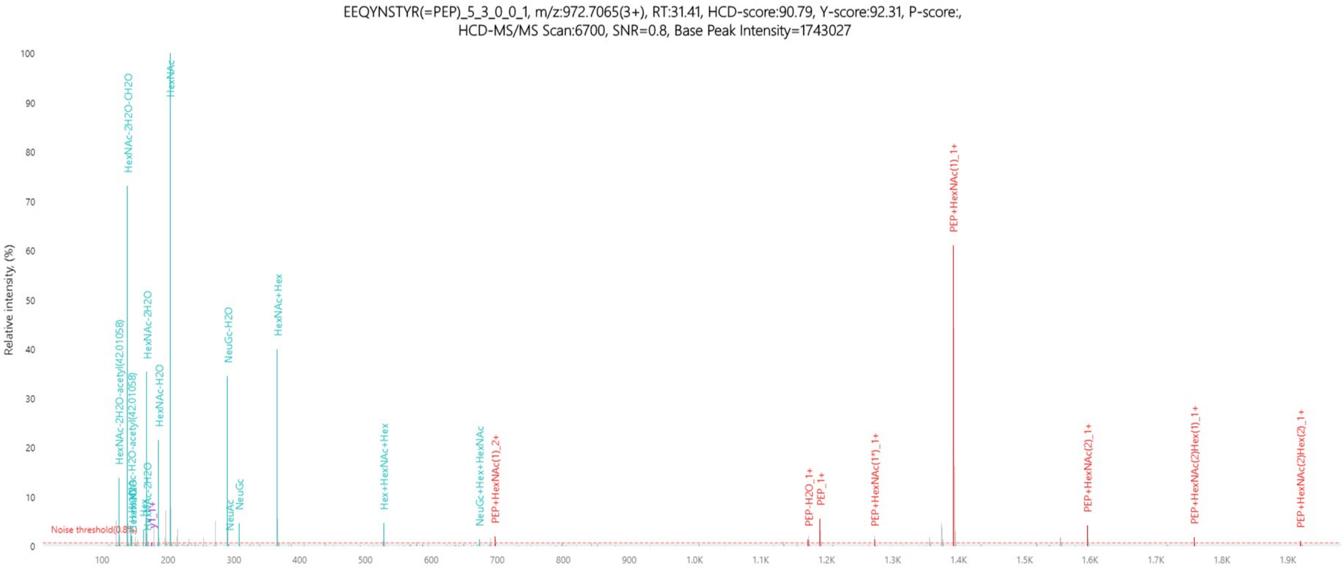
EEQYNSTYR(=PEP)_5_3_0_0_1, m/z:972.7065(3+), RT:31.41, HCD-score:90.79, Y-score:92.31, P-score:,
HCD-MS/MS Scan:6700, SNR=0.8, Base Peak Intensity=1743027
Relative intensity, (%)
HexNAc
HexNAc-2H2O-CH2O
HexNAc-2H2O
HexNAc-H2O
NeuGc-H2O
NeuAc
NeuGc
HexNAc+Hex
Hex+HexNAc+Hex
NeuGc+Hex+HexNAc
PEP+HexNAc(1)_2+
PEP-H2O_1+
PEP_1+
PEP+HexNAc(1)_1+
PEP+HexNAc(2)_1+
PEP+HexNAc(2)Hex(1)_1+
PEP+HexNAc(2)Hex(2)_1+
Noise threshold(0.8%)

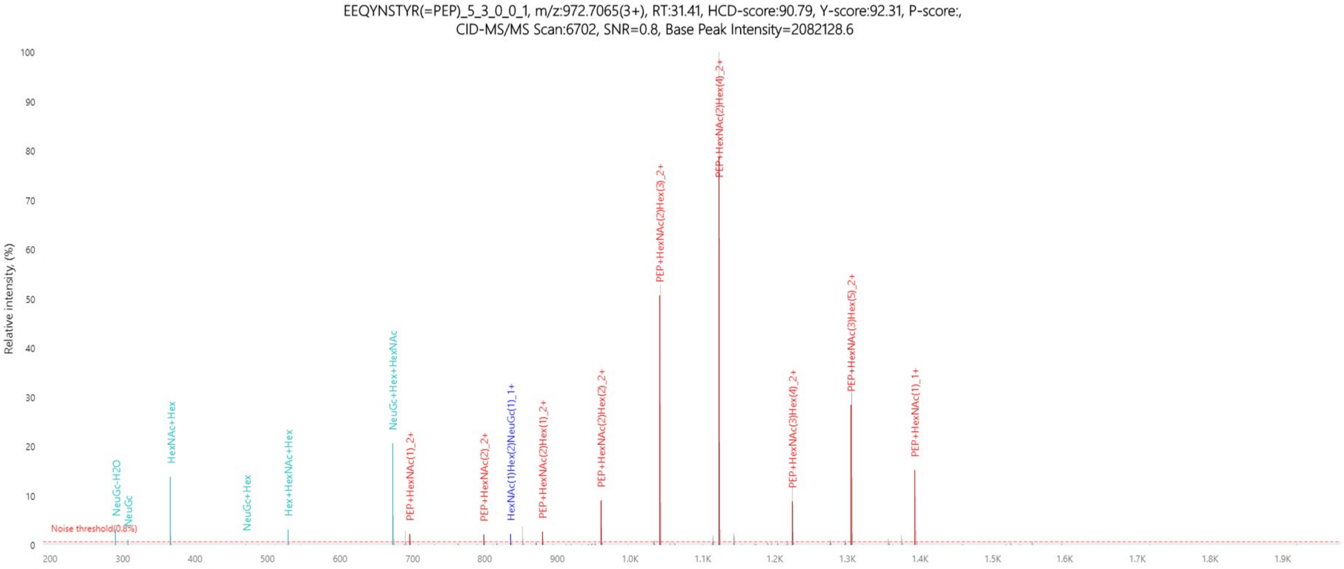
EEQYNSTYR(=PEP)_5_3_0_0_1, m/z:972.7065(3+), RT:31.41, HCD-score:90.79, Y-score:92.31, P-score:,
CID-MS/MS Scan:6702, SNR=0.8, Base Peak Intensity=2082128.6
Relative intensity, (%)
NeuGc-H2O
NeuGc
HexNAc+Hex
NeuGc+Hex
Hex+HexNAc+Hex
NeuGc+Hex+HexNAc
PEP+HexNAc(1)_2+
PEP+HexNAc(2)_2+
HexNAc(1)Hex(2)NeuGc(1)_1+
PEP+HexNAc(2)Hex(1)_2+
PEP+HexNAc(2)Hex(2)_2+
PEP+HexNAc(2)Hex(3)_2+
PEP+HexNAc(2)Hex(4)_2+
PEP+HexNAc(3)Hex(4)_2+
PEP+HexNAc(3)Hex(5)_2+
PEP+HexNAc(1)_1+
Noise threshold(0.8%)

EEQYNSTYR(=PEP)_5_3_1_0_0, m/z:1378.0426(2+), RT:23.21, HCD-score:96.82, Y-score:85.77, P-score:0.00,
HCD-MS/MS Scan:4412, SNR=0.8, Base Peak Intensity=4459029

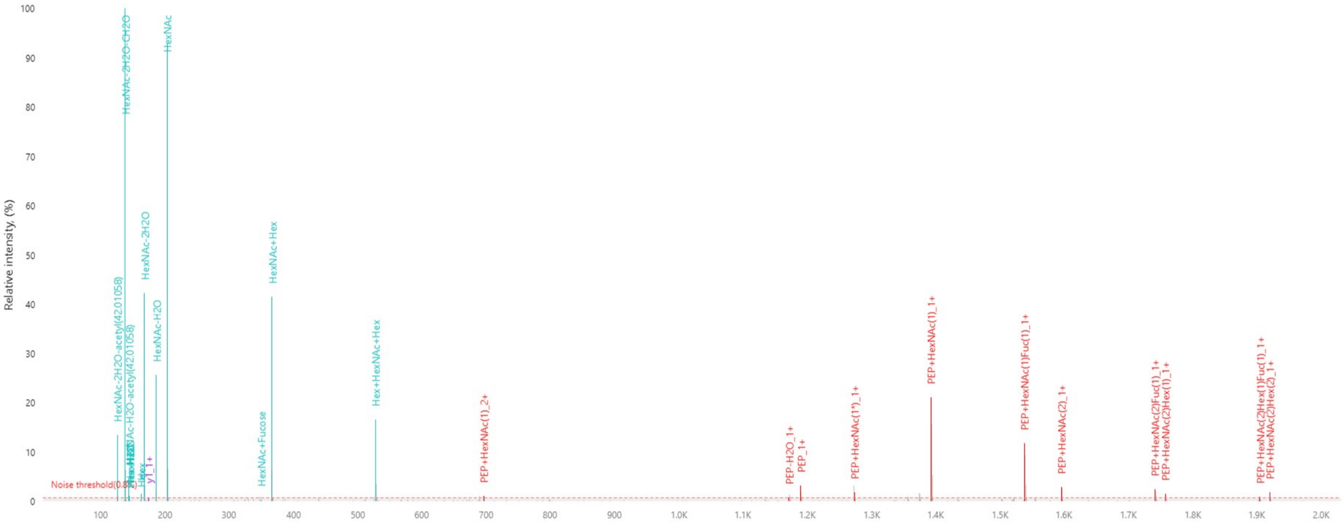

EEQYNSTYR(=PEP)_5_3_1_0_0, m/z:1378.0426(2+), RT:23.22, HCD-score:96.82, Y-score:85.77, P-score:0.00,
CID-MS/MS Scan:4414, SNR=0.8, Base Peak Intensity=1856941.8

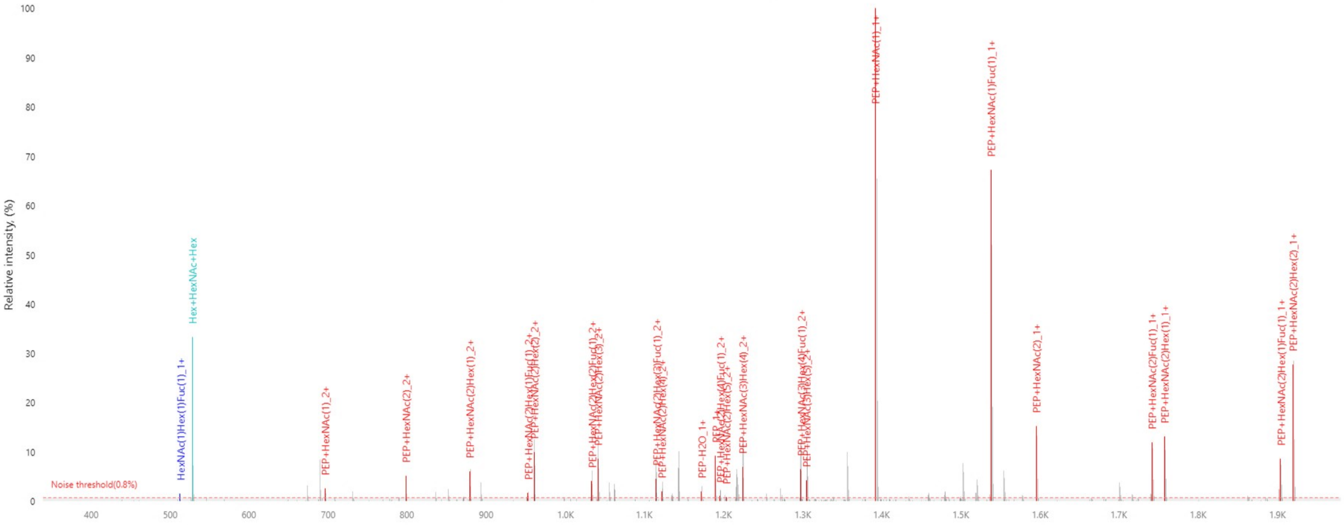

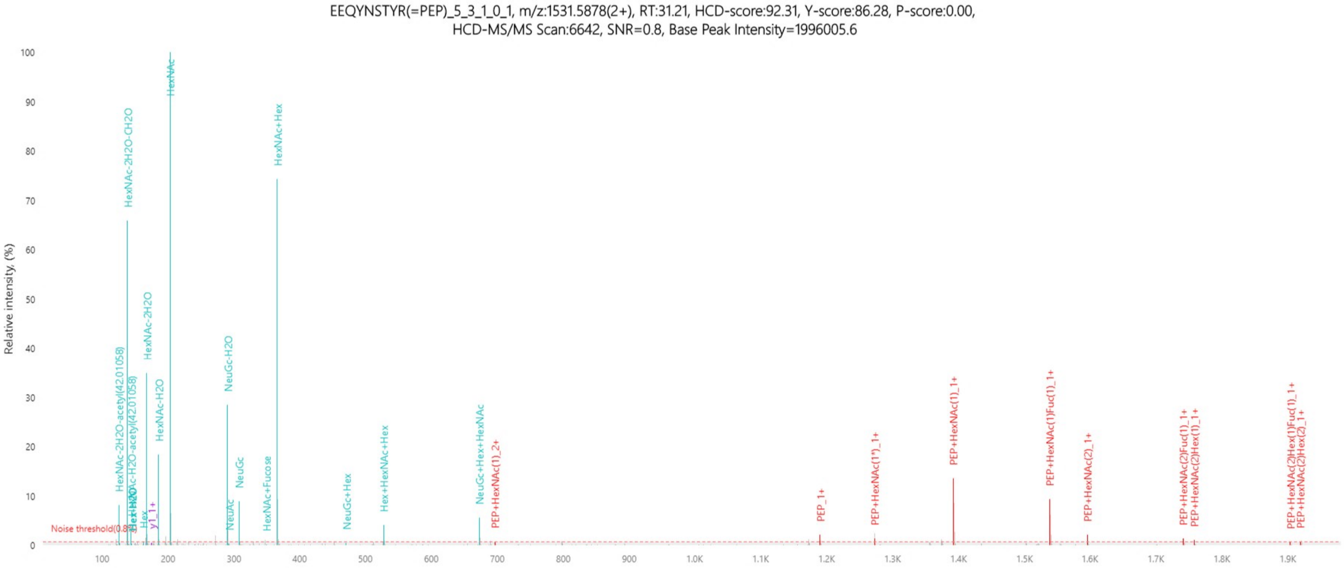

EEQYNSTYR(=PEP)_5_3_1_0_1, m/z:1531.5878(2+), RT:31.21, HCD-score:92.31, Y-score:86.28, P-score:0.00,
HCD-MS/MS Scan:6642, SNR=0.8, Base Peak Intensity=1996005.6
Relative intensity, (%)
Noise threshold(0.8%)
HexNAc
HexNAc-2H2O-CH2O
HexNAc+Hex
HexNAc-2H2O
HexNAc-H2O
NeuGc-H2O
NeuGc
NeuAc
HexNAc+Fucose
NeuGc+Hex
Hex+HexNAc+Hex
NeuGc+Hex+HexNAc
PEP+HexNAc(1)_2+
PEP_1+
PEP+HexNAc(1*)_1+
PEP+HexNAc(1)_1+
PEP+HexNAc(1)Fuc(1)_1+
PEP+HexNAc(2)_1+
PEP+HexNAc(2)Fuc(1)_1+
PEP+HexNAc(2)Hex(1)_1+
PEP+HexNAc(2)Hex(1)Fuc(1)_1+
PEP+HexNAc(2)Hex(2)_1+

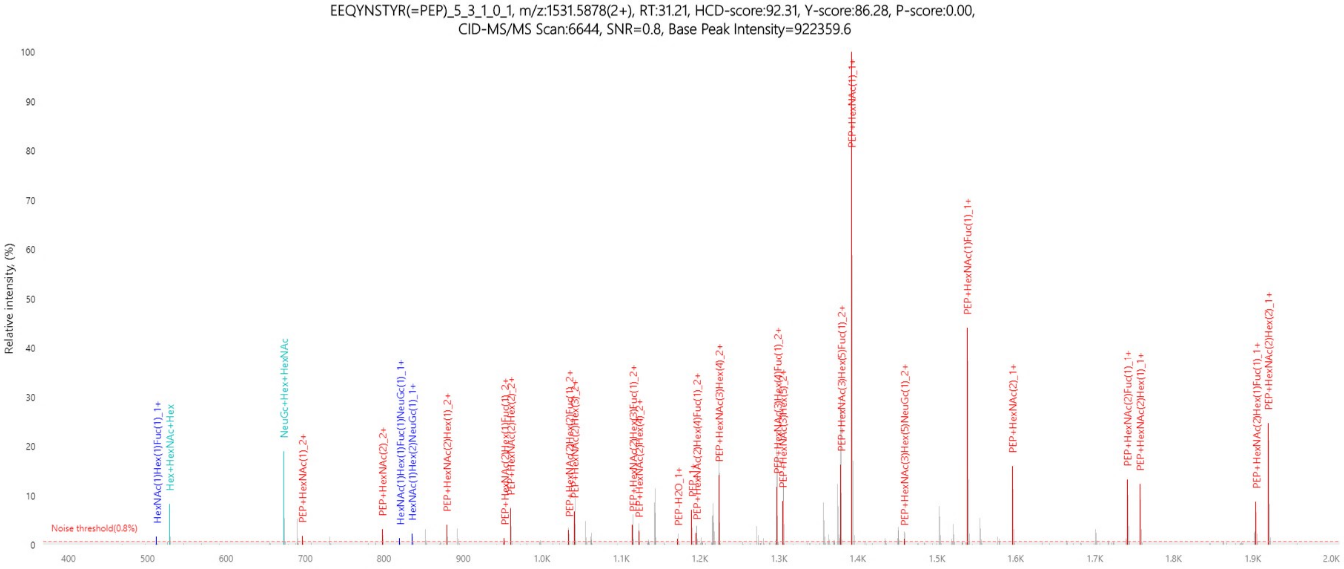

EEQYNSTYR(=PEP)_5_3_1_0_1, m/z:1531.5878(2+), RT:31.21, HCD-score:92.31, Y-score:86.28, P-score:0.00,
CID-MS/MS Scan:6644, SNR=0.8, Base Peak Intensity=922359.6
Relative intensity, (%)
Noise threshold(0.8%)
HexNAc(1)Hex(1)Fuc(1)_1+
Hex+HexNAc+Hex
NeuGc+Hex+HexNAc
PEP+HexNAc(1)_2+
PEP+HexNAc(2)_2+
HexNAc(1)Hex(1)Fuc(1)NeuGc(1)_1+
HexNAc(1)Hex(2)NeuGc(1)_1+
PEP+HexNAc(2)Hex(1)_2+
PEP-H2O_1+
PEP+HexNAc(3)Hex(4)_2+
PEP+HexNAc(3)Hex(5)Fuc(1)_2+
PEP+HexNAc(1)_1+
PEP+HexNAc(3)Hex(5)NeuGc(1)_2+
PEP+HexNAc(1)Fuc(1)_1+
PEP+HexNAc(2)_1+
PEP+HexNAc(2)Fuc(1)_1+
PEP+HexNAc(2)Hex(1)_1+
PEP+HexNAc(2)Hex(1)Fuc(1)_1+
PEP+HexNAc(2)Hex(2)_1+

EEQYNSTYR(=PEP)_5_4_1_0_0, m/z:986.7224(3+), RT:23.67, HCD-score:96.31, Y-score:96.80, P-score:33.33, HCD-MS/MS Scan:4448, SNR=0.8, Base Peak Intensity=8874729

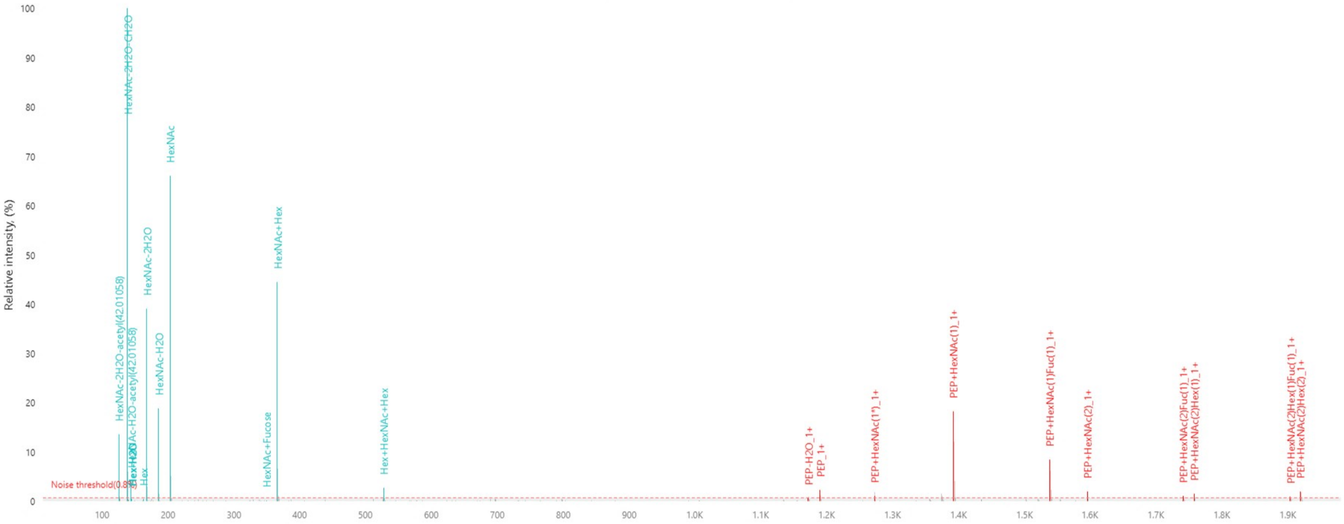

EEQYNSTYR(=PEP)_5_4_1_0_0, m/z:986.7224(3+), RT:23.68, HCD-score:96.31, Y-score:96.80, P-score:33.33, CID-MS/MS Scan:4450, SNR=0.8, Base Peak Intensity=6555218

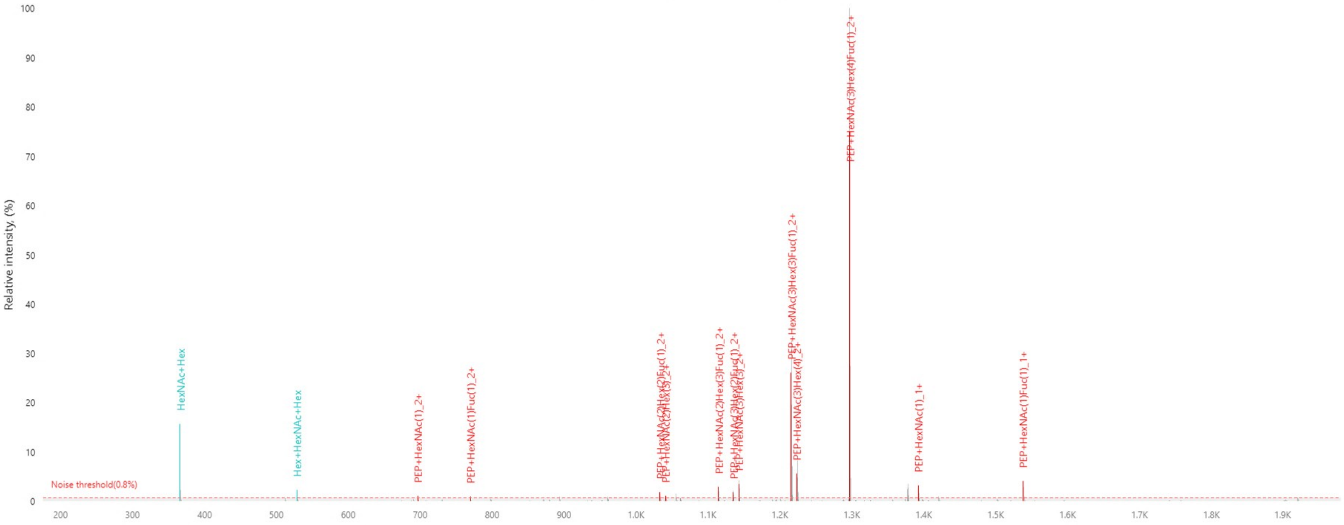

EEQYNSTYR(=PEP)_5_4_1_0_1, m/z:1089.0906(3+), RT:31.09, HCD-score:96.58, Y-score:97.85, P-score:11.11,
HCD-MS/MS Scan:6607, SNR=0.8, Base Peak Intensity=10377150

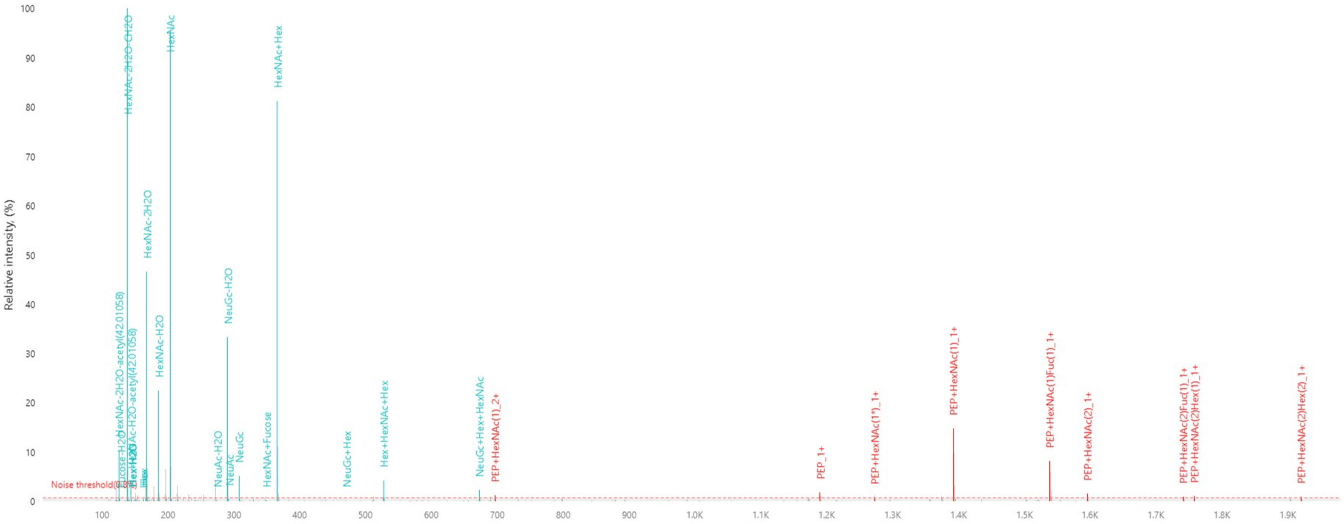

EEQYNSTYR(=PEP)_5_4_1_0_1, m/z:1089.0906(3+), RT:31.10, HCD-score:96.58, Y-score:97.85, P-score:11.11,
CID-MS/MS Scan:6609, SNR=0.8, Base Peak Intensity=10282135

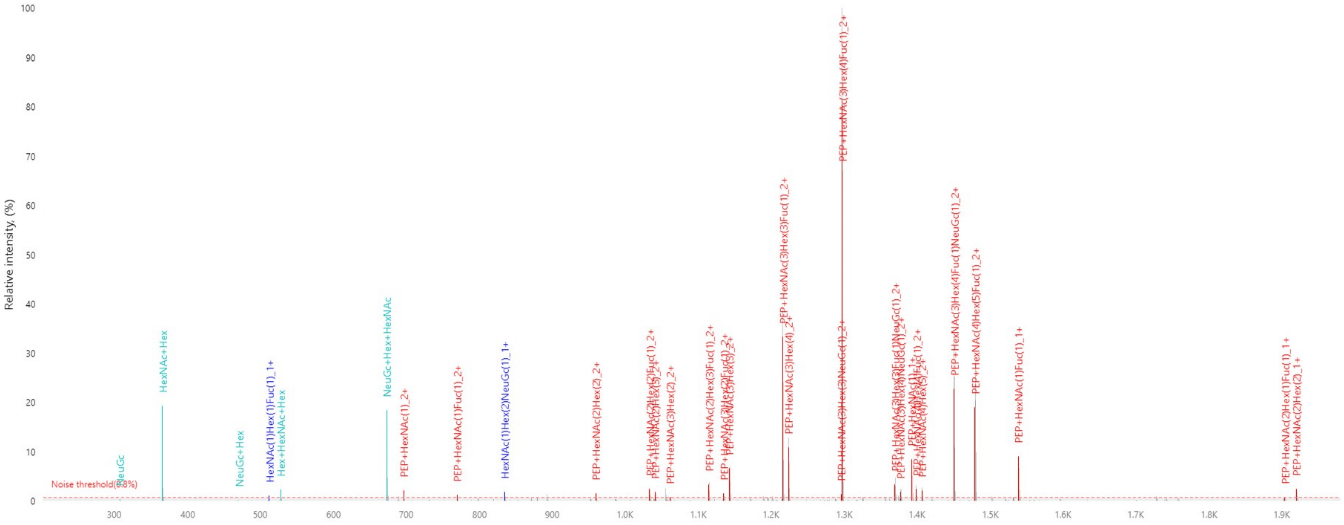

EEQYNSTYR(=PEP)_5_4_1_0_2, m/z:1191.4493(3+), RT:37.60, HCD-score:97.30, Y-score:98.05, P-score:33.33,
HCD-MS/MS Scan:8685, SNR=0.8, Base Peak Intensity=2748714

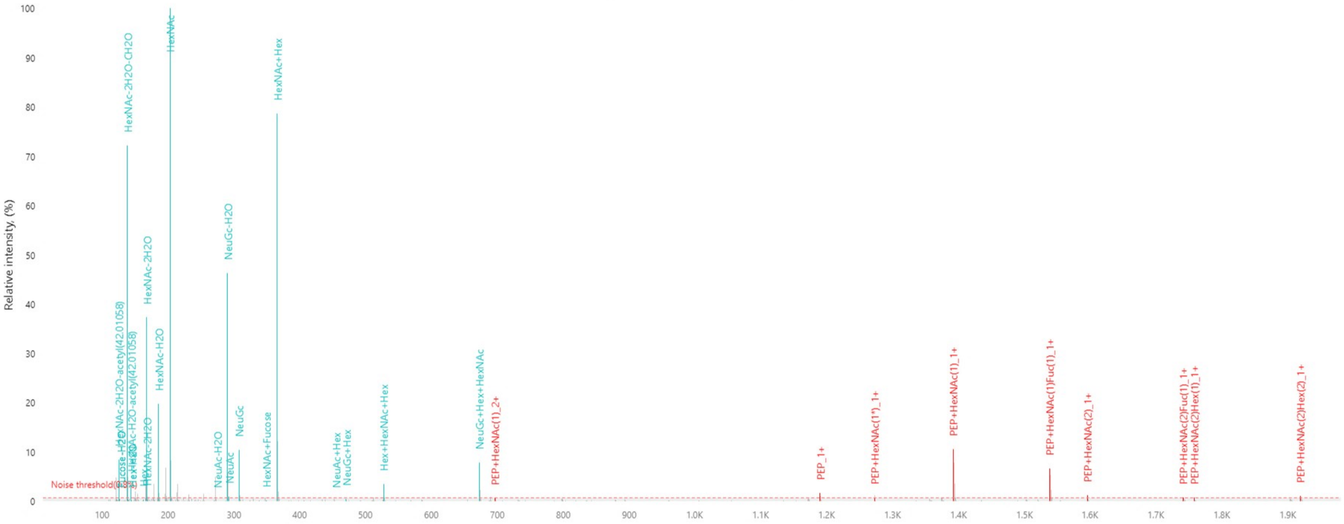

EEQYNSTYR(=PEP)_5_4_1_0_2, m/z:1191.4493(3+), RT:37.61, HCD-score:97.30, Y-score:98.05, P-score:33.33,
CID-MS/MS Scan:8687, SNR=0.8, Base Peak Intensity=2005993.8

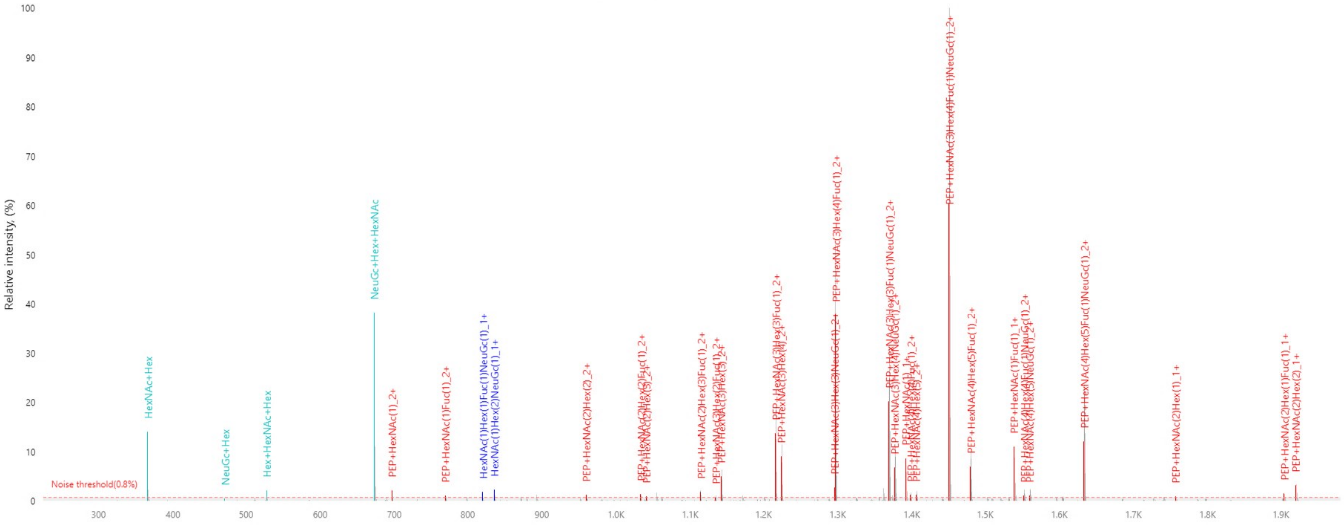

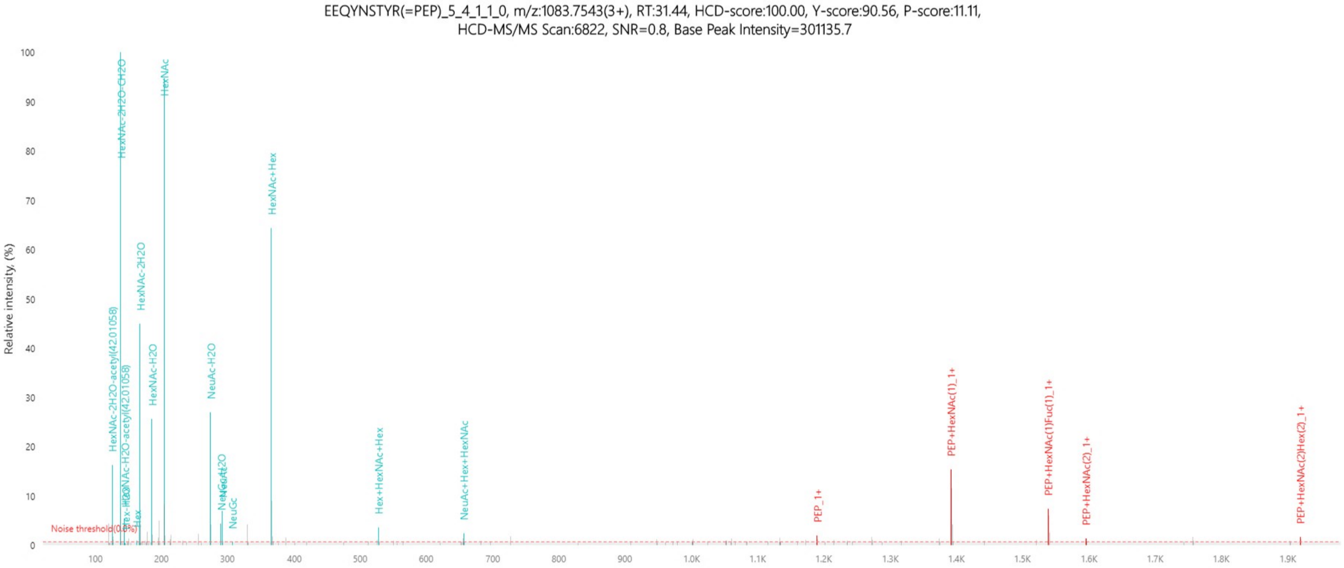
EEQYNSTYR(=PEP)_5_4_1_1_0, m/z:1083.7543(3+), RT:31.44, HCD-score:100.00, Y-score:90.56, P-score:11.11,
HCD-MS/MS Scan:6822, SNR=0.8, Base Peak Intensity=301135.7
Relative intensity, (%)
Noise threshold(0.8%)
HexNAc
HexNAc+Hex
HexNAc-2H2O
HexNAc-H2O
NeuAc-H2O
Hex+HexNAc+Hex
NeuAc+Hex+HexNAc
PEP_1+
PEP+HexNAc(1)_1+
PEP+HexNAc(1)Fuc(1)_1+
PEP+HexNAc(2)_1+
PEP+HexNAc(2)Hex(2)_1+

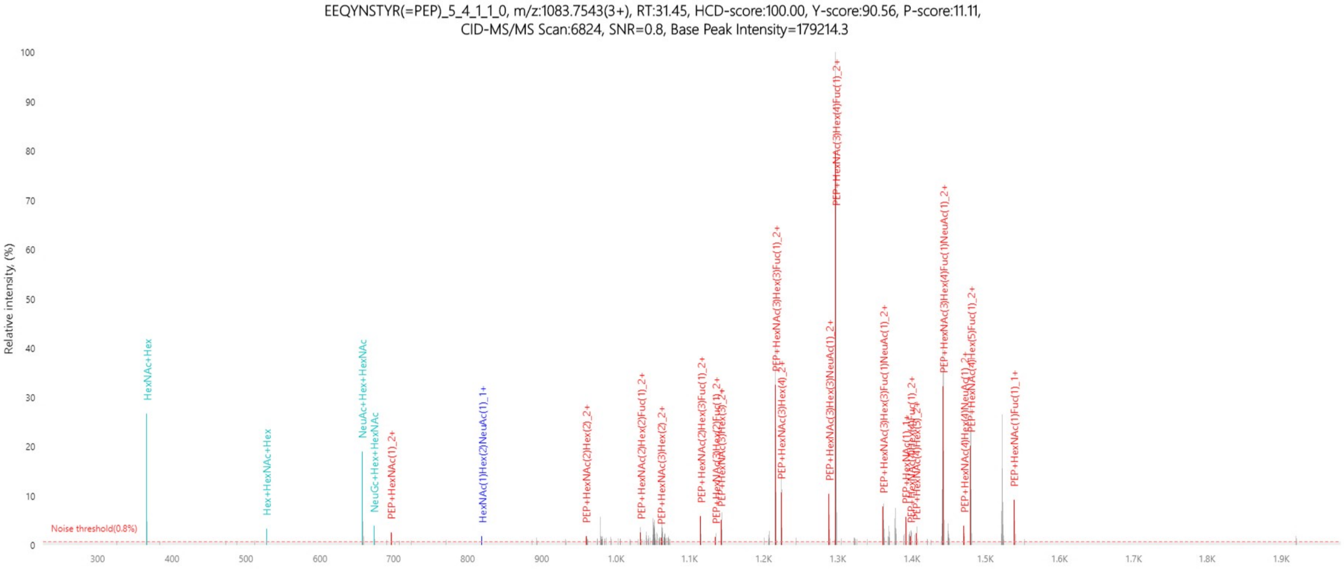
EEQYNSTYR(=PEP)_5_4_1_1_0, m/z:1083.7543(3+), RT:31.45, HCD-score:100.00, Y-score:90.56, P-score:11.11,
CID-MS/MS Scan:6824, SNR=0.8, Base Peak Intensity=179214.3
Relative intensity, (%)
Noise threshold(0.8%)
HexNAc+Hex
Hex+HexNAc+Hex
NeuAc+Hex+HexNAc
NeuGc+Hex+HexNAc
PEP+HexNAc(1)_2+
HexNAc(1)Hex(2)NeuAc(1)_1+
PEP+HexNAc(2)Hex(2)_2+
PEP+HexNAc(2)Hex(2)Fuc(1)_2+
PEP+HexNAc(3)Hex(2)_2+
PEP+HexNAc(3)Hex(3)Fuc(1)_2+
PEP+HexNAc(3)Hex(4)_2+
PEP+HexNAc(3)Hex(4)Fuc(1)_2+
PEP+HexNAc(3)Hex(3)NeuAc(1)_2+
PEP+HexNAc(3)Hex(3)Fuc(1)NeuAc(1)_2+
PEP+HexNAc(3)Hex(4)Fuc(1)NeuAc(1)_2+
PEP+HexNAc(4)Hex(5)Fuc(1)_2+
PEP+HexNAc(1)Fuc(1)_1+

EEQYNSTYR(=PEP)_6_2_0_0_0, m/z:1284.4993(2+), RT:22.51, HCD-score:88.22, Y-score:80.22, P-score:0.00,
HCD-MS/MS Scan:4124, SNR=0.8, Base Peak Intensity=1334294.9

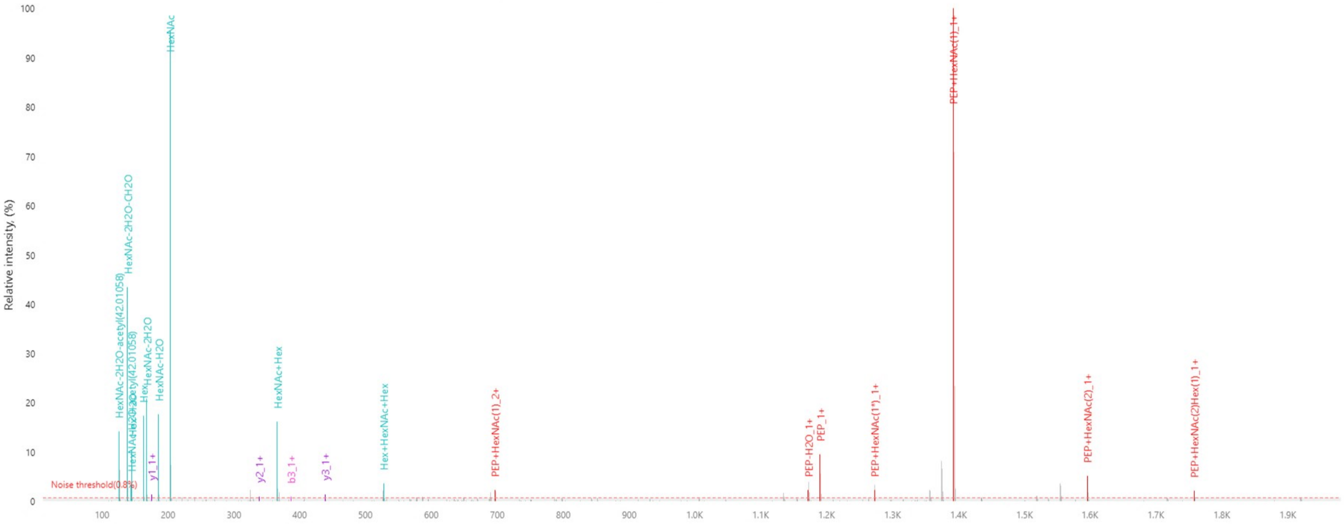

EEQYNSTYR(=PEP)_6_2_0_0_0, m/z:1284.4993(2+), RT:22.51, HCD-score:88.22, Y-score:80.22, P-score:0.00,
CID-MS/MS Scan:4126, SNR=0.8, Base Peak Intensity=2259892.2

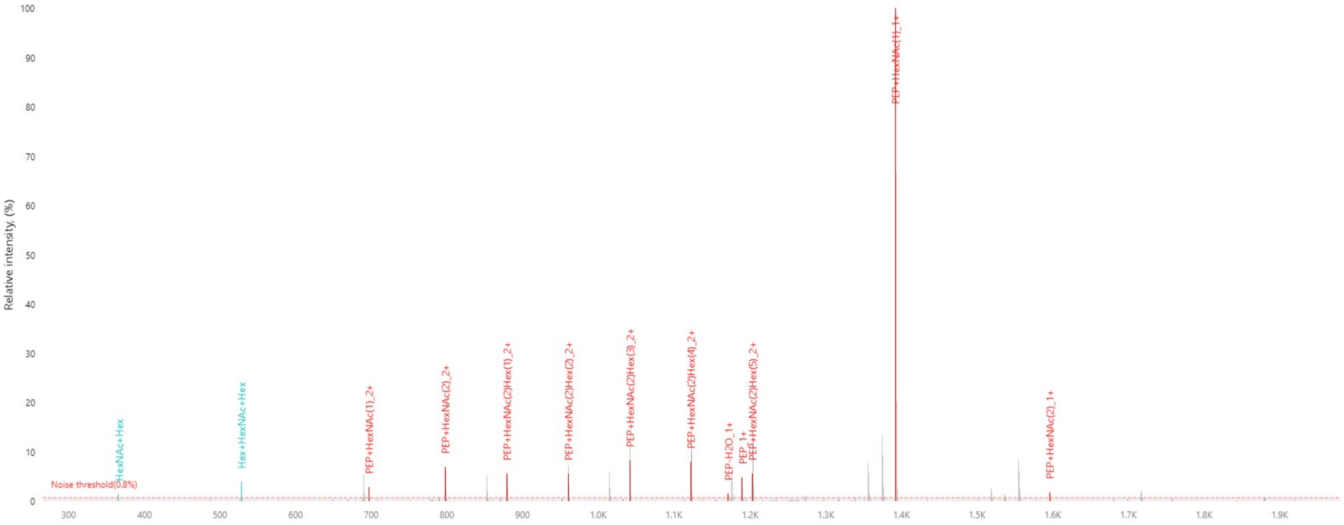

EEQYNSTYR(=PEP)_6_3_0_0_0, m/z:1386.0391(2+), RT:22.95, HCD-score:92.86, Y-score:79.84, P-score:0.00,
HCD-MS/MS Scan:4247, SNR=0.8, Base Peak Intensity=9041562

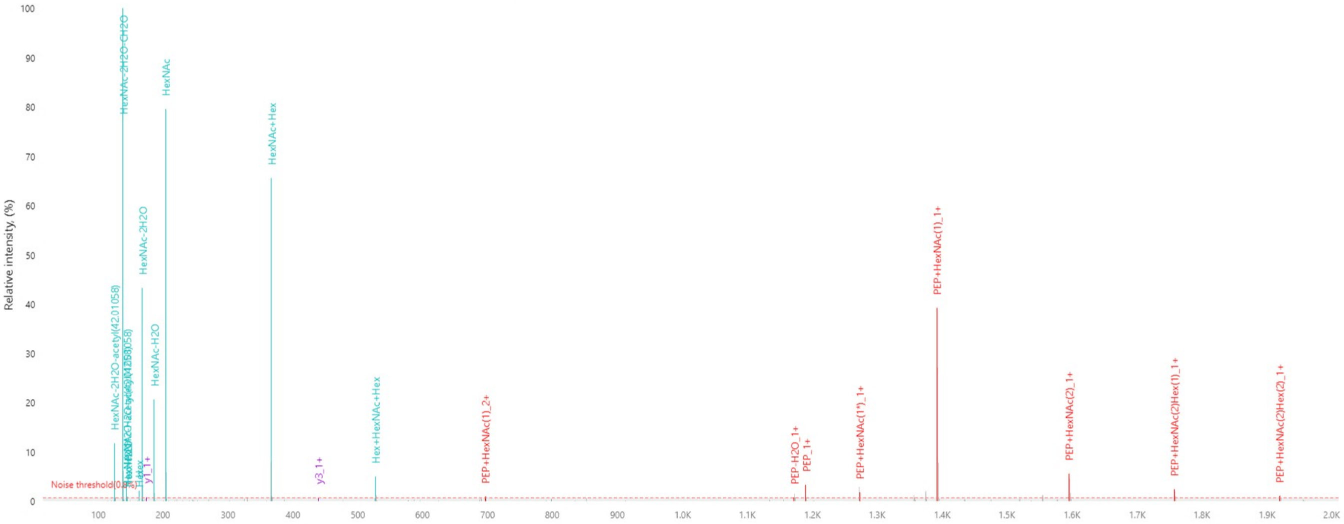

EEQYNSTYR(=PEP)_6_3_0_0_0, m/z:1386.0391(2+), RT:22.96, HCD-score:92.86, Y-score:79.84, P-score:0.00,
CID-MS/MS Scan:4249, SNR=0.8, Base Peak Intensity=4059398

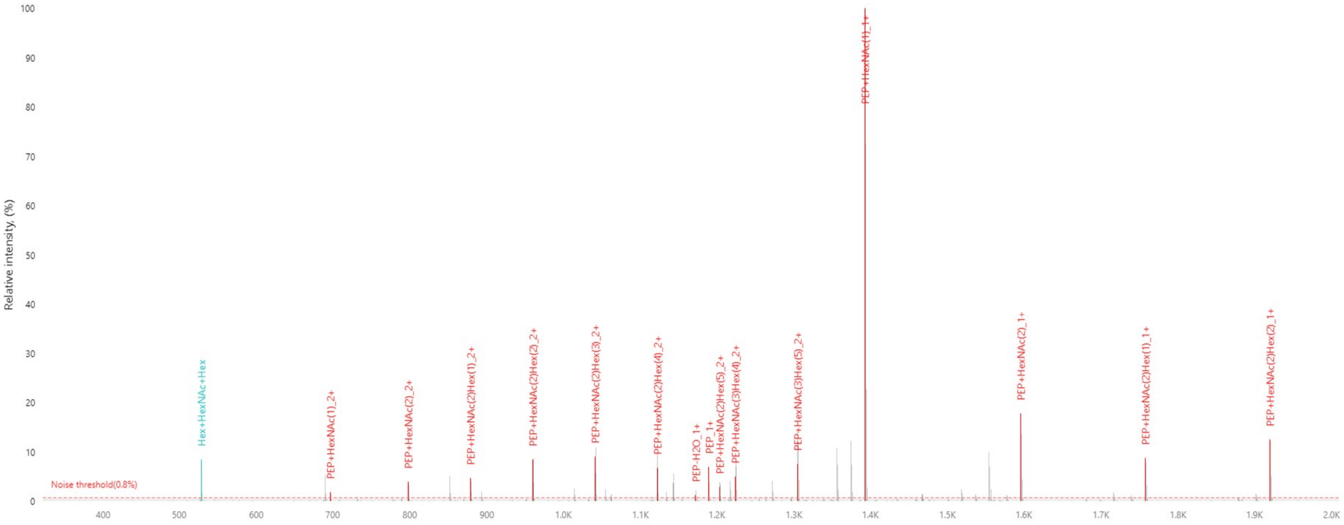

EEQYNSTYR(=PEP)_6_3_0_0_1, m/z:770.2969(4+), RT:31.24, HCD-score:81.50, Y-score:92.24, P-score:11.11,
HCD-MS/MS Scan:6650, SNR=0.8, Base Peak Intensity=508660.1

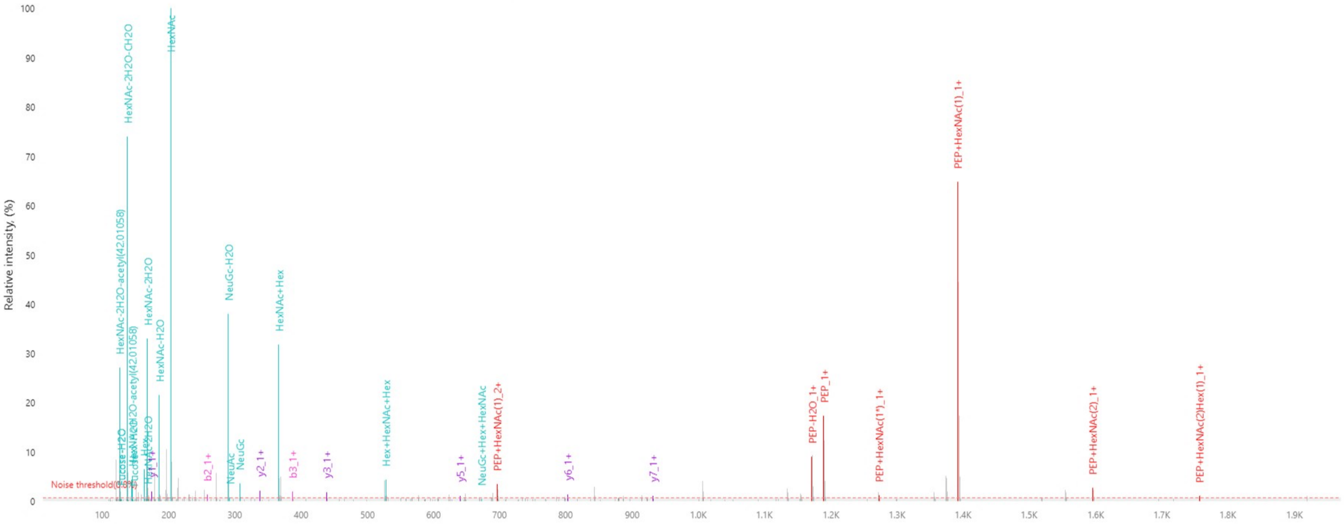

EEQYNSTYR(=PEP)_6_3_0_0_1, m/z:770.2969(4+), RT:31.24, HCD-score:81.50, Y-score:92.24, P-score:11.11,
CID-MS/MS Scan:6652, SNR=0.8, Base Peak Intensity=351401.3

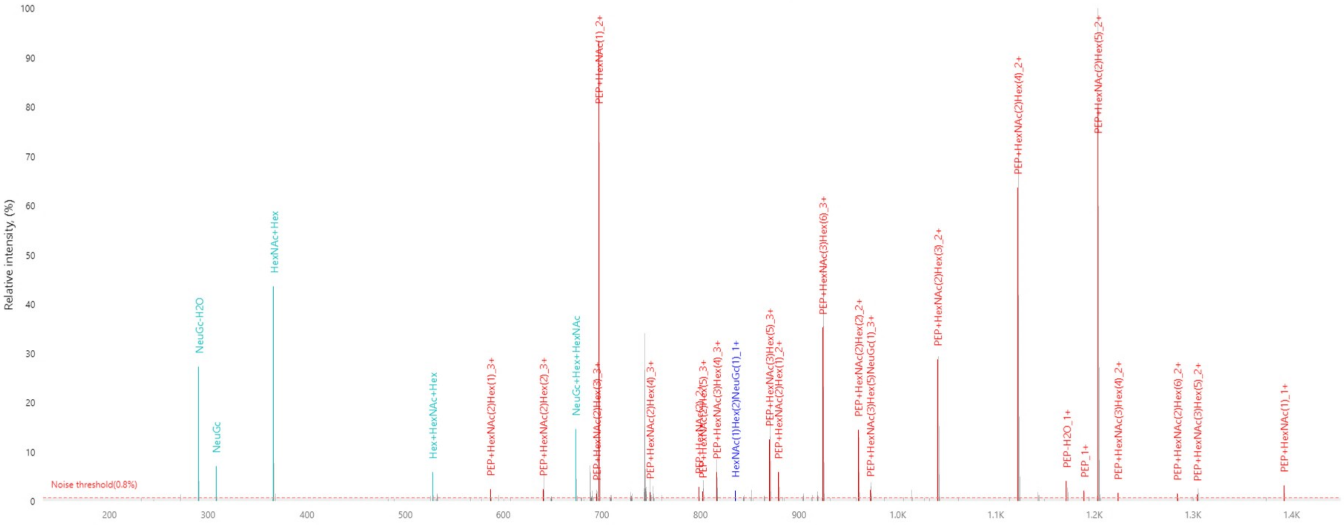

EEQYNSTYR(=PEP)_6_3_1_0_0, m/z:973.0532(3+), RT:22.68, HCD-score:90.78, Y-score:95.64, P-score:44.44,
HCD-MS/MS Scan:4173, SNR=0.8, Base Peak Intensity=5217756.5

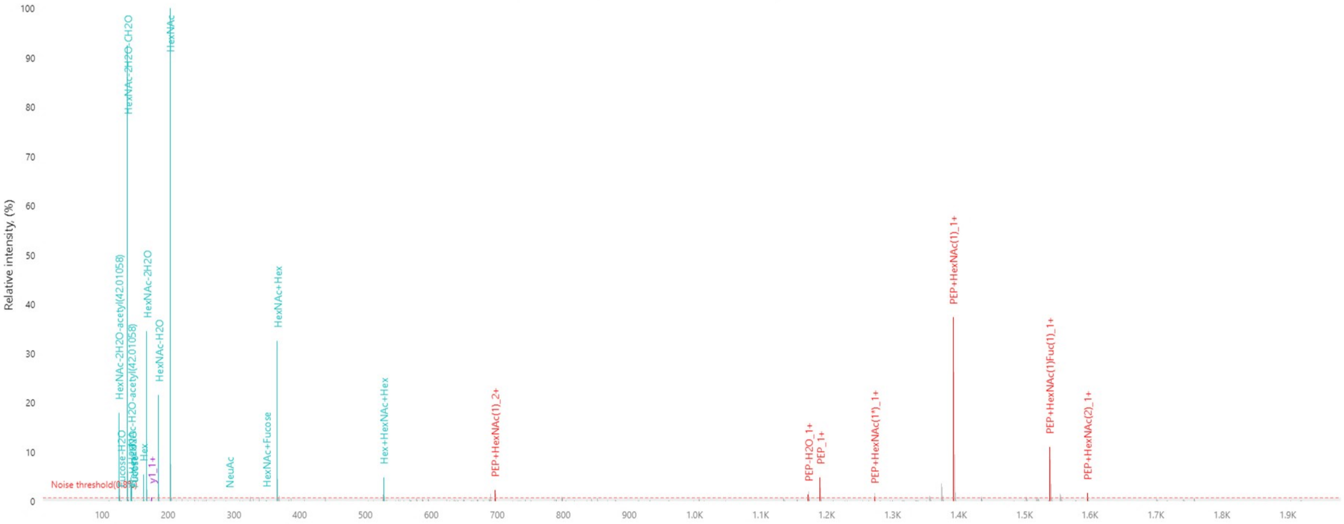

EEQYNSTYR(=PEP)_6_3_1_0_0, m/z:973.0532(3+), RT:22.69, HCD-score:90.78, Y-score:95.64, P-score:44.44,
CID-MS/MS Scan:4175, SNR=0.8, Base Peak Intensity=7952449.5

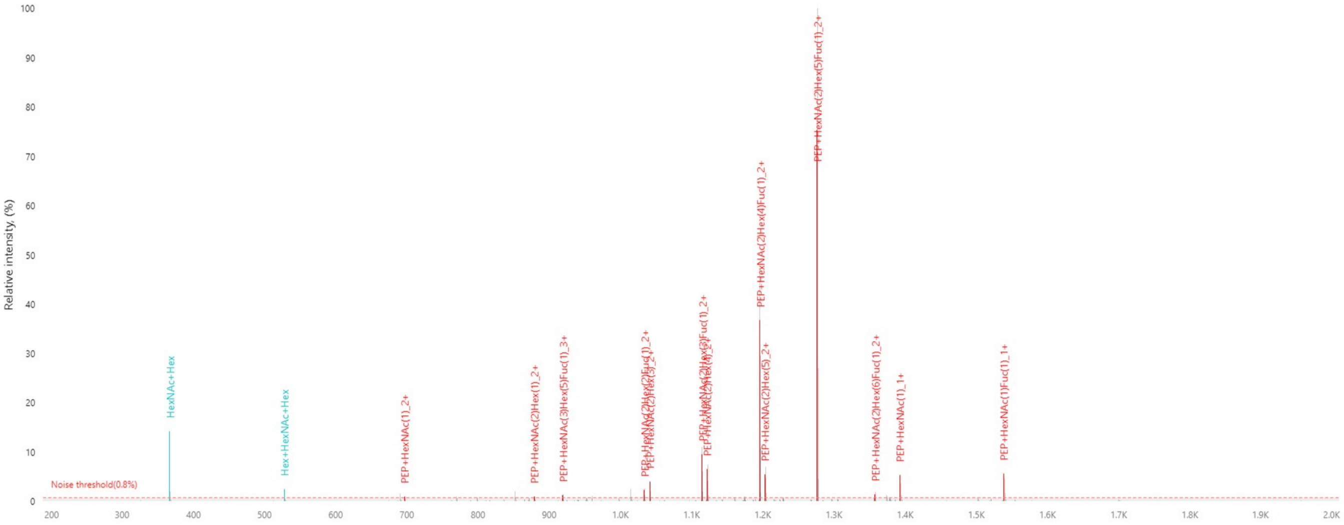

EEQYNSTYR(=PEP)_6_3_1_0_1, m/z:1075.4141(3+), RT:30.89, HCD-score:93.09, Y-score:93.79, P-score:33.33,
HCD-MS/MS Scan:6544, SNR=0.8, Base Peak Intensity=15256513

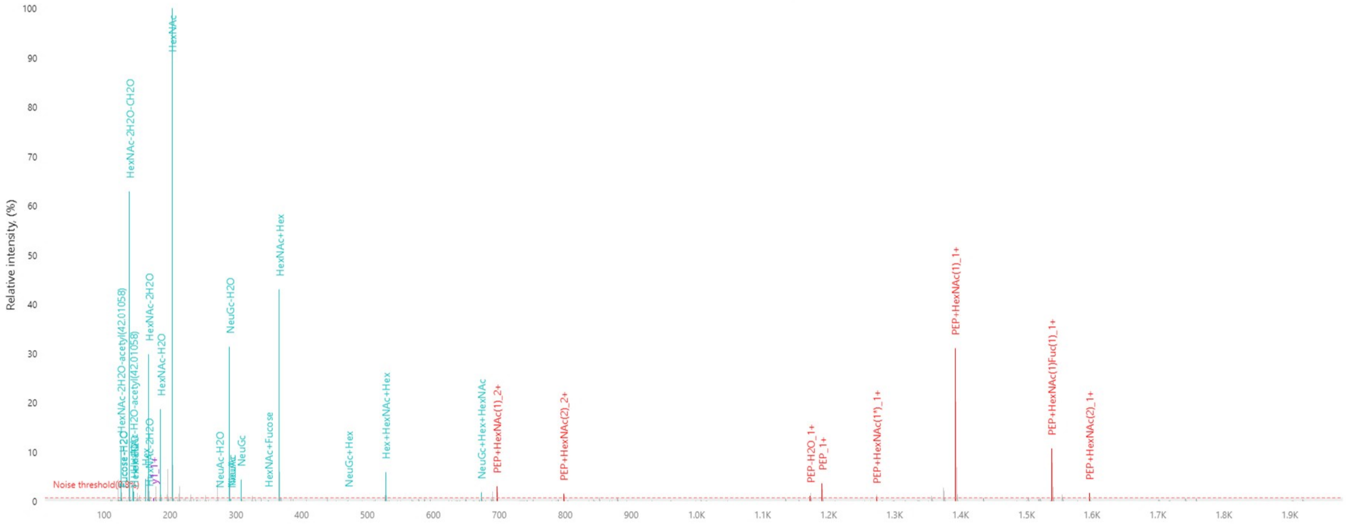

EEQYNSTYR(=PEP)_6_3_1_0_1, m/z:1075.4141(3+), RT:30.89, HCD-score:93.09, Y-score:93.79, P-score:33.33,
CID-MS/MS Scan:6546, SNR=0.8, Base Peak Intensity=9434490

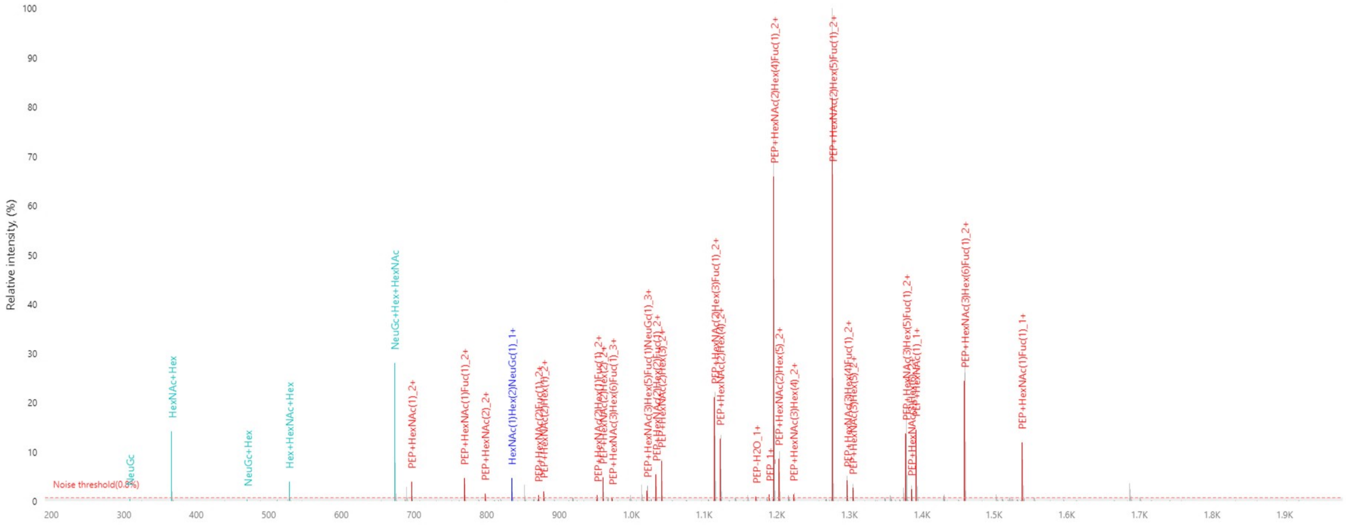

EEQYNSTYR(=PEP)_6_4_1_0_0, m/z:1040.7424(3+), RT:23.00, HCD-score:92.53, Y-score:90.59, P-score:,
HCD-MS/MS Scan:4260, SNR=0.8, Base Peak Intensity=4740147

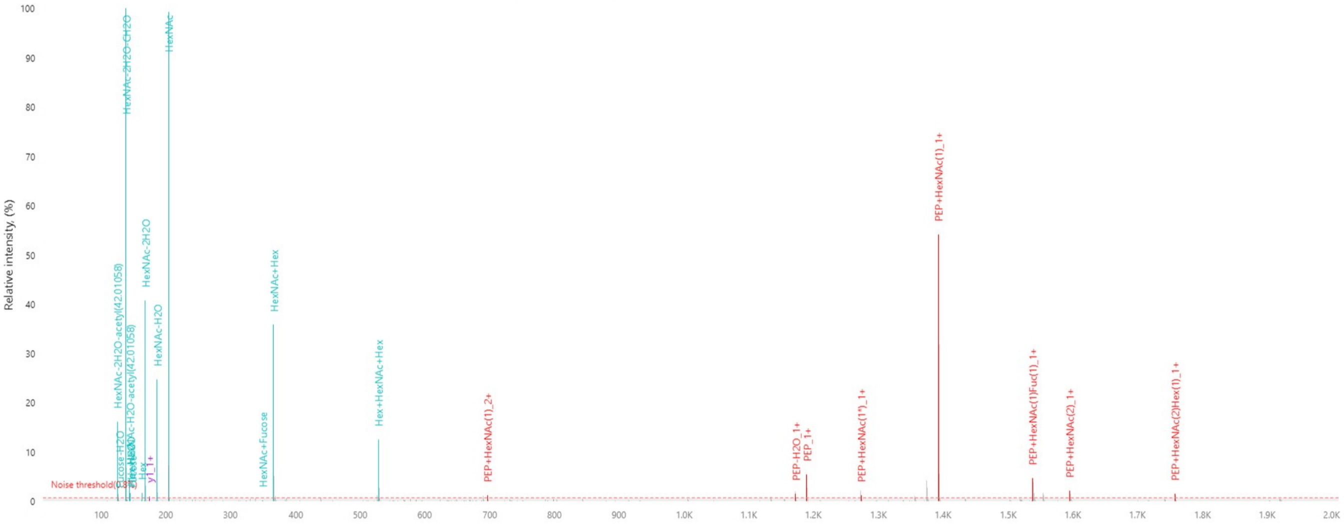

EEQYNSTYR(=PEP)_6_4_1_0_0, m/z:1040.7424(3+), RT:23.00, HCD-score:92.53, Y-score:90.59, P-score:,
CID-MS/MS Scan:4262, SNR=0.8, Base Peak Intensity=3268135.8

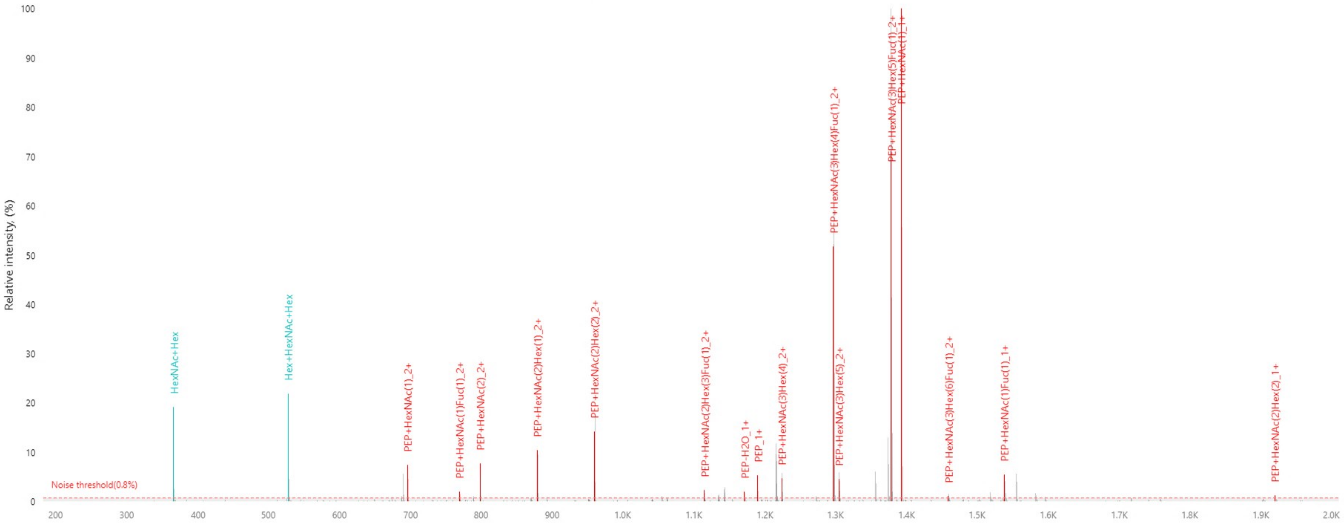

EEQYNSTYR(=PEP)_6_4_1_0_1, m/z:857.5813(4+), RT:30.96, HCD-score:92.66, Y-score:88.97, P-score:22.22,
HCD-MS/MS Scan:6567, SNR=0.8, Base Peak Intensity=490706.5

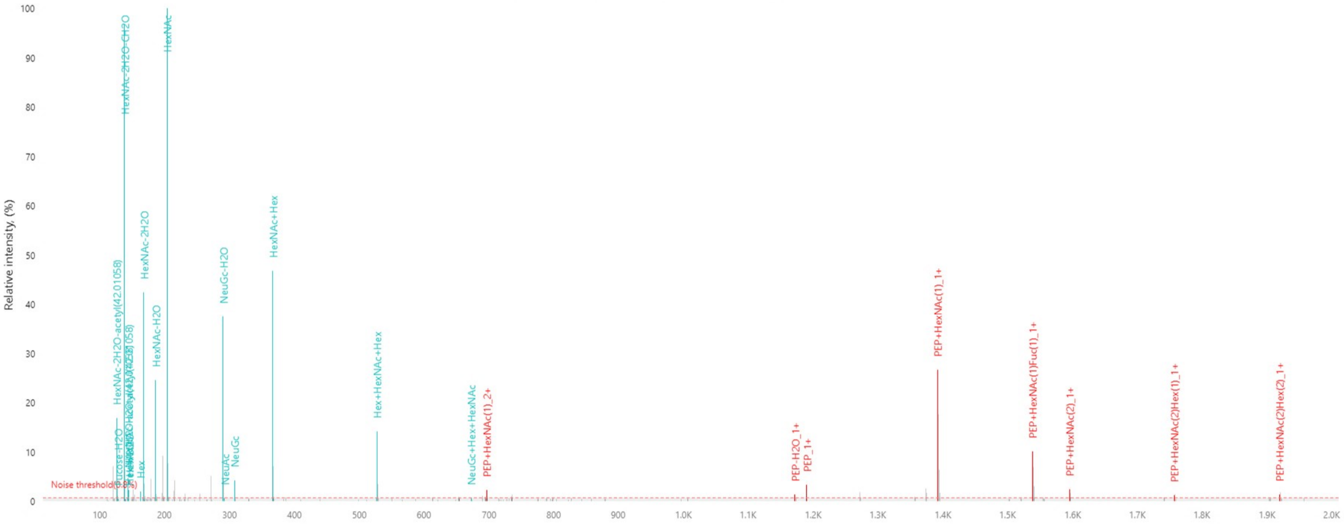

EEQYNSTYR(=PEP)_6_4_1_0_1, m/z:857.5813(4+), RT:30.97, HCD-score:92.66, Y-score:88.97, P-score:22.22,
CID-MS/MS Scan:6569, SNR=0.8, Base Peak Intensity=212473

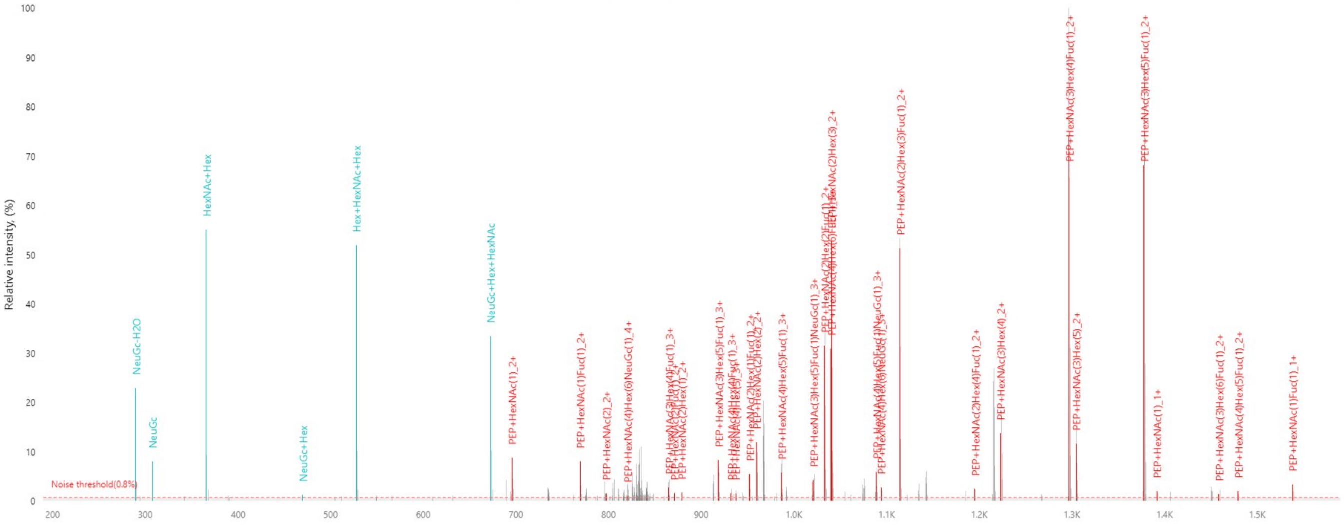

EEQYNSTYR(=PEP)_7_2_0_0_0, m/z:1365.5250(2+), RT:22.12, HCD-score:87.30, Y-score:79.28, P-score:0.00,
HCD-MS/MS Scan:4018, SNR=0.8, Base Peak Intensity=432378.8

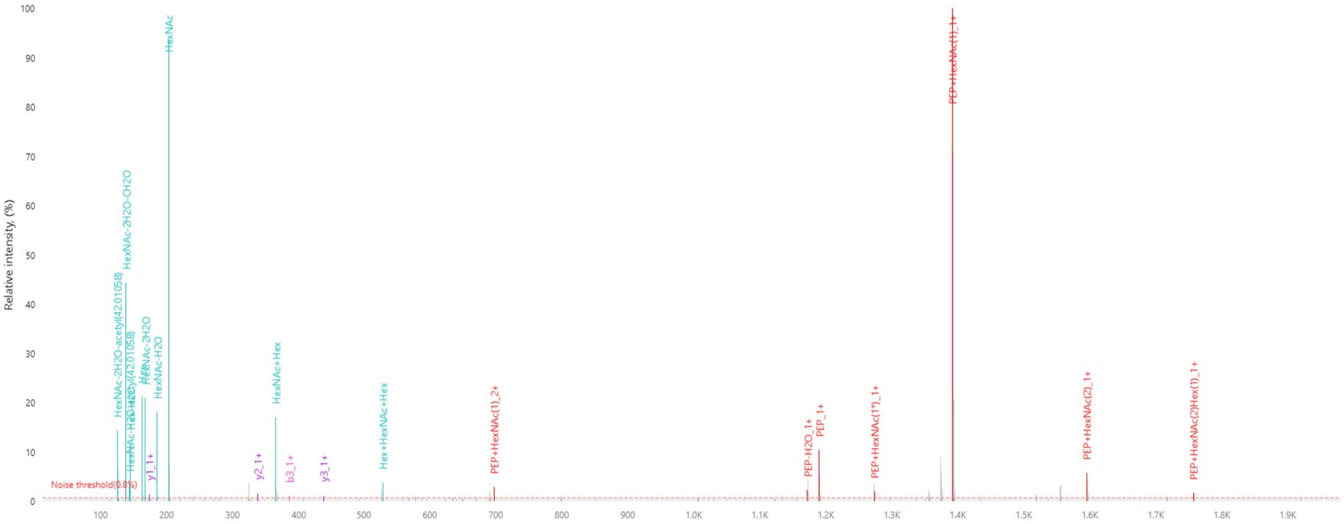

EEQYNSTYR(=PEP)_7_2_0_0_0, m/z:1365.5250(2+), RT:22.12, HCD-score:87.30, Y-score:79.28, P-score:0.00,
CID-MS/MS Scan:4020, SNR=0.8, Base Peak Intensity=669567

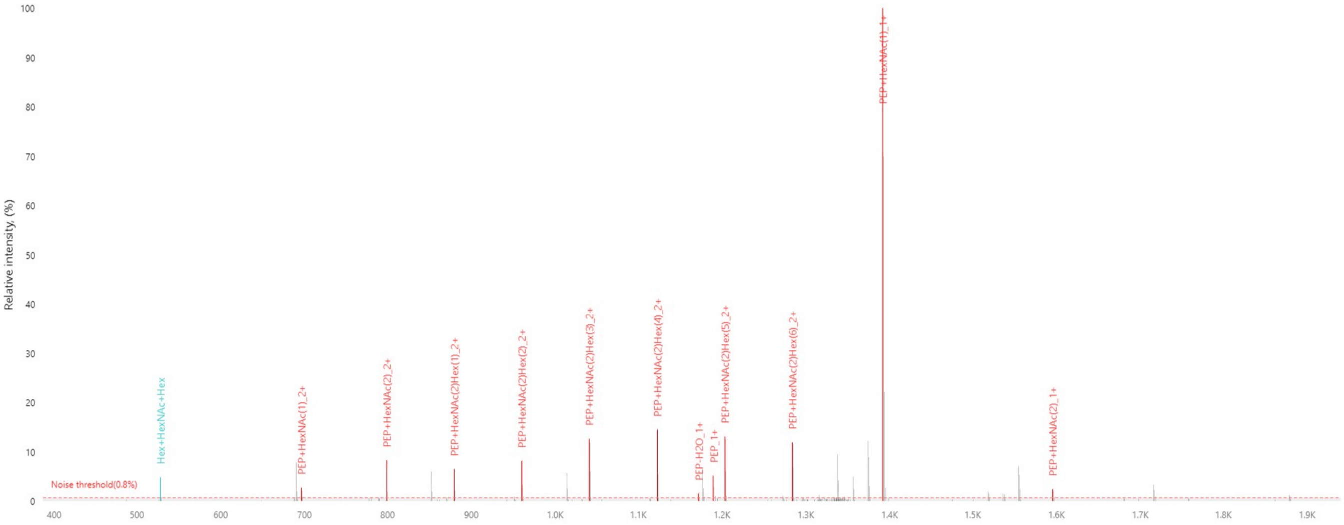

EEQYNSTYR(=PEP)_7_4_1_0_0, m/z:1094.7594(3+), RT:22.86, HCD-score:96.39, Y-score:95.32, P-score:22.22,
HCD-MS/MS Scan:4223, SNR=0.8, Base Peak Intensity=1666759.5

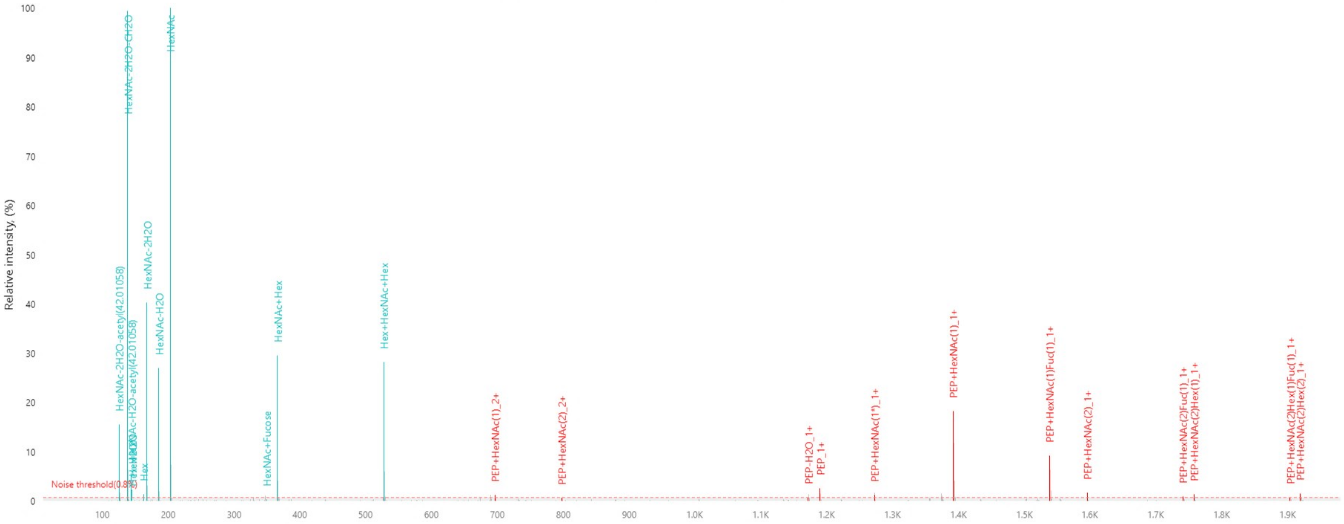

EEQYNSTYR(=PEP)_7_4_1_0_0, m/z:1094.7594(3+), RT:22.87, HCD-score:96.39, Y-score:95.32, P-score:22.22,
CID-MS/MS Scan:4225, SNR=0.8, Base Peak Intensity=2136334

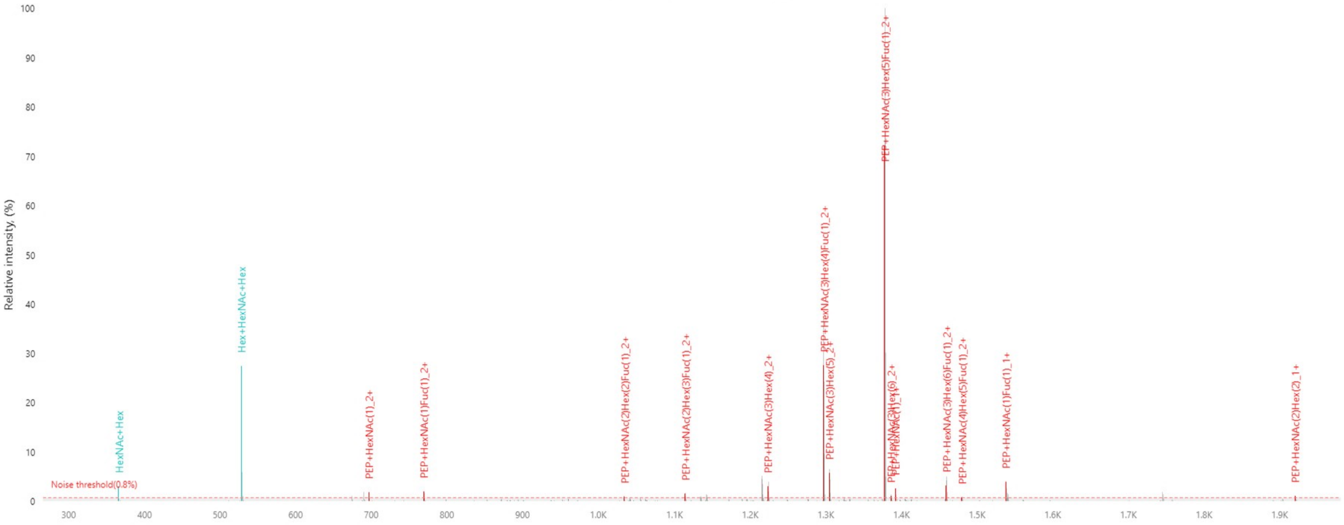

EEQYNSTYR(=PEP)_8_2_0_0_0, m/z:964.7035(3+), RT:21.87, HCD-score:85.15, Y-score:92.22, P-score:0.00,
HCD-MS/MS Scan:3951, SNR=0.8, Base Peak Intensity=631524.3

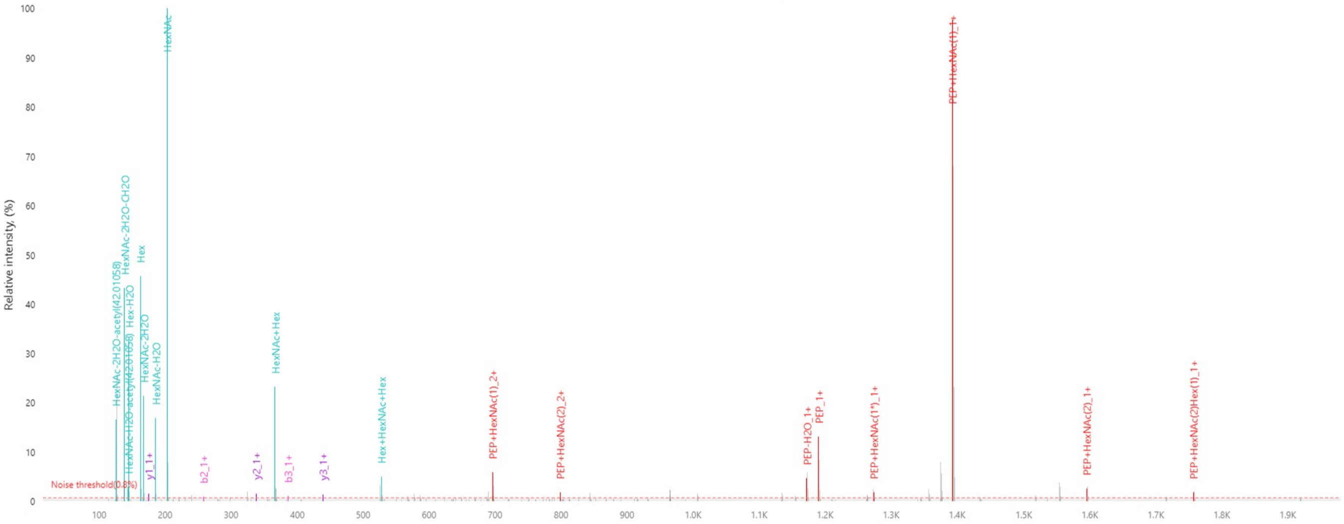

EEQYNSTYR(=PEP)_8_2_0_0_0, m/z:964.7035(3+), RT:21.89, HCD-score:85.15, Y-score:92.22, P-score:0.00,
CID-MS/MS Scan:3954, SNR=0.8, Base Peak Intensity=545202.2

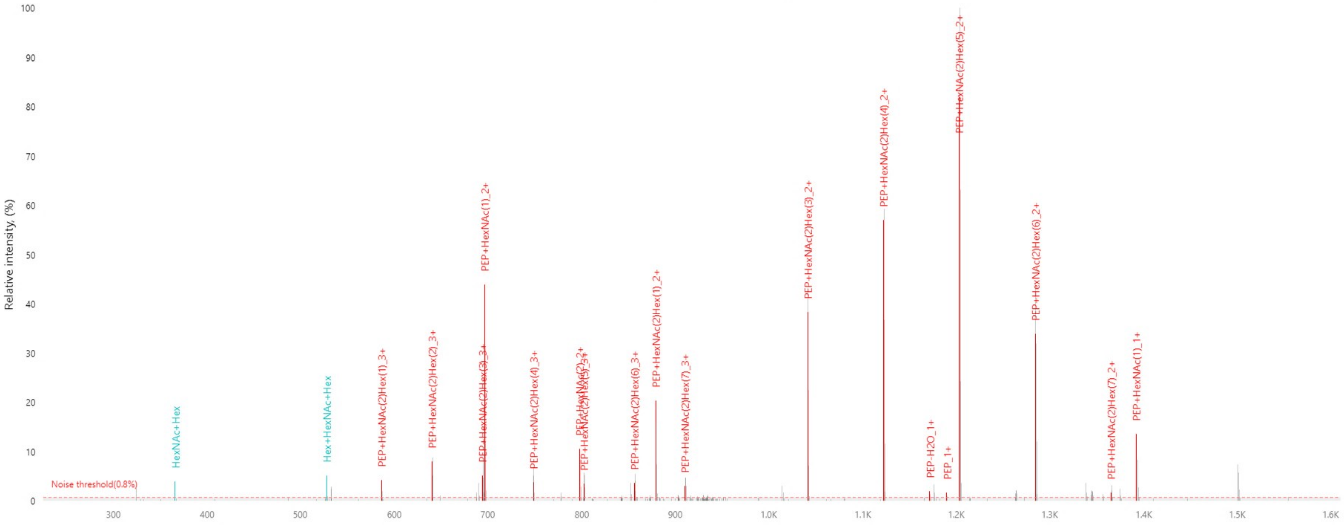

TNGSPR(=PEP)_5_2_0_0_0, m/z:924.3748(2+), RT:13.92, HCD-score:95.97, Y-score:88.40, P-score:16.67,
HCD-MS/MS Scan:1903, SNR=0.8, Base Peak Intensity=5053980.5

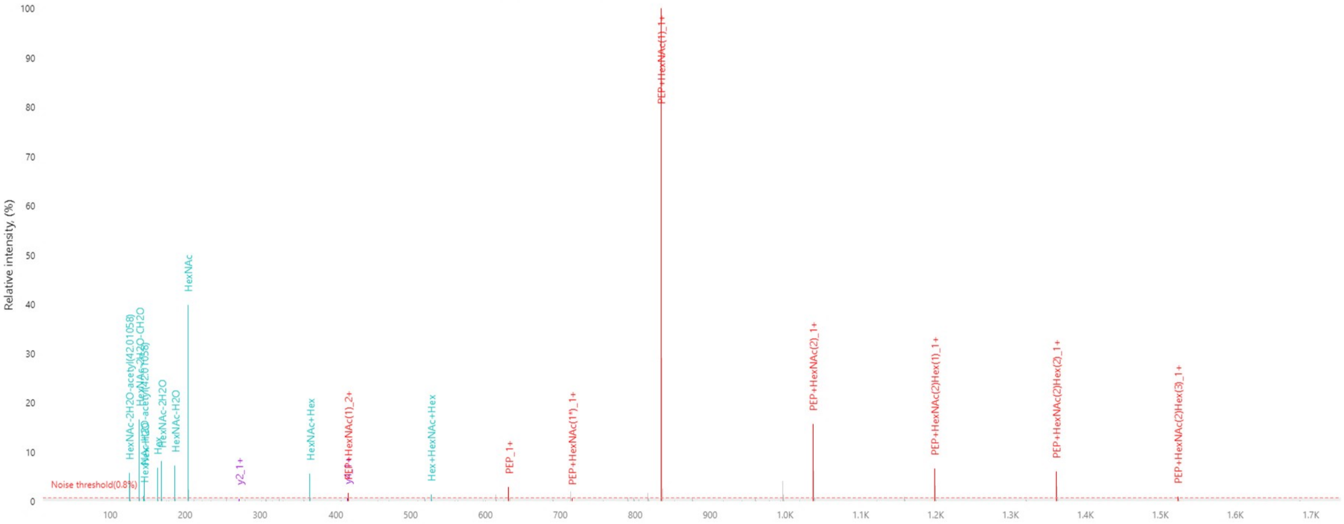

TNGSPR(=PEP)_5_2_0_0_0, m/z:924.3748(2+), RT:13.92, HCD-score:95.97, Y-score:88.40, P-score:16.67,
CID-MS/MS Scan:1905, SNR=0.8, Base Peak Intensity=1327800.1

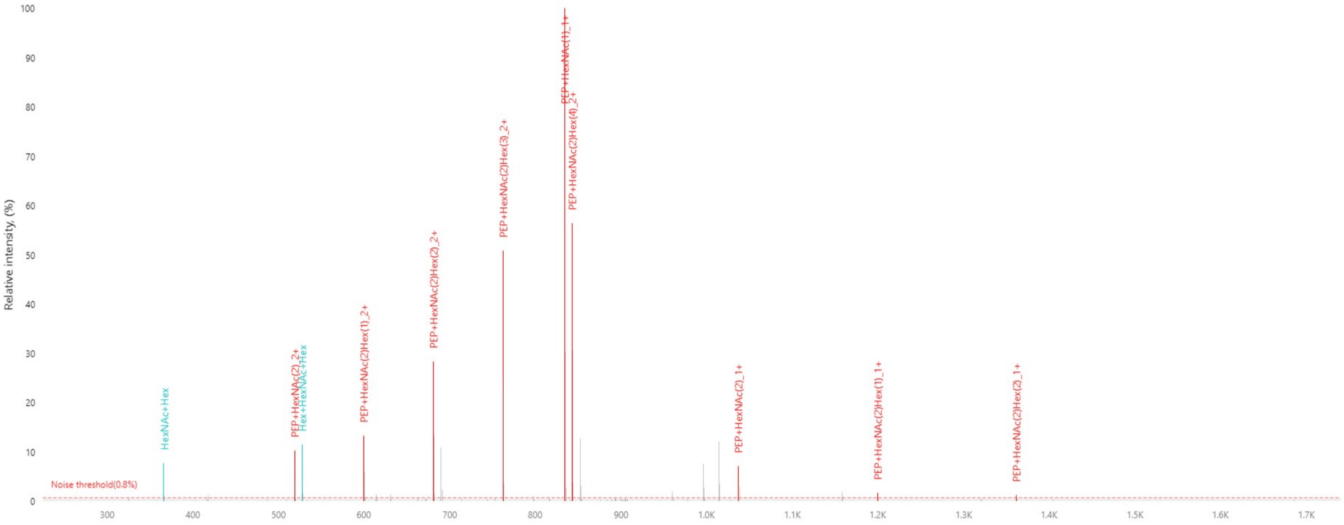

TNGSPR(=PEP)_5_3_0_0_0, m/z:1025.9143(2+), RT:14.04, HCD-score:91.87, Y-score:81.66, P-score:0.00,
HCD-MS/MS Scan:1907, SNR=0.8, Base Peak Intensity=21645.4

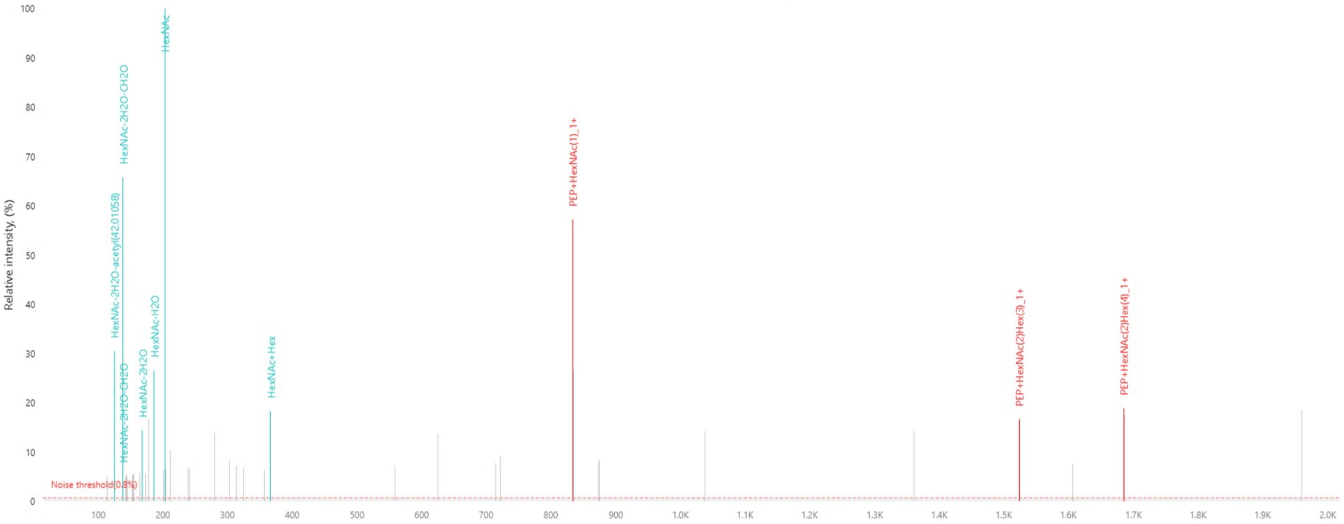

TNGSPR(=PEP)_5_3_0_0_0, m/z:1025.9143(2+), RT:14.05, HCD-score:91.87, Y-score:81.66, P-score:0.00,
CID-MS/MS Scan:1909, SNR=0.8, Base Peak Intensity=9519.5

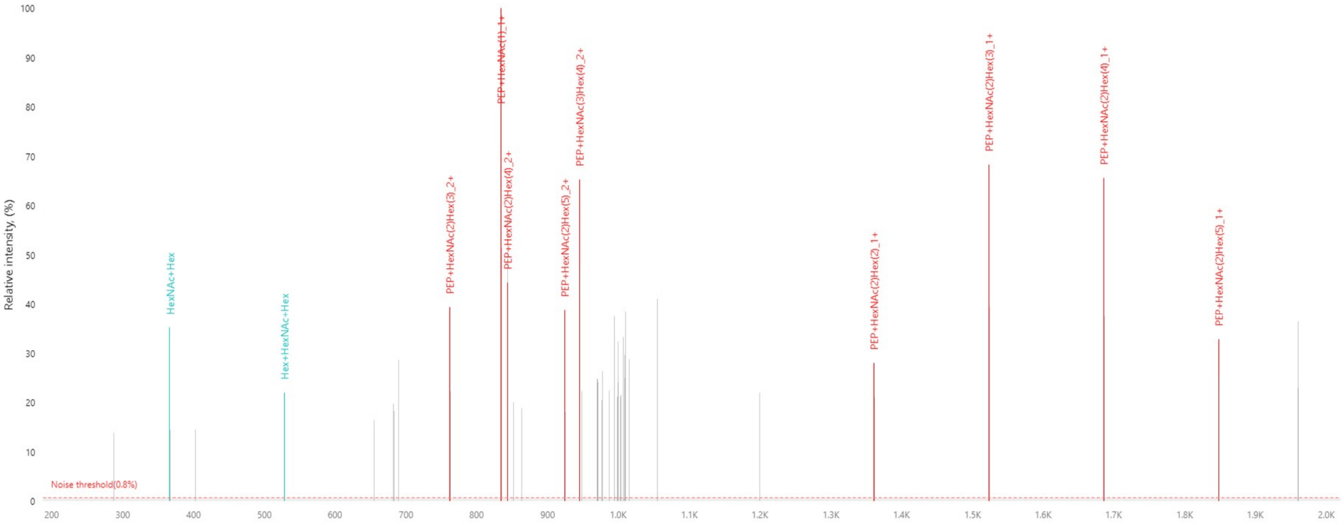

TNGSPR(=PEP)_6_3_0_0_0, m/z:1106.9398(2+), RT:13.99, HCD-score:100.00, Y-score:94.52, P-score:0.00,
HCD-MS/MS Scan:1884, SNR=0.8, Base Peak Intensity=26562.5

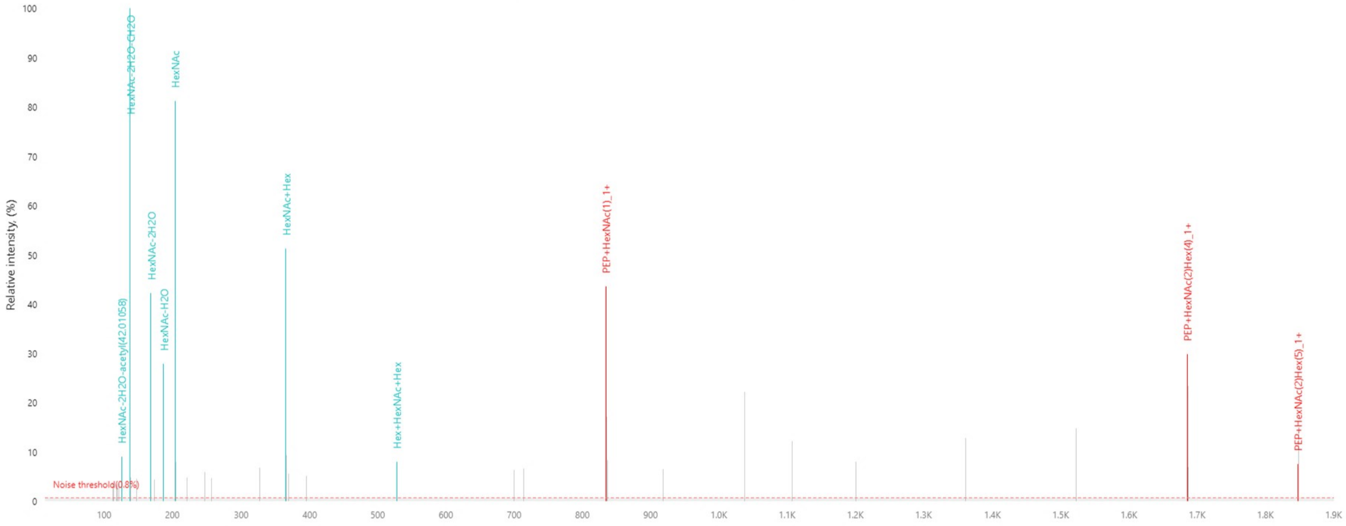

TNGSPR(=PEP)_6_3_0_0_0, m/z:1106.9398(2+), RT:14.00, HCD-score:100.00, Y-score:94.52, P-score:0.00,
CID-MS/MS Scan:1886, SNR=0.8, Base Peak Intensity=14019.2

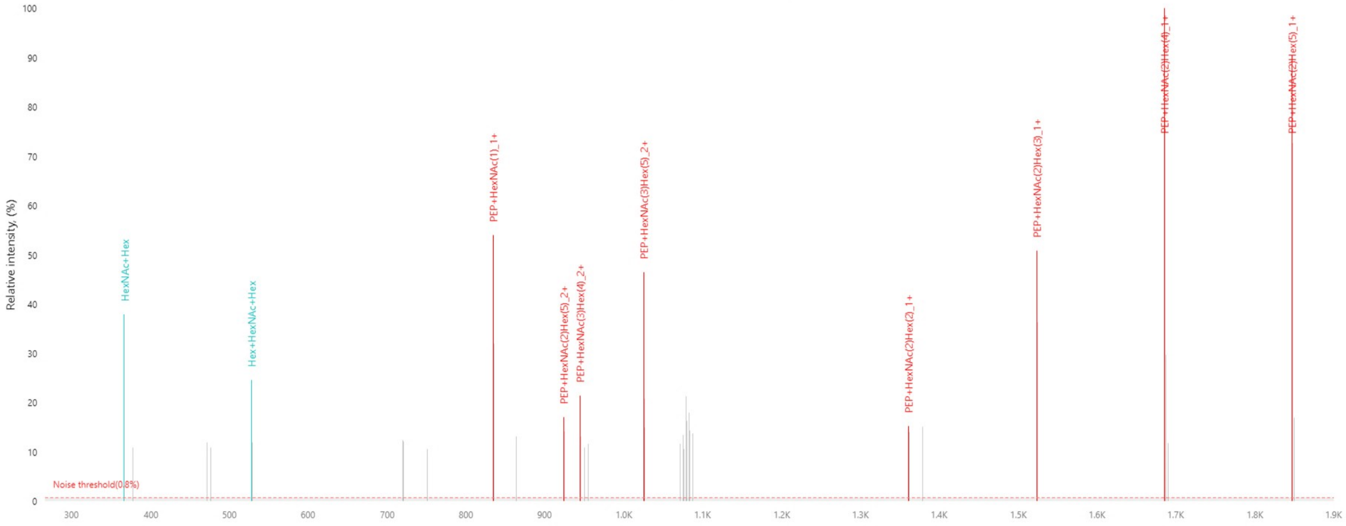

TNGSPR(=PEP)_6_3_0_0_1, m/z:840.6597(3+), RT:14.02, HCD-score:95.96, Y-score:91.63, P-score:,
HCD-MS/MS Scan:1923, SNR=0.8, Base Peak Intensity=78469.4

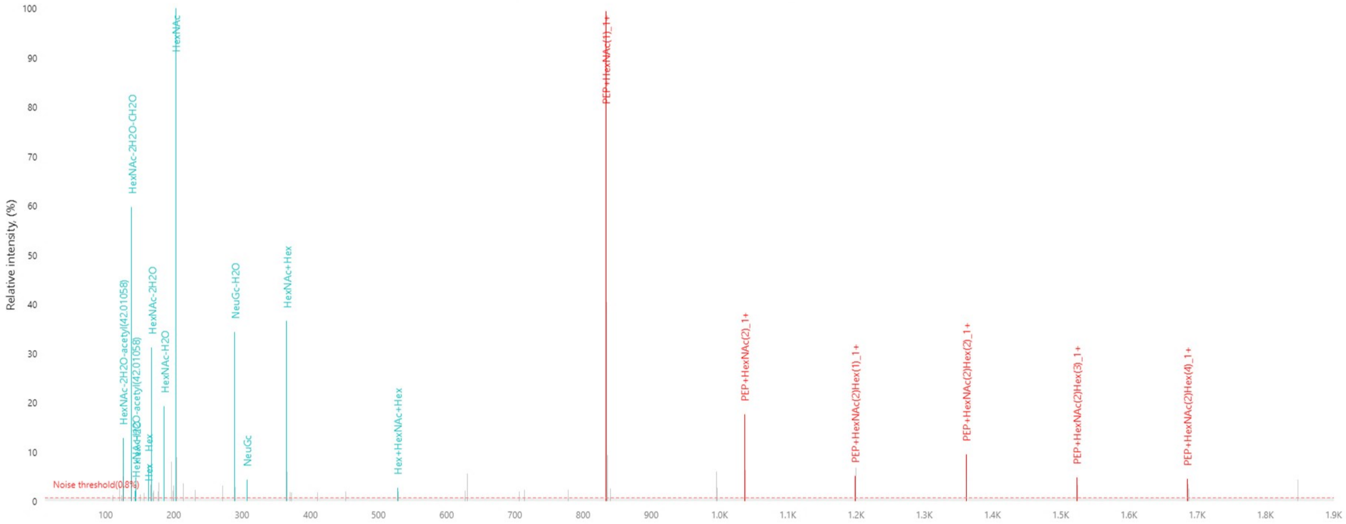

TNGSPR(=PEP)_6_3_0_0_1, m/z:840.6597(3+), RT:14.03, HCD-score:95.96, Y-score:91.63, P-score:,
CID-MS/MS Scan:1925, SNR=0.8, Base Peak Intensity=50202

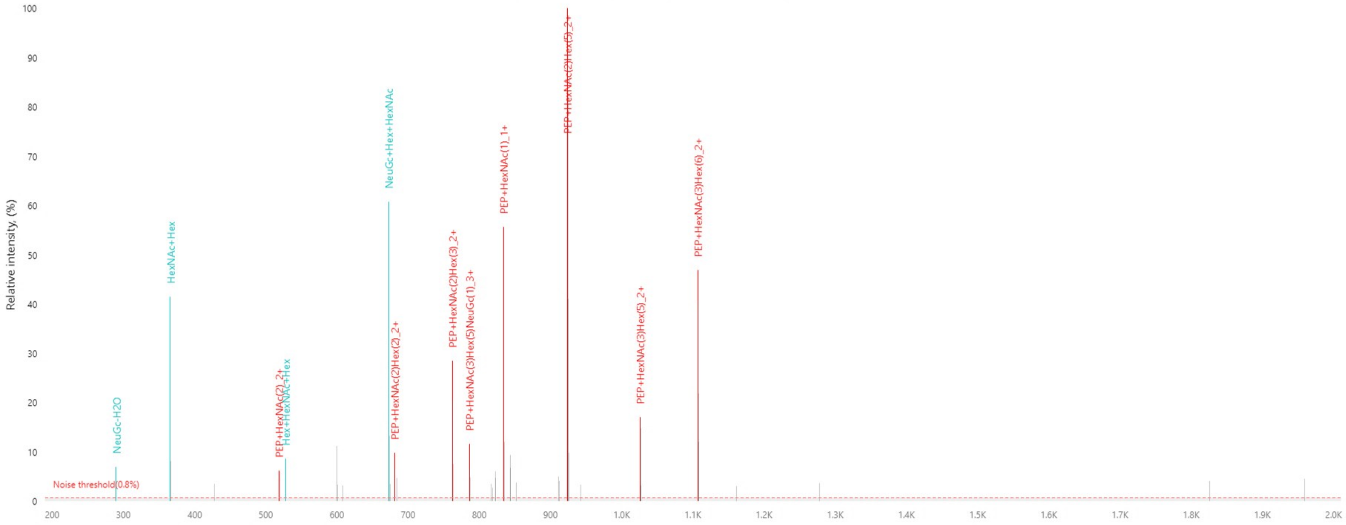

TNGSPR(=PEP)_7_2_0_0_0, m/z:1086.4261(2+), RT:8.76, HCD-score:93.59, Y-score:98.08, P-score:0.00,
HCD-MS/MS Scan:1247, SNR=0.8, Base Peak Intensity=18923.1

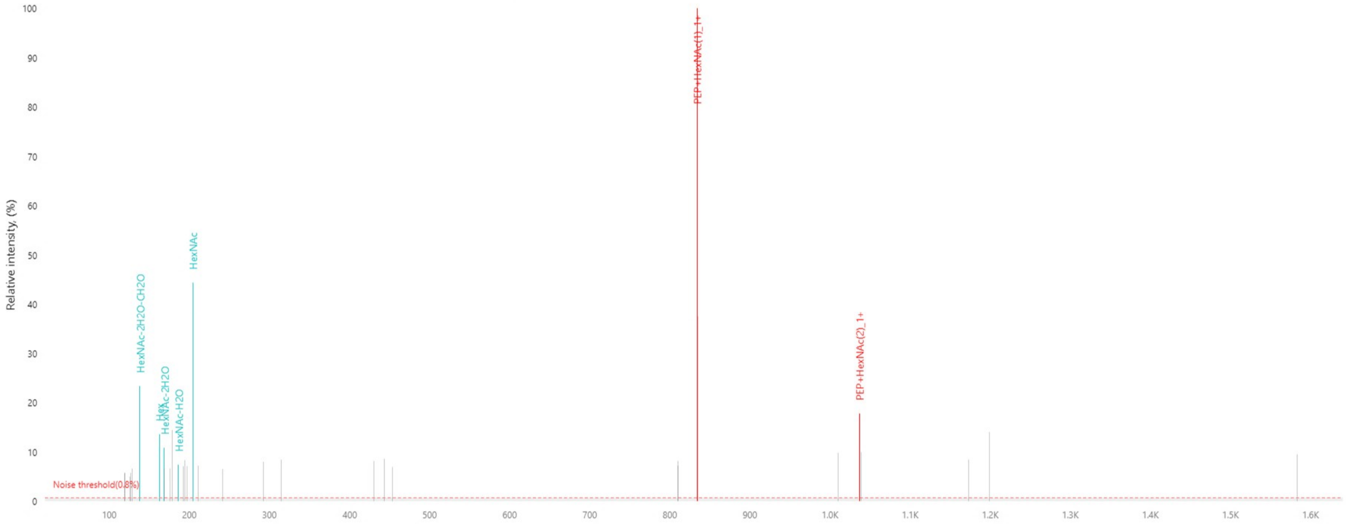

TNGSPR(=PEP)_7_2_0_0_0, m/z:1086.4261(2+), RT:8.76, HCD-score:93.59, Y-score:98.08, P-score:0.00,
CID-MS/MS Scan:1248, SNR=0.8, Base Peak Intensity=13109.3

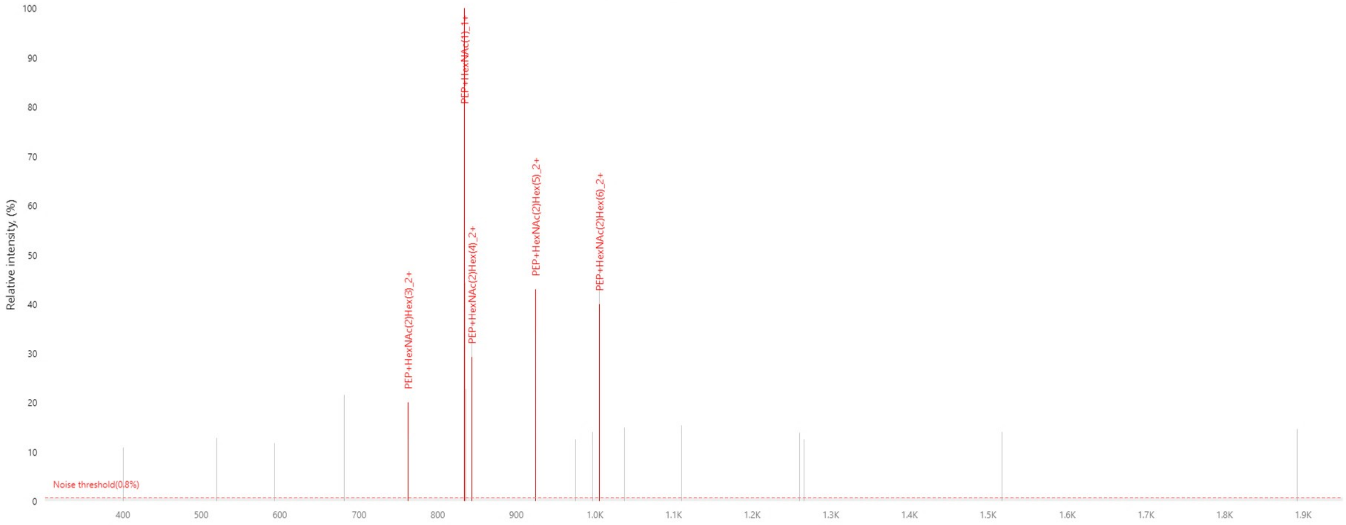

Supplement: Supplementary file 4 [file Image1.pdf]
